# Living Organisms Author Their Read-Write Genomes in Evolution

**DOI:** 10.3390/biology6040042

**Published:** 2017-12-06

**Authors:** James A. Shapiro

**Affiliations:** Department of Biochemistry and Molecular Biology, University of Chicago GCIS W123B, 979 E. 57th Street, Chicago, IL 60637, USA; jsha@uchicago.edu; Tel.: +1-773-702-1625

**Keywords:** genome rewriting, natural genetic engineering, symbiogenesis, holobiont, hybrid speciation, horizontal DNA transfer, mobile DNA elements, network rewiring, repetitive DNA formatting, ecological challenge

## Abstract

Evolutionary variations generating phenotypic adaptations and novel taxa resulted from complex cellular activities altering genome content and expression: (i) Symbiogenetic cell mergers producing the mitochondrion-bearing ancestor of eukaryotes and chloroplast-bearing ancestors of photosynthetic eukaryotes; (ii) interspecific hybridizations and genome doublings generating new species and adaptive radiations of higher plants and animals; and, (iii) interspecific horizontal DNA transfer encoding virtually all of the cellular functions between organisms and their viruses in all domains of life. Consequently, assuming that evolutionary processes occur in isolated genomes of individual species has become an unrealistic abstraction. Adaptive variations also involved natural genetic engineering of mobile DNA elements to rewire regulatory networks. In the most highly evolved organisms, biological complexity scales with “non-coding” DNA content more closely than with protein-coding capacity. Coincidentally, we have learned how so-called “non-coding” RNAs that are rich in repetitive mobile DNA sequences are key regulators of complex phenotypes. Both biotic and abiotic ecological challenges serve as triggers for episodes of elevated genome change. The intersections of cell activities, biosphere interactions, horizontal DNA transfers, and non-random Read-Write genome modifications by natural genetic engineering provide a rich molecular and biological foundation for understanding how ecological disruptions can stimulate productive, often abrupt, evolutionary transformations.

## 1. Introduction and Goals

Over the past 40 years, several books and numerous review articles have detailed the molecular mechanisms that cells utilize to alter their genomes [1,2,3,4,5,6,7,8]. These “natural genetic engineering” (NGE) processes are biochemical tools that living organisms possess to make adaptive use of their DNA databases as “Read-Write Genomes” [9]. Taking these mechanistic discoveries as established science, the goal of this review is to explore how bioinformatics has documented some of the ways that living organisms stimulate and benefit from NGE in the course of evolutionary change. Much of the relevant information will appear in lists and tables that have two objectives: (1) To make the primary literature accessible to the reader, and (2) to manifest how extensive the literature has become verifying that genome change in evolution results from a series of active biological processes, not from passive accidents. Previous reviews have summarized the outline of the basic arguments presented below [10,11,12], but this article presents each topic in greater depth and detail than earlier publications.

## 2. Parsing the Fundamental Question in Evolution: How Do Heritable Adaptive Novelties and New Groups of Organisms Arise?

What does it mean to say that living organisms author their read-write genomes? The answer encompasses a set of ideas about how and why genomes change in the course of evolution. The most basic idea is that evolutionary genome change results from biological activities, not from random accidents. The activities are both biochemical NGE functions that form new DNA sequences in genomes and cellular/organismal processes that lead to cell fusions, which alter genome content, and that regulate NGE. Considering genome change as an active biological process in these two ways makes it appropriate to consider the genome a “read-write” (RW) information storage component of the cell [9], different from the “read-only memory” (ROM) that is postulated by conventional evolutionary and molecular biological theories [13,14,15].

When Darwin first published his theory of evolutionary change involving modification with descent, he wanted to give pride of place as a causal mechanism to Natural Selection. Consequently, he had to reduce hereditary Variation to a minor and haphazard process. Thus, he wrote in Chapter 6, *Origin of Species*: “If it could be demonstrated that any complex organ existed, which could not possibly have been formed by numerous, successive, slight modifications, my theory would absolutely break down. But I can find out no such case.” [16]. Over time, however, Darwin came to recognize cases where hereditary variation had its own creative power that he had initially ignored. In later editions of *Origin of Species*, he wrote about natural “sports” or “…variations which seem to us in our ignorance to arise spontaneously. It appears that I formerly underrated the frequency and value of these latter forms of variation, as leading to permanent modifications of structure independently of natural selection” (6th edition, Chapter XV, p. 395) [17]. Since Darwin’s day, classical and molecular genetics have uncovered a dizzying array of internal NGE mechanisms that organisms possess for generating hereditary variation in their genomes [1,2,3,4,5,6,7,8].

Given the new knowledge about how living organisms actively change their genomes, it is appropriate to raise the basic question of evolutionary biology: what are the sources of adaptive change and taxonomic variation in evolution? Natural selection operates after the fact, as a test for the biological value of changes that have already taken place. By definition, natural selection cannot explain the *origins* of novel inherited characteristics. A related question about the processes of evolutionary change is whether important hereditary variations are limited, as Darwin initially argued, to “numerous, successive, slight modifications”, or can they occur abruptly and involve major changes in phenotypic characters? The question about gradual versus saltatory change has come up repeatedly in the history of evolutionary studies, and a number of distinguished evolutionary biologists have argued for abrupt major changes by various means (Table 1).

The evidence for the adaptive and genealogical importance of saltatory variations involving biological processes in evolutionary history is now overwhelming (Table 2). The symbiogentic merger of two different cell types or the hybridization of two different species must necessarily produce an abruptly distinct organism with a novel combination of multiple traits. Moreover, it cannot be emphasized too strongly that much of evolutionary change occurs interactively in the biosphere, not isolated within the discrete genomes of species that are completely separated from each other. The recognition that different organisms are interconnected at the cellular and genomic levels and that novel lineages frequently do not owe their new hereditary constitution to a strictly vertical process of inheritance has been transformational in our understanding of how evolution occurs. The current deluge of genome sequence data reveals DNA exchanges between diverse lineages at all levels of taxonomic diversification (Section 5). Moreover, genomics has heightened our awareness that the biosphere is populated by organisms that metabolize, behave, reproduce, and evolve interactively, not in isolation, bringing many biologists to abandon the abstract idea of the single-genome species and embrace the concept of the “holobiont”, an integrated amalgam of smaller and larger species, which exhibits collective phenotypic properties (Section 3.3) [18,19,20].

While most of this review will focus on molecular data, the conclusions are consistent with real time observations documenting unexpectedly rapid evolutionary changes among organisms in the wild. Among the most outstanding examples of such observations are those of Peter and Rosemary Grant’s four decades tracking Darwin’s finches *Geospiza* in the Galapagos Islands [21,22,23]. Their reporting of rapid modifications in *Geospiza* beaks following interspecific hybridization is particularly noteworthy because alteration of beak morphology was one of the paradigms that Darwin chose to exemplify the action of natural selection on minute, gradual phenotypic variations [16,22].

## 3. Biomath: One + One = One [24,25]; Ubiquitous Cell Mergers in Reproduction and Evolution (Reviewed in [26])

### 3.1. Symbiogenetic Origins of Eukaryotic Cells [26,27,28,29,30,31,32] and Their Photosynthetic Lineages

The ability of cells to merge, invade, and engulf one another is the basis of many basic biological processes that include endosymbiosis, sexual reproduction, phagocytosis, and pathogenesis [27]. Reviewing the genomic evidence, the highest-level taxonomic innovations that we can document involve interactions between distantly related organisms. In evolutionary history, molecular evidence clearly indicates that symbiogenesis underlies the origins of major eukaryotic lineages, as proposed by Mereschkowsky, Wallin, Kozo-Polyansky, and Margulis (Table 1). Symbiogenesis is the process by which an endosymbiotic microorganism loses its ability to reproduce outside the host cell and becomes an obligate intracellular organelle [33,34]. In addition, other simpler symbiotic events that do not involve the loss of autonomous reproduction contribute in multiple ways to eukaryotic evolution.

Although there is still uncertainty about whether the earliest eukaryotic ancestors possessed mitochondria [35], there is no doubt that the vast majority of extant eukaryotic lineages have oxidative mitochondria or related non-oxidative organelles, called hydrogenosomes and mitosomes [36,37,38]. Mitochondria contain DNA, carry out transcription and translation, and are clearly related in membrane, protein, and ribosome structure to *α-proteobacteria* [27,39,40,41]. Consequently, most (perhaps all) eukaryotic lineages originated in a symbiogenetic event involving an *α-proteobacterium*.

Photosynthetic eukaryotes have additional DNA-containing organelles that are labeled chloroplasts or plastids, which are related in their photosynthetic pathway, proteins, ribosomes, and membranes to photosynthetic bacteria [27,39,42,43,44]. Molecular analysis of photosynthetic eukaryotes distinguishes three major groups (Table 3):

(1) lineages that arose from **primary** *cyanobacteria* endosymbiosis with a non-photosynthetic eukaryotic cell: *Arachaeaplastida* = red and green algae, *Glaucophytes* = algae containing peptidoglycans, and green plants = *Embryophyta*;
(2) a separate lineage of photosynthetic amoebae *Paulinella chromatophora* that arose from a **primary** endosymbiosis with photosynthetic bacteria from the *Synechococcus*-*Prochloron* clade; and,
(3) highly diverse photosynthetic lineages that arose from a **secondary** or even **tertiary** endosymbiosis of a photosynthetic eukaryotic cell with a non-photosynthetic eukaryote.

While primary plastid symbiogenesis creates a cell with three genome compartments (nucleus, mitochondrion, and plastid), a higher-order photosynthetic symbiogenesis creates a cell with four or more genome compartments (nucleus, mitochondrion, plastid, and the “nucleomorph” descended from the photosynthetic partner’s nucleus).

The organic products of the abrupt symbiogenetic evolutionary events listed in Table 3 are multiple and diverse lineages of photosynthetic organisms with a wide range of phenotypes that occupy virtually all ecologies exposed to light (and likely some not exposed to light). In at least one photosynthetic lineage, the *Warnowiid* dinoflagellates, serial symbiogenetic events have been linked to the morphogenesis of a light-collecting organelle (“ocelloid”) that bears a remarkable resemblance to the camera eyes in animals, with a cornea composed of mitochondria and a retinal body formed by plastids acquired from a red alga [45].

Genomic analysis of mitochondria and plastids make it clear that abrupt symbiogenetic events with major physiological consequences for cell structure and energy metabolism were foundational in establishing the phylogenetic bases of eukaryotic evolution. In addition, genome evolution continued after the initial symbiogenetic event. There is abundant evidence for subsequent DNA exchanges from organelle to host nuclear genomes [46] and between mitochondrial and plastid genomes [47,48,49,50,51]. As a result of DNA transfers and rearrangements, coding content and physical organization diverge widely across taxonomic groups in plastid, as well as mitochondrial genomes [52,53]. Because of DNA loss from mitochondrial and plastid genomes and transfers to the nucleus, the large majorities of mitochondrial and plastid proteins are encoded by the host cell nuclear genome and are transported into the organelles. Moreover, while mitochondria and plastids contain nuclear-encoded proteins evolved from *α-proteobacteria* and *cyanobacteria* ancestors, they also contain proteins that derive phylogenetically from eukaryotes, from other bacterial lineages, and even from bacteriophages [48,54,55]. These findings highlight how deeply eukaryotic and organelle evolution reflect DNA exchanges across the biosphere.

### 3.2. Symbiosis as an Adaptive and Evolutionary Stimulus; Speciation by Endosymbiosis and Mating Incompatibility

As foreseen by Wallin, the combination of large eukaryotic hosts and their microbial symbionts (nowadays collectively labeled “the microbiome”) extend the adaptive capabilities of the resulting composite organism [56,57]. Well-known examples include the symbiotic acquisition of nitrogen fixation in legumes by *Rhizobium* root nodule formation [58,59], mycorrhizal fungi providing root functions for orchids, and other plants that are hampered in the ability to generate their own roots [60,61] and expansion of digestive and biosysnthetic capacities conferred by microbial symbionts in the animal intestinal tract [62,63]. Symbiotic associations are particularly important in the broad range of animals that live on plant material and depend on associated microbes to digest cellulose and other phytopolymers [64,65]. There are even photosynthetic metazoa that are formed by secondary algal symbiosis, including corals [66], sea slugs [67], and salamanders [68]. In these animal symbioses, the endosymbiotic algae retain the capacity for autonomous reproduction.

Clearly, the establishment of these symbiotic relationships represented both a quantum leap in host adaptive potential, as well as an expansion of the symbiont’s ecological range and evolutionary potential [69,70,71]. DNA analysis has expanded our knowledge of symbiotic relationships and has led to increased recognition that associated microorganisms (collectively, the “microbiome”) of humans and other macroscopic organisms have powerful impacts on phenotypes that were previously ascribed only to the host. Some examples include:

- embryonic development and metabolic homeostasis [72,73,74];
- immunity of multicellular organisms [75,76], such as the Hawaiian squid *Euprymna scolopes*, host to the marine luminous bacterium *Vibrio fischeri* [77];
- susceptibility to infection by bacteria, parasites and viruses [78,79,80]; and,
- higher nervous system functions and behavior [81,82].

The fact that these complex multicellular phenotypes do not purely depend on the expression of the host genome means that we cannot account for their evolutionary trajectories only by genetic changes within a single organism.

One particular phenotype that is influenced by microbial symbiosis can have direct effects on taxonomic divergence in host evolution. In invertebrates, bacteria belonging to the genus *Wolbachia* are common intracellular endosymbionts [83,84]. *Wolbachia* colonize germline cells [85] and influence sexual differentiation with profound effects on mating [86]. In *Drosophila* species, mosquitos [87], parasitic wasps [88], and other arthropods, the infection of males with *Wolbachia* can lead to “cytoplasmic incompatibility” and sterility in mating with *Wolbachia*-free females [89,90]. The mating incompatibility that is generated by *Wolbachia* infection thus genetically isolates two populations from one species and provides a trigger for “speciation by symbiosis” [91,92]. Intriguingly, the intensity of cytoplasmic incompatibility in *Drosophila* is subject to control by viral bacteriophage functions expressed from a WO prophage integrated in the *Wolbachia* genome [93].

The few examples just cited illustrate how the phenotypic and reproductive effects of symbioses provide important accelerations to the evolution of eukaryotic host organisms. These inter-organismal biological influences typically result in taxonomic diversifications below the genus level. Nonetheless, they illustrate the principle that was stated at the beginning of this review. It is essential to think about the evolutionary process as occurring in a biosphere where distinct organisms are continually interacting, not as a purely endogenous process limited to the genome of an isolated species. Indeed, we now recognize that the organism of classical theory, evolving solely by internal changes, has become a largely abstract idealization, divorced from the real world of microbiomes, infections, and other biosphere interactions.

### 3.3. Holobiont Evolution: Lamarckian Acquisition and Inheritance of Novel Traits

An even more radical view of host-symbiont interactions has been to go beyond the concept of isolated species altogether and consider organisms as “holobionts”, each a consortium of distinct cell types transmitted across generations [18,19,20]. In many cases, like the examples in Section 3.2 above, holobionts comprise macroscopic eukaryotic hosts and smaller associated microorganisms, both prokaryotic and eukaryotic. Examples include corals [94], plants [95,96], tsetse flies [97], and termites (with their numerous eukaryotic intestinal protozoa [98,99]). Hereditary transmission of the symbiotic microorganisms typically occurs by the incorporation into germ cells or by reinfection of the newly formed zygote or embryo [86,100,101].

The holobiont concept is not limited to macroscopic hosts and their microbiomes, but also extends to consortia composed purely of different microorganisms, such as microbial mats [102], stromatolites [103], and microbial communities at deep-sea hydrothermal vents [104,105]. It is interesting to note that, the holobiont concept historically was applied unconsciously to composite organisms like lichens before their multi-species nature was recognized [106,107,108,109].

From the holobiont perspective, gain or loss of one or more members of the heritable consortium constitutes an evolutionary transition, often with major phenotypic consequences. Such evolution has been demonstrated experimentally [110]. Since heritable adaptive traits can be gained by infection, an intriguing feature of holobiont evolution is that it proceeds by the hereditary transmission of acquired characteristics. Aphid acquisition of resistance to parasitic wasps and other useful ecological traits are an example of this process [111,112]. In other words, holobiont evolution effectively constitutes a Lamarckian process based on well-documented biological mechanisms.

Frequently, as in *Wolbachia*-stimulated speciation by symbiosis, a change in holobiont composition constitutes the initial step in a series of evolutionary transitions. These transitions include DNA transfers within the holbiont, both between different microbial constituents and between symbiotic microbes and the host nuclear genome [113]. These DNA transfers are analogous to those from symbiogenetic organelles to the nuclear genome mentioned in Section 3.1 above. There are now numerous examples of symbiont to host genome DNA transfers [114]. Aphid genomes have acquired sequences from the bacterial endosymbiont *Buchnera* [115]. In the well-studied arthropod-*Wolbachia* system, *Wolbachia* sequences have entered the genomes of mosquitoes [116,117], tsetse flies (*Glossina*) [118], beetles [119,120], wasps, honeybees, ticks, and pathogenic filarial nematode worms [113], as well as numerous *Drosophila* species [113,121]. In *Drosophila ananassae*, for example, more than 2% of total nuclear DNA sequences come from *Wolbachia* endosymbionts and include the entire bacterial genome [122,123].

## 4. “Cataclysmic Evolution” by Interspecific Hybridization

The cytogenetic study of cultivated plants in the first half of the 20th Century uncovered the highly significant fact that many crop genomes are in fact hybrids, combinations of two genomes inherited from a pair of related species. In 1951, one of the principle cytogeneticists doing these studies, and a leading evolutionary theorist at that time, G. Ledyard Stebbins, published an article in *Scientific American* on such hybrid species [124]. Stebbins labeled the process of hybrid species formation “Cataclysmic Evolution” and commented particularly on the fact that hybrids frequently have novel properties that are quite different from those of either parent:

“The remarkable fact about the wheat story is that the combination of chromosomes of a moderately useful plant, emmer wheat (*Triticum turgidum*), and those of a completely useless and noxious weed (goat grass, *Aegilops squarrosa*) produced the world’s most valuable crop plant (bread wheat, *Triticum aestivum*). This example should tell us that we cannot always predict in advance whether a particular hybrid will be worthless or a priceless new addition”. [124].

Contrary to the expectations of many conventional evolutionists, interspecific hybridization like that producing *Triticum aestivum* is not an exception but rather a common process that turns out to be a major factor in eukaryotic evolution [125].

### 4.1. Abundant Examples of Speciation and Adaptive Radiations by Interspecific Hybridization and Whole Genome Duplications (WGDs) in Plants and Animals

Stebbins explained in his 1951 article that, cataclysmic evolution actually involves two discrete biological activities: (1) mating between two related but distinct species (inter-specific hybridization), and (2) whole genome doubling (WGD) of the chromosomes in the resulting hybrid genome. WGD is essential to the accelerated generation of a hybrid species because eukaryotic chromosomes each need to pair with a homologous partner chromosome during the meiotic divisions that produce the haploid gametes needed for ongoing sexual reproduction. Experimental investigation indicates that hybridization itself, as well as other stresses and stimuli, trigger the genome replication events that are necessary for WGD [126,127]. The frequency of WGD events in the evolutionary histories of many eukaryotic lineages is indicative that inter-specific hybridization may have been a key factor in the origins of the new taxa [128]. Major taxonomic origins involving WGD include yeasts and fungi [129,130,131], ciliated protozoa [132], cereals [133], flowering plants [134], and crabs [135]. Vertebrate evolution, in particular, involved two successive WGD events [136,137], followed by further WGDs in fishes [138,139,140]. Intriguingly, WGD has been credited with the evolution of electric potential in fishes [141].

WGD is one feature of hybrid speciation that has captured the attention of geneticists that are interested in the establishment of novel regulatory networks in the course of evolution [142,143,144,145,146]. WGD provides at least two kinds of impetus for regulatory innovation. First, it doubles the content of loci encoding regulatory factors that are subject to modification and utilization in newly established mutant networks. Second, by duplicating the entire content of the genome, WGD assures that essential multi-locus functions can be maintained in one unmodified copy of the entire network, while individual components of the second copy are no longer essential, and thus are free for rewiring and evolutionary experimentation [147].

Abundant cytogenetic and genomic evidence has led to the conclusion that hybrid speciation has played a key role in the evolutionary diversification of a wide range of organisms (Table 4).

Two animal groups in Table 4 deserve special mention because they occupy key places in ongoing discussions about the nature of evolutionary processes: (1) The first one is *Geospiza*, Galapagos finches, whose beaks have served as primary paradigms for proponents of incremental gradualist evolution from Darwin on. Painstaking field study over four decades by Rosemary and Peter Grant [21], supplemented by genome sequencing [148,149,150], has clearly established the real time importance of introgressive hybridization in the adaptive evolution of beaks in this group. (2) The second paradigmatic group are the cichlid fishes of African lakes, which are considered exemplars of rapid vertebrate speciation and adaptive radiation [151,152]. Here too, hybrid speciation has proven to be critical to a remarkable phenomenon of accelerated and ongoing evolutionary diversification [153,154]. A further example of a role for hybridization in adaptive evolution comes from the generation of mimicry patterns by *Heliconius* butterflies [155,156,157].

The importance of introgressive hybridization between distinct populations in their evolutionary development is yet another factor that has led certain authors to point out that it is more appropriate to consider reticulate (net-like) over conventional branching (tree-like) models for inheritance by new species [158,159,160]. Altogether, we can see from the *Geospiza*, cichlid, and other cases like those cited in Table 4, that biological activity in the form of interspecific hybridization provides a key impetus to many (perhaps most?) paradigms of higher organism evolution [161,162,163,164,165,166]. So it is appropriate to look more closely at how such hybridization influences genome reorganization.

### 4.2. Genomic Consequences of Interspecific Hybridization

For practical reasons, chiefly ease of fertilization, experimental work on interspecific hybridization has been carried out more extensively in plants than in animals. Nonetheless, there is some work on experimental hybridization in certain animals, such as *Drosophila* and mice. Table 5 lists some of the genome expression and reorganization consequences that are observed in interspecific crosses of both plants and animals. The changes listed in Table 5 (ploidy, epigenetic alterations, new genome expression patterns, activation of mobile DNA elements and related genome rearrangements, karyotype modifications, and alteration of tandemly repeated DNA arrays) have all been associated with major novelties in adaptive phenotype and mating incompatibility.

It appears that a basic consequence of merging gametes from different species is to upset the established patterns of epigenetic regulation for the parental species, and that the breakdown in control then leads to the activation of multiple genome reorganization functions [168]. The activation of mobile DNA elements by interspecific hybridization is particularly relevant to the main argument of this review because these elements are primary biological agents for rewriting genome content [1,2,3,4,5,6,7].

### 4.3. The Special Genomic Impacts of Interspecific Hybridization on Evolutionary Innovation

In thinking about interspecific hybridization as a genome change process from the evolutionary perspective, it is helpful to point out how it differs from other mutagenic events that have been considered in traditional evolutionary thinking. Within-species mutations are generally limited to one or a small number of genome locations. As a consequence, they typically have effects on one or a few organismal characters. That is why conventional evolutionary theory has long postulated species change to be a gradual, cumulative, multi-generational process. In contrast, mergers between the complete genomes of two distinct species involve all regions of those genomes, and therefore have the potential to affect a large number of phenotypic traits in a single generation. That is the reason some authors have denoted interspecific hybrids to be the “hopeful monsters” postulated by Richard Goldschmidt (Table 1) [153,169,170,171,172,173].

All in all, the major role of interspecific hybridization in stimulating taxonomic and adaptive divergence exemplifies the importance of purely biological functions (mating, cell fusion at fertilization, epigenetic modification, and triggering of NGE activities) as primary agents of evolutionary variation. It requires little imagination to see how ecological deterioration and mating population declines can lead to an increased incidence of interspecific matings, and thus accelerate the evolution of new species, some of which may be better adapted to the altered ecology (Section 8.1).

## 5. Widespread Horizontal DNA Sequence Mobility between Organisms

A separate form of rapid evolutionary variation involves genomic communication between different (and often quite distant) species. This is the phenomenon known as horizontal or lateral DNA transfer, frequently abbreviated as HGT (“horizontal gene transfer”) [174]. We have already discussed one aspect of this phenomenon in Section 3, comprising DNA transfers from symbiogenetic organelles and endosymbionts to the host nuclear genome. But, horizontal DNA transfers are more widespread than those cases and have been documented to occur between all domains of living organisms [175,176].

### 5.1. Distinct Modes of Intercellular DNA Transfer

We have known for over half a century that bacteria possess three distinct mechanisms of transferring DNA between cells: transformation by uptake of exogenous DNA, transduction by encapsidation of DNA in bacteriophage (viral) particles, and conjugation by direct cell-to-cell contact (Table 6) [177]. These mechanisms mediate extensive DNA transfers across different prokaryotic lineages, both bacterial and archaeal, and similar processes have also been found to facilitate DNA exchanges from bacteria to eukaryotes, and even among various eukaryotes (Table 6). The molecular biology of these DNA transfer and uptake modalities is well understood. Both conjugal DNA transfer and DNA uptake by bacteria that are competent for transformation is based on the ability of the cells to elaborate complex cell-surface structures labeled “type IV secretion systems” that facilitate macromolecular transport across the bacterial cell envelope (they are also used by pathogenic bacteria during infection to introduce “virulence factor” proteins into target mammalian cells) [178,179,180,181].

A limited number of examples of regular bacteria to eukaryote DNA exchange have been well known for several decades, such as the conjugal transfer of specific “T-DNA” from *Agrobacterium tumefaciens* to plant cells during crown gall tumor formation [182]. The *Agrobacterium*-plant T-DNA transfer system also became well known for its biotechnology application as a tool for the genetic engineering of plants [183,184]. Nonetheless, despite our knowledge of various bacterial-eukaryotic cell interactions, it has been rather surprising to discover the extent and variety of naturally-occurring DNA transfers that occur across the widest taxonomic distances (Table 6). It has also been surprising to find that certain processes that were thought to be exclusively prokaryotic, like transformation, also occur naturally in eukaryotes [185].

In addition to these natural processes, investigators routinely use various techniques to introduce DNA into eukaryotic cells [186], including the modalities listed at the bottom of Table 6. It is probable that intercellular DNA transfer inside membrane vesicles will turn out to be more ecologically and evolutionarily important than we currently know [187,188]. The use of DNA uptake by sperm cells has proved to be easy, and, like T-DNA, has found broad biotechnology applications in the genetic engineering of animals [189,190]. Sperm-mediated DNA transfer illustrates the principle that horizontal transfer to multicellular eukaryotes encounters fewer obstacles at early stages of development, when genome isolation is less elaborated than in the mature organism [191].

### 5.2. Lessons on Rapid Evolution from the Smallest Living Cells

To fully appreciate the evolutionary potential of horizontal DNA transfer, we need to look back to the middle of the 20th Century, when humanity undertook a global evolution experiment by initiating the widespread use of antibiotics to combat bacterial pathogens. This experiment quickly demonstrated unforeseen evolutionary capabilities as pathogenic bacteria rapidly acquired resistance to one antibiotic after another, eventually creating the current “superbug” crisis of pathogens that are resistant to all known antibiotics [192]. When extensive antibiotic therapy began in the 1940s–1950s, there was a well-elaborated theory of how bacteria would evolve resistance: bacterial cells would mutate and alter cell structures so that they were less susceptible to antibiotic action or the cells became less permeable to the antibiotic [177]. The acquisition of streptomycin resistance by bacterial ribosome protein alteration is an example of this process [193]. Increased levels of resistance would result from the accumulation of successive mutations, and this multi-step mutational process could easily be confirmed for many different antibiotics in the laboratory [177,194].

Although the step-by-step mutational path for evolution of antibiotic resistance has been found relevant in isolated disease situations [195], this process did not explain most bacterial resistance. In the 1960s, it was discovered that the major source of clinically significant antibiotic resistance evolved in natural bacterial populations by the acquisition and spread of transmissible antibiotic resistance determinants [196]. This previously unsuspected evolutionary mechanism differed from the predicted (and experimentally confirmed!) mutational process in two significant ways: (1) resistance resulted from the synthesis of proteins that either inactivated or expelled the antibiotic from the target bacterium [193,197], and (2) resistance was encoded on plasmids, independently replicating genetic determinants, or other mobile DNA elements that were readily transferred to sensitive bacteria [1,198,199,200].

While the initial focus of studies on horizontal DNA transfer in bacteria was antibiotic resistance, it has become evident that DNA elements encoding many other important adaptive phenotypes in both bacterial and archaeal prokaryotic cells are subject to intercellular transmission [201,202]. The transfers occur across large taxonomic distances within Archaea and Bacteria, and between the two prokaryotic kindgdoms [203,204]. The prokaryotic functions subject to horizontal transfer range widely: metabolism and translation [205], mutagenic DNA repair [206], nitrogen fixation [207], and complex phenotypes like symbiosis [207,208,209] and infectivity/virulence [210,211,212,213]. Given what we know about the ability of prokaryotic cells to mobilize DNA both within and between cells, there appear to be no limits on the traits that can be transferred from one prokaryote to another. Genomic evidence indicates that such transfers have contributed to the evolution of major new Archaeal taxa [214,215,216].

Rapid prokaryotic evolution is further enhanced by specialized structures in the genome that have molecular features endowing them with the propensity to accumulate tandem DNA modules that are encoding related functions, thereby facilitating their joint transfer from one host to another. These specialized structures go by a variety of names: integrons [217,218], integrative conjugative elements (ICEs) [219], genomic islands (GIs) [220], mobilizable genomic islands (MGIs) [221,222], and the recently discovered GInts, a new type of mobile genomic island widespread in bacteria but not archaea [200]. The modes of genome integration and intercellular transfer of these compound elements vary [223]. As their name indicates, ICEs encode their own excision, conjugative transfer, and integration functions, whereas the MGIs encode their own excision and integration functions, but parasitize the conjugation apparatus of plasmids or other helper elements for conjugal transfer. Integrons and certain GIs may integrate into self-replicating transmissible plasmids, while some GIs and MGIs use recombination activities from the recipient cell to insert into the genome following conjugal transfer.

The ability to build up and transfer defined DNA regions encoding multiple proteins relating to a single phenotype enables the virtually instantaneous acquisition of complex adaptations by intercellular horizontal DNA transfer. These composite elements accrete protein-coding cassettes by using NGE activities related either to bacteriophage site-specific recombinases [224] or to mobile DNA element transposases [223,225,226]. They can vary in size from elements encoding a few antibiotic resistance determinants, to much larger structures, such as the 126 kb “super-integron” segment of the *Vibrio cholera* genome encoding 214 proteins (including virulence/pathogenicity functions) [227], the 502 kb “ICE*Ml*R7A” symbiosis island from *Mesorhizobium loti* [228], and the 674 kb PAI*St* pathogenicity island from *Streptomyces turgidiscabies* [229,230].

### 5.3. Horizontal DNA Transfer across Large Taxonomic Boundaries

The ability to receive DNA from different species provides numerous opportunities to acquire novel adaptive capabilities in all domains of life, not just prokaryotes (Table 7) [176,231,232,233,234,235,236,237,238,239]. The evidence for horizontal DNA transfer across large taxonomic distances comes from the phylogenetic analysis of genome sequence data [176]. A genetic locus or element is concluded to have entered an organism’s genome by horizontal transfer when sequence analysis finds the closest related examples in distant taxa, and the locus or element is absent from the genomes of taxa more closely related to the putative horizontal transfer recipient. For example, the phytopolymer digesting enzymes of plant pathogenic nematodes are most closely related to enzymes that are encoded by various bacteria and fungi than they are to enzymes with similar functions that are found in related nematode species [240]. Note that in this type of phylogenomic analysis, the recipient species can be identified with far more precision than the donor.

As Table 7 illustrates, there appear to be no absolute barriers to DNA transfers between organisms, and the genetic loci horizontally transferred frequently encode biochemical activities or functions adaptive for the lifestyle of the recipient organisms. This is clear in the example just cited and other cases, where the acquisition of microbial enzymes allows for animals to digest plant material as a food source and become herbivorous [240,241,242].

At the end of Table 7, there are several examples of horizontal transfers in both directions between viruses and various kinds of organisms. These cases were included to emphasize the point that the biosphere contains a large number of infectious agents that can be vectors for DNA transfer, and which may play a role in the origination of novel cell-based organisms [243,244]. The recently discovered “nucleocytoplasmic large DNA viruses” (NCLDVs) are particularly notable in this regard and have genomes in the megabase-pair range that readily acquire cellular DNA from all three major domains of life [245]. Many NCLDVs infect amoeba and other protists that are phagocytic, engulf other microorganisms, and also serve as hosts to bacteria like *Legionella*, *Helicobacter*, *Listeria*, and *Mycobacterium,* which also infect animal cells [246,247,248]. The amoeba-NCLDV-bacteria realm of the biosphere thus provides opportunities to mobilize DNA across multiple taxonomic barriers and, accordingly, has been characterized as a genomic “melting pot” [249,250].

In addition to DNA encoding specific adaptive traits, there is also abundant horizontal transfer of genome modification potential in the form of different classes of mobile DNA elements [251,252,253]. The horizontal transfer of mobile DNA is a further indicator of the range of genome connections between different organisms at all taxonomic levels: bacteria-eukaryotic microbes [254], metazoans-eukaryotic microbes [255], birds-nematodes [256], insect-mammal [257,258], mammal-insect [259], crustacean-insect [260], insect-insect [260,261], vertebrate-vertebrate [262,263], mammal-mammal [264,265], arthropod-plant [266], plant-plant [267,268], and vertebrates-invertebrates (and invertebrate viruses) [269,270,271].

All of the examples of horizontal DNA transfers presented above reinforce the two major themes that are articulated in Section 3. First of all, significant adaptive changes result from biological activities that mobilize DNA molecules between cells and across taxonomic boundaries. Secondly, the multiple genome connections that are documented across the biosphere add to the evidence showing how unrealistic has become the conventional view of evolution occurring solely by vertically inherited genome changes within an isolated species. Increasingly, we recognize that genome evolution results from a networked, or reticulate, process that connects multiple lineages [272].

## 6. Genome Writing by Natural Genetic Engineering—Protein Evolution by Natural Genetic Engineering, Exon Rearrangements and Exon Originations

Based on the central role of proteins in executing cellular biochemistry and determining adaptive organismal functions, a major focus of molecular evolution studies has been the dynamics of innovation in genomic protein-coding capacity. In Section 5, we saw how organisms could transfer the ability to synthesize different proteins across taxonomic boundaries. But, horizontal DNA transfer does not address fundamental questions of how living organisms originate and modify protein-coding sequences to confer novel or improved functionalities. While early theories of protein evolution were based on the sequential accumulation of individual amino acid changes, the last three decades have revolutionized our understanding of protein organization, protein coding in the genome, and cellular activities that reorganize and innovate protein coding DNA elements.

### 6.1. The Modular Domain-Based Structure of Proteins

Protein biochemistry and genomics have led to the recognition that the majority of proteins are not unitary functional entities, but are, instead, modular structures that are composed of distinct “domains,” each conferring a discrete aspect of overall protein activity [273,274,275,276]. For example, one domain may bind to a particular class of molecular substrate, another domain may contain the amino acids that execute the protein’s catalytic activity, a third domain may bind regulatory signaling molecules, and a fourth domain may connect the protein to other macromolecules in a multi-component complex at a defined subcellular location. In other words, each protein’s functionality is a combinatorial systems phenomenon reflecting the integrated properties of and interactions between its various domains.

Table 8 lists some of the most frequently cited protein domain search terms that are listed in the PubMed database (https://www.ncbi.nlm.nih.gov/pubmed/). While the kinase domain indicates a particular catalytic activity, it is significant that the other highly cited domains determine cellular localization or the specificity of interactions with other proteins, DNA, or lipids. The role of protein domains as combinatorial tools for molecular recognition, especially between proteins [277,278,279], makes them essential components in the elaboration of highly specific cell signaling [280,281] and regulatory networks [282,283]. Protein connectivity networks that are based on evolutionarily mobile domain modules exemplify the value of thinking about biological functionalities as information systems.

Each domain has its own phylogeny, and homologous domains can occur in functionally different proteins in combination with other divergent domains [284]. There are protein databases dedicated to identifying individual domains rather than complete proteins [285,286], and it is common practice in contemporary comparative genomics to describe a coding region and its cognate protein product by its domain content. While many domains are shared across broad phylogenetic distances, patterns of multi-domain architectures are specific to each taxon [287,288].

The modular nature of proteins as domain combinations means that basic protein evolution questions must be posed at several levels:

- How does a particular domain combination vary as it is transmitted within and between taxonomic groups?
- How do different combinations of domains assemble?
- How does each separate domain originate and enter into its various protein contexts?

Question (i) indicates that proteins evolve by gaining or losing domains [289], which occurs most commonly at either end of the protein chain [290]. Gain goes by the term “domain accretion” [291], and documentation of this process in the course of eukaryotic protein evolution from yeast through nematodes and *Drosophila* to man was highlighted in Figures 42 and 45 of the original publication of the draft human genome sequence [292]. Domain accretion is perhaps the most common mechanism for proteins to acquire additional activities or other properties, such as protein-protein interaction (Table 8). Domain loss is less well documented, and apparently represents a mode of protein specialization.

Question (ii) indicates that novel protein functionalities can arise rapidly by the aggregation of domains in particular combinations and that these combinations are key features in the evolutionary history of various taxa, such as metazoa [293], vertebrates [294], and plants [295]. Naturally, domain-coding sequences are subject to horizontal transfer [296]. Question (ii) further implicates the operation of molecular processes that join discrete domain-coding elements into combinatorial genomic determinants for adaptive functions [297,298]. These combinations include domain repetitions, which play a special role in molecular pattern recognition by proteins [299,300]. Repetitive domains are more common in eukaryotes than prokaryotes [301], but are found in all three kingdoms of cellular life [302]. Section 6.2 will discuss the various molecular mechanisms that organisms possess to assemble and rearrange domain combinations, a process that is labeled “domain shuffling”, which was highlighted in Figures 42 and 45 of the original publication of the draft human genome sequence [292]. It goes without saying that both domain accretion and domain shuffling are active examples of biological RW genome authorship.

Question (iii) posits mechanisms capable of generating a complex genetic coding element that did not previously exist. This may be the deepest question of all and represents the highest degree of RW genome creativity in protein evolution. We will discuss some of the currently recognized *de novo* domain origination processes in Section 6.3.

### 6.2. Protein Evolution by Exon Shuffling and Exon Accumulation, Changes to Alternative Splicing Patterns, and Insertion of Reverse-Transcribed Coding Sequences [303]

A major advance in understanding protein domain rearrangements in evolution was the discovery from early DNA sequencing studies that protein coding sequences in many eukaryote genomes are not continuous, but are rather composed of DNA segments that are expressed into partial protein chains (“exons”) separated by intervening non-coding DNA segments (“introns”) [304,305]. The segmented exons are stitched together into a continuous protein coding sequence prior to translation at the messenger RNA level by removal of the introns (“splicing”) [306]. Although the correspondence is not absolute, there is a high correlation between the boundaries of exons and of the encoded protein domains [307]. Thus, for organisms with discontinuous protein coding sequences, questions about domain behaviors in evolution can be reframed in terms of exon and intron behaviors at the DNA level. Virtually all of the studies on the processes underlying exon accretion, exon swapping, and exon origination have been done with those organisms. In our discussion, the term “genetic locus” replaces the more common “gene” to emphasize the composite nature of each coding region. As we shall see, a genetic locus may encode multiple protein products and thus not be a unitary genomic coding element, as the term “gene” generally implies.

A key feature of discontinuous protein coding is that mRNA splicing patterns are not necessarily fixed. The splicing apparatus can skip the splice sites (intron/exon boundaries) demarcating one or more exons in a particular coding region [308,309]. Consequently, alternative splicing of primary transcripts from a single locus can produce mRNAs containing different exon combinations encoding proteins with different domain architectures [310]. Alternative splicing thus enriches the protein repertoire [311], and it is intricately regulated in response to diverse ecological [312,313] and developmental signals [314,315].

Alternative splicing patterns evolve and confer different coding potentials on duplicated loci within a genome [316]. A recent paper ascribes a major role to alternative splicing in the evolution of phenotypic novelty [317]. Insertions of mobile DNA elements into introns frequently induce alternative splicing changes [318,319], and the acquisition of new introns has a similar effect [320]. As with other features of genome function, hybrid speciation and WGD events frequently lead to new patterns of alternative splicing, thereby diversifying the expression of duplicated copies of multiple genetic loci in a single episode of accelerated genome modification [321,322]. The effects of local duplications and WGDs on the evolution of domain architectures have been analyzed in yeast [323], *Drosophila* [324], and plants [325].

The enrichment of alternative splicing patterns is particularly characteristic of vertebrate evolution, which was apparently stimulated by the two successive WGD events at the origins of the vertebrate lineage [136]. These WGDs may explain why vertebrates display more variation in alternative splicing than do invertebrates [326]. A study of O_2_-binding globin domains in various hemoglobin, myoglobin, and cytoglobin proteins that are encoded by vertebrate genomes implicated the two successive vertebrate WGDs in the evolution of a broad diversity of vertebrate oxygen transport systems [327]. In addition, various globin proteins have evolved domain fusions and globin domain repeats [328].

Domain rearrangements, protein fusions, and protein splits have been documented by genomic analysis in prokaryotes (“3.8 million potential fusions across 11,473 genomes”) [329], fungi [330], *Drosophila* [331], metazoa [332,333], and the human genome [334,335], as well as in the virosphere [336]. In addition to shuffling exons from cell genomes, the virosphere provides an external source of exons encoding protein domains that are not found in cellular organisms [337].

New domain/exon arrangements arise by a wide variety of natural genetic engineering (NGE) processes. DNA level exchanges can generate fused genetic loci combining exons to encode chimeric proteins [338]. Exclusively at the DNA level, NGE can involve non-allelic homologous recombination (NAHR) events between dispersed intronic repeat elements in the genome [339] or non-homologous recombination between introns [305,340]. Non-homologous intron-intron recombination can occur during repair of DS breaks by the well-studied process known as “non-homologous end-joining” (NHEJ) [8,341,342].

While we are accustomed to thinking of evolutionary change as a DNA level process, it is difficult to overstate the importance of reverse transcription-based events in eukaryotic evolution. Many novel domain arrangements have been found to originate at the RNA level and involve the insertion of reverse-transcribed cDNA copies into the genome (“retroposition”) to generate loci encoding chimeric fusion proteins [343,344,345,346,347,348,349,350,351,352,353,354]. Retroposed coding sequences have been documented in nematodes [355], multicellular green algae *Volvox carteri* and *Chlamydomonas reinhardtii* [356], plants [357], silkworm [358], non-mammalian chordates [359], zebrafish [360], mammals [361], and primates [362,363]. Retrocopying is reported to have played a special role in the evolution of highly variable sex chromosomes [364]. Mammalian evolution has been impacted by episodic bursts of retrocopy formation [365]. One of these bursts occurred in the primate lineage that was leading up to the appearance of humans [366]. In primate genomes, there are approximately 17,500 retrocopies, of which, at least 3600 are expressed, many with “tissue-specific and even species-specific expression patterns” [367].

The innovative role of reverse transcribed cDNAs is further enhanced by the well-documented phenomenon of “trans-splicing”, which involves the joining of sequences from two distinct transcripts [368,369,370,371,372,373,374,375,376,377,378,379]. Genome insertion of cDNA reverse-transcribed from a trans-spliced RNA molecule generates a novel chimeric domain coding sequence [379,380]. The locus encoding the chimeric Jingwei protein in *Drosophila* apparently arose in this manner by adding exons to a processed retrocopy of the alcohol dehydrogenase (*Adh*) mRNA [381], and trans-splicing-generated protein rearrangements have been significant in vertebrate evolution [382].

In addition to the NGE processes outlined above, diverse mobile DNA elements, both DNA transposons and retrotransposons, play active roles in exon rearrangements (Table 9).

### 6.3. Protein Evolution by Domain/Exon Origination

Exon shuffling and alternative splicing generate functional protein novelties by rearranging existing coding elements. While those Lego-like processes have played important roles in evolutionary variation, they do not tell us anything about how novel domain coding sequences arise in the first instance. This is a topic where our ignorance is much greater than our knowledge. Conventional gradualist evolutionary theories would postulate that the novel domains emerge by the accumulation of nucleotide substitutions converting an exon encoding one sequence of amino acids into an exon encoding an entirely different polypeptide. While there is abundant evidence that related exons often undergo minor sequence changes that modify the encoded amino acid composition, often in adaptive ways (e.g., producing changes in DNA- or protein-binding specificities), there is genomic evidence indicating that other processes generate new exons more rapidly from both coding and non-coding precursor sequences.

The de novo origination of novel domains or complete novel protein-coding sequences is indicated in genome databases by the presence of so-called “orphan” coding sequences (also called ORFans, from ORF = “open reading frame” or uninterrupted polypeptide coding sequence) [383,384]. These are sequences that appear abruptly in the phylogenetic record without any apparent homology to sequences in ancestral lineages; consequently, they have a restricted taxonomic distribution. Orphan coding sequences were initially noted in the yeast genome, but have subsequently appeared in every class of sequenced genome (Table 10), and there is an ORFan database [385]. It is possible that many orphan sequences do not actually indicate de novo coding sequence originations, but instead appear phylogenetically isolated due to gaps in the sequence databases. In particular, unknown coding sequences may abruptly appear due to horizontal DNA transfer from an as yet unsequenced genome [386]. Nonetheless, there are many well-documented examples where the appearance of novel coding sequences can be traced back to their origins in the genome. The orphan examples in Table 10 indicate a variety of ways that coding and non-coding sequences can serve as precursors for novel exons:

- “Overprinting” of an existing exon by translating the sequence in a new reading frame [387];
- Cooption of the antisense strand from an expressed locus [388];
- Loss of a stop codon at the 3′ end of a terminal exon, thereby extending the protein C terminus by continuing translation into previously non-coding sequence [389];
- Cooption of non-coding but transcribed sequences [390,391], especially transposable elements [392,393,394]; and,
- Retroposition [356,395,396], frequently from non-coding RNA [391,397], which may account for some ORFans arising from non-coding transcribed regions [390]. The novel coding potential of long non-coding RNAs has been cited as a general phenomenon [398], and we will see in Section 7 how abundant these RNAs are in eukaryotic genomes.

The importance of mobile DNA elements in protein evolution was initially recognized when Nekrutenko and Li demonstrated the presence of numerous transposable element contributions to the coding sequences for many human proteins [399]. Since then, the discovery of mobile DNA sequences contributions to proteins has subsequently expanded (Table 11), especially in the human genome [400]. In addition to providing novel protein-coding exons, mobile element-derived non-coding exons can also modulate transcription and confer novel tissue-specific splicing patterns on human transcripts [401]. The relatively frequent exonization of segments from certain transposable elements results from the presence of cryptic splice sites that are embedded in their sequences [402]. But not all cases of exonization from a particular element are identical, and the same element can contribute different sequences to novel coding loci, as exemplified by the many cases of *Alu* exonization that are found in primate and human genomes [403,404,405,406].

The heavy bias towards transposable element exonization in the generation of novel protein coding-sequences is probably due, at least in part, to the overwhelming emphasis in genomic studies on the analysis of the human genome, where *Alu* elements, other SINEs (such as *SVA*), and LINE elements predominate in the retrotransposed fraction of the genome [292,344,407,408,409]. Nonetheless, the few examples that are documented in Table 10 and Table 11 of transposable element exonizations in algae, plants, and non-primate vertebrates do hint at something special about transposable elements as sources of novel coding potential. A number of authors have speculated that mobile DNA elements play dedicated roles in providing rapid genomic variability during episodes of evolutionary change (e.g., [410,411]). This view is buttressed by the well-documented role that mobile DNA plays in rewiring genomic structural and regulatory networks as evolution produces increasingly complex multicellular organisms (Section 7).

## 7. Genome Writing by Natural Genetic Engineering: Mobile and Repetitive DNA Elements Actively Contributing to Genome Organization, Organismal Complexity and Genome Regulation

Our view of what genomes are has changed fundamentally since genetics and evolution first combined in the early 20th Century to form the Modern Synthesis [412]. At that time, geneticists could only examine genome function by the mutational analysis of phenotypes, recombinational mapping, and cytological examination of chromosome structure and behavior. The nature of genome analysis changed in the middle of the 20th Century, beginning with a series of key discoveries: DNA as the carrier of genetic information [413], the DNA double helix [414], regulation of DNA-encoded protein synthesis [415], and the existence of mobile DNA elements that were capable of altering genome structure and patterns of genome expression [416]. This section will review some consequences of these discoveries from a conceptual perspective leading to a detailed empirical discussion of the key roles that mobile repetitive DNA elements play in evolutionary variation.

### 7.1. Regulatory Studies Led to Recognizing the Syntactical Organization of Genomes

In the early days of molecular biology, there was a temptation to simplify genome functions to replication and protein coding: DNA makes more DNA and also RNA, RNA makes protein [13,14,417]. However, the reductionist view of genome action started to dissolve even as it was being formulated. Beginning with the study of how cells control protein synthesis in response to nutritional changes [415], it rapidly became clear in the 1960s and 1970s that there exist in bacteria, and in all organisms, elaborate multifactorial genomic codes that adaptively regulate the transcription of DNA sequences into RNA (e.g., [418,419]). As regulation studies extended to more complex eukaryotic organisms, the generic term “transcription factor” was adopted for proteins that interact with countless distinct binding sites to construct appropriate multimolecular complexes connecting coding sequences with signaling networks, and thereby controlling genome transcription [420,421].

With increasingly detailed studies of genome regulation in yeast and other eukaryotic organisms, the phenomenon of “epigenetic” control revealed further codes for organizing genomes into extended “euchromatic” and “heterochromatic” domains that were, respectively, accessible or inaccessible for transcription [422,423]. The application of sequencing-based technologies to genome function during the cell cycle and multicellular development uncovered yet more codes governing the three-dimensional organization of genomes during cellular differentiation [424,425,426]. From these, and many other lines of molecular biology research, it has become abundantly clear that every genome is a densely formatted molecular database, syntactically organized to respond appropriately to the dynamic conditions of cellular life.

### 7.2. Repetitive DNA Elements Provide Distributed Copies of Each Class of Regulatory Site

Contemporaneously with the early expansion of genome regulatory studies, purely physical studies of DNA extracted from different organisms unexpectedly revealed the presence of significant fractions of multi-copy repeated sequence components. These repeated sequences were present at higher concentrations than unique sequences, such as those encoding proteins. They were detected because single strands that were carrying repetitive DNA sequences renatured to a double helical configuration more rapidly following denaturation than single-copy sequences [427,428,429]. By closely following DNA renaturation kinetics, it was possible to estimate the fractional composition of repetitive sequences in DNA extracted from any particular organism. The fraction could be considerable. Advances in whole-genome sequencing technologies over the last two decades enabled far more precise measurements of total DNA content and the fractional contribution of different classes of DNA sequence components, in particular, genomes [430,431,432].

Special attention has been paid to repetitive DNA elements in genome sequencing studies [433,434,435]. Data on animal genomes from one database shows a wide variation in repetitive DNA content across taxa (Table 12). Repeats include tandem arrays of DNA at centromeres, telomeres, and loci encoding structural RNAs for ribosomes and nucleoli, but in almost all species, the vast majority of repetitive DNA consists of “interspersed” mobile DNA elements distributed throughout the genome (the fractions in parentheses in Table 12). In mammals, generally considered the most highly evolved animal group, at least one-third of the genome consists of repetitive DNA, and some primates (including humans), as well as other mammalian species have more than half of their genomes as repetitive sequences [408]. Other vertebrate genomes vary from a low of 1.2% in spotted gar, a primitive fish, to almost 38% in reptiles [436,437,438,439,440].

Repetitive DNA elements that do not encode proteins had no place in classical genetic and evolutionary theories, and so were labeled “junk” or “selfish” DNA by some prominent scientists [441,442]. Nonetheless, the potential significance of repetitive DNA elements for establishing distributed regulatory networks became evident to certain molecular biologists soon after their discovery by renaturation kinetics [429]. Today, the recognition of repetitive DNA elements as genome formatting entities occupies a key role in the analysis of sequence data and functional genome organization by large-scale genomics projects like ENCODE (Section 7.4) [443]. From a theoretical perspective, maximum computational efficiency (e.g., to minimize the total number of distinct DNA recognition proteins) requires that the diverse DNA elements that control genome behavior must function similarly at many different genomic locations [444,445,446]. Thus, numerous copies of many distinct classes of DNA sequence must be present throughout each genome [447,448]. Even an organism with one of the smallest known cellular genomes, *Mycoplasma genitaleum*, contains repetitive DNA [449].

By contrast with the large fractions of repetitive DNA in most eukaryotic genomes (Table 12), the protein-coding DNA content is often rather modest (under 1.5% in the human genome [450]). A semi-quantitative proxy for repetitive DNA involvement in evolutionary innovation turns out to be the fraction of the genome that is composed of non-protein-coding DNA. Unlike protein-coding DNA sequences, which peak at 10^7^–10^8^ base-pairs per genome for all of the organisms sequenced, non-coding DNA sequences per genome continue to increase and scale with organismal complexity (measured by number of distinct cell types) up to levels as high as or greater than 2–3 × 10^9^ base-pairs per genome [450,451]. This remarkable correlation means that the most complex organisms have the largest amounts of repetitive and non-coding DNA in their genomes. In other words, the organisms we assume to be the most highly evolved have apparently gained much of their complexity by accumulating non-coding DNA content rather than increasing the protein-coding capacity. This pattern indicates that non-coding DNA abundance contributes to the complexity of biological control circuits, both through well-documented roles as mobile transcriptional signals in the genome (Section 7.4), and, in other, more recently discovered, ways. As Section 7.5 explains, precisely such an unpredicted connection between mobile DNA repeats and biological control circuitry has appeared in the last two decades.

### 7.3. How Do Organisms Use Repetitive DNA for Genome Rewriting in Evolution? Dispersed Mobile DNA Elements

Conventional theories of evolutionary change based on the accumulation of random mutations due to copying errors in genome replication could not account for adaptive change in the repetitive DNA content of genomes. But, a strikingly appropriate (if at first poorly appreciated) solution was available in Barbara McClintock’s discovery of mobile genetic elements in the maize genome [416,452]. These mobile components could transpose to novel locations, and thus accumulate throughout the genome [453]. Because they altered the patterns of expression from genetic loci where they were inserted, McClintock named her discoveries “controlling elements” [454]. She recognized that they could generate novel regulatory networks in the nucleus and pointed out parallels with the transcriptional control systems that were being studied in bacteria [455,456].

The discovery of analogous mobile DNA elements in bacteria, yeast, *Drosophila*, and eventually all of the nspecies examined, made it clear by the last two decades of the 20th Century that living organisms possess the molecular tools to reformat their genomes by actively mobilizing repetitive DNA [1,2,3,4,5,6]. Any skepticism about the functionally adaptive role of mobile repetitive DNA elements has been answered by the accumulation of sequence data establishing connections between these elements and functional regulatory motifs in genomic DNA. The human genome is naturally the most intensively studied. In our cells, mobile genetic elements contribute to DNAseI-hypersensitive sites [457], transcription factor binding sites [458], enhancer elements [459], RNA splicing control [460], epigenetic control of transcription [461], and the establishment of the core human embryonic stem cell regulatory network [462].

There is a rapidly growing literature that is dedicated to the central role this class of mobile supposedly “non-coding” DNA plays in evolutionary variation in general [463], as well as in specific phylogenetic groups (Table 13). Repetitive DNA elements also play major roles in restructuring genome architecture. In part, this role results from providing many dispersed sites for homology-dependent recombination, but mobile DNA activity also generates rearrangements at non-homologous sites [3]. The role of a taxonomically-specific retotransposon was recently invoked to explain the rapid karyotype evolution distinguishing the Gibbon genome from other primates [464]. In other cases, speciation events have been attributed to mobile element activity: *Arabidopsis* [465], fish [466], *Anopheles gambiae* mosquitoes [467], and volcanic island radiations [468]. A number of contemporary evolutionary theorists have even come to ascribe a primary role to mobile DNA elements as drivers of evolutionary transitions [244,468,469,470].

### 7.4. Rewiring Transcriptional Regulatory Networks in Evolution of Complex Organisms

By virtue of their abundance and the capacity to move throughout the genome, mobile DNA elements have specific properties necessary for rewiring and innovating transcriptional regulatory networks in evolution. They are not tied to any specific phenotype and can reposition promoters, enhancers, heterochromatin markers [471], insulators [472], splicing signals, and other *cis*-acting control elements that are embedded in their sequences [463,473,474]. It is noteworthy that biological triggers of speciation like interspecific hybridization and genome doubling activate mobile elements (Table 5) [475], as do a wide variety of ecological stressors [476] (http://shapiro.bsd.uchicago.edu/StimuliDocumentedActivateNGE.html). Consequently, network rewiring by mobile elements can connect to episodes of biosphere and ecological challenge. The literature contains a number of articles attributing evolutionary network innovation to mobile genetic elements in fungi, plants and animals (Table 13).

There is particularly compelling quantitative data on the roles of mobile DNA elements in evolution of certain well-studied mammalian regulatory networks [477,478,479]. A 2013 analysis of data from various human cell cultures found both phylogenetic and functional specificity of mobile DNA control regions: transposable elements contained 63% of the primate-specific sites for open chromatin (i.e., actively expressed regions), and fully 80% of the endogenous retrovirus (ERV) sequences in the human genome formed open chromatin in a cell type-specific manner, which was frequently associated with cell type-specific expression of neighboring genetic loci [480]. A bioinformatic approach to identify transcription factor binding sites found that approximately 110,000 are located in human ERVs or LTR retransposons [481]. Looking at specific stages and features of the mammalian life cycle provides a more detailed perspective on the roles of different types of mobile DNA in wiring regulatory networks, as well as giving an idea of how the literature has expanded in recent years, documenting the outcomes of natural genetic engineering in evolution.

#### 7.4.1. Embryonic Stem Cells

A 2015 study of human embryonic stem cells (hESCs) identified fully 99.8% of the candidate human-specific transcription factor-binding sites within human-specific retrotransposable element-derived sequences, most notably LTR7/HERV-H, LTR5_Hs, and L1HS [459,482]. These observations confirmed the earlier reports that species-specific retrotransposons are (i) enriched in hESC-specific hypomethylated regions of the genome [483] and (ii) bind both transcriptional activators and repressors to shape hESC transcription [484,485]. A recent study analyzed the mouse-specific RLTR9 family of endogenous retroviruses (not present in rats), and found that a significant fraction bound one and frequently several ESC-specific transcription factors, and experimentally verified that these mERVs provided an enhancer function in both synthetic constructs and for nearby ESC-specific loci [486]. When differentiated, mouse embryonic fibroblasts, human CD34(+) cells, or human primary hepatocytes are reprogrammed to become induced pluripotent stem cells (IPSCs), expression of all the endogenous retroelements is up-regulated so that their transcriptional profiles come closer to that of ESCs [487].

#### 7.4.2. Early Embryonic Development

A 2017 analysis of 259 mouse embryonic cells at different stages from zygote to blastocyst reports enrichment of mobile DNA in expressed promoters and stage-specific utilization of different classes of mobile element [488]. At the 2–4 cell stage, LTR retrotransposons predominate and provide binding sites for homeobox transcription factors. For subsequent zygotic transcription upregulation, *B1* and *B2* SINE retrotransposons come into play as enhancer elements, paralleling the results for human *Alu* and bovine tRNA SINE elements in embryonic stem cells. A prior study similarly found that conversion of human embryonic stem cells (hESCs) to an epiblast-like state activates hundreds of blastocyst-specific hERV elements containing splice sites for linking LTR initiation transcripts to nearby exons for epiblast-specific functions [489].

#### 7.4.3. Both Sides of the Fetal-Maternal Interface in Viviparous Reproduction

The ***placental*** tissue necessary for prenatal embryonic development depends on endogenous retroviruses for syncytial trophoblast development, as well as transcriptional programming. The essential “syncytin” proteins that fuse the cell membranes and provide an immune-protective barrier in the placenta are actually endogenous retroviral Env (envelope) proteins that are expressed from different ERV families in each mammalian order: “…the capture of *syncytin* or *syncytin*-like genes, sometimes as pairs, was found to have occurred independently from different endogenous retroviruses in diverse mammalian lineages, such as primates—including humans—muroids (rodents), leporids (rabbits), carnivores, caviids (S. American rodents), and ovis (sheep), between around 10 and 85 million years ago” [490]. At the transcriptional control level, in human placenta, there is hypomethylation of retrotransposon promoters for placenta-specific transcription [491]. Like *Env* exaptation for *syncytin* function, placental utilization of ERV promoters is also taxonomically-specific. In mouse, where detailed analysis of placental expressions patterns can be done, the species-specific ERV family RLTR13 contributes hundreds of enhancers for development of placental trophoblast stem cells (regulating over a third of all placenta-specific transcripts) [492]. Taxonomic specificity was so strong that a large majority of the mouse ERV enhancers were absent from rats.

The maternal or ***uterine*** side of viviparous reproduction displays a similar dependence on mobile DNA, but the elements are different. DNA transposons in the MER20 family provide enhancers for 200 uterine functions stimulated by progesterone and cAMP [493]. When all uterine-expressed Progesterone Receptor binding sites are tabulated, 1721 are found within Mammalian- or Eutherian-specific mobile DNA elements [494]. Although universally produced in the mammalian pituitary gland, certain mammalian orders have independently evolved the capacity to express the lactation-stimulating hormone prolactin from the uterus as well. Prolactin is not expressed from the uterus in rabbits, pigs, dogs, or armadillos, but it is in primates, mice, and elephants. Focusing on the promoter for uterine expression of decidual prolactin (dPRL) provides an interesting view of convergent mobile DNA behaviors in mammalian evolution. The human and spider monkey primate *dPRL* promoter contains a MER20 DNA transposon, as well as a MER39 LTR retrotransposon [495], while the mouse *dPRL* promoter derives from the MER77 LTR retrotransposon, and the elephant *dPRL* promoter originates from the lineage-specific LINE retrotransposon L1-2_LA [496].

These examples in mammalian viviparous reproduction of similar transcriptional wirings accomplished by non-homologous, species-specific families of mobile DNA elements exemplify an intriguing process of convergent evolution that remains to be explained. Do distinct mobile elements come to execute functionally equivalent transcript rewiring in different mammalian orders purely by chance, or are the elements responding to some as-yet-to-be-defined regulatory process that guides the adaptive integration of newly established regulatory signals? It would be a useful exercise to calculate realistic probabilities for random insertions of mobile DNA elements into the appropriate genomic positions. Such calculations would allow for us to see whether that process constitutes a realistic basis for the repeated evolutionary accumulation of taxonomically-specific transcriptional circuits involving mobile DNA families. At the same time, we need to keep in mind that regulatory guidance of mobile DNA activity is not implausible because the targeting of mobile DNA insertions within genomes is a well-documented process (http://shapiro.bsd.uchicago.edu/ExtraRefs.TargetingNaturalGeneticEngineeringInGenome.shtml; http://shapiro.bsd.uchicago.edu/Targeting_retroviral_and_retrotransposon_insertions.html).

#### 7.4.4. Brain and Nervous System Development

Since it was first proposed [497], several connections between SINE retrotransposons and mammalian neural development have been observed. A 2015 article reports the utilization of evolutionarily ancient MER130 SINEs to form an integrated enhancer network for embryonic development of mouse dorsal neocortex [498]. MER130 SINE elements are present in *Xenopus laevis* amphibians and reptiles (1135 copies in the green sea turtle genome) but not in fish, and so they arose close to the emergence of quadrupeds in vertebrate evolution. In the mouse, 23 of the total 90 MER130 copies in the genome act as experimentally verified developmental enhancers in the embryonic hindbrain, but not in the forebrain. The involvement of 24% of all genomic MER130 repeats in the dorsal brain development represents a 73-fold enrichment over random association with hindbrain-specific functions. The conclusion from this correlation is that the ancient MER130 elements were co-opted for this particular developmental function. That result is consistent with a 2016 survey analysis of mammalian neocortical enhancers that found a 30% overlap with mobile DNA repeats for eutherian-specific enhancers in more advanced mammals, as compared to less than a 6% overlap of older enhancers shared across all mammals [499].

A fascinating 2013 report links SINE elements to the epigenetic regulation of transcript elongation in the somatosensory cortex of young mice when they respond to novel enriched environmental (NEE) cues [500,501]. NEE stimulation modifies dendritic growth and synapse formation in brain neurons, providing a cellular basis for learning from experience [500]. The regulatory SINE elements were found located distal to NEE-induced promoters and embedded in silent chromatin that is subject to histone acetylation after NEE exposure. NEE-triggered acetylation relocates the SINE element chromatin to active transcription factories, and thereby facilitates the expression of NEE-induced proteins [472,500,502].

The role of SINE elements in the epigenetic control of neural transcript elongation in response to experience illustrates how versatile mobile DNA elements can be in regulating genome function, and how they can contribute to a higher nervous system phenotype like learning from experience [503,504]. A further connection between non-LTR retrotransposons and mammalian nervous system differentiation has been suggested to lie in activation of LINE-1 element retrotransposition. LINE-1 elements are silent in virtually all mammalian tissues, but they become active and retrotranspose to new locations in neurons [505]. This genome mobility creates somatic diversity within the nervous system and may prove adaptive by adding to neural complexity.

#### 7.4.5. Innate Immunity

There are multiple connections between the mammalian immune systems and mobile DNA repeats. Although our topic here is the rewiring of transcriptional networks, it should be noted in passing that the ability of the adaptive immune system to cut and splice DNA to generate antibody diversity by VDJ joining of variable (V), diversity (D), and join (J) coding segments descended directly from the transposase activity of a DNA transposon belonging to the *Transib* superfamily, originating in primitive metazoans [506,507]. While adaptive immunity in vertebrates uses mobile DNA functions to tailor antibodies specific to each particular invading organism or virus, the innate immune response in both plants and animals produces a generic response to infection that helps neutralize many different invaders [508,509].

In mammals, a key feature of innate immunity is a complex interferon (IFN)-dependent inflammatory response that attacks both invading pathogens and the host cells that they have infected to combat the spread of infection [510]. A recent study in human cells identified 962 MER41 family primate-specific endogenous retroviral elements as binding sites for IFN-triggered transcription factors [511]. Using CRISPR knockout technology, the authors directly demonstrated that four of these MER41 elements are essential for the expression of innate immunity proteins. In addition, luciferase reporter construct assays showed that MER41 and the LTRs of similar ERV elements from dog and cow drive IFN-inducible transcription in HeLa cells. Mice lack the MER41 ERV family, but the muroid-specific endogenous γ-retrovirus RLTR30B also drives IFN-inducible transcription in the same assay system. Moreover, bioinformatic analysis of the mouse genome reveals a significant association of RLTR30B elements and functionally annotated immunity loci. So, it is difficult to escape the conclusion that these two distinct ERV families have contributed in a convergent manner to formatting the innate immune response networks in mice and humans.

### 7.5. Mobile DNA Elements Are Major Contributors to “Non-Coding” Regulatory RNA Molecules

It is only in the last couple of decades that molecular biologists have become broadly aware of a major class of genome regulatory molecules that are linked to mobile DNA elements. In 2003, the National Institutes of Health established the ENCODE project to analyse the informational content of rapidly growing human genome data. The name of the ENCODE project stands for Encyclopedia of DNA Elements [512,513,514,515]. The goal of ENCODE is to use modern genome sequence data to understand the manifold ways that DNA encodes biological properties, in the first instance of humans (based on the Human Genome Sequence) but more broadly of all forms of life. The name is well chosen because it avoids making assumptions about the nature of these DNA “elements” (as using a term like “gene” would have done). The neutral term “DNA element” came out of increasing experimental data showing that genomes do far more than replicate and encode proteins. In particular, molecular studies have documented a diverse and ever-growing repertoire of RNA molecules that do not encode proteins, but nonetheless contribute to determining cellular and organismal properties [516,517].

In 2007, ENCODE project researchers examined the expression of 1% of the human genome [518,519]. To their surprise, they found that 80% of the DNA sequences examined templated RNA transcripts, even though only <1.5% of the DNA actually coded for proteins [520]. This result posed a challenge to conventional views of genome content devoted primarily to encoding proteins. In 2012, the ENCODE project published a follow-up series of papers on the functionality of the full human genome [521]. The analysis confirmed the earlier results, showing that at least 76% of the human genome is transcribed into RNA, and expanded the repertoire of distinct RNA types that have been found [522,523]. As the news article reporting the 2012 analysis expressed in its title, “ENCODE project writes eulogy for junk DNA”, because it documented the active expression of most repetitive components of the human genome [443].

#### 7.5.1. MicroRNAs

Among the first of the novel non-coding RNA products to be widely investigated in detail were short molecules (2–3 dozen nucleotides in length) that were grouped under the term “micro-RNAs”. MicroRNAs play key roles in guiding the epigenetic and transcriptional formatting of eukaryotic genomes, thereby affecting virtually every phenotypic character [524,525,526]. MicroRNAs provide target-specific guidance to genome formatting complexes by sequence complementarity with DNA or nascent RNAs. Examples of microRNA-regulated phenotypes include pluripotency [527], hematopoiesis [528], neural development [529,530], and DNA damage response [531] in mammals, and heat stress response [532], innate immunity [533], and fruit development [534] in plants. Some microRNAs transfer intercellularly in membrane-bounded vesicles, where they serve as signaling molecules [535,536] and can affect properties, such as immune responsiveness [537].

As we might expect, the microRNA repertoire of each species is taxonomically specific [538,539,540], and thus is a key product of evolutionary variation [541,542,543]. A significant role for miRNAs was recently reported in sympatric evolution of naked mole rats [544]. Mobile and repetitive DNA elements encode or have contributed to the evolution of microRNAs in many different species [545,546,547,548]. In Vesper bats, for example, over 61% of taxon-specific microRNAs can be traced back to mobile elements, largely from bat-specific DNA transposons [549]. Mobile DNA-derived microRNAs arise from lineage-specific transposition events [550,551] that can occur in bursts of genome innovation [552]. From a functional point of view, it should be easy to appreciate that the presence of dispersed mobile DNA sequences in other classes of cellular transcripts, such as mRNAs, provides targets for recognition by complementary microRNAs that are derived from the same mobile element [553].

#### 7.5.2. Long Non-Coding lncRNAs

Another major group of regulatory transcripts are called lncRNAs, for “long non-coding RNAs”, which are arbitrarily defined as molecules >200 nucleotides in length. Various lncRNA molecules have been linked empirically to the control and the expression of an extensive range of organismal phenotypes in eukaryotes, from the speed of metabolic induction in budding yeast to nervous system development in humans (Table 14). It is evident from the functions that are cited in Table 14 that some lncRNAs have rather specific effects on cell physiology or developmental processes (e.g., *RZE1* in *Cryptococcus neoformans*), while other lncRNAs participate in regulatory circuits common to many different cell types and organismal processes (e.g., *HOTAIR* and *ANRIL* in humans).

The connections are strong between lncRNAs, rapid evolutionary change, and mobile repetitive DNA elements in tomatoes [554], as well as in mammals, where we have the most abundant information [555,556,557]. A 2012 analysis reported that 97% of human lncRNAs are primate-specific and found mobile DNA element sequences in 83% of 9241 human lncRNAs, where they constituted 42% of total lncRNA sequence [558]. Multiple reports have noted a strong correlation between lncRNA expression in stem cells and the presence in the corresponding genomic DNA of transcriptional regulatory signals from the HERVH family of human endogenous retroviruses [558,559]. HERVH sequences are present in the promoters of more than 100 lncRNAs, evidently providing transcriptional regulatory signals for pluripotent stem cell-specific expression [485,558,560]. A parallel stem cell-ERV association was found in mouse lncRNAs [561].

Because they are repetitive sequences, the mobile DNA-derived segments in lncRNAs are able to serve as recognition modules for regulatory interactions with other cellular RNAs or with genomic DNA regions that have complementary repeats. A few cases of this kind of recognition have been indicated in Table 14. *Alu* SINE-based lncRNA recognition to increase the synthesis of proteins from *Alu*-containing mRNAs has been called the “SINEUP” phenotype [562]. The role of mobile DNA sequences as functional domains of lncRNA molecules has similarly been assigned the acronym of RIDL, for Repeat Insertion Domains of lncRNA [563]. In human fibroblasts, *Alu* repeats in lncRNAs allow for them to serve as “sponges” to lower the content of *Alu*-derived micro-RNAs [564]. Some repetitive sequence interactions can be quite precise. It has been reported that a single point mutation in an *Alu* SINE retrotransposon sequence in a human lncRNA is pathogenic and leads to brainstem atrophy and death [564].

In addition to the relatively few species where functional studies have taken place (e.g., Table 14), reports of lncRNA repertoires have been published for diverse species, such as the fungus *Neurospora crassa* [565], the parasite *Trichomonas vaginalis* [566], the mosquito malaria vector *Anopheles gambiae* [567], the silkworm *Bombyx mori* [568], various plants [569,570], the early metazoan demosponge *Amphimedon queenslandica* [571], and Rainbow trout [572]. The abundance of lncRNA molecules in these various organisms has been interpreted as being indicative of the regulatory complexities involved in their reproduction and development. There are several articles reporting attempts to construct taxonomies of the different types of lncRNAs [573,574,575,576], but many years of transcriptomic and experimental studies almost certainly lay ahead before we have anything like a comprehensive classification and functional understanding of these versatile molecules.

## 8. Ecological Disruption and Read-Write Genome Modifications

As Eldgredge and Gould noted over four decades ago, the paleontological record is characterized by episodes of relative evolutionary stasis that is punctuated by episodes of intense evolutionary variability and innovation [577] (Table 1). This pattern of “punctuated equilibrium” indicates that there is a connection between ecological stability or instability and evolutionary change. Now that we know about so many biological processes of genome rewriting, it is important to consider how ecological disruption relates to their activity. Although it has long been taboo in some evolutionary circles to assert that environmental influences can impact genome variability, we will see that there exists the beginning of a significant literature documenting precisely those impacts (http://shapiro.bsd.uchicago.edu/ExtraRefs.CanGenomeChangeBeLinkedEcologicalDisruption.shtml).

### 8.1. Ecological Change, Mating Population Decline and Interspecific Hybridization

Section 4 detailed the impact that interspecific hybridization has on speciation, WGDs, and the activation of mobile DNA elements. For hybrid speciation to occur, individuals of each parental species have to mate with individuals of the other species rather than their normal conspecific mating partners. It is easy to see that an adverse ecology and a consequent decline in the size of the mating population for one or both species will make cross-species mate choices more likely. This is precisely the pattern that has been observed in small island populations, where hybrid speciation is unexpectedly common [468,578,579]. Continued observation of Darwin’s finches *Geospiza* in the Galapagos Islands [580] has made it possible to link episodes of abrupt climate change to increased evolutionary variability [21,23].

Much of the variability in hybrid speciation comes from the well-documented activation of mobile DNA elements [468,475] (Table 5). Such hybrid activation can amplify the abundance of mobile DNA elements in the genome, as observed in *Drosophila* [581], and these newly dispersed elements in turn can lead to the formation of novel regulatory networks with the potential to generate adaptive phenotypic novelties (Section 7). The predicted accumulation of mobile DNA elements with adaptive radiation has occurred in Hox clusters of *Anolis* lizard genomes [582]. Similar roles for hybridization and mobile DNA activation have been invoked to provide “a more complete and satisfactory explanation for Darwin’s ‘abominable mystery’: the spectacular success of the angiosperms” [583,584]. There is a report of exactly this hybridization-driven series of mobile DNA changes in the recently formed invasive cordgrass species *Spartina anglic* [585], and they have been invoked to account for the more general “genetic paradox” of invasive species [586]. A parallel role for interspecific hybridization, mobile DNA, and network rewiring (cf., Section 7.4) has also been suggested in primate evolution [587,588].

### 8.2. Regulated Biochemistry at the Basis of Point Mutations, Deletions, Translocations and Mutational “Storms”

It is useful to keep in mind that the first demonstrations of induced mutagenesis in bacteria documented quantitatively significant effects of environmental conditions, such as UV radiation, chemicals, and temperature [589]. Today we understand in exquisite detail the role of the SOS DNA damage-sensing control circuit and of SOS-regulated mutagenic trans-lesion DNA polymerases in producing the mutational responses to such environmental factors [590,591,592]. Y-family mutagenic DNA polymerases are present in both eukaryotes, as well as prokaryotes, where they are essential to both “spontaneous” and induced mutations [593] (http://shapiro.bsd.uchicago.edu/Translesion_mutator_polymerases.html). These mutagenic Y-family polymerases have also been implicated in chromosome breakages and complex genome rearrangements [594]. In other words, localized genetic change in response to DNA-damaging agents is fully as much an active biological process as the transposition or retrotransposition of mobile DNA elements.

In recent years, we have come to identify a variety of biochemical and cellular processes that mediate localized point mutations, deletions, and translocations, plus two newly recognized forms of clustered mutational changes frequently detected in cancer cell genomes: “kataegis” and “chromothripsis” (Table 15). Kataegis comes from the Greek word for thunder and designates a “shower” of point mutations that are spread over a contiguous region of the genome that can range from a few dozen bp to many kb in length [595]. Molecular characterization of the mutations in these kataegic showers indicates that they result from the action of the AID/APOBEC family of cytosine deaminases on exposed single-stranded domains [596]: mutations are predominantly (>70%) C-to-T transitions, as expected for C-to-U deamination products [597,598], and are largely fixed in the same DNA strand, a sign of catalytic processivity [599]. Chromothripsis means “chromosome shattering”, and indicates cases where multiple chromosome fragments, usually from a single chromosome, are ligated together in a new arrangement with multiple transpositions, inversions, deletions, and duplications [600,601,602]. A variety of processes are thought to lead to the underlying multiple chromosome breakage and DS break repair events that are producing each chromothripsis occurence (see Supplementary Table S15 for references), but we know that chromothripsis usually occurs with a single chromosome because breakage and repair processes can be seen to occur on individual chromosomes that are isolated in micronuclei compartments [603].

While being observed initially in studies of cancer cell genomes (Section 9.2), both kataegis and chromothripsis also occur in the tissue of healthy individuals [604,605]. Somatic hypermutation for antibody maturation takes place in activated B cells as a tightly regulated and targeted form of kataegis that are catalyzed by the AID cytosine deaminase [606]. A form of kataegis occurs in the yeast genome at highly transcribed loci [607,608] and also at tRNA loci [609]. Chromothripsis affects multiple protein-coding loci in healthy individuals [604], and has been found to arise more frequently than previously thought in both gametogenesis and early human embryogenesis [601,610,611,612]. Obviously, germline episodes of either kataegis, chromothripsis, or both can contribute to major genome changes in evolutionary diversification and innovation [613,614]. The most important point from a conceptual perspective is that we now have experimentally established and mechanistically realistic processes for triggering multiple mutations “all at once” in a single cell division cycle. Intriguingly, chromothripsis can also result from L1 LINE-mediated retrotransposition and *Alu*-*Alu* non-allelic recombination [615]. Thus, ecological or cellular factors that activate L1 LINE elements are also able to trigger chromothripsis. These factors include gamma irradiation [616], benzyprene [617], oxidative stress [618], and heavy metals [619,620,621].

### 8.3. Diverse Ecological Impacts on Natural Genetic Engineering Functions

We have known for many decades that ecological disruption and stressors have direct effects on natural genetic engineering functions, as well as indirect effects by means of interspecific hybridization. The SOS response to DNA damage serves as a paradigm of a cellular sensing and regulatory system controlling mutagenic activities [591,622]. All organisms have DNA damage response (DDR) systems to correct errors that occur during genome replication or from a wide range of cellular insults that lead to DNA breakage, oxidation, or other chemical modifications [8,623]. An enormous literature documents how these systems are regulated and can operate in both error-free and error-prone modes (e.g., http://shapiro.bsd.uchicago.edu/ExtraRefs.DNADamageRepairAndMutagenesis.shtml), either to restore the original genome sequence or to generate novel genome sequences—and even novel genome configurations, as we have seen in the case of chromothripsis (Table 15).

Having well-studied models makes it easy to understand that many mutagenic DNA repair processes and other NGE systems are stress-induced or are otherwise sensitive to ecological changes (Table 16). The ecological factors that influence genome change fall into three broad categories:

1. organismal growth conditions (starvation, etc.), growth phase and cellular differentiation;
2. interactions with biomolecules, including antibiotics, hormones, nutrients, signals, extracellular products of pathogens (toxins, etc.), as well as biotic stresses, such as bacterial, fungal, and virus infection; and,
3. abiotic stresses, including heat, cold, drought, oxidizing agents, heavy metals, wounding, and even space travel.

Looking at the results that are summarized in Table 16 and those reported online in 2011 (http://shapiro.bsd.uchicago.edu/StimuliDocumentedActivateNGE.html), it is significant to note the many cases where genome change is triggered by biosphere interactions. These include the inter-species effects of biological molecules (e.g., antibiotics on bacteria or bacterial toxins on plants and animals) and direct interactions between different organisms (e.g., the many different bacterial infections that induce DNA damage and mutagenic responses in human cells, viral infections that stimulate mutagenic genome repair or mobile DNA activity in plants and animals). There are two major take-home lessons from these initial data about the ecological effects on active genome rewriting: (1) They reinforce the idea that it does not make sense to think of evolution as a process that only involves the isolated genome of one species at a time, and (2) they make it clear that ecological disruptions both stimulate and influence the nature of evolutionary genome change in complex ways [624,625,626,627,628,629,630,631].

An instructive example of the danger of ignoring ecological challenge as a stimulant for genome change in evolution studies is the case of a citrate-utilizing strain emerging unexpectedly in *E. coli* cultures after years of growth under standard laboratory conditions [632]. The appearance of this strain, which activated the expression of a citrate transporter (CitT) by a non-homologous transcriptional fusion, was originally claimed to be so unusual as to be considered equivalent to a paleontological speciation event [633,634]. However, when *E. coli* was subjected to direct selection for citrate utilization, and experienced the NGE triggering effect of aerobic starvation [635], the same class of CitT activating fusion was obtained repeatedly in each of a series of replicate cultures within a matter of days [632,636,637].

## 9. Further Reflections on Genome Rewriting by NGE as a Core Biological Capability

Certainly, one of the most basic facts of life for all organisms is that their reproduction and evolution take place in a highly dynamic environment. Ecology and biosphere interactions are subject to constant change. The unceasing flux in the conditions of life means that survival requires constant adaptation and change on the part of each organism. In the short term, adaptive variability operates through the action of physiological and behavioral control networks, as well as opportunistic symbiotic associations. Over the longer term, however, adaptation requires more fundamental changes in organismal structure and genome composition. That is what we mean by the term “evolution”. As a consequence of this evolutionary fact of life, the active biological processes outlined in the preceding sections of this review—symbiogenetic cell fusions, intercellular DNA transfers, and natural genetic engineering functions—must be considered as core biological capabilities. In this final section of the review, it is appropriate to consider lessons to be learned from cases where active genome rewriting occurs outside the evolutionary context and to pose one fundamental unanswered question that we need to address in future research.

### 9.1. Natural Genetic Engineering as Part of the Normal Life Cycle

A further appreciation of cell capacities for genome rewriting is apparent in the range of organisms that have evolved dedicated DNA modification capabilities to meet special needs of their normal life cycles. These dedicated NGE activities typically display a high degree of specificity within the genome, and thus exemplify mechanisms that living cells possess for targeting DNA rewriting to sequences with particular functional significance. We may consider these life cycle systems as examples of cellular virtuosity in genome modification:

#### 9.1.1. Diversity-Generating Retroelements (DGRs)

DGRs are targeted DNA mutagenesis operators that are found in bacterial and bacteriophage genomes that promote rapid and repeated diversification of a specific region in a protein coding sequence [638,639]. DGR diversification utilizes a highly mutagenic reverse transcriptase to generate novel DNA segments for the incorporation into a specific region of the target coding sequence (“mutagenic homing”). Mutagenic homing allows for bacteriophages to alter their attachment specificity for host cell receptors and bacteria to diversify their surface proteins.

#### 9.1.2. Bacterial Phase Variation

Bacterial phase variation [640] signifies the ability of bacteria to alternate between different protein expression states [641,642,643]. The action of site-specific recombinases to invert promoter elements relative to the transcribed coding sequence reversibly activates or inhibits protein expression [644,645,646]. Reversible inactivation of expression also occurs by the insertion and excision of DNA transposons (insertion sequences or IS elements) at the target locus [647], as well as thr expansion and contraction of simple sequence repeats (SSRs) that change reading frame [648,649].

#### 9.1.3. Bacterial Antigenic Variation

Bacterial antigenic variation signifies the ability of bacteria to change the structure or identity of surface proteins [641,642,643]. The molecular mechanisms adapted for changing surface proteins include site-specific recombination to invert parts of the coding sequence (“shufflons”) [650] and segmental coding sequence replacement from silent cassettes by targeted homologous recombination [651,652,653].

#### 9.1.4. CRISPR Systems for Adaptive Immunity

Both archaea and eubacteria possess the famous CRISPR systems for adaptive immunity against the entrance of bacteriophage or plasmid DNA. Upon infection, the CRISPR defense involves rapid target sequence acquisition [654,655] (adaptation) into a specialized genomic expression site (the CRISPR array) to enable RNA-directed cleavage of complementary invading DNA (immunity) [656,657,658]. Separate dedicated DNA cleavage-ligation complexes are involved in spacer integration into genomic CRISPR arrays [659], on the one hand, and RNA-targeted attack of invading target DNA, on the other [660,661]. The adaptation activities appear to have evolved from a family of mobile DNA elements called “casposons” [662,663].

#### 9.1.5. Prokaryotic DNA-Targeted Adaptive Immune Defense

Some archaea and bacteria possess a distinct adaptive immune defense against incoming plasmid DNA that is targeted by acquired DNA segments that are bound to a prokaryotic Argonaute (Ago) protein [664,665,666]. Little is known yet about how the targeting DNA is acquired. It should be noted that the prokaryotic Ago is the ancestor of eukaryotic Argonaute family proteins that participate in piRNA-targeted defense against invading mobile DNA elements, as well as siRNA regulation of genome expression [667].

#### 9.1.6. Prokaryotic Systems for Aggregating Coding Sequence Cassettes

As previously described in Section 5.2, both archaea and bacteria possess multiple complex genomic elements that utilize site-specific recombinases or transposases for aggregating coding sequence cassettes that are related to a particular adaptive phenotype, such as antibiotic resistance, pathogenicity, or symbiosis in plasmids, transposons, integrons, genomic islands, and integrative and conjugative elements.

#### 9.1.7. Yeast Mating-Type Switching

In certain yeasts, haploid cells execute tightly regulated switches from one cell mating-type (MAT) to the complementary cell type by DNA rearrangements [668]. (i) In the more primitive mating-type switches, a DNA segment carrying the two distinct MAT loci is flanked by inverted repeat (IR) sequences and borders an epigenetically silenced region of the genome. As a consequence of this arrangement, recombination between the IRs inverts the segment and changes which MAT locus is silenced and which expressed [669]. (ii) In the more highly evolved process, the genome contains one expressed MAT locus determining cell type, and two silent cassettes that contain protein sequences that determine each of the different mating types. A targeted process of directional recombination switches mating type by replacing DNA at the functional MAT locus with sequences from the silent cassette of the opposite type. The molecular details of the cassette replacement mechanism differ significantly in budding yeast and fission yeast, and thus appear to have evolved independently [668].

#### 9.1.8. Trypanosome Antigenic Variation

Trypanosomes and certain other eukaryotic pathogens undergo antigenic variation by introducing sequences from silent DNA cassettes into genome expression sites [670,671,672]. Variation typically only affects a segment of the expressed surface protein (“segmental gene conversion”) [673], and variability is enhanced by the presence in the genome of arrays of multiple silent cassette sequences (~1000 or more in some trypanosomes). The process of segmental gene conversion involves homologous recombination at embedded repeats and starts with targeted DNA breaks.

#### 9.1.9. Ciliate Macronucleus Genome Restructuring

The most prolific natural genetic engineers in the normal life cycle are the ciliates, also known as ciliated protozoa [674,675,676,677]. After each mating cycle, these remarkable unicellular organisms completely restructure their typically eukaryotic germ-line genomes into a distinct somatic genome organized as numerous multi-copy mini-chromosomes that are contained within a large transcriptionally active “macronucleus”. The generation of the macronucleus genome involves programmatic RNA-directed cleavage of the germ-line DNA and the elimination of germ-line specific DNA, followed by a distinct process of RNA-directed reassembly of the remaining DNA segments into functional mini-chromosomes that are capped with telomeres at each end. The exact details of germ-line DNA loss and mini-chromosome construction differ between ciliate taxa, but in the well-studied case of *Oxytricha*, at least 90% of germ-line DNA is eliminated, and over 200,000 remaining micronucleus fragments are assembled into ~16,000 intact single coding sequence mini-chromosomes. The fragments are often joined together in a different order from that which is present in germ-line DNA, so the macronucleus arrangement is designated as “scrambled”. During assembly of the ~16,000 scrambled *Oxytricha* mini-chromosomes [678], there are both error correction processes [679] as well as the occasional generation of novel coding sequence combinations [680]. The ability of *Oxytricha* and other ciliates to rapidly generate so many DNA cleavage and rejoining events and properly order the resulting constructs establishes a high standard for cellular control of complex NGE processes.

#### 9.1.10. Mammalian Adaptive Immune System Rearrangements

An outstanding series of highly targeted and integrated NGE processes in response to biological signals takes place in cells of the mammalian adaptive immune system. Mammalian B lymphocytes produce, refine, and diversify a virtually limitless repertoire of highly specific defense molecules to detect and counter invading pathogens by constructing and modifying antibody heavy (H) and light (L) chain coding sequences [681]. The first step is to generate a limitless array of diverse antibody binding specificities. B cells use a domesticated transposase protein in a spatially organized process to cleave stochastically chosen cassettes from genomic arrays, then to add untemplated nucleotides to some of them, and finally to use classical NHEJ repair activity to join the appropriately cleaved cassettes into novel coding sequences, determining the variable (V) region of one H and one L chain per lymphocyte (“V(D)J joining”) [507,682,683]. Paired V_H_ and V_L_ domains form an extremely diverse population of combinatorial antigen binding sites on the B cell surfaces. When the appropriate combination binds an invader antigen, the encoding B cell is “activated” to multiply and undergo a further sequence of specific DNA changes involving the AID activation-induced cytosine deaminase. The DNA sequence encoding the V region (but not the constant or C region) of each chain is subjected to a kataegis-like somatic hypermutation “storm”, which is precisely targeted by transcriptional signals [684,685]. As some of the hypermutated antibodies bind the target antigen with a higher affinity, the activated B cells are further stimulated to multiply and undergo a “class switch recombination” (CSR) process that replaces the exon encoding the C region of the H chain and thereby targets the higher affinity antibody to the appropriate region of the body for each particular infection without altering its binding specificity. C region exon choice is determined by transcription signals determining the sites of AID-dependent DSB and NHEJ nonhomologous recombination events joining V region exons to new C region exons [686,687]. The highly orchestrated NGE processes that are involved in antibody formation and maturation are especially noteworthy because they illustrate how living cells are able to combine great precision in DNA modifications with extraordinary diversification of outcomes for a well-defined adaptive purpose.

### 9.2. Lessons on the Real Time Potential of Natural Genetic Engineering from Cancer Genomes

Cancer provides a model evolutionary system, where we can observe the possible extent of genome changes that are occurring in real time (the months or years it takes for a tumor population to develop). Genome changes are usually linked to tumor progression (the appearance of more aggressive and rapidly proliferating cancer cells) and consequently are of great interest to clinicians, who try to combat the disease. For our purposes, however, we are interested in cancer as a demonstration of how widely and rapidly complex genome changes have been documented to occur. It is broadly accepted that cancer cells are destabilized in maintenance of genome stability [688], and the exceptional tumor state can serve as a paradigm for genome destabilization that is triggered by ecological disruption in periods of evolutionary change [689]. It is no coincidence that many of the ecological triggers for NGE activity in Table 16 are also carcinogenic (e.g., genome-destabilizing microbial infections) [690,691,692].

Cancer cells reproducibly display many forms of natural genetic engineering discussed earlier in this review (Table 17). This is solid evidence for the repeated occurrence of rapid genome change by NGE in real time under the regulatory context of tumor development. The actions of RAG transposase and AID cytosine deaminase in immune system tumors demonstrate the adaptive utilization of available NGE resources in tumor evolution [693,694,695].

### 9.3. What Factors May Bias Genome Rewriting to Generate Selectively Positive Outcomes?

The preceding discussion lays out only a small selection of the massive experimental and genomic data that show that genome change in evolution is invariably a product of cellular and biochemical action. From single nucleotide changes involving error-prone repair functions (Table 15), through to the formation of distributed regulatory networks by mobile DNA formatting (Section 7.4), to whole genome restructuring that is triggered by interspecific hybridization events, non-random biological action is fundamental to genome innovation. Since cellular and biochemical activities are regulated and sensitive to multiple biotic and abiotic ecological inputs (Table 16), the recognition of biological agency in genome rewriting enables us to pose a question that has long been considered taboo in orthodox discussions of evolution: What factors may bias genome rewriting to generate selectively positive outcomes? We know unambiguously that such bias is at work in the somatic genome targeting processes described in Section 9.1. Why not in the course of evolution as well?

A useful way to address the global question of bias towards selective utility in NGE evolution processes is to separate out a series of easier-to-answer subordinate questions. If we can answer those affirmatively, then there is a solid basis for pursuing a scientific approach to adaptive guidance in evolutionary NGE genome variability.

#### 9.3.1. Do Living Organisms Possess NGE Operators of Clear Evolutionary Utility?

The answer to this question is unambiguously yes. The NGE operators range from horizontal DNA transfer systems (Table 6 and Table 7) to the DNA-based and RNA-based mutational operators that play key roles in protein evolution (Table 9 and Table 10) to mobile DNA elements that rewire transcriptional regulatory networks (Table 13 and Section 7.4). Additional NGE processes that contribute to taxonomic and adaptive innovation in evolution include large-scale genome rearrangements, as documented in Table 5, Table 15 and Table 17.

#### 9.3.2. Can the Evolutionarily Useful NGE Operators Be Regulated and/or Targeted in Ways that Could Enhance Their Adaptive Utility?

Here again, the answer is clearly yes. We see both regulation and targeting at work in adaptively useful ways in the various somatic NGE systems described in the preceding Section 9.1. Diverse ecological triggers for NGE activity are presented in Section 8 and Table 16, and posted online (http://shapiro.bsd.uchicago.edu/StimuliDocumentedActivateNGE.html). There is also extensive documentation of targeting mechanisms at work directing mobile DNA element insertions to specific genome sites or regions in organisms that range from bacteria to plants and animals [696,697,698,699], (http://shapiro.bsd.uchicago.edu/ExtraRefs.TargetingNaturalGeneticEngineeringInGenome.shtml; http://shapiro.bsd.uchicago.edu/Targeting_retroviral_and_retrotransposon_insertions.html; http://shapiro.bsd.uchicago.edu/P_factor_homing.html). Targeting and activation are particularly relevant to the larger question of positive bias in evolution when they operate via a global genome control process like epigenetic genome modification as, for example, in mouse embryonic stem cells [696].

#### 9.3.3. Are There Generic Features of Cellular Genome Function That Can Favor Evolutionarily Adaptive NGE Outcomes?

Once again, the answer is positive, but in this case, we need to make a distinction between genome features that are already described as having contributed to evolutionary success and other features whose potential contributions to evolutionary innovation remain to be explored further.

The aspects of NGE activity on cellular genomes that facilitate the positive adaptations detailed above include active nucleotide sequence modification (Table 15), the capacity for both homologous and non-homologous DNA exchanges, interactions with viral genomes, the presence of mobile DNA elements, and the formation of genome duplications [325,700,701,702,703,704,705,706,707]. In prokaryotes, the most numerous organisms in the biosphere, mobile DNA, includes transmissible plasmids and other conjugative elements. But, intercellular DNA transfers are by no means restricted to prokaryotes because all organisms can exchange nucleic acids that are encapsidated in viral particles, and in some cases, take up DNA directly from the environment, in lipid vesicles (exosomes), or by direct contact with other cells, such as endosymbionts (Table 6). In eukaryotes (but not exclusively), mobile DNA includes reverse-transcribed cDNA retrocopies of processed cell RNAs, which are important contributors to protein evolution (Table 9). The non-random nature of certain NGE operations, in particular the movement of defined DNA transposon and retrotransposon elements, favor their adaptive utility in mobilizing transcription and other signals to new genome locations. As stated in the title of this section, producing genome change to generate adaptive novelties is clearly “a core biological capacity”.

In addition to those features of genome structure and function that enable adaptive DNA rewriting, there are higher-order systemic features that may work informatically to bias genome change towards adaptively useful outcomes. A number of authors from the Evo-Devo school have pointed to modularity and repetition in control networks as features that favor successful innovation in developmental and morphogenetic processes [708,709,710]. Some have even cited Richard Goldschmidt, considered by some as the “father” of Evo-Devo thinking (Table 1), and pointed out that organisms bearing morphogenetic innovations may be thought of as Goldschmidt’s “hopeful monsters” [170,172,173].

A further generic feature of genome structure and action that could help bias NGE towards useful outcomes is that genome expression adapted to environmental conditions is critically dependent upon the physical and chemical status of cellular DNA. Chemical modifications like nucleotide methylation, DNA binding to specific packaging, replication, transcription and regulatory proteins, organization of the genome into functionally distinct epigenetic domains, and three-dimensional alignment of sometimes distant DNA sites by interactions with protein and lncRNA networks are all information-rich features of genome structure, which have the potential to target and coordinate NGE activities [711,712,713,714].

#### 9.3.4. Are There Feasible Experimental Approaches to Demonstrating and Dissecting Selectively Advantageous Bias in Complex Evolutionary NGE Events?

This is a critical question for evolution research in the coming decades. There have been numerous studies of how organisms acquire one or a few individual traits, such as the *E. coli* citrate utilization case described above (Section 8.3, [636,637], but (as far as I am aware) it still remains to be demonstrated that a truly complex and adaptive “hopeful monster” innovation can be generated de novo in real time so that successful cases can be subjected to genomic analysis to determine the underlying NGE operations. The approach is similar to that employed in real time adaptive evolution of microbial strains for biotechnology applications [715]. The “hopeful monster” class might include complex innovations like (i) origination of a new multi-step catabolic or biosynthetic pathway in a microorganism, (ii) morphogenesis of a useful appendage or organismal structure on a plant or animal that is not simply a modification of a pre-existing feature, or (iii) elaboration of a functional signaling system that is based on a novel signal molecule, receptor, and receptor-linked signal transduction cascade.

The major challenge to observing complex evolutionary events in real time is the elaboration of a suitable selection scheme. In the case of microbial pathways, this is achievable by utilizing a novel selective substrate (i.e., one for which no catabolic pathway exists) as the source of an essential growth requirement (C, N, etc.), or starting with an organism that is completely lacking the ability to synthesize structures required for a selectable property, such as motility in fluid medium or across a solid surface.

If the selection proves unexpectedly easy, then it is likely that silent precursor coding elements already existed in the genome, and sequence analysis will reveal their reproducible activation in independently selected clones. However, if the selection proves difficult and requires prolonged incubation (perhaps several weeks or months for bacterial systems, cf., [716]), then it may well be that a complex NGE process was at work. Genome sequence analysis will reveal how many distinct steps were required, as well as how reproducible those steps proved to be in independently selected isolates. Repeatedly finding certain DNA rearrangements in the adaptation process (e.g., the movement of mobile DNA elements or retroposition of protein-coding sequences) would be evidence for genome features that bias changes towards adaptive success. The confirmation of such a conclusion is possible by removing or altering the putative biasing feature and measuring the resulting impact on successful responses to selection. Fortunately, contemporary CRISPR-based technologies make all manner of genome alterations feasible on virtually any scale in all organisms [717]. In some cases, horizontal DNA transfer may be required for a complex selection to succeed. If so, then the starting culture would have to contain two or more species, and that requirement itself would be a significant result, which is consistent with genomic observations on adaptive sequence acquisitions across taxonomic boundaries (Section 5).

It is beyond the competence of a bacterial geneticist to speculate on selective regimes for truly complex adaptations in multicellular organisms. Nonetheless, two aspects of successful “hopeful monster” hunts in plants and animals can be predicted based on the biological genome rewriting activities that are discussed above. (1) The first is that success is most likely to depend upon the stimulating effect of interspecific hybridization for providing greater starting sequence variability and generating increased NGE activity prior to selection (Table 5). This effect of interspecific hybridization has been observed in *Saccharomyces* yeasts, where interspecific hybrids reproducibly evolve a chromosome translocation that confers adaption to ammonia limitation [718]. (2) The second is that plants and animals all exist as holobionts, and it is probable that complex evolutionary success will involve microbial NGE triggering (Table 16) or horizontal DNA transfer (Section 5). Thus, it may turn out that success in plant or animal “hopeful monster” hunts may be dependent upon the presence of certain symbiotic or even pathogenic microbes in the experimental material. Clearly, the discovery that experimental plant or animal evolution is sensitive to microbial stimulation would be a highly significant result, demonstrating the holobiont principle [18,719]. Tests for microbial stimulation could include viruses, which have been found to be significant contributors to the evolution of species, like our own [720].

### 9.4. Conclusions

Hopefully, the preceding discussion will make it clear that there are well-defined empirical approaches to addressing key questions about what active biological processes are operational in real-time evolution of complex adaptive traits. The fact that evolution experiments may well involve interspecific hybridization, mixed cultures, and microbial infections of test organisms only serves to highlight how far contemporary thinking about hereditary change has advanced from the sterile abstractions of the past two centuries. (The word “sterile” is used here in both usual meanings: (i) devoid of contaminating organisms, and (ii) unable to lead to a productive conclusion.) The experimental and conceptual palette that we have today for probing and depicting the nature of biological variation is far richer than the information that was available in the 20th Century. Integrating powerful genome analysis and modification technologies with imaginative experimental design is certain to provide us with a surprising picture of the many ways living organisms change themselves in the course of evolution.

## Figures and Tables

**Table 1:** Key Scientists Advocating non-Gradualist Evolution in the 19th and 20th Centuries.

Evolutionist	Non-Gradual Evolutionary Process
William Bateson (1861–1926)	Discontinuous variation
Hugo de Vries (1848–1935)	Abrupt mutational variation
Konstantin Mereschkowsky (1855–1921)	Evolution by symbiogenesis
Ivan E. Wallin (1883–1969)	Evolution by symbiogenesis (“Symbionticism”)
Boris Mikhailovich Kozo-Polyansky, (1890–1957)	Evolution by symbiogenesis
George Gaylord Simpson (1902–1984)	Quantum evolution
Richard Goldschmidt (1878–1958)	“Hopeful monsters” formed by redirecting developmental programs
George Ledyard Stebbins (1906–2000)	Hybrid Speciation (“Cataclysmic Evolution”)
Niles Eldredge (b. 1943) and Stephen J. Gould (1941–2002)	Punctuated equilibrium
Lynn Margulis (1938–2011)	Evolution by symbiogenesis

A fully referenced version of this table is available online as Supplementary Table S1.

**Table 2:** Selected Variation Processes that Lead Repeatedly and Abruptly to Adaptive and Taxonomic Novelties.

Symbiosis, Symbiogenesis and “Holobiont” Modifications by Cell Mergers (Section 3)
Interspecific hybridization (hybrid speciation) (Section 4)
Horizontal DNA transfers (Section 5)
Protein evolution by domain rearrangements and coding region innovation (Section 6)
Mobile DNA activity to restructure genomes, rewire developmental regulatory networks and form regulatory long non-coding RNA molecules (Section 7)

**Table 3:** Photosynthetic eukaryotic lineages resulting from symbiogenesis.

Taxonomic Group	Symbiogenetic Origin
*Archaeaplastida*	
Green algae (*Chlorophyta*)	Primary cyanobacterial endosymbiosis
*Glaucophytes* (order *Chlorococcales*)	Primary cyanobacterial endosymbiosis
Red algae (*Rhodophyta*)	Primary cyanobacterial endosymbiosis
Land plants (*Embryophyta*)	Primary cyanobacterial endosymbiosis

Euglyphid amoeba *Paulinella chromatophora*	Primary cyanobacterial endosymbiosis (*Synechococcus-Prochloron* clade)

*Euglenids* (flagellated algae)	Secondary green algal endosymbiosis
*Chlorarachniophytes* (marine algae)	Secondary green algal endosymbiosis
*Chromalveolates* (multiple lineages including organisms responsible for a large fraction of atmospheric oxygen, such as brown algae, coccolithotrphs, cryptophytes and diatoms)	Secondary red algal endosymbiosis
*Dinoflagellates* (flagellated marine and fresh water protists)	Tertiary *chromalveolate* endosymbiosis or serial green or red alga endosymbioses
*Warnowiid* dinoflagellates with camera eye-like “ocelloids”	Ocelloid “cornea” formed by mitochondria and “retina” formed by red algae-derived plastids

A referenced version of this table is available online as Supplementary Table S3.

**Table 4:** Examples of Speciation and Adaptive Radiation Involving Interspecific Hybridization and Changes in Chromosome Number.

**Fungi:** *Saccharomyces* Yeast
**Ciliated Protozoa**
**Plants:** Tragopogon (*Asteraceae*), *Pinus densata*, *Primula*, Sunflowers (*Helianthus anomalus*), Irises (*Iris fulva*, *I. hexagona*, and *I. nelsonii*), *Nicotiana* (*Solanaceae*), *Orchidaceae*, *Brassica napus*, *Arabidopsis*, Potatoes (*Solanum stoloniferum* and *S. hjertingii*), Wheats (*Aegilops*-*Triticum* group), Cotton (*Gossypium*)
**Animals:** Tephritid fruitflies, Mosquitoes, Tiger Swallowtail Butterflies, *Heliconius* butterflies, Ants, Sculpins (*Cottus* sp., *Teleostei*), Sailfin silversides (*Teleostei*), East African Cichlids, Sparrows, Yellow-rumped (Audubon’s) warbler, Galapagos finches (*Geospiza*), Clymene dolphin (*Delphininae* (*Cetacea*, *Mammalia*)), Bats, Chinese hares (genus *Lepus*), Cats (*Felidae*), Colobine monkeys, Southern African baboons

A fully referenced copy of this table is available as Supplementary Table S4.

**Table 5:** Genomic consequences of experimental interspecific hybridization in plants and animals [167].

Genome Effect
Changes in ploidy (mostly WGD)
Alteration of epigenetic modifications to the genome
Alterations in expression patterns across the genome
Activation and spread of mobile DNA elements
Genome restructuring involving mobile DNA elements
Changes in chromosome structure and karyotype
Alteration of tandem repetitive DNA arrays and centromeres

A fully referenced version of this table is available as Supplementary Table S5. Additional references are available on the internet at http://shapiro.bsd.uchicago.edu/Hybrid_dysgenesis_interspecific_hybridization.html and http://shapiro.bsd.uchicago.edu/GenomicResponsesToChangesInPloidyInterspecificHybridization.html.

**Table 6:** Modes of Horizontal DNA Transfer.

DNA Transfer Mode	Donor-Recipient Taxa
Liberation and uptake of extracellular DNA (“Transformation”)	Archaea-archaea; Bacteria-bacteria; Yeast-yeast; *Stramenopile* red alga-alga; Plant-bacteria
Encapsidation in and delivery by a virus or virus-like particle (“Transduction”)	Archaea-archaea; Bacteria-bacteria; Insect-insect; Bacteria-mammal; Mammal-mammal
Establishment of a DNA transport pore between two cell envelopes (“Conjugation”)	Archaea-archaea; Bacteria-bacteria; Bacteria-yeast; Bacteria-fungi and mushrooms; Bacteria-plant; Bacteria-mammalian cells; Bacteria-diverse eukaryotes
Cell fusion	Archaea-archaea
Phagotrophism	Bacteria-protist
Additional DNA transfer modes within taxa:	
Protoplast fusion	Bacteria, Yeast, Fungi, Plants
Liposomal or membrane vesicle-mediated transfer	Archaea, Mammalian cells
Sperm-mediated DNA transfer	Insect larva, Mammals
Parasite- and endosymbiont-mediated transfer (inferred)	Red algae; Aphids, parasitoid wasps; a beetle; *Drosophila*; Butterflies; Vertebrates; Reptile-mammal; Mammals

A fully referenced version of this table is available as Supplementary Table S6.

**Table 7:** Selected examples of inter-phylum adaptive horizontal DNA transfers based on genomic data.

Donor	Recipient	Function(s)
**Prokaryote-Prokaryote**		
Bacteria	Methanogenic Archaea	Aerobic metabolic activities
Bacteria	Thermophilic Archaea	Mesophilic metabolic activities
**Prokaryote-Eukaryote microbe**		
Bacteria	Plant pathogenic fungi	Extracellular proteins, interference with plant defense-response, degradation of plant cell walls, carbohydrate metabolism
Bacteria and Archaea	Red alga *Galdieria sulphuraria*	Growth in high temperature, toxic metal-rich, acidic environments
Proteobacteria, cyanobacteria and archaea	Diatom *Phaeodactylum tricornutum*	7.5% of genetic loci (784 loci); metabolic and biosynthetic activities
Bacteria, archaea	Rumen ciliates	Catabolism of complex carbohydrates
β, γ-*Proteobacteria*, *Chlamydiae*, other bacteria	Red alga *Cyanidioschyzon*	Biosynthetic activities
Rumen bacteria *Fibrobacter succinogenes*	Rumen fungus *Orpinomyces joyonii*	Endoglucanase (polysaccharide digestion)
**Eukaryote microbe-Eukaryote microbe**		
Fungi	Plant parasitic *oomycetes*	Plant cell wall digestion, resisting plant defenses
Algae, bacteria	*Choanoflagellate* (unicellular animal precursor)	405 genetic loci (4.4% nuclear genome); metabolic activities
**Prokaryote-metazoa**		
Bacteria	Sponge	Metabolic and biosynthetic activities
Bacteria	Sponge	Biomineralization
Bacteria	Starlet sea anemone	Biosynthetic activities
Bacteria	Hydra	Metabolic and biosynthetic activities
Bacteria	Plant parasitic nematodes	Cellulose and phytopolymer digestion
Bacteria	Stick and Leaf Insects	Pectinases
Bacteria	Phytophagous mites and *Lepidoptera*	Detoxification of plant defense molecules
Bacteria	Coffee berry borer beetle	Digestion of coffee storage polymer
Bacteria	Silkworm *Bombyx mori* and related *Lepidoptera*	Glycosyl hydrolase family, oxidoreductase family, and amino acid metabolism
Bacteria	Arthropods, echinoderms, and vertebrates, including platypus and opossum, but not in placental mammals	Glyoxylate cycle enzymes
Bacteria	Urochordate *Ciona intestinalis*	Cellulose synthase
**Eukaryote microbe-metazoa**		
Fungi	Plant parasitic nematodes	Cellulose and phytopolymer digestion
Fungi	Pea aphid, spider mite	Carotenoid pigment biosynthesis, cyanate metabolism
Algae	Tunicate *Ciona intestinalis*	Molecule transport, cellular regulation and methylation signaling
**Microbe-plants**		
Bacteria, Archaea, viruses, and sea anemones	Moss *Physcomitrella patens* (primitive land plant)	Xylem formation, plant defense, nitrogen recycling, plus biosynthesis of starch, polyamines, hormones and glutathione; water stress adaptation
Bacteria, fungi	Plants	Biosynthetic activities
Fungi	Plants (Bryophyte moss)	Metabolic activities
**Plants-microbes**		
Plants	Bacteria, fungi, *amoebozoa*	Expansins (plant cell-wall loosening proteins)
Plants	Fungi	Regulatory proteins
**Plant-plant**		
Parasitic plant (*Cuscuta* sp.)	Host plant (*Plantago* sp.)	Mitochondrial loci *atp1*, *atp6* and *matR*
*Rafflesiae* host plant	Parasitic flowering plant *Rafflesia cantleyi*	Metabolic and mitochondrial activities
Grasses	Barley (*Hordeum*)	Ribosomal RNA (rDNA) sequences
Bryophyte hornworts	Fern	Novel chimeric photoreceptor—neochrome
**Animal-animal**		
Fish (herring or sea raven)	Rainbow smelt, *Osmerus mordax*	Type II anti-freeze protein
**Eukaryote-prokaryote**		
Eukaryotic cells (Impossible to identify donor because *Legionella* infects and grows in amoebae, protozoa, *Paramecium*, macrophages, and many other eukaryotic cells)	Bacteria *Legionella pneumophila*	Eukaryote-like regulatory “effector” proteins injected in the course of infecting eukaryotic cells
**Virus-host cell**		
Halovirus	*Halobacterium salinarum* (Archaea)	B-type DNA polymerase B1
T3/T7 family bacteriophage (bacterial virus)	Ancestral eukaryote nuclear and mitochondrial genomes	Mitochondrial replication and transcription activities
Double-stranded RNA viruses (*totiviruses* and *partitiviruses*)	Eukaryote nuclear genomes (plants, arthropods, fungi, nematodes, and protozoa)	Capsid protein and RNA-dependent RNA polymerases
Circular single-stranded DNA viruses (*geminiviruses*, *nanoviruses* and *circoviruses*)	Eukaryotic nuclear genomes (plants, fungi, animals and protists)	Replication initiation protein (Rep)-related sequences
**Host cell-virus**		
Bacteria	Temperate bacteriophages (capable of insertion into bacterial genome in repressed “prophage” state)	Host infectivity and virulence activities expressed in prophage state
Bacteria (primarily, endosymbionts and parasites), bacteriophages, protists, animals	Nucleocytoplasmic large DNA viruses (NCLDVs), including *Poxviruses* and *Iridoviruses* infecting insects and vertebrates, *Mimiviruses* and *Phycodnaviruses* infecting amoebae, protists and algae	Diverse functions including rDNAs, nucleic acid metabolism, signaling and regulatory activities, defenses against apoptosis, immune response, growth factors, and resistance of cells to oxidative stress
Mammal	Influenza virus	28S rDNA insert in hemagglutinin sequence increases pathogenicity
γ-proteobacteria and insects (viral hosts)	Baculovirus	DNA ligase, ribonucleotide reductase 1, SNF2 global transactivator, inhibitor of apoptosis, chitinase, and UDP-glucosyltransferase
Marine phytoplankton *prasinophytes*	Prasinovirus	Glycosyltransferases, methyltransferases and amino acid synthesis enzymes

A more complete, detailed and fully referenced version of this table is available as Supplementary Table S7.

**Table 8:** Highly cited protein domain search terms in the PubMed database.

Domain	Function
Kinase domain	Catalytic (phosphorylation)
Transmembrane domain	Subcellular localization
PDZ domain	Protein-protein interaction module
SH3 domain	Proline-rich protein-protein interaction module
DNA binding domain	Molecular recognition
PAS domain	Sequence-specific DNA binding
WW domain	Protein-protein interaction module
PH domain	Phosphatidylinositol binding in membranes
SET domain	Binding methylated DNA
SH2 domain	Binding proteins containing phosphor-tyrosine residues

A fully referenced version of this table is available as Supplementary Table S8.

**Table 9:** Selected Reports of Exon Rearrangements and Retroposition by Mobile DNA element Activity.

Rearrangements	Taxa
Group II intron retrotransposition and exon shuffling	Yeast
Chimeric LINE-mediated retrogene formation	Rice blast fungus *Magnaporthe grisea*
DNA “splicing” by transposase-related excision functions	Ciliated protists
Exon shuffling by DNA transposons (Pack-MULE, Ac/Ds, CACTA, Helitron)	Plants, including beans and maize
LINE-mediated duplications	Dicots
Retrotransposon-mediated exon shuffling	Maize, *Medicago sativa*
Exon shuffling by DNA transposons (Helitron, FB transposon)	Lepidoptera, *Drosophila*
Retrotransduction by LINEs and SINEs	*Drosophila*
Exon shuffling by retrotransposition	Mammals
Retrotransposon exon shuffling	Primates
TRIM5-Cyclophilin A (TRIMCyp) fusion by retrotransposition	Tree shrews and owl monkeys
Exon shuffling by retrotransposition	Humans
L1/LINE mediated retrotransduction	Humans
*Alu* or *SVA* SINE-mediated retrotransduction	Humans
Chimeric “retrogenes”, by LINE-mediated template switches at the RNA level	Humans

A more detailed and fully referenced version of this table is available as Supplementary Table S9.

**Table 10:** Some Indepedently Reported Instances of Orphan Coding Sequences (“New Genes” and New Exons) Discovered in Sequenced Genomes.

Organism(s)	Characterization of Orphan Coding Sequences
Viruses (bacteriophages)	“Almost one-third of all ORFs in 1456 complete virus genomes correspond to ORFans…38.4% of phage ORFs have no homologs in other phages, and 30.1% have no homologs neither in the viral nor in the prokaryotic world…”
Viruses	“*de novo* genes…in which an existing gene has been “overprinted” by a new open reading frame, a process that generates a new protein-coding gene overlapping the ancestral gene”
Prokaryotes (Archaea and Eubacteria)	20,000 orphan sequences: “…only 2.8% of all microbial ORFans have detectable homologs in viruses, while the percentage of non-ORFans with detectable homologs in viruses is 7.9%, a significantly higher figure.”
*Escherichia coli* O157:H7 (EHEC), *Escherichia coli* K12	“72 genes are taxonomically restricted and, therefore, appear to have evolved relatively recently de novo”…“origin of a new gene through overprinting in *Escherichia coli* K12”
*Saccharomyces cerevisiae*	“BSC4 …encoding a 132-amino-acid-long peptide… no homologous ORF in…closely related species…Because the corresponding noncoding sequences in *S. paradoxus*, *S. mikatae*, and *S. bayanus* also transcribe, we propose that a new de novo protein-coding gene may have evolved from a previously expressed noncoding sequence.”
Yeast (*Saccharomyces cerevisiae*) and *Drosophila*	Protein C-termini: the co-option of short segments of noncoding sequence into the C-termini of existing proteins via the loss of a stop codon: “…54 examples of C-terminal extensions in *Saccharomyces* and 28 in *Drosophila*…Four of the *Saccharomyces* C-terminal extensions (to ADH1, ARP8, TPM2, and PIS1)…are predicted to lead to significant modification of a protein domain structure.”
Green multicellular algae *Chlamydomonas* and *Volvox carteri*	PHD domain added to condensin II by exonization of mobile DNA sequences; “141 retrogene candidates in total in both genomes, with their fraction being significantly higher in the multicellular *Volvox*.”
*Plasmodium vivax*	“…recent de novo origin of at least 13 protein-coding genes in the genome of *Plasmodium vivax*… five of the genes identified in our analysis contain introns…likely evolved from previously intergenic regions together with the coding sequences.”
Nematode *Pristionchus pacificus*	“3818–7545 (39–76%) of orphan genes are under negative selection”
*Drosophila melanogaster*	“…a significant excess of retrogenes that originate from the X chromosome and retropose to autosomes; new genes retroposed from autosomes are scarce…most of these X-derived autosomal retrogenes had evolved a testis expression pattern.”
*Drosophila melanogaster*	“142 segregating and 106 fixed testis-expressed de novo genes in a population sample of *Drosophila melanogaster*…appear to derive primarily from ancestral intergenic, unexpressed open reading frames (ORFs), with natural selection playing a significant role in their spread.”
*Drosophila melanogaster*	“…six putatively protein-coding de novo genes…two de novo genes emerged from novel long non-coding RNAs…four other de novo genes evolved a translated open reading frame and transcription…suggesting that nascent open reading frames (proto-ORFs)…can contribute to the emergence of a new de novo gene”
Insects: arthropod genomes, focusing on seven recently sequenced ant genomes…comparison between social *Hymenoptera* (ants and bees) and nonsocial *Diptera* (flies and mosquitoes)…	“…between the two insect orders *Hymenoptera* and *Diptera*, orphan genes are more abundant and emerge more rapidly in *Hymenoptera*, in particular, in leaf-cutter ants. With respect to intragenomic localization, we find that ant orphan genes show little clustering…”
Entelegyne spiders (Araneae, Entelegynae)	“…transcriptomes of six entelegyne spider species from three genera (*Cicurina travisae*, *C. vibora*, *Habronattus signatus*, *H. ustulatus*, *Nesticus bishopi*, and *N. cooperi*)… between ~550 and 1100 unique orphan genes were found in each genus.”
Rodents	“75 murine genes (69 mouse genes and 6 rat genes)…good evidence of de novo origin since the divergence of mouse and rat. Each of these genes is only found in either the mouse or rat lineages, with no candidate orthologs nor evidence for potentially-unannotated orthologs in the other lineage…For 11 of the 75 candidate novel genes we could identify a mouse-specific mutation that led to the creation of the open reading frame (ORF) specifically in mouse…A large number of them (51 out of 69 mouse genes and 3 out of 6 rat genes) also overlap with other genes, either within introns, or on the opposite strand.”
Mouse and human	“…over 5000 new genes were integrated into the ancestral GGI {gene-gene interaction} networks of human and mouse”
Primates	“an unexpected important role of transposable elements in the formation of novel protein-coding genes in the genomes of primates.”
Human and Chimpanzee	“…retrocopies of coding transcripts to generate proteins with novel N-terminal domains. Examples include thymopoietin beta (*TMPO*), eukaryotic translation initiation factor 3 subunit 5 (*EIF3F*), and the 5′-inverted retrocopy of small nuclear ribonucleoprotein polypeptide N (*SNRPN*).
Humans	“…human-specific de novo protein-coding gene, *FLJ33706* (alternative gene symbol *C20orf203*)…originated from noncoding DNA sequences: insertion of repeat elements especially *Alu* contributed to the formation of the first coding exon and six standard splice junctions on the branch leading to humans and chimpanzees, and two subsequent substitutions in the human lineage escaped two stop codons and created an open reading frame of 194 amino acids.”
Humans	“…60 new protein-coding genes that originated de novo on the human lineage since divergence from the chimpanzee…highest expression levels in the cerebral cortex and testes…”
Humans	“24 hominoid-specific de novo protein-coding genes with precise origination timing in vertebrate phylogeny… most of the hominoid-specific de novo protein-coding genes encoded polyadenylated non-coding RNAs in rhesus macaque or chimpanzee with a similar transcript structure and correlated tissue expression profile…”
Humans	“…*de novo* origin of at least three human protein-coding genes since the divergence with chimp…chimp, gorilla, gibbon, and macaque share the same disabling sequence difference, supporting the inference that the ancestral sequence was noncoding.”
Humans	“… 426 different annotated young domains, totaling 995 domain occurrences, which represent about 12.3% of all human domains. We have observed that 61.3% of them arose in newly formed genes, while the remaining 38.7% are found combined with older domains…Young domains are preferentially located at the N-terminus of the protein…”
*Arabidopsis*	“…lineage-specific genes within the nuclear (1761 genes) and mitochondrial (28 genes) genomes are identified…Almost a quarter of lineage-specific genes originate from non-lineage-specific paralogs, while the origins of ~10% of lineage-specific genes are partly derived from DNA exapted from transposable elements (twice the proportion observed for non-lineage-specific genes). Lineage-specific genes are also enriched in genes that have overlapping CDS, which is consistent with such novel genes arising from overprinting. Over half…of the 958 lineage-specific genes (LSGs)…in *Arabidopsis thaliana* have alignments to intergenic regions in *Arabidopsis lyrata*, consistent with either de novo origination or differential gene loss and retention…LSGs are enriched for genes responsive to a wide range of abiotic stresses…”
*Arabidopsis*	:“… new genes…show a bias in expression to mature pollen… Transposable elements are significantly enriched in the new genes…high activity of transposable elements in the vegetative nucleus, compared with the germ cells, suggests that new genes…generated in the vegetative nucleus in the mature pollen. We propose an “out of pollen” hypothesis for the origin of new genes in flowering plants.

A more complete and fully referenced version of this table is available as Supplementary Table S10.

**Table 11:** Origination of Novel Exons (“Exonization”) from Mobile DNA Elements.

Taxa	Mobile DNA Exonized
Green algae	Transposable elements (TEs)
Plants (coffee, rice, *Arabidopsis*, etc.)	*Ds* transposons and other transposable elements
Ancestral vertebrate	“…a more than 200-base-pair ultraconserved region, 100% identical in mammals, and 80% identical to the coelacanth SINE, contains a 31-amino-acid-residue alternatively spliced exon of the messenger RNA processing gene *PCBP2*…”
Mammal	“Although…not evolutionarily related, mammalian *TMPO* and *ZNF451*…both code for splice isoforms that contain LAP2alpha domains…related to the first ORF from a *DIRS1*-like retrotransposon…domestication happened separately and resulted in proteins that combine retrotransposon and host protein domains. The alternative splicing of the retrotransposed sequence allowed the production of both the new and the untouched original isoforms…”
Mammal, Primate	*MIR* retrotransposons
Mouse	*L1* retrotransposons; “antisense insertions results in an increased potential for exonization”
Rat	*L1* retrotransposon and ERV (endogenous retrovirus) in embryonically expressed *Rtdpoz-T1* and -*T2* locus
Human and mouse	“…exonization of transposed elements is biased towards the beginning of the coding sequence in both human and mouse genes…cases of primate-specific *Alu* elements that depend on RNA editing for their exonization…”
Primate	*Alu* exonization in *BCS1L*, RNA edited *Alu* element in human nuclear prelamin A recognition factor gene transcript
Primate	LINE retrotransposon in *ZRANB2* locus
Human	Anti-sense *Alu* SINEs; *Alu*-derived segments in two Bcl-family proteins.
Human	“Exons derived from *Alu* SINEs but also the exons from the TEs of other families were preferentially established in zinc finger (ZNF) genes.”
Human	“Long Terminal Repeat (LTR) retrotransposons are associated with 1057 human genes (5.8%). In 256 cases LTR retrotransposons were observed in protein-coding regions, while 50 distinct protein coding exons in 45 genes were comprised exclusively of LTR RetroTransposon Sequence (LRTS)…an alternatively spliced exon of the Interleukin 22 receptor, alpha 2 gene (*IL22RA2*) derived from a sequence of retrotransposon of the Mammalian apparent LTR retrotransposons (*MaLR*) family …hypothesize that the recruitment of the part of LTR as a novel exon…a result of a single mutation in the proto-splice site…”
Human	“…human nuclear prelamin A recognition factor contains a primate-specific *Alu*-exon that exclusively depends on RNA editing for its exonization.”

A more complete, detailed and fully referenced version of this table is available as Supplementary Table S11.

**Table 12:** Repetitive DNA Content of Some Annotated Animal Genomes (data source—http://www.repeatmasker.org/genomicDatasets/RMGenomicDatasetsAlt.html).

Species	Genome Size	Fraction Total Repetitive DNA (Interspersed Mobile DNA)
Primates		
Human *Homo sapiens*	3,049,315,783 bp	52.58% (48.49%)
Orangutan *Pongo pygmaeus abelii*	3,093,543,172 bp	52.16% (48.79%)
Gorilla *Gorilla gorilla gorilla*	2,822,760,080 bp	49.43% (46.12%)
Gibbon *Nomascus leucogenys*	2,756,609,047 bp	51.96% (47.90%)
Chimp *Pan troglodytes*	2,902,338,967 bp	51.72% (48.77%)
Other Mammals		
Mouse *Mus musculus*	2,652,783,500 bp	45.03% (41.73%)
Cow *Bos taurus*	2,804,673,174 bp	49.38% (47.98%)
Killer whale *Orcinus orca*	2,249,582,112 bp	44.53% (43.23%)
Dolphin *Tursiops truncatus*	2,332,402,443 bp	44.00% (41.24%)
Squirrel *Spermophilus tridecemlineatus*	2,311,060,300 bp	36.30% (34.32%)
Elephant *Loxodonta africana*	3,118,565,340 bp	57.63% (56.38%)
Microbat *Myotis lucifugus*	1,966,419,868 bp	37.03% (35.51%)
Megabat *Pteropus vampyrus*	1,839,436,660 bp	35.82% (33.40%)
Birds		
Chicken *Gallus gallus*	1,032,854,810 bp	11.47% (9.74%)
Zebra finch *Taeniopygia guttata*	1,222,864,691 bp	8.45% (7.17%)
Mallard duck *Anas platyrhynchos*	1,069,972,754 bp	6.53% (4.69%)
Reptiles		
Lizard *Anolis carolinensis*	1,701,353,767 bp	36.01% (34.26%)
Alligator *Alligator mississippiensis*	2,098,626,832 bp	37.68% (36.96%)
Gharial crocodile *Gavialis gangeticus*	2,139,715,393 bp	37.55% (36.86%)
Painted turtle *Chrysemys picta bellii*	2,158,289,746 bp	30.86% (30.20%)
Amphibian		
Western clawed frog *Xenopus tropicalis*	1,365,936,747 bp	34.91% (31.88%)
Fish		
Coelacanth *Latimeria chalumnae*	2,183,592,768 bp	9.13% (6.82%)
Stickleback *Gasterosteus aculeatus*	446,627,861 bp	5.78% (3.23%)
Fugu *Takifugu rubripes*	350,961,831 bp	8.66% (5.92%)
Spotted gar *Lepisosteus oculatus*	869,414,359 bp	1.20% (0.00%)
Lower vertebrate		
Lamprey *Petromyzon marinus*	647,368,134 bp	35.46% (30.35%)
Chordate		
Lancelet amphioxus *Branchiostoma floridae*	480,418,582 bp	13.63% (12.35%)
Insects		
Bee *Apis mellifera*	231,030,884 bp	6.21% (0.24%)
Mosquito *Anopheles gambiae*	263,156,584 bp	14.10% (11.34%)
Fruit fly *Drosophila melanogaster*	162,367,812 bp	28.61% (20.44%)
Mollusca		
Sea hare mollusk *Aplysia californica*	619,228,092 bp	13.00% (4.86%)
Echinoderm		
Sea Urchin *Strongylocentrotus purpuratus*	809,969,717 bp	18.00% (13.82%)
Nematodes		
*Caenorhabditis briggsae*	105,451,667 bp	20.07% (16.02%)
*Caenorhabditis elegans*	100,286,070 bp	12.59% (10.31%)
Tunicate		
*Ciona intestinalis*	141,233,565 bp	16.57% (14.82%)

**Table 13:** Distributed genome network innovation attributed to mobile DNA elements.

Organisms	Phenotypes
18 Fungal Genomes	Whole-Genome Architecture and Transcriptional Profiles
Plants	Epigenetic Controls
Plants	C4 photosynthesis
Plants	Stress Response
Maize	Abiotic Stress Response
Maize	Helitron transposons reshuffle the transcriptome
Cotton	Fiber cell development
*Coffea*	Drought stress response
*Drosophila*	X chromosome dosage compensation
Mammals	Estrogen receptor network
Mammals	Pregnancy
Human	c-Myc regulatory subnetwork
Human	Core embryonic stem cell development

A fully referenced version of this table is available as Supplementary Table S13.

**Table 14:** Diverse Regulatory Functions Reported for Long Non-coding lncRNA Molecules.

Organism or Taxon	Function(s)
Budding yeast	Galactose metabolism, controls speed of transcriptional induction by galactose
Fission yeast	Phosphate-induced epigenetic silencing
Budding, fission yeast	Cellular responses to environmental changes
Fungal pathogen *Cryptococcus neoformans*	lncRNA *RZE1* regulates yeast-to-hypha transition
*Plasmodium falciparum*	Gametocyte development; Telomere associated lncRNAs
Plants	Flower development and timing
*Arabidopsis*	Stress response lncRNAs; lncRNA *TER* regulates telomerase activity
Tomato	Fruit ripening
*Drosophila*	Sex determination; X chromosome dosage compensation
Tetrapods	Spermatogenesis, synaptic transmission, placenta development
Mammals	mRNA transcription; Stem cell pluripotency; Epigenetic chromatin formatting; Placental membrane integrity; Androgen receptor-regulated transcription; Synaptic connectivity in brain
Marsupial (*Monodelphis domestica*)	Female X chromosome inactivation by repeat-rich lncRNA *Rsx*
Goat (*Capra hircus*)	Skin pigmentation
Mouse	Enhancer methylation and transcription control; Diurnal metabolic regulation; Cell reprogramming to pluripotent stem cells; Imprinted *Igfr2* silencing; Myogenesis (SINE-containing lncRNA acting on *STAU-1* mRNA decay); lncRNA *Dum* regulates myogenic differentiation and muscle regeneration; Translation of brain UCHL1 protein involved in preventing neurodegeneration, regulation by *SINEB2* recognition; lncRNA *Evf2* modulates chromatin formation in forebrain development; lncRNA-*HIT* mediates TGF beta-induced epithelial to mesenchymal transition in mammary epithelia and is essential for chondrogenic differentiation in the limb mesenchyme (cartilage formation); lncRNA *Fendrr* regulates heart and body wall development; Male germline development
Rat	Long-term potentiation of synaptic connectivity in adult brain development
Primate	lncRNA *ANRIL* regulates expression of three cyclin-dependent kinase inhibitors and atherogenesis, *ANRIL Alu* exons acquired in primate lineage; Primate-specific lncRNA *HPAT-5* required for stem cell pluripotency
Human	18,871,097 lncRNA-RNA base-pairings likely involved in processing, stability control and functions of 57,303 transcripts; Centromere function; Steroid receptor activation; Endocrine regulation; Rb and p53 signaling pathways; lncRNA *RoR* is a p53 repressor in response to DNA damage and acts as microRNA sponge in transcription factor control; lncRNA *LED* stimulates p53 activated transcription; lncRNA *H19* modulates S-adenosylhomocysteine hydrolase and DNA methylation; lncRNA *HOTAIR* regulates chromatin dynamics; lncRNA *NBR2* regulates AMP-activated protein kinase under energy stress; lncRNA *Xist* required for X inactivation; lncRNA *XACT* in active X chromosome expression; Apoptosis and lysosomal processes (*Alu* recognition by lncRNA *GAS5*); *STAU-1* mRNA decay (stimulated by *Alu* pairing); Cell cycle regulation by lncRNA *APTR*; lncRNA *Firre* controls mRNA retention in the nucleus, nucleolar anchoring of inactive X; lncRNA *ROR* regulates stem cell pluripotency; has sequences from >12 mobile elements, including a long *HERVH* 5’ sequence characteristic of several pluripotent cell lncRNAs, acts as microRNA sponge; Stem cell specificities; Stem cell pluripotency and cancer cell proliferation, transcription from retroviral promoters; Pluripotency and neuronal differentiation regulation of chromatin modifiers and transcription factors; lncRNA-mediated regulation of the interferon response; lncRNA *ANRIL* regulates inflammatory responses as a novel component of NF-kappaB pathway; Innate and adaptive immune responses; lncRNA *TINCR* regulates epidermal differentiation; Neurodevelopment and brain function; Chromatin modification, epigenetic regulation, alternative splicing, and translational control by *MALAT1*, *HOTAIR* and *TRE* lncRNAs represent important examples of lncRNA-mediated control of cell migration and invasion, epithelial-to-mesenchyme transition and metastasis

A more detailed and fully referenced version of this table is available as Supplementary Table S14.

**Table 15:** Diverse Mutagenic Natural Genetic Engineering Outcomes.

Mutation Type	Biochemical Activity
Single nucleotide substitutions	Y-family mutagenic trans-lesion DNA polymerase; error-prone repair systems
Frameshifts	Y-family mutagenic trans-lesion DNA polymerase; error-prone repair systems
Deletions (bacteria)	Y-family mutagenic trans-lesion DNA polymerase; error-prone repair systems
Deletions and translocations (often with microhomologies)	Mre11, CltP exonucleases; canonical or alternative non-homologous end-joining (NHEJ) complexes
Deletions and translocations	Non-allelic homologous recombination (NAHR) between mobile DNA repeats
Deletion	Elevated transcription, Topoisomerase I; *LINE-1*-mediated; non-allelic homologous recombination between dispersed mobile repeats
Translocations	Nonhomologous end joining or microhomology-mediated break-induced replication
Somatic hypermutation and kataegis: multiple clustered nucleotide substitutions	AID or APOBEC cytosine deaminase
Chromothripsis and complex chromosome segment insertions	Loss of p53-dependent checkpoints; Rad51 homologous recombination; NHEJ; premature chromosome condensation; segregation of chromosome breakage and repair into a micronucleus; L1-Mediated Retrotransposition and *Alu*-*Alu* NAHR (Not all these processes are involved in each chromothripsis event.)

A more detailed and fully referenced version of this table is available as Supplementary Table S15.

**Table 16:** Ecological Factors that Induce Mutagenic DNA Repair or Modulate Natural Genetic Engineering (NGE) Responses.

Ecological Factors and NGE Effects	Affected Organisms
**Growth conditions and cellular differentiation**	
Stationary phase mutagenesis; anaerobic growth enhanced point mutations; aging colonies produce mutational hotspots and retromutations (8-oxo-guanosine, formed exclusively on the transcribed strand); adaptive selection-induced retromutations; nutrient-dependent mutability (Phosphorus/carbon limitation increase point mutations, iron/oxygen/carbon limitation increase *IS150* insertions, and phosphorus limitation increases indels)	*B. subtilis* and *E. coli*
Cystic Fibrosis lung growth hypermutability	*P. aeruginosa*
Adenine starvation stimulates *Ty1* retrotransposition; transcription induces APOBEC kataegis; Glucose- or phosphate-limited growth produced frequent genomic amplifications, rearrangements and novel retrotransposition; starvation leads to genome restructuring but has <2× effect on point mutation; nitrogen starvation increases copy number variations (CNVs)	Yeast *Saccharomyces cerevisaea*
Domestication leads to increase in repetitive DNA and retrotransposons	Maize
Early embryogenesis activates *mPing* DNA transposition	Rice
Plant regeneration activates chromovirus *LORE1* (ERV) retrotransposition	Model legume *Lotus japonicus*
Neural differentiation activates *L1* retrotransposition.	Rodents, humans
Aging induces retrotransposition (effect counter-acted by calorie restriction)	Mouse germline and somatic tissue
Early embryonic development displays a mutator state for copy number variation (CNV) of genomic duplications	Humans
**Abiotic stresses**	
UV irradiation stimulates hypermutation; oxidative stress induces DNA transposon non-allelic homolgous recombination (NAHR)	*Pseudomonas aeruginosa* and *Burkholderia cenocepacia*
Copper induces expansion and contraction of *CUP1* arrays encoding copper-binding protein (copy number variation, CNV)	Budding yeast *Saccharomyces cerevisaea*
Heat shock, oxidative and copper sulphate stresses activate LTR-retrotransposons Pyret and MAGGY, DNA transposons Pot3, MINE, Mg-SINE, Grasshopper and MGLR3	Fungal pathogen *Magnaporthe oryzae*
Mild heat stress and UV activate *mariner*-*Mos1* transposition	*Drosophila simulans*
Uranium induces alternative NHEJ DSB repair processes	Embryonic zebrafish cells
“Two mechanisms … of cadmium mutagenicity: (i) induction of reactive oxygen species (ROS); and (ii) inhibition of DNA repair.”	Various mammals
Arsenic, vanadium, iron induce *VL30* retrotransposition	Mouse NIH3T3 cells
“Environmental stressors such as ionizing radiation (terrestrial, space, and UV-radiation), air pollution (including particulate matter {PM}-derived and gaseous), persistent organic pollutants, and metals” activate mobile DNA elements; mercury induces *LINE1* retrotransposition; low doses of NiCl_2_ and CdCl_2_ contributed to an increase in mutagenic deletions by *Alu*-*Alu* NAHR…cells exposed to arsenic trioxide preferentially repaired using the “error prone” non-homologous end joining (alt-NHEJ) while inhibiting repair by HR; Aluminum and low-level As_2_O_3_ induce *LINE1* retrotransposition while copper treatment downregulated *L1* retrotransposition; exposure to CdCl_2_ and CdAc_2_ inhibits NHEJ and activates MRE11-dependent repair; Cold, heat, hypoxic, and oxidative stresses induce trinucleotide repeat mutagenesis	Human cells and tissues
Heat stress activates *ONSEN*, *COPIA* retrotransposition	*Brassicaceae* and *Arabidopsis*
Microsatellite mutation rate is significantly greater at 26 °C than at 18 °C	*C. elegans*
Hyper salinity, stressed lineages accumulate ∼100% more mutations, and these mutations exhibit a distinctive molecular mutational spectrum (specific increases in relative frequency of transversion and insertion/deletion {indel} mutations).	*A. thaliana*
Nitric oxide modulator, sodium nitroprusside induces *Tos17* LTR retrotransposition; laser irradiation stimulates DNA methylation changes and *mPing* DNA transposition	Rice
Fungicides boscalid (respiration inhibitor), iprodione (unclear mode of action), thiophanate methyl (inhibition of microtubulin synthesis) and azoxystrobin and pyraclostrobin (quinone outside inhibitors) raised mutation rates 1.7- to 60-fold compared to neutral conditions.	Plant pathogen *Sclerotinia sclerotiorum*
**Biotic Stresses and Biomolecules**	
Ethanol stress induces transient hypermutator state; food additive sepiolite stimulates antibiotic resistance plasmid transfer	*E. coli* and other bacteria
Joint action of LL-37 (antimicrobial peptide) and free iron induces mutagenesis	*P. aeruginosa*
Antibiotics induce SOS response and conjugal DNA transfer	*V. cholera*, *Pseudomonas aeruginosa*
Fluoroquinolone and norfloxacin antibiotics induced point mutations, *IS1* NAHR deletions, *IS5* NAHR duplications (but not transpositions)	*E. coli*
Beta-lactam antibiotics induced RpoS-dependent mutagenesis	*E. coli*
Subinhibitory concentrations of ciprofloxacin and vancomycin activate *IS256* transposition, induce SOS response	*Staphylococcus aureus*
Tigecycline induces hypermutation	*Acinetobacter baumannii*
Cationic antimicrobial peptide human cathelicidin LL-37 induces mutagenesis in CF lungs	*P. aeruginosa*
Canavanine proteotoxic stress induces mutagenesis	Yeast *S. cerevisaea*
Infection by the following bacteria can produces DNA damage, genome instabilities and alterations in DNA repair activities: *Chlamydia trachomatis*, *N. gonorrhea*, *Helicobacter pylori*, *E. coli*, *Campylobacter jejuni*, *Haemophilus ducreyi*, *Actinobacillus actinomycetemcomitans*, *Shigella dysenteriae*, *Helicobacter cinaedi*, *Helicobacter hepaticus*, *Salmonella* species, *Shigella* strains, *Klebsiella pneumoniae*, *Enterobacter aerogenes*, *Citrobacter koseri*, *P. aeruginosa*, *Listeria monocytogenes*	Human cells
Infection by the following viruses can produce DNA damage, genome instabilities and alterations in DNA repair activities: human cytomegalovirus, Human T-cell lymphotropic virus 1 (HTLV-1) retrovirus, and Zika virus.	Human cells
*P. syringae* pathovar *tomato* infection induces DSB formation… abundance of infection-induced DSBs reduced by salicylic acid	*Arabidopsis*
Attack by the *oomycete* pathogen *Peronospora parasitica* stimulates somatic recombination	*Arabidopsis*
Tobacco mosaic virus (TMV) or oilseed rape mosaic virus (ORMV) tobacco leaf infection resulted in a systemic increase in homologous recombination (HR)…a similar phenomenon occurs in *Arabidopsis thaliana* plants infected with ORMV.	*Arabidopsis*, tobacco
*Bs1* Transposition detected in maize lines following barley stripe mosaic virus infection	*Zea mays*
Physiological stress, induced by climate change or invasion of new habitats, disrupts epigenetic regulation and activates mobile DNA elements	Diverse organisms

A more detailed and fully referenced version of this table is available as Supplementary Table S16.

**Table 17:** Different Genome Changes Observed in Cancer Cells.

Stress-Induced Mutagenic Activity
Hypermutability following loss of replication proofreading functions
Massive genome rearrangements (“karyotype chaos”)
Homology-independent rearrangements (NHEJ)
Retrotransposon activation
Non-canonical termination of homologous recombination
Kataegis and somatic hypermutation
Cytosine deaminase-dependent chromosome translocation
Chromothripsis
Chromothripsis linked to oncogene amplification
Complex insertion-deletion mutations (indels)
Tandem duplications as well as formation of “amplicons” with rearranged and amplified chromosomal segments, *a.k.a.* copy number variations (CNVs)
Formation of amplified circular extrachromosomal DNAs
Processed pseudogene formation
L1 retrotransposition
Extensive L1 retrotransduction of non-repetitive DNA
Transfer of mitochondrial DNA into nuclear genome
RAG transposase/recombinase-mediated chromosome rearrangement in immune system tumors
Somatic hypermutation involving a reverse transcriptase-based mutator activity

A fully referenced version of this table is available as Supplementary Table S17. (See also http://shapiro.bsd.uchicago.edu/Cancer%20Genome%20Changes.pdf).

## Supplementary Materials

The following are available online at www.mdpi.com/2079-7737/6/4/42/s1, Supplementary Table S1: Key Scientists Advocating non-Gradualist Evolution in the 19th and 20th Centuries, Table S2: No supplementary table needed (To keep table numbers to be the same in the text and Supplementary Material, the author added Table S2), Table S3: Photosynthetic eukaryotic lineages resulting from symbiogenesis, Table S4: Selected Examples of Speciation and Adaptive Radiation Involving Interspecific Hybridization and Changes in Chromosome Number, Table S5: Genomic consequences of experimental interspecific hybridization in plants and animals, Table S6: Modes of Horizontal DNA Transfer, Table S7: Selected examples of inter-phylum adaptive horizontal DNA transfers based on genomic data, Table S8: Highly cited protein domain search terms in the PubMed database, Supplementary Table S9: Selected Reports of Exon Rearrangements and Retroposition by Mobile DNA element Activity, Table S10: Some Reported Instances of Orphan Coding Sequences (“New genes” and New exons) Discovered in Sequenced Genomes, Table S11: Origination of Novel Exons from Mobile DNA Elements, Table S12, No supplementary table needed (To keep table numbers to be the same in the text and Supplementary Material, the author added Table S12), Table S13: Distributed genome network innovation attributed to mobile DNA elements, Table S14: Diverse Regulatory Functions Reported for Long Non-coding lncRNA molecules, Table S15: Diverse Mutagenic Natural Genetic Engineering Activities, Table S16: Ecological Factors that Induce Mutagenic DNA Repair or Modulate NGE Responses, Table S17: Different Genome Changes Observed in Cancer Cells.

## Abbreviations

**Ac**	long interspersed nucleotide element
**AID**	activation-induced deaminase
**AP**	apurinic
**BER**	base excision repair
**BFB**	breakage-fusion-bridge cycle
**CDS**	coding sequence
**CDT**	cytolethal distending toxin
**CNV**	copy number variation
**CRISPR**	clustered regularly interspaced short palindromic repeats
**DDR**	DNA damage response
**DGR**	diversity-generating retroelement
**dPRL**	decidual prolactin
**Ds**	Dissociator transposon
**DSB**	double-strand break
**ENV**	envelope protein (retrovirus)
**ERV**	endogenous retrovirus
**ESC**	embryonic stem cell
**HERV/hERV**	human ERV
**HGT**	horizontal gene transfer
**HR**	homologous recombination
**Indel**	insertion plus deletion
**IPSC**	induced pluripotent stem cell
**IR**	inverted repeat
**lincRNA**	long intergenic non-coding RNA
**LINE**	long interspersed nucleotide element
**lncRNA**	long non-coding RNA
**LSG**	lineage-specific gene
**LTRs**	long terminal repeats
**MaLR**	mammalian apparent LTR-retrotransposon
**MER**	mammalian endogenous repeat
**mERV**	mouse endogenous retrovirus
**MIR**	mammalian-wide interspersed repeat
**miRNA**	microRNA
**MMR**	mutagenic mismatch repair
**mRNA**	messenger RNA
**MT**	mouse transposon
**NAHR**	non-allelic homologous recombination
**NCLDV**	nucleocytoplasmic large DNA virus
**NEE**	novel enriched environment
**NER**	nucleotide excision repair
**NGE**	natural genetic engineering
**NHEJ**	non-homologous end-joining
**ORF**	open reading frame
**ORR**	origin region repeat
**RAG**	recombination-activating gene, transposase activity needed for V(D)J recombination in adaptive immunity
**RIDL**	repeat insertion domains of lncRNA
**RLEs**	retrovirus-like elements
**ROS**	reactive oxygen species
**SINE**	short interspersed nucleotide element
**SOS**	DNA damage signal-inducible repair response in bacteria
**SSB**	single-strand break
**SSR**	simple sequence repeat
**SVA**	a hominid retrotransposon containing SINE, VNTR and *Alu* components
**TE**	transposable element
**WGD**	whole genome duplication
