# Supplementary material for: Living Organisms Author Their Read-Write Genomes in Evolution
Source: Biology (Basel). 2017 Dec 6;6(4):42. doi: 10.3390/biology6040042 (PMC5745447; doi:10.3390/biology6040042)
Supplement: Supplementary file 1 [file biology-06-00042-s001.tgz › biology-224185-supplementary & PUBMED links/biology-224185.zip/Shapiro - Living Organisms Author Their Read-Write Genomes in Evolution - Supplemental Material.Renumbered and Approved + PUBMED links/Additional References for Living Organisms Author Their Read.docx]

**Additional References for** **Living Organisms Author Their Read-Write Genomes in Evolution**

**Section 2.** Parsing the Fundamental Question in Evolution: How do Heritable Adaptive Novelties and New Groups of Organisms Arise?[[1-13](#_ENREF_1)].

**Section 3.** Biomath: One + One = One [[14](#_ENREF_14), [15](#_ENREF_15)]; Ubiquitous Cell Mergers in Reproduction and Evolution

**3.1.** Symbiogenetic Origins of Eukaryotic Cells and Their Photosynthetic Lineages [[16-72](#_ENREF_16)]

**3.2.** Symbiosis as an Adaptive and Evolutionary Stimulus; Speciation by Endosymbiosis and Mating Incompatibility [[73-135](#_ENREF_73)]

Symbiont effects on complex host phenotypes: [[86](#_ENREF_86), [91-101](#_ENREF_91), [136-148](#_ENREF_136)]

**3.3.** Holobiont Evolution: Lamarckian Acquisition and Inheritance of Novel Traits [[1-5](#_ENREF_1), [125](#_ENREF_125), [149-160](#_ENREF_149)]

**Section 4.1.** Abundant Examples of Speciation and Adaptive Radiations by Interspecific Hybridization and Whole Genome Duplications (WGDs) in Plants and Animals

**Genome duplications in vertebrate origins;** [[161](#_ENREF_161)]

**Cichlids:** [[162-169](#_ENREF_162)]

**Section 5.** Widespread Horizontal DNA Sequence Mobility between Organisms [[170-172](#_ENREF_170)].

**5.2.** Lessons on Rapid Evolution from the Smallest Living Cells [[106](#_ENREF_106), [173-193](#_ENREF_173)]

**5.3.** Horizontal DNA Transfer across Large Taxonomic Boundaries [[194-199](#_ENREF_194)]

**Section 6** Genome Writing by Natural Genetic Engineering—Protein Evolution by Natural Genetic Engineering, Exon Rearrangements and Exon Originations

**6.1.** The Modular Domain-Based Structure of Proteins [[200-217](#_ENREF_200)]

**6.2.** Protein Evolution by Exon Shuffling and Exon Accumulation, Changes to Alternative Splicing Patterns, and Insertion of Reverse-Transcribed Coding Sequences [[218-241](#_ENREF_218)]

- **Chimeric proteins from trans-splicing plus retroposition and from NHEJ:** [[242-256](#_ENREF_242)].

**6.3.** Protein Evolution by Domain/Exon Origination [[257-280](#_ENREF_257)]

- **Synthesis of novel coding sequences without a template:** Terminal transferase enzymes synthesize nucleic acids with a template determining sequence [[281-283](#_ENREF_281)]. Terminal deoxyribonucleotidyltransferase (TdT) is utilized to generate novel DNA-coding segments (“N region diversity”) in formation of antibody heavy chains and other immune system receptor proteins [[284-286](#_ENREF_284)]. X family DNA polymerases, including TdT, insert untemplated nucleotides at NHEJ joining of broken DNA molecules, a process which also generates DNA sequences that did not previously exist in the genome [[287-291](#_ENREF_287)]. In cancer cells, there is evidence that error-prone trans-lesion DNA polymerases important in microhomology-mediated chromosome rearrangements [[292](#_ENREF_292)]. RNA-templated DNA can also participate in NHEJ DS break repair to introduce novel sequences at the repair junction [[293-297](#_ENREF_293)]. TdT action has also been identified in cancer cells [[298](#_ENREF_298), [299](#_ENREF_299)].

RNA-templated DNA synthesis is more error-prone than DNA replication because reverse transcriptases lacks the exonuclease-proofreading domains of replicative DNA polymerases [[300](#_ENREF_300), [301](#_ENREF_301)]. Thus, many retroposed DNAs inserted into the genome have altered sequences from the original DNA template. Significant post-transcriptional processing of RNAs, including *cis*- and *trans*-splicing and chemical modifications to individual nucleotides, such as RNA editing by cytidine deamination to uracil [[302](#_ENREF_302), [303](#_ENREF_303)] and adenosine deamination to inosine (which base-pairs like guanosine) [[304-306](#_ENREF_304)]. So RNA templates can be significantly modified prior to reverse transcription, and there is even a report of template-independent DNA synthesis by a retrotransposon-encoded reverse transcriptase [[307](#_ENREF_307)]. Furthermore, retroposed DNA sequences come from non-coding as well as protein-coding RNA molecules, and non-coding retroelements can contribute completely novel coding exons if their transcripts can be spliced into mRNAs [[308](#_ENREF_308)]. As with other mutagenic processes [[309-312](#_ENREF_309)], retroposition of cellular RNAs has been observed to occur in real time in cancer origins and progression [[313](#_ENREF_313)].

**Section 7.** Genome Writing by Natural Genetic Engineering: Mobile and Repetitive DNA Elements Actively Contributing to Genome Organization, Organismal Complexity and Genome Regulation

**7.1.** Regulatory Studies Led to Recognizing the Syntactical Organization of Genomes**.** [[314-319](#_ENREF_314)]

**7.2.** Repetitive DNA Elements Provide Distributed Copies of Each Class of Regulatory Site [[318](#_ENREF_318), [320-323](#_ENREF_320)]

**7.3.** How Do Organisms Use Repetitive DNA for Genome Rewriting in Evolution? Dispersed Mobile DNA Elements [[324-337](#_ENREF_324)]

**7.4.** Rewiring Transcriptional Regulatory Networks in Evolution of Complex Organisms [[338-346](#_ENREF_338)]

**7.5.** Mobile DNA Elements Are Major Contributors to “Non-Coding” Regulatory RNA Molecules [[347-355](#_ENREF_347)]

**Section 8.** Ecological Disruption and Read-Write Genome Modifications

**8.2.** Regulated Biochemistry at the Basis of Point Mutations, Deletions, Translocations and Mutational “Storms”[[356-358](#_ENREF_356)]

**Section 9.** Further Reflections on Genome Rewriting by NGE As a Core Biological Capability

**9.1.8.** Trypanosome Antigenic Variation [[359-361](#_ENREF_359)] OK

**9.2.** Lessons on the Real Time Potential of Natural Genetic Engineering from Cancer Genomes [[362-365](#_ENREF_362)]

Cancer genome changes are tumor specific: (1) L1 retrotransposition repeatedly observed in three epithelial cancers (colorectal, prostate, and ovarian), no insertions found in blood and brain cancers [[366](#_ENREF_366)]. (2) Chromothripsis prevalence vary from 0 in basal type breast cancer and ovarian cancer to 100% in “Sonic Hedgehog (SHH) medulloblastoma with mutant *TP53*,” twelve other tumor types show chromothripsis prevalence above 30% [[367](#_ENREF_367)]. Chromothripsis alters different chromosome regions in distinct tumor types [[368](#_ENREF_368)]. In SHH meduloblastomas, amplified regions typically contain medulloblastoma oncogenes, such as SHH pathway members *MYCN*, *GLI2* and *BOC* [[369](#_ENREF_369)]. (3) Complex indels display strong tissue specificity (*VHL* in kidney cancer, *GATA3* in breast cancer) [[370](#_ENREF_370)]. (4) Certain cancers display repeat changes, such as “Philadelphia chromosome” translocation in chronic myeloid leukemias but not other tumors [[371](#_ENREF_371)].

**9.3.** What Factors May Bias Genome Rewriting to Generate Selectively Positive Outcomes**?** [[372-378](#_ENREF_372)]

REFERENCES

1. Guerrero, R., L. Margulis, and M. Berlanga, *Symbiogenesis: the holobiont as a unit of evolution.* Int Microbiol, 2013. **16**(3): p. 133-43. <http://www.ncbi.nlm.nih.gov/pubmed/24568029>.

2. Salvucci, E., *Microbiome, holobiont and the net of life.* Crit Rev Microbiol, 2014: p. 1-10. <http://www.ncbi.nlm.nih.gov/pubmed/25430522>.

3. Vandenkoornhuyse, P., et al., *The importance of the microbiome of the plant holobiont.* New Phytol, 2015. **206**(4): p. 1196-206. <http://www.ncbi.nlm.nih.gov/pubmed/25655016>.

4. Bordenstein, S.R. and K.R. Theis, *Host Biology in Light of the Microbiome: Ten Principles of Holobionts and Hologenomes.* PLoS Biol, 2015. **13**(8): p. e1002226. <http://www.ncbi.nlm.nih.gov/pubmed/26284777>.

5. Zilber-Rosenberg, I. and E. Rosenberg, *Role of microorganisms in the evolution of animals and plants: the hologenome theory of evolution.* FEMS Microbiol Rev, 2008. **32**(5): p. 723-35. <http://www.ncbi.nlm.nih.gov/pubmed/18549407>.

6. Mallet, J., *Hybridization as an invasion of the genome.* Trends Ecol Evol, 2005. **20**(5): p. 229-37. <http://www.ncbi.nlm.nih.gov/pubmed/16701374>.

7. Chaffron, S., et al., *A global network of coexisting microbes from environmental and whole-genome sequence data.* Genome Res, 2010. **20**(7): p. 947-59. <http://www.ncbi.nlm.nih.gov/pubmed/20458099>.

8. Grassi, L., J. Grilli, and M.C. Lagomarsino, *Large-scale dynamics of horizontal transfers.* Mob Genet Elements, 2012. **2**(3): p. 163-167. <http://www.ncbi.nlm.nih.gov/pubmed/23061026>.

9. Koonin, E.V., *Horizontal gene transfer: essentiality and evolvability in prokaryotes, and roles in evolutionary transitions.* F1000Res, 2016. **5**. <http://www.ncbi.nlm.nih.gov/pubmed/27508073>.

10. Lacroix, B. and V. Citovsky, *Transfer of DNA from Bacteria to Eukaryotes.* MBio, 2016. **7**(4). <http://www.ncbi.nlm.nih.gov/pubmed/27406565>.

11. Soucy, S.M., J. Huang, and J.P. Gogarten, *Horizontal gene transfer: building the web of life.* Nat Rev Genet, 2015. **16**(8): p. 472-82. <http://www.ncbi.nlm.nih.gov/pubmed/26184597>.

12. Gao, C., et al., *Horizontal gene transfer in plants.* Funct Integr Genomics, 2013. <http://www.ncbi.nlm.nih.gov/pubmed/24132513>.

13. Rosenzweig, B.K., et al., *Powerful methods for detecting introgressed regions from population genomic data.* Mol Ecol, 2016. **25**(11): p. 2387-97. <http://www.ncbi.nlm.nih.gov/pubmed/26945783>.

14. Margulis, L. and *Symbiogenesis. A new principle of evolution rediscovery of Boris Mikhaylovich Kozo-Polyansky (1890–1957).* Paleontological Journal, 2010. **44**(12): p. 1525–1539. .

15. Kozo-Polyansky, B.M., *Symbiogenesis: A New Principle of Evolution (1924)*, ed. E.a.t.b.V. Fet and E.b.L. Margulis2010, Cambridge, MA: Harvard University Press. .

16. Martin, W.F., S. Garg, and V. Zimorski, *Endosymbiotic theories for eukaryote origin.* Philos Trans R Soc Lond B Biol Sci, 2015. **370**(1678): p. 20140330. <http://www.ncbi.nlm.nih.gov/pubmed/26323761>.

17. Ku, C., et al., *Endosymbiotic origin and differential loss of eukaryotic genes.* Nature, 2015. **524**(7566): p. 427-32. <http://www.ncbi.nlm.nih.gov/pubmed/26287458>.

18. Meheust, R., P. Lopez, and E. Bapteste, *Metabolic bacterial genes and the construction of high-level composite lineages of life.* Trends Ecol Evol, 2015. **30**(3): p. 127-9. <http://www.ncbi.nlm.nih.gov/pubmed/25601290>.

19. Lane, C.E. and J.M. Archibald, *The eukaryotic tree of life: endosymbiosis takes its TOL.* Trends Ecol Evol, 2008. **23**(5): p. 268-75. <http://www.ncbi.nlm.nih.gov/pubmed/18378040>.

20. Meheust, R., et al., *Protein networks identify novel symbiogenetic genes resulting from plastid endosymbiosis.* Proc Natl Acad Sci U S A, 2016. **113**(13): p. 3579-84. <http://www.ncbi.nlm.nih.gov/pubmed/26976593>.

21. Nasir, A., et al., *Arguments Reinforcing the Three-Domain View of Diversified Cellular Life.* Archaea, 2016. **2016**: p. 1851865. <http://www.ncbi.nlm.nih.gov/pubmed/28050162>.

22. Burki, F., *The eukaryotic tree of life from a global phylogenomic perspective.* Cold Spring Harb Perspect Biol, 2014. **6**(5): p. a016147. <http://www.ncbi.nlm.nih.gov/pubmed/24789819>.

23. Burki, F., K. Shalchian-Tabrizi, and J. Pawlowski, *Phylogenomics reveals a new 'megagroup' including most photosynthetic eukaryotes.* Biol Lett, 2008. **4**(4): p. 366-9. <http://www.ncbi.nlm.nih.gov/pubmed/18522922>.

24. Lane, N., *Plastids, genomes, and the probability of gene transfer.* Genome Biol Evol, 2011. **3**: p. 372-4. <http://www.ncbi.nlm.nih.gov/pubmed/21292628>.

25. Sousa, F.L., et al., *Early bioenergetic evolution.* Philos Trans R Soc Lond B Biol Sci, 2013. **368**(1622): p. 20130088. <http://www.ncbi.nlm.nih.gov/pubmed/23754820>.

26. Lane, N., *Energetics and genetics across the prokaryote-eukaryote divide.* Biol Direct, 2011. **6**: p. 35. <http://www.ncbi.nlm.nih.gov/pubmed/21714941>.

27. Lane, N. and W. Martin, *The energetics of genome complexity.* Nature, 2010. **467**(7318): p. 929-34. <http://www.ncbi.nlm.nih.gov/pubmed/20962839>.

28. Price, D.C., et al., *Cyanophora paradoxa genome elucidates origin of photosynthesis in algae and plants.* Science, 2012. **335**(6070): p. 843-7. <http://www.ncbi.nlm.nih.gov/pubmed/22344442>.

29. Spring, J., *Major transitions in evolution by genome fusions: from prokaryotes to eukaryotes, metazoans, bilaterians and vertebrates.* J Struct Funct Genomics, 2003. **3**(1-4): p. 19-25. <http://www.ncbi.nlm.nih.gov/pubmed/12836681>.

30. van der Giezen, M. and J. Tovar, *Degenerate mitochondria.* EMBO Rep., 2005. **6**: p. 525-530. .

31. van der Giezen, M., *Endosymbiosis: past and present.* Heredity, 2005. **95**(5): p. 335-6. <http://www.ncbi.nlm.nih.gov/pubmed/15931237>.

32. Koumandou, V.L., et al., *Molecular paleontology and complexity in the last eukaryotic common ancestor.* Crit Rev Biochem Mol Biol, 2013. **48**(4): p. 373-96. <http://www.ncbi.nlm.nih.gov/pubmed/23895660>.

33. Hackstein, J.H., J. Tjaden, and M. Huynen, *Mitochondria, hydrogenosomes and mitosomes: products of evolutionary tinkering!* Curr Genet, 2006. **50**(4): p. 225-45. <http://www.ncbi.nlm.nih.gov/pubmed/16897087>.

34. Lithgow, T. and A. Schneider, *Evolution of macromolecular import pathways in mitochondria, hydrogenosomes and mitosomes.* Philos Trans R Soc Lond B Biol Sci, 2010. **365**(1541): p. 799-817. <http://www.ncbi.nlm.nih.gov/pubmed/20124346>.

35. Dolezal, P., et al., *Evolution of the molecular machines for protein import into mitochondria.* Science, 2006. **313**(5785): p. 314-8. <http://www.ncbi.nlm.nih.gov/pubmed/16857931>.

36. Rotte, C., et al., *Origins of hydrogenosomes and mitochondria.* Curr Opin Microbiol, 2000. **3**(5): p. 481-6. <http://www.ncbi.nlm.nih.gov/pubmed/11050446>.

37. Hjort, K., et al., *Diversity and reductive evolution of mitochondria among microbial eukaryotes.* Philos Trans R Soc Lond B Biol Sci, 2010. **365**(1541): p. 713-27. <http://www.ncbi.nlm.nih.gov/pubmed/20124340>\.

38. Gray, M.W., B.F. Lang, and G. Burger, *Mitochondria of protists.* Annu Rev Genet, 2004. **38**: p. 477-524. <http://www.ncbi.nlm.nih.gov/pubmed/15568984>.

39. Gray, M.W., G. Burger, and B.F. Lang, *Mitochondrial evolution.* Science, 1999. **283**(5407): p. 1476-81. <http://www.ncbi.nlm.nih.gov/pubmed/10066161>.

40. Lang, B.F., M.W. Gray, and G. Burger, *Mitochondrial genome evolution and the origin of eukaryotes.* Annu Rev Genet, 1999. **33**: p. 351-97. <http://www.ncbi.nlm.nih.gov/pubmed/10690412>.

41. Bhattacharya, D., H.S. Yoon, and J.D. Hackett, *Photosynthetic eukaryotes unite: endosymbiosis connects the dots.* Bioessays, 2004. **26**(1): p. 50-60. <http://www.ncbi.nlm.nih.gov/pubmed/14696040>.

42. Rockwell, N.C., J.C. Lagarias, and D. Bhattacharya, *Primary endosymbiosis and the evolution of light and oxygen sensing in photosynthetic eukaryotes.* Front Ecol Evol, 2014. **2**(66). <http://www.ncbi.nlm.nih.gov/pubmed/25729749>.

43. Gross, J. and D. Bhattacharya, *Endosymbiont or host: who drove mitochondrial and plastid evolution?* Biol Direct, 2011. **6**: p. 12. <http://www.ncbi.nlm.nih.gov/pubmed/21333023>.

44. Reyes-Prieto, A., et al., *Differential gene retention in plastids of common recent origin.* Mol Biol Evol, 2010. **27**(7): p. 1530-7. <http://www.ncbi.nlm.nih.gov/pubmed/20123796>.

45. Nosenko, T., et al., *Chimeric plastid proteome in the Florida "red tide" dinoflagellate Karenia brevis.* Mol Biol Evol, 2006. **23**(11): p. 2026-38. <http://www.ncbi.nlm.nih.gov/pubmed/16877498>.

46. Moreira, D., L. Ranjard, and P. Lopez-Garcia, *The nucleolar proteome and the (endosymbiotic) origin of the nucleus.* Bioessays, 2004. **26**(10): p. 1144-5; author reply 1145-7. <http://www.ncbi.nlm.nih.gov/pubmed/15382131>.

47. Deschamps, P. and D. Moreira, *Signal conflicts in the phylogeny of the primary photosynthetic eukaryotes.* Mol Biol Evol, 2009. **26**(12): p. 2745-53. <http://www.ncbi.nlm.nih.gov/pubmed/19706725>.

48. Moreira, D. and P. Lopez-Garcia, *Symbiosis between methanogenic archaea and delta-proteobacteria as the origin of eukaryotes: the syntrophic hypothesis.* J Mol Evol, 1998. **47**(5): p. 517-30. <http://www.ncbi.nlm.nih.gov/pubmed/9797402>.

49. Moreira, D. and P. Deschamps, *What was the real contribution of endosymbionts to the eukaryotic nucleus? Insights from photosynthetic eukaryotes.* Cold Spring Harb Perspect Biol, 2014. **6**(7): p. a016014. <http://www.ncbi.nlm.nih.gov/pubmed/24984774>.

50. Gomez, F., P. Lopez-Garcia, and D. Moreira, *Molecular phylogeny of the ocelloid-bearing dinoflagellates erythropsidinium and warnowia (warnowiaceae, dinophyceae).* J Eukaryot Microbiol, 2009. **56**(5): p. 440-5. <http://www.ncbi.nlm.nih.gov/pubmed/19737196>.

51. Deschamps, P., et al., *Metabolic symbiosis and the birth of the plant kingdom.* Mol Biol Evol, 2008. **25**(3): p. 536-48. <http://www.ncbi.nlm.nih.gov/pubmed/18093994>.

52. Reyes-Prieto, A., A.P. Weber, and D. Bhattacharya, *The origin and establishment of the plastid in algae and plants.* Annu Rev Genet, 2007. **41**: p. 147-68. <http://www.ncbi.nlm.nih.gov/pubmed/17600460>.

53. Bhattacharya, D., et al., *Genome of the red alga Porphyridium purpureum.* Nat Commun, 2013. **4**: p. 1941. <http://www.ncbi.nlm.nih.gov/pubmed/23770768>.

54. Hackett, J., Anderson, DM, Erdner, DL, Bhattacharya, D, *Dinoflagellates: A remarkable evolutionary experiment.* Am J Bot, 2004. **91**: p. 1523–1534. .

55. Nowack, E.C. and M. Melkonian, *Endosymbiotic associations within protists.* Philos Trans R Soc Lond B Biol Sci\, 2010. **365\**(1541\): p. 699-712\. <http://www.ncbi.nlm.nih.gov/pubmed/20124339>\.

56. Nowack, E.C. and A.R. Grossman, *Trafficking of protein into the recently established photosynthetic organelles of Paulinella chromatophora.* Proc Natl Acad Sci U S A, 2012. **109**(14): p. 5340-5. <http://www.ncbi.nlm.nih.gov/pubmed/22371600>.

57. Mackiewicz, P., A. Bodyl, and P. Gagat, *Possible import routes of proteins into the cyanobacterial endosymbionts/plastids of Paulinella chromatophora.* Theory Biosci, 2012. **131**(1): p. 1-18. <http://www.ncbi.nlm.nih.gov/pubmed/22209953>.

58. Dorrell, R.G. and A.G. Smith, *Do red and green make brown?: perspectives on plastid acquisitions within chromalveolates.* Eukaryot Cell, 2011. **10**(7): p. 856-68. <http://www.ncbi.nlm.nih.gov/pubmed/21622904>.

59. Dorrell, R.G., et al., *Evolution of chloroplast transcript processing in Plasmodium and its chromerid algal relatives.* PLoS Genet, 2014. **10**(1): p. e1004008. <http://www.ncbi.nlm.nih.gov/pubmed/24453981>.

60. Dorrell, R.G. and C.J. Howe, *Integration of plastids with their hosts: Lessons learned from dinoflagellates.* Proc Natl Acad Sci U S A, 2015. **112**(33): p. 10247-54. <http://www.ncbi.nlm.nih.gov/pubmed/25995366>.

61. Chan, C.X., et al., *Red and green algal monophyly and extensive gene sharing found in a rich repertoire of red algal genes.* Curr Biol, 2011. **21**(4): p. 328-33. <http://www.ncbi.nlm.nih.gov/pubmed/21315598>.

62. Yoon, H.S., et al., *Tertiary endosymbiosis driven genome evolution in dinoflagellate algae.* Mol Biol Evol, 2005. **22**(5): p. 1299-308. <http://www.ncbi.nlm.nih.gov/pubmed/15746017>.

63. Hayakawa, S., et al., *Function and evolutionary origin of unicellular camera-type eye structure.* PLoS One, 2015. **10**(3): p. e0118415. <http://www.ncbi.nlm.nih.gov/pubmed/25734540>.

64. Adams, K.L., et al., *Punctuated evolution of mitochondrial gene content: high and variable rates of mitochondrial gene loss and transfer to the nucleus during angiosperm evolution.* Proc Natl Acad Sci U S A, 2002. **99**(15): p. 9905-12. <http://www.ncbi.nlm.nih.gov/pubmed/12119382>.

65. Adams, K.L., et al., *Repeated, recent and diverse transfers of a mitochondrial gene to the nucleus in flowering plants.* Nature, 2000. **408**(6810): p. 354-7. <http://www.ncbi.nlm.nih.gov/pubmed/11099041>.

66. Adams, K.L. and J.D. Palmer, *Evolution of mitochondrial gene content: gene loss and transfer to the nucleus.* Mol Phylogenet Evol, 2003. **29**(3): p. 380-95. <http://www.ncbi.nlm.nih.gov/pubmed/14615181>.

67. Gandini, C.L. and M.V. Sanchez-Puerta, *Foreign Plastid Sequences in Plant Mitochondria are Frequently Acquired Via Mitochondrion-to-Mitochondrion Horizontal Transfer.* Sci Rep, 2017. **7**: p. 43402. <http://www.ncbi.nlm.nih.gov/pubmed/28262720>.

68. Lang, B.F., et al., *An ancestral mitochondrial DNA resembling a eubacterial genome in miniature.* Nature, 1997. **387**(6632): p. 493-7. <http://www.ncbi.nlm.nih.gov/pubmed/9168110>.

69. Gray, M.W., et al., *Genome structure and gene content in protist mitochondrial DNAs.* Nucleic Acids Res, 1998. **26**(4): p. 865-78. <http://www.ncbi.nlm.nih.gov/pubmed/9461442>.

70. Boussau, B., et al., *Computational inference of scenarios for alpha-proteobacterial genome evolution.* Proc Natl Acad Sci U S A, 2004. **101**(26): p. 9722-7. <http://www.ncbi.nlm.nih.gov/pubmed/15210995>.

71. Howe, C.J., et al., *The origin of plastids.* Philos Trans R Soc Lond B Biol Sci, 2008. **363**(1504): p. 2675-85. <http://www.ncbi.nlm.nih.gov/pubmed/18468982>.

72. Bhattacharya, D., et al., *Single cell genome analysis supports a link between phagotrophy and primary plastid endosymbiosis.* Sci Rep, 2012. **2**: p. 356. <http://www.ncbi.nlm.nih.gov/pubmed/22493757>.

73. Bucher, M., et al., *Development and Symbiosis Establishment in the Cnidarian Endosymbiosis Model Aiptasia sp.* Sci Rep, 2016. **6**: p. 19867. <http://www.ncbi.nlm.nih.gov/pubmed/26804034>.

74. Hoogenboom, M.O., et al., *Effects of light, food availability and temperature stress on the function of photosystem II and photosystem I of coral symbionts.* PLoS One, 2012. **7**(1): p. e30167. <http://www.ncbi.nlm.nih.gov/pubmed/22253915>.

75. Stefano, G.B., C. Snyder, and R.M. Kream, *Mitochondria, Chloroplasts in Animal and Plant Cells: Significance of Conformational Matching.* Med Sci Monit, 2015. **21**: p. 2073-8. <http://www.ncbi.nlm.nih.gov/pubmed/26184462>.

76. Qiu, H., H.S. Yoon, and D. Bhattacharya, *Algal endosymbionts as vectors of horizontal gene transfer in photosynthetic eukaryotes.* Front Plant Sci, 2013. **4**: p. 366. <http://www.ncbi.nlm.nih.gov/pubmed/24065973>.

77. Dearnaley, J.D., *Further advances in orchid mycorrhizal research.* Mycorrhiza, 2007. **17**(6): p. 475-86. <http://www.ncbi.nlm.nih.gov/pubmed/17582535>.

78. Bonnardeaux, Y., et al., *Diversity of mycorrhizal fungi of terrestrial orchids: compatibility webs, brief encounters, lasting relationships and alien invasions.* Mycol Res, 2007. **111**(Pt 1): p. 51-61. <http://www.ncbi.nlm.nih.gov/pubmed/17289365>.

79. Cameron, D.D., J.R. Leake, and D.J. Read, *Mutualistic mycorrhiza in orchids: evidence from plant-fungus carbon and nitrogen transfers in the green-leaved terrestrial orchid Goodyera repens.* New Phytol, 2006. **171**(2): p. 405-16. <http://www.ncbi.nlm.nih.gov/pubmed/16866946>.

80. Bucher, M., S. Wegmuller, and D. Drissner, *Chasing the structures of small molecules in arbuscular mycorrhizal signaling.* Curr Opin Plant Biol, 2009. **12**(4): p. 500-7. <http://www.ncbi.nlm.nih.gov/pubmed/19576840>.

81. Neish, A.S., *Microbes in gastrointestinal health and disease.* Gastroenterology, 2009. **136**(1): p. 65-80. <http://www.ncbi.nlm.nih.gov/pubmed/19026645>.

82. Hoffmeister, M. and W. Martin, *Interspecific evolution: microbial symbiosis, endosymbiosis and gene transfer.* Environ Microbiol, 2003. **5**(8): p. 641-9. <http://www.ncbi.nlm.nih.gov/pubmed/12871231>.

83. Ohkuma, M., *Termite symbiotic systems: efficient bio-recycling of lignocellulose.* Appl Microbiol Biotechnol, 2003. **61**(1): p. 1-9. <http://www.ncbi.nlm.nih.gov/pubmed/12658509>.

84. Venkatesh, M., et al., *Symbiotic Bacterial Metabolites Regulate Gastrointestinal Barrier Function via the Xenobiotic Sensor PXR and Toll-like Receptor 4.* Immunity, 2014. <http://www.ncbi.nlm.nih.gov/pubmed/25065623>.

85. Li, M., et al., *Symbiotic gut microbes modulate human metabolic phenotypes.* Proc Natl Acad Sci U S A\, 2008. **105\**(6\): p. 2117-22\. <http://www.ncbi.nlm.nih.gov/pubmed/18252821>\.

86. Fast, E.M., et al., *Wolbachia Enhance Drosophila Stem Cell Proliferation and Target the Germline Stem Cell Niche.* Science, 2011. <http://www.ncbi.nlm.nih.gov/pubmed/22021671>.

87. Liang, Y., et al., *Nonlegumes respond to rhizobial Nod factors by suppressing the innate immune response.* Science, 2013. **341**(6152): p. 1384-7. <http://www.ncbi.nlm.nih.gov/pubmed/24009356>.

88. Zook, D., *Symbiosis—Evolution’s Co-Author*, in *Reticulate Evolution, Interdisciplinary Evolution Research 3*, N. Gontier, Editor 2015, Soringer: Heidelberg. .

89. Frugier, F., et al., *Cytokinin: secret agent of symbiosis.* Trends Plant Sci\, 2008. **13\**(3\): p. 115-20\. <http://www.ncbi.nlm.nih.gov/pubmed/18296104>\.

90. Crespi, M. and F. Frugier, *De novo organ formation from differentiated cells: root nodule organogenesis.* Sci Signal, 2008. **1**(49): p. re11. <http://www.ncbi.nlm.nih.gov/pubmed/19066400>.

91. Walker, T., et al., *The wMel Wolbachia strain blocks dengue and invades caged Aedes aegypti populations.* Nature, 2011. **476**(7361): p. 450-3. <http://www.ncbi.nlm.nih.gov/pubmed/21866159>.

92. Kambris, Z., et al., *Wolbachia stimulates immune gene expression and inhibits plasmodium development in Anopheles gambiae.* PLoS Pathog, 2010. **6**(10). <http://www.ncbi.nlm.nih.gov/pubmed/20949079>.

93. Frentiu, F.D., et al., *Wolbachia-mediated resistance to dengue virus infection and death at the cellular level.* PLoS One, 2010. **5**(10): p. e13398. <http://www.ncbi.nlm.nih.gov/pubmed/20976219>.

94. Mousson, L., et al., *Wolbachia modulates Chikungunya replication in Aedes albopictus.* Mol Ecol, 2010. <http://www.ncbi.nlm.nih.gov/pubmed/20345686>.

95. Osborne, S.E., et al., *Variation in antiviral protection mediated by different Wolbachia strains in Drosophila simulans.* PLoS Pathog, 2009. **5**(11): p. e1000656. <http://www.ncbi.nlm.nih.gov/pubmed/19911047>.

96. Teixeira, L., A. Ferreira, and M. Ashburner, *The bacterial symbiont Wolbachia induces resistance to RNA viral infections in Drosophila melanogaster.* PLoS Biol, 2008. **6**(12): p. e2. <http://www.ncbi.nlm.nih.gov/pubmed/19222304>.

97. Pfeiffer, J.K. and J.L. Sonnenburg, *The intestinal microbiota and viral susceptibility.* Front Microbiol, 2011. **2**: p. 92. <http://www.ncbi.nlm.nih.gov/pubmed/21833331>.

98. Kuss, S.K., et al., *Intestinal microbiota promote enteric virus replication and systemic pathogenesis.* Science, 2011. **334**(6053): p. 249-52. <http://www.ncbi.nlm.nih.gov/pubmed/21998395>.

99. Cash, H.L., et al., *Symbiotic bacteria direct expression of an intestinal bactericidal lectin.* Science, 2006. **313**(5790): p. 1126-30. <http://www.ncbi.nlm.nih.gov/pubmed/16931762>.

100. Dash, S., et al., *The gut microbiome and diet in psychiatry: focus on depression.* Curr Opin Psychiatry, 2015. **28**(1): p. 1-6. <http://www.ncbi.nlm.nih.gov/pubmed/25415497>.

101. Cryan, J.F. and S.M. O'Mahony, *The microbiome-gut-brain axis: from bowel to behavior.* Neurogastroenterol Motil, 2011. **23**(3): p. 187-92. <http://www.ncbi.nlm.nih.gov/pubmed/21303428>.

102. Masson-Boivin, C., et al., *Establishing nitrogen-fixing symbiosis with legumes: how many rhizobium recipes?* Trends Microbiol, 2009. **17**(10): p. 458-66. <http://www.ncbi.nlm.nih.gov/pubmed/19766492>.

103. Marchetti, M., et al., *Experimental evolution of a plant pathogen into a legume symbiont.* PLoS Biol, 2010. **8**(1): p. e1000280. <http://www.ncbi.nlm.nih.gov/pubmed/20084095>.

104. Guan, S.H., et al., *Experimental evolution of nodule intracellular infection in legume symbionts.* Isme J, 2013. **7**(7): p. 1367-77. <http://www.ncbi.nlm.nih.gov/pubmed/23426010>.

105. Marchetti, M., et al., *Shaping bacterial symbiosis with legumes by experimental evolution.* Mol Plant Microbe Interact, 2014. **27**(9): p. 956-64. <http://www.ncbi.nlm.nih.gov/pubmed/25105803>.

106. Remigi, P., et al., *Transient Hypermutagenesis Accelerates the Evolution of Legume Endosymbionts following Horizontal Gene Transfer.* PLoS Biol, 2014. **12**(9): p. e1001942. <http://www.ncbi.nlm.nih.gov/pubmed/25181317>.

107. Cairney, J.W., *Evolution of mycorrhiza systems.* Naturwissenschaften, 2000. **87**(11): p. 467-75. <http://www.ncbi.nlm.nih.gov/pubmed/11151665>.

108. Finlay, R.D., *Ecological aspects of mycorrhizal symbiosis: with special emphasis on the functional diversity of interactions involving the extraradical mycelium.* J Exp Bot, 2008. **59**(5): p. 1115-26. <http://www.ncbi.nlm.nih.gov/pubmed/18349054>.

109. Willing, B.P., S.L. Russell, and B.B. Finlay, *Shifting the balance: antibiotic effects on host-microbiota mutualism.* Nat Rev Microbiol, 2011. **9**(4): p. 233-43. <http://www.ncbi.nlm.nih.gov/pubmed/21358670>.

110. Bonfante, P. and N. Requena, *Dating in the dark: how roots respond to fungal signals to establish arbuscular mycorrhizal symbiosis.* Curr Opin Plant Biol, 2011. **14**(4): p. 451-7. <http://www.ncbi.nlm.nih.gov/pubmed/21489861>.

111. Navazio, L., et al., *A diffusible signal from arbuscular mycorrhizal fungi elicits a transient cytosolic calcium elevation in host plant cells.* Plant Physiol, 2007. **144**(2): p. 673-81. <http://www.ncbi.nlm.nih.gov/pubmed/17142489>.

112. Salvioli, A., et al., *Endobacteria affect the metabolic profile of their host Gigaspora margarita, an arbuscular mycorrhizal fungus.* Environ Microbiol, 2010. <http://www.ncbi.nlm.nih.gov/pubmed/20545745>.

113. Tisserant, E., et al., *Genome of an arbuscular mycorrhizal fungus provides insight into the oldest plant symbiosis.* Proc Natl Acad Sci U S A, 2013. <http://www.ncbi.nlm.nih.gov/pubmed/24277808>.

114. Ghignone, S., et al., *The genome of the obligate endobacterium of an AM fungus reveals an interphylum network of nutritional interactions.* Isme J, 2011. <http://www.ncbi.nlm.nih.gov/pubmed/21866182>.

115. Ghignone, S., et al., *The genome of the obligate endobacterium of an AM fungus reveals an interphylum network of nutritional interactions.* Isme J, 2012. **6**(1): p. 136-45. <http://www.ncbi.nlm.nih.gov/pubmed/21866182>.

116. Torres-Cortes, G., et al., *Mosaic genome of endobacteria in arbuscular mycorrhizal fungi: Transkingdom gene transfer in an ancient mycoplasma-fungus association.* Proc Natl Acad Sci U S A, 2015. **112**(112): p. 7785-90. <http://www.ncbi.nlm.nih.gov/pubmed/25964335>.

117. Martin, F., et al., *Perigord black truffle genome uncovers evolutionary origins and mechanisms of symbiosis.* Nature, 2010. <http://www.ncbi.nlm.nih.gov/pubmed/20348908>.

118. Bonfante, P., *Plants, mycorrhizal fungi and endobacteria: a dialog among cells and genomes.* Biol Bull, 2003. **204**(2): p. 215-20. <http://www.ncbi.nlm.nih.gov/pubmed/12700157>.

119. Bonfante, P. and I.A. Anca, *Plants, mycorrhizal fungi, and bacteria: a network of interactions.* Annu Rev Microbiol, 2009. **63**: p. 363-83. <http://www.ncbi.nlm.nih.gov/pubmed/19514845>.

120. Ohkuma, M., et al., *Acetogenesis from H2 plus CO2 and nitrogen fixation by an endosymbiotic spirochete of a termite-gut cellulolytic protist.* Proc Natl Acad Sci U S A, 2015. <http://www.ncbi.nlm.nih.gov/pubmed/25979941>.

121. Noda, S., et al., *Complex coevolutionary history of symbiotic Bacteroidales bacteria of various protists in the gut of termites.* BMC Evol Biol, 2009. **9**: p. 158. <http://www.ncbi.nlm.nih.gov/pubmed/19586555>.

122. Hongoh, Y., et al., *The motility symbiont of the termite gut flagellate Caduceia versatilis is a member of the "Synergistes" group.* Appl Environ Microbiol, 2007. **73**(19): p. 6270-6. <http://www.ncbi.nlm.nih.gov/pubmed/17675420>.

123. Ohkuma, M., *Symbioses of flagellates and prokaryotes in the gut of lower termites.* Trends Microbiol, 2008. **16**(7): p. 345-52. <http://www.ncbi.nlm.nih.gov/pubmed/18513972>.

124. Kudo, T., *Termite-microbe symbiotic system and its efficient degradation of lignocellulose.* Biosci Biotechnol Biochem, 2009. **73**(12): p. 2561-7. <http://www.ncbi.nlm.nih.gov/pubmed/19966490>.

125. Balmand, S., et al., *Tissue distribution and transmission routes for the tsetse fly endosymbionts.* J Invertebr Pathol, 2013. **112 Suppl**: p. S116-22. <http://www.ncbi.nlm.nih.gov/pubmed/22537833>.

126. Fenn, K., et al., *Phylogenetic relationships of the Wolbachia of nematodes and arthropods.* PLoS Pathog\, 2006. **2\**(10\): p. e94\. <http://www.ncbi.nlm.nih.gov/pubmed/17040125>\.

127. Zouache, K., et al., *Persistent Wolbachia and cultivable bacteria infection in the reproductive and somatic tissues of the mosquito vector Aedes albopictus.* PLoS One, 2009. **4**(7): p. e6388. <http://www.ncbi.nlm.nih.gov/pubmed/19633721>.

128. Raychoudhury, R., et al., *Modes of acquisition of Wolbachia: horizontal transfer, hybrid introgression, and codivergence in the Nasonia species complex.* Evolution, 2009. **63**(1): p. 165-83. <http://www.ncbi.nlm.nih.gov/pubmed/18826448>.

129. Hughes, G.L., et al., *Invasion of Wolbachia into Anopheles and other insect germlines in an ex vivo organ culture system.* PLoS One, 2012. **7**(4): p. e36277. <http://www.ncbi.nlm.nih.gov/pubmed/22558418>.

130. Narita, S., et al., *Unexpected mechanism of symbiont-induced reversal of insect sex: feminizing Wolbachia continuously acts on the butterfly Eurema hecabe during larval development.* Appl Environ Microbiol, 2007. **73**(13): p. 4332-41. <http://www.ncbi.nlm.nih.gov/pubmed/17496135>.

131. Gazla, I.N. and M.C. Carracedo, *Effect of intracellular Wolbachia on interspecific crosses between Drosophila melanogaster and Drosophila simulans.* Genet Mol Res, 2009. **8**(3): p. 861-9. <http://www.ncbi.nlm.nih.gov/pubmed/19731208>.

132. Landmann, F., et al., *Wolbachia-mediated cytoplasmic incompatibility is associated with impaired histone deposition in the male pronucleus.* PLoS Pathog, 2009. **5**(3): p. e1000343. <http://www.ncbi.nlm.nih.gov/pubmed/19300496>.

133. Kutschera, U. and K.J. Niklas, *Endosymbiosis, cell evolution, and speciation.* Theory Biosci, 2005. **124**(1): p. 1-24. <http://www.ncbi.nlm.nih.gov/pubmed/17046345>.

134. Taylor, F.J., *Symbionticism revisited: a discussion of the evolutionary impact of intracellular symbioses.* Proc R Soc Lond B Biol Sci, 1979. **204**(1155): p. 267-86. <http://www.ncbi.nlm.nih.gov/pubmed/36627>.

135. Brucker, R.M. and S.R. Bordenstein, *Speciation by symbiosis.* Trends Ecol Evol, 2012. **27**(8): p. 443-51. <http://www.ncbi.nlm.nih.gov/pubmed/22541872>.

136. Round, J.L., R.M. O'Connell, and S.K. Mazmanian, *Coordination of tolerogenic immune responses by the commensal microbiota.* J Autoimmun, 2010. **34**(3): p. J220-5. <http://www.ncbi.nlm.nih.gov/pubmed/19963349>.

137. Petersen, C. and J.L. Round, *Defining dysbiosis and its influence on host immunity and disease.* Cell Microbiol, 2014. **16**(7): p. 1024-33. <http://www.ncbi.nlm.nih.gov/pubmed/24798552>.

138. Round, J.L. and S.K. Mazmanian, *The gut microbiota shapes intestinal immune responses during health and disease.* Nat Rev Immunol, 2009. **9**(5): p. 313-23. <http://www.ncbi.nlm.nih.gov/pubmed/19343057>.

139. Round, J.L. and S.K. Mazmanian, *Inducible Foxp3+ regulatory T-cell development by a commensal bacterium of the intestinal microbiota.* Proc Natl Acad Sci U S A, 2010. **107**(27): p. 12204-9. <http://www.ncbi.nlm.nih.gov/pubmed/20566854>.

140. Molloy, M.J., N. Bouladoux, and Y. Belkaid, *Intestinal microbiota: shaping local and systemic immune responses.* Semin Immunol, 2012. **24**(1): p. 58-66. <http://www.ncbi.nlm.nih.gov/pubmed/22178452>.

141. Kaplan, J.L., H. Ning Shi, and W.A. Walker, *The Role of Microbes in Developmental Immunologic Programming.* Pediatr Res, 2011. <http://www.ncbi.nlm.nih.gov/pubmed/21364495>.

142. Chrostek, E., et al., *Wolbachia Variants Induce Differential Protection to Viruses in Drosophila melanogaster: A Phenotypic and Phylogenomic Analysis.* PLoS Genet, 2013. **9**(12): p. e1003896. <http://www.ncbi.nlm.nih.gov/pubmed/24348259>.

143. Borre, Y.E., et al., *The impact of microbiota on brain and behavior: mechanisms & therapeutic potential.* Adv Exp Med Biol, 2014. **817**: p. 373-403. <http://www.ncbi.nlm.nih.gov/pubmed/24997043>.

144. Bravo, J.A., et al., *Ingestion of Lactobacillus strain regulates emotional behavior and central GABA receptor expression in a mouse via the vagus nerve.* Proc Natl Acad Sci U S A, 2011. <http://www.ncbi.nlm.nih.gov/pubmed/21876150>.

145. Dinan, T.G. and J.F. Cryan, *Melancholic microbes: a link between gut microbiota and depression?* Neurogastroenterol Motil, 2013. **25**(9): p. 713-9. <http://www.ncbi.nlm.nih.gov/pubmed/23910373>.

146. Cryan, J.F. and T.G. Dinan, *Mind-altering microorganisms: the impact of the gut microbiota on brain and behaviour.* Nat Rev Neurosci, 2012. **13**(10): p. 701-12. <http://www.ncbi.nlm.nih.gov/pubmed/22968153>.

147. O'Mahony, S.M., et al., *Serotonin, tryptophan metabolism and the brain-gut-microbiome axis.* Behav Brain Res, 2015. **277**: p. 32-48. <http://www.ncbi.nlm.nih.gov/pubmed/25078296>.

148. Shin, S.C., et al., *Drosophila microbiome modulates host developmental and metabolic homeostasis via insulin signaling.* Science, 2011. **334**(6056): p. 670-4. <http://www.ncbi.nlm.nih.gov/pubmed/22053049>.

149. Hongoh, Y., *Diversity and genomes of uncultured microbial symbionts in the termite gut.* Biosci Biotechnol Biochem, 2010. **74**(6): p. 1145-51. <http://www.ncbi.nlm.nih.gov/pubmed/20530908>.

150. Aanen, D.K., et al., *High symbiont relatedness stabilizes mutualistic cooperation in fungus-growing termites.* Science, 2009. **326**(5956): p. 1103-6. <http://www.ncbi.nlm.nih.gov/pubmed/19965427>.

151. Serbus, L.R. and W. Sullivan, *A cellular basis for Wolbachia recruitment to the host germline.* PLoS Pathog, 2007. **3**(12): p. e190. <http://www.ncbi.nlm.nih.gov/pubmed/18085821>.

152. Aksoy, S., X. Chen, and V. Hypsa, *Phylogeny and potential transmission routes of midgut-associated endosymbionts of tsetse (Diptera:Glossinidae).* Insect Mol Biol, 1997. **6**(2): p. 183-90. <http://www.ncbi.nlm.nih.gov/pubmed/9099582>.

153. Koga, R., et al., *Cellular mechanism for selective vertical transmission of an obligate insect symbiont at the bacteriocyte-embryo interface.* Proc Natl Acad Sci U S A, 2012. **109**(20): p. E1230-7. <http://www.ncbi.nlm.nih.gov/pubmed/22517738>.

154. Webster, N.S., et al., *Deep sequencing reveals exceptional diversity and modes of transmission for bacterial sponge symbionts.* Environ Microbiol, 2010. **12**(8): p. 2070-82. <http://www.ncbi.nlm.nih.gov/pubmed/21966903>.

155. Bright, M. and S. Bulgheresi, *A complex journey: transmission of microbial symbionts.* Nat Rev Microbiol, 2010. **8**(3): p. 218-30. <http://www.ncbi.nlm.nih.gov/pubmed/20157340>.

156. Schmitt, S., et al., *Vertical transmission of a phylogenetically complex microbial consortium in the viviparous sponge Ircinia felix.* Appl Environ Microbiol, 2007. **73**(7): p. 2067-78. <http://www.ncbi.nlm.nih.gov/pubmed/17277226>.

157. Schneider, D., et al., *Phylogenetic analysis of a microbialite-forming microbial mat from a hypersaline lake of the Kiritimati atoll, Central Pacific.* PLoS One, 2013. **8**(6): p. e66662. <http://www.ncbi.nlm.nih.gov/pubmed/23762495>.

158. Baumgartner, L.K., et al., *Microbial diversity in modern marine stromatolites, Highborne Cay, Bahamas.* Environ Microbiol, 2009. **11**(10): p. 2710-9. <http://www.ncbi.nlm.nih.gov/pubmed/19601956>.

159. Papineau, D., et al., *Composition and structure of microbial communities from stromatolites of Hamelin Pool in Shark Bay, Western Australia.* Appl Environ Microbiol, 2005. **71**(8): p. 4822-32. <http://www.ncbi.nlm.nih.gov/pubmed/16085880>.

160. Reid, R.P., et al., *The role of microbes in accretion, lamination and early lithification of modern marine stromatolites.* Nature, 2000. **406**(6799): p. 989-92. <http://www.ncbi.nlm.nih.gov/pubmed/10984051>.

161. Robinson-Rechavi, M., B. Boussau, and V. Laudet, *Phylogenetic dating and characterization of gene duplications in vertebrates: the cartilaginous fish reference.* Mol Biol Evol, 2004. **21**(3): p. 580-6. <http://www.ncbi.nlm.nih.gov/pubmed/14694077>.

162. Hulsey, C.D. and F.J. Garcia-de-Leon, *Introgressive hybridization in a trophically polymorphic cichlid.* Ecol Evol, 2013. **3**(13): p. 4536-47. <http://www.ncbi.nlm.nih.gov/pubmed/24340193>.

163. Magalhaes, I.S., et al., *Untangling the evolutionary history of a highly polymorphic species: introgressive hybridization and high genetic structure in the desert cichlid fish Herichtys minckleyi.* Mol Ecol, 2015. **24**(17): p. 4505-20. <http://www.ncbi.nlm.nih.gov/pubmed/26175313>.

164. Keller, I., et al., *Population genomic signatures of divergent adaptation, gene flow and hybrid speciation in the rapid radiation of Lake Victoria cichlid fishes.* Mol Ecol, 2013. **22**(11): p. 2848-63. <http://www.ncbi.nlm.nih.gov/pubmed/23121191>.

165. Smith, P.F., A. Konings, and I. Kornfield, *Hybrid origin of a cichlid population in Lake Malawi: implications for genetic variation and species diversity.* Mol Ecol, 2003. **12**(9): p. 2497-504. <http://www.ncbi.nlm.nih.gov/pubmed/12919487>.

166. Joyce, D.A., et al., *Repeated colonization and hybridization in Lake Malawi cichlids.* Curr Biol, 2011. **21**(3): p. R108-9. <http://www.ncbi.nlm.nih.gov/pubmed/21300271>.

167. Loh, Y.H., et al., *Origins of shared genetic variation in African cichlids.* Mol Biol Evol, 2013. **30**(4): p. 906-17. <http://www.ncbi.nlm.nih.gov/pubmed/23275489>.

168. Seehausen, O., *Process and pattern in cichlid radiations - inferences for understanding unusually high rates of evolutionary diversification.* New Phytol, 2015. **207**(2): p. 304-12. <http://www.ncbi.nlm.nih.gov/pubmed/25983053>.

169. Seehausen, O., *African cichlid fish: a model system in adaptive radiation research.* Proc Biol Sci, 2006. **273**(1597): p. 1987-98. <http://www.ncbi.nlm.nih.gov/pubmed/16846905>.

170. Syvanen, M. and C.I. Kado, *Horizontal Gene Transfer 2nd Ed*2002, London: Academic Press. .

171. Syvanen, M., *Cross-species gene transfer; implications for a new theory of evolution.* J Theor Biol, 1985. **112**(2): p. 333-43. <http://www.ncbi.nlm.nih.gov/pubmed/2984477>.

172. Katz, L.A., *Recent events dominate interdomain lateral gene transfers between prokaryotes and eukaryotes and, with the exception of endosymbiotic gene transfers, few ancient transfer events persist.* Philos Trans R Soc Lond B Biol Sci, 2015. **370**(1678): p. 20140324. <http://www.ncbi.nlm.nih.gov/pubmed/26323756>.

173. Benveniste, R. and J. Davies, *Mechanisms of antibiotic resistance in bacteria.* Annu Rev Biochem, 1973. **42**: p. 471-506. <http://www.ncbi.nlm.nih.gov/pubmed/4581231>.

174. Watanabe, T., *Selected Methods of Genetic Study of Episome-Mediated Drug Resistance in Bacteria.* Methods Med Res, 1964. **10**: p. 202-20. <http://www.ncbi.nlm.nih.gov/pubmed/14284923>.

175. Watanabe, T., *Episome-Mediated Transfer of Drug Resistance in Enterobacteriaceae. Vi. High-Frequency Resistance Transfer System in Escherichia Coli.* J Bacteriol, 1963. **85**: p. 788-94. <http://www.ncbi.nlm.nih.gov/pubmed/14044944>.

176. Svara, F. and D.J. Rankin, *The evolution of plasmid-carried antibiotic resistance.* BMC Evol Biol, 2011. **11**(1): p. 130. <http://www.ncbi.nlm.nih.gov/pubmed/21595903>.

177. Domingues, S., K.M. Nielsen, and G.J. da Silva, *Various pathways leading to the acquisition of antibiotic resistance by natural transformation.* Mob Genet Elements, 2012. **2**(6): p. 257-260. <http://www.ncbi.nlm.nih.gov/pubmed/23482877>.

178. Stalder, T., et al., *Integron involvement in environmental spread of antibiotic resistance.* Front Microbiol, 2012. **3**: p. 119. <http://www.ncbi.nlm.nih.gov/pubmed/22509175>.

179. Norman, A., L.H. Hansen, and S.J. Sorensen, *Conjugative plasmids: vessels of the communal gene pool.* Philos Trans R Soc Lond B Biol Sci, 2009. **364**(1527): p. 2275-89. <http://www.ncbi.nlm.nih.gov/pubmed/19571247>.

180. Faguy, D.M. and W.F. Doolittle, *Horizontal transfer of catalase-peroxidase genes between archaea and pathogenic bacteria.* Trends Genet, 2000. **16**(5): p. 196-7. <http://www.ncbi.nlm.nih.gov/pubmed/10782109>.

181. Nesbo, C.L., et al., *Phylogenetic analyses of two "archaeal" genes in thermotoga maritima reveal multiple transfers between archaea and bacteria.* Mol Biol Evol, 2001. **18**(3): p. 362-75. <http://www.ncbi.nlm.nih.gov/pubmed/11230537>.

182. Chen, J., et al., *Pathogenicity island-directed transfer of unlinked chromosomal virulence genes.* Mol Cell, 2015. **57**(1): p. 138-49. <http://www.ncbi.nlm.nih.gov/pubmed/25498143>.

183. Maiques, E., et al., *Beta-lactam antibiotics induce the SOS response and horizontal transfer of virulence factors in Staphylococcus aureus.* J Bacteriol, 2006. **188**(7): p. 2726-9. <http://www.ncbi.nlm.nih.gov/pubmed/16547063>.

184. Saisongkorh, W., et al., *Evidence of transfer by conjugation of type IV secretion system genes between Bartonella species and Rhizobium radiobacter in amoeba.* PLoS One, 2010. **5**(9): p. e12666. <http://www.ncbi.nlm.nih.gov/pubmed/20856925>.

185. Venner, S., et al., *Ecological networks to unravel the routes to horizontal transposon transfers.* PLoS Biol, 2017. **15**(2): p. e2001536. <http://www.ncbi.nlm.nih.gov/pubmed/28199335>.

186. Guglielmini, J., et al., *The Repertoire of ICE in Prokaryotes Underscores the Unity, Diversity, and Ubiquity of Conjugation.* PLoS Genet, 2011. **7**(8): p. e1002222. <http://www.ncbi.nlm.nih.gov/pubmed/21876676>.

187. Burrus, V., J. Marrero, and M.K. Waldor, *The current ICE age: biology and evolution of SXT-related integrating conjugative elements.* Plasmid, 2006. **55**(3): p. 173-83. <http://www.ncbi.nlm.nih.gov/pubmed/16530834>.

188. Wozniak, R.A., et al., *Comparative ICE genomics: insights into the evolution of the SXT/R391 family of ICEs.* PLoS Genet, 2009. **5**(12): p. e1000786. <http://www.ncbi.nlm.nih.gov/pubmed/20041216>.

189. Burrus, V. and M.K. Waldor, *Shaping bacterial genomes with integrative and conjugative elements.* Res Microbiol, 2004. **155**(5): p. 376-86. <http://www.ncbi.nlm.nih.gov/pubmed/15207870>.

190. Burrus, V., et al., *Conjugative transposons: the tip of the iceberg.* Mol Microbiol, 2002. **46**(3): p. 601-10. <http://www.ncbi.nlm.nih.gov/pubmed/12410819>.

191. Lautner, M., et al., *Regulation, integrase-dependent excision, and horizontal transfer of genomic islands in Legionella pneumophila.* J Bacteriol, 2013. **195**(7): p. 1583-97. <http://www.ncbi.nlm.nih.gov/pubmed/23354744>.

192. Gal-Mor, O. and B.B. Finlay, *Pathogenicity islands: a molecular toolbox for bacterial virulence.* Cell Microbiol, 2006. **8**(11): p. 1707-19. <http://www.ncbi.nlm.nih.gov/pubmed/16939533>.

193. Kers, J.A., et al., *A large, mobile pathogenicity island confers plant pathogenicity on Streptomyces species.* Mol Microbiol, 2005. **55**(4): p. 1025-33. <http://www.ncbi.nlm.nih.gov/pubmed/15686551>.

194. Haggerty, L.S., et al., *A pluralistic account of homology: adapting the models to the data.* Mol Biol Evol, 2014. **31**(3): p. 501-16. <http://www.ncbi.nlm.nih.gov/pubmed/24273322>.

195. Fournier, G.P., C.P. Andam, and J.P. Gogarten, *Ancient horizontal gene transfer and the last common ancestors.* BMC Evol Biol, 2015. **15**: p. 70. <http://www.ncbi.nlm.nih.gov/pubmed/25897759>.

196. Filee, J., *Lateral gene transfer, lineage-specific gene expansion and the evolution of Nucleo Cytoplasmic Large DNA viruses.* J Invertebr Pathol, 2009. **101**(3): p. 169-71. <http://www.ncbi.nlm.nih.gov/pubmed/19457437>.

197. Filee, J. and M. Chandler, *Gene exchange and the origin of giant viruses.* Intervirology, 2010. **53**(5): p. 354-61. <http://www.ncbi.nlm.nih.gov/pubmed/20551687>.

198. Filee, J., N. Pouget, and M. Chandler, *Phylogenetic evidence for extensive lateral acquisition of cellular genes by Nucleocytoplasmic large DNA viruses.* BMC Evol Biol, 2008. **8**: p. 320. <http://www.ncbi.nlm.nih.gov/pubmed/19036122>.

199. Boyer, M., et al., *Giant Marseillevirus highlights the role of amoebae as a melting pot in emergence of chimeric microorganisms.* Proc Natl Acad Sci U S A, 2009. **106**(51): p. 21848-53. <http://www.ncbi.nlm.nih.gov/pubmed/20007369>.

200. Forslund, K. and E.L. Sonnhammer, *Evolution of protein domain architectures.* Methods Mol Biol, 2012. **856**: p. 187-216. <http://www.ncbi.nlm.nih.gov/pubmed/22399460>.

201. Doolittle, R.F. and P. Bork, *Evolutionarily mobile modules in proteins.* Sci Am, 1993. **269**(4): p. 50-6. <http://www.ncbi.nlm.nih.gov/pubmed/8235550>.

202. Itzhaki, Z. and H. Margalit, *Reduced polymorphism in domains involved in protein-protein interactions.* PLoS One, 2012. **7**(4): p. e34503. <http://www.ncbi.nlm.nih.gov/pubmed/22509312>.

203. Kanaan, S.P., et al., *Inferring protein-protein interactions from multiple protein domain combinations.* Methods Mol Biol, 2009. **541**: p. 43-59. <http://www.ncbi.nlm.nih.gov/pubmed/19381530>.

204. Bondos, S.E. and X.X. Tan, *Combinatorial transcriptional regulation: the interaction of transcription factors and cell signaling molecules with homeodomain proteins in Drosophila development.* Crit Rev Eukaryot Gene Expr, 2001. **11**(1-3): p. 145-71. <http://www.ncbi.nlm.nih.gov/pubmed/11693959>.

205. Sanselicio, S. and P.H. Viollier, *Convergence of alarmone and cell cycle signaling from trans-encoded sensory domains.* MBio, 2015. **6**(5): p. e01415-15. <http://www.ncbi.nlm.nih.gov/pubmed/26489861>.

206. Wuchty, S. and E. Almaas, *Evolutionary cores of domain co-occurrence networks.* BMC Evol Biol, 2005. **5**(1): p. 24. <http://www.ncbi.nlm.nih.gov/pubmed/15788102>.

207. Weiner, J., 3rd, A.D. Moore, and E. Bornberg-Bauer, *Just how versatile are domains?* BMC Evol Biol, 2008. **8**: p. 285. <http://www.ncbi.nlm.nih.gov/pubmed/18854028>.

208. Kummerfeld, S.K. and S.A. Teichmann, *Protein domain organisation: adding order.* BMC Bioinformatics, 2009. **10**: p. 39. <http://www.ncbi.nlm.nih.gov/pubmed/19178743>.

209. Bornberg-Bauer, E., et al., *The evolution of domain arrangements in proteins and interaction networks.* Cell Mol Life Sci, 2005. **62**(4): p. 435-45. <http://www.ncbi.nlm.nih.gov/pubmed/15719170>.

210. Wang, Z., et al., *A protein domain co-occurrence network approach for predicting protein function and inferring species phylogeny.* PLoS One, 2011. **6**(3): p. e17906. <http://www.ncbi.nlm.nih.gov/pubmed/21455299>.

211. Buljan, M., A. Frankish, and A. Bateman, *Quantifying the mechanisms of domain gain in animal proteins.* Genome Biol, 2010. **11**(7): p. R74. <http://www.ncbi.nlm.nih.gov/pubmed/20633280>\.

212. Da Lage, J.L., G. Feller, and S. Janecek, *Horizontal gene transfer from Eukarya to bacteria and domain shuffling: the alpha-amylase model.* Cell Mol Life Sci, 2004. **61**(1): p. 97-109. <http://www.ncbi.nlm.nih.gov/pubmed/14704857>.

213. Ponting, C.P. and R.R. Russell, *The natural history of protein domains.* Annu Rev Biophys Biomol Struct, 2002. **31**: p. 45-71. <http://www.ncbi.nlm.nih.gov/pubmed/11988462>.

214. Basu, M.K., et al., *Evolution of protein domain promiscuity in eukaryotes.* Genome Res, 2008. **18**(3): p. 449-61. <http://www.ncbi.nlm.nih.gov/pubmed/18230802>.

215. Bjorklund, A.K., et al., *Domain rearrangements in protein evolution.* J Mol Biol, 2005. **353**(4): p. 911-23. <http://www.ncbi.nlm.nih.gov/pubmed/16198373>.

216. Kosak, S.T. and M. Groudine, *Gene order and dynamic domains.* Science, 2004. **306**(5696): p. 644-7. <http://www.ncbi.nlm.nih.gov/pubmed/15499009>.

217. Deeds, E.J., H. Hennessey, and E.I. Shakhnovich, *Prokaryotic phylogenies inferred from protein structural domains.* Genome Res, 2005. **15**(3): p. 393-402. <http://www.ncbi.nlm.nih.gov/pubmed/15741510>.

218. Liu, M., et al., *Significant expansion of exon-bordering protein domains during animal proteome evolution.* Nucleic Acids Res, 2005. **33**(1): p. 95-105. <http://www.ncbi.nlm.nih.gov/pubmed/15640447>.

219. Sammeth, M., S. Foissac, and R. Guigo, *A general definition and nomenclature for alternative splicing events.* PLoS Comput Biol, 2008. **4**(8): p. e1000147. <http://www.ncbi.nlm.nih.gov/pubmed/18688268>.

220. Carvalho, R.F., C.V. Feijao, and P. Duque, *On the physiological significance of alternative splicing events in higher plants.* Protoplasma, 2013. **250**(3): p. 639-50. <http://www.ncbi.nlm.nih.gov/pubmed/22961303>.

221. Kelemen, O., et al., *Function of alternative splicing.* Gene, 2013. **514**(1): p. 1-30. <http://www.ncbi.nlm.nih.gov/pubmed/22909801>.

222. Kalsotra, A. and T.A. Cooper, *Functional consequences of developmentally regulated alternative splicing.* Nat Rev Genet, 2011. **12**(10): p. 715-29. <http://www.ncbi.nlm.nih.gov/pubmed/21921927>.

223. Venables, J.P., J. Tazi, and F. Juge, *Regulated functional alternative splicing in Drosophila.* Nucleic Acids Res, 2012. **40**(1): p. 1-10. <http://www.ncbi.nlm.nih.gov/pubmed/21908400>.

224. Chen, M. and J.L. Manley, *Mechanisms of alternative splicing regulation: insights from molecular and genomics approaches.* Nat Rev Mol Cell Biol, 2009. **10**(11): p. 741-54. <http://www.ncbi.nlm.nih.gov/pubmed/19773805>.

225. House, A.E., Lynch, K.W., *Regulation of Alternative Splicing: More than Just the ABCs.* J Biol Chem, 2008. **283**(3): p. 1217 - 1221. .

226. Tarn, W., *Cellular signals modulate alternative splicing.* J Biomed Sci, 2007. **14**: p. 517-522. .

227. Fedor, M.J., *Alternative Splicing Minireview Series: Combinatorial Control Facilitates Splicing Regulation of Gene Expression and Enhances Genome Diversity.* J Biol Chem, 2008. **283**(3): p. 1209 - 1210. .

228. Chen, T.W., et al., *Interrogation of alternative splicing events in duplicated genes during evolution.* BMC Genomics, 2011. **12 Suppl 3**: p. S16. <http://www.ncbi.nlm.nih.gov/pubmed/22369477>.

229. Zhang, P.G., et al., *Extensive divergence in alternative splicing patterns after gene and genome duplication during the evolutionary history of Arabidopsis.* Mol Biol Evol, 2010. **27**(7): p. 1686-97. <http://www.ncbi.nlm.nih.gov/pubmed/20185454>.

230. Evlampiev, K. and H. Isambert, *Modeling protein network evolution under genome duplication and domain shuffling.* BMC Syst Biol, 2007. **1**: p. 49. <http://www.ncbi.nlm.nih.gov/pubmed/17999763>.

231. Barbosa-Morais, N.L., et al., *The evolutionary landscape of alternative splicing in vertebrate species.* Science, 2012. **338**(6114): p. 1587-93. <http://www.ncbi.nlm.nih.gov/pubmed/23258890>.

232. Mudge, J.M., et al., *The origins, evolution, and functional potential of alternative splicing in vertebrates.* Mol Biol Evol, 2011. **28**(10): p. 2949-59. <http://www.ncbi.nlm.nih.gov/pubmed/21551269>.

233. Furuta, Y., et al., *Domain movement within a gene: a novel evolutionary mechanism for protein diversification.* PLoS One, 2011. **6**(4): p. e18819. <http://www.ncbi.nlm.nih.gov/pubmed/21533192>.

234. Paulding, C.A., M. Ruvolo, and D.A. Haber, *The Tre2 (USP6) oncogene is a hominoid-specific gene.* Proc Natl Acad Sci U S A, 2003. **100**(5): p. 2507-11. <http://www.ncbi.nlm.nih.gov/pubmed/12604796>.

235. Ciccarelli, F.D., et al., *Complex genomic rearrangements lead to novel primate gene function.* Genome Res, 2005. **15**(3): p. 343-51. <http://www.ncbi.nlm.nih.gov/pubmed/15710750>.

236. She, X., et al., *The structure and evolution of centromeric transition regions within the human genome.* Nature, 2004. **430**(7002): p. 857-64. <http://www.ncbi.nlm.nih.gov/pubmed/15318213>.

237. Babushok, D.V., et al., *A novel testis ubiquitin-binding protein gene arose by exon shuffling in hominoids.* Genome Res, 2007. **17**(8): p. 1129-38. <http://www.ncbi.nlm.nih.gov/pubmed/17623810>.

238. Williams, G.J., et al., *Structural insights into NHEJ: building up an integrated picture of the dynamic DSB repair super complex, one component and interaction at a time.* DNA Repair (Amst), 2014. **17**: p. 110-20. <http://www.ncbi.nlm.nih.gov/pubmed/24656613>.

239. Sisu, C., et al., *Comparative analysis of pseudogenes across three phyla.* Proc Natl Acad Sci U S A, 2014. **111**(37): p. 13361-6. <http://www.ncbi.nlm.nih.gov/pubmed/25157146>.

240. Xing, J., et al., *Emergence of primate genes by retrotransposon-mediated sequence transduction.* Proc Natl Acad Sci U S A, 2006. **103**(47): p. 17608-13. <http://www.ncbi.nlm.nih.gov/pubmed/17101974>.

241. Cordaux, R. and M.A. Batzer, *The impact of retrotransposons on human genome evolution.* Nat Rev Genet, 2009. **10**(10): p. 691-703. <http://www.ncbi.nlm.nih.gov/pubmed/19763152>.

242. Jividen, K. and H. Li, *Chimeric RNAs generated by intergenic splicing in normal and cancer cells.* Genes Chromosomes Cancer, 2014. **53**(12): p. 963-71. <http://www.ncbi.nlm.nih.gov/pubmed/25131334>.

243. Okonechnikov, K., et al., *InFusion: Advancing Discovery of Fusion Genes and Chimeric Transcripts from Deep RNA-Sequencing Data.* PLoS One, 2016. **11**(12): p. e0167417. <http://www.ncbi.nlm.nih.gov/pubmed/27907167>.

244. Kozlov, A.P., *Expression of evolutionarily novel genes in tumors.* Infect Agent Cancer, 2016. **11**: p. 34. <http://www.ncbi.nlm.nih.gov/pubmed/27437030>.

245. Narsing, S., et al., *Genes that contribute to cancer fusion genes are large and evolutionarily conserved.* Cancer Genet Cytogenet, 2009. **191**(2): p. 78-84. <http://www.ncbi.nlm.nih.gov/pubmed/19446742>.

246. Kumar-Sinha, C., S. Kalyana-Sundaram, and A.M. Chinnaiyan, *Landscape of gene fusions in epithelial cancers: seq and ye shall find.* Genome Med, 2015. **7**: p. 129. <http://www.ncbi.nlm.nih.gov/pubmed/26684754>.

247. Annala, M.J., et al., *Fusion genes and their discovery using high throughput sequencing.* Cancer Lett, 2013. **340**(2): p. 192-200. <http://www.ncbi.nlm.nih.gov/pubmed/23376639>.

248. Saleem, M. and N.M. Yusoff, *Fusion genes in malignant neoplastic disorders of haematopoietic system.* Hematology, 2016. **21**(9): p. 501-12. <http://www.ncbi.nlm.nih.gov/pubmed/26871368>.

249. Mertens, F., C.R. Antonescu, and F. Mitelman, *Gene fusions in soft tissue tumors: Recurrent and overlapping pathogenetic themes.* Genes Chromosomes Cancer, 2016. **55**(4): p. 291-310. <http://www.ncbi.nlm.nih.gov/pubmed/26684580>.

250. Qi, M., et al., *Morphologic features of carcinomas with recurrent gene fusions.* Adv Anat Pathol, 2012. **19**(6): p. 417-24. <http://www.ncbi.nlm.nih.gov/pubmed/23060067>.

251. Latysheva, N.S. and M.M. Babu, *Discovering and understanding oncogenic gene fusions through data intensive computational approaches.* Nucleic Acids Res, 2016. **44**(10): p. 4487-503. <http://www.ncbi.nlm.nih.gov/pubmed/27105842>.

252. Mertens, F., et al., *The emerging complexity of gene fusions in cancer.* Nat Rev Cancer, 2015. **15**(6): p. 371-81. <http://www.ncbi.nlm.nih.gov/pubmed/25998716>.

253. Jia, Y., Z. Xie, and H. Li, *Intergenically Spliced Chimeric RNAs in Cancer.* Trends Cancer, 2016. **2**(9): p. 475-484. <http://www.ncbi.nlm.nih.gov/pubmed/28210711>.

254. Li, H., et al., *Gene fusions and RNA trans-splicing in normal and neoplastic human cells.* Cell Cycle, 2009. **8**(2): p. 218-22. <http://www.ncbi.nlm.nih.gov/pubmed/19158498>.

255. Seki, Y., T. Mizukami, and T. Kohno, *Molecular Process Producing Oncogene Fusion in Lung Cancer Cells by Illegitimate Repair of DNA Double-Strand Breaks.* Biomolecules, 2015. **5**(4): p. 2464-76. <http://www.ncbi.nlm.nih.gov/pubmed/26437441>.

256. Lawson, A.R., et al., *RAF gene fusion breakpoints in pediatric brain tumors are characterized by significant enrichment of sequence microhomology.* Genome Res, 2011. **21**(4): p. 505-14. <http://www.ncbi.nlm.nih.gov/pubmed/21393386>.

257. Khalturin, K., et al., *More than just orphans: are taxonomically-restricted genes important in evolution?* Trends Genet, 2009. **25**(9): p. 404-13. <http://www.ncbi.nlm.nih.gov/pubmed/19716618>.

258. Zhou, K., A. Kuo, and I.V. Grigoriev, *Reverse transcriptase and intron number evolution.* Stem Cell Investig, 2014. **1**: p. 17. <http://www.ncbi.nlm.nih.gov/pubmed/27358863>.

259. Pavesi, A., G. Magiorkinis, and D.G. Karlin, *Viral proteins originated de novo by overprinting can be identified by codon usage: application to the "gene nursery" of Deltaretroviruses.* PLoS Comput Biol, 2013. **9**(8): p. e1003162. <http://www.ncbi.nlm.nih.gov/pubmed/23966842>.

260. Delaye, L., et al., *The origin of a novel gene through overprinting in Escherichia coli.* BMC Evol Biol, 2008. **8**: p. 31. <http://www.ncbi.nlm.nih.gov/pubmed/18226237>.

261. Murphy, D.N. and A. McLysaght, *De novo origin of protein-coding genes in murine rodents.* PLoS One, 2012. **7**(11): p. e48650. <http://www.ncbi.nlm.nih.gov/pubmed/23185269>.

262. Reinhardt, J.A., et al., *De novo ORFs in Drosophila are important to organismal fitness and evolved rapidly from previously non-coding sequences.* PLoS Genet, 2013. **9**(10): p. e1003860. <http://www.ncbi.nlm.nih.gov/pubmed/24146629>.

263. Donoghue, M.T., et al., *Evolutionary origins of Brassicaceae specific genes in Arabidopsis thaliana.* BMC Evol Biol, 2011. **11**: p. 47. <http://www.ncbi.nlm.nih.gov/pubmed/21332978>.

264. Wu, D.D., et al., *"Out of pollen" hypothesis for origin of new genes in flowering plants: study from Arabidopsis thaliana.* Genome Biol Evol, 2014. **6**(10): p. 2822-9. <http://www.ncbi.nlm.nih.gov/pubmed/25237051>.

265. Li, C.Y., et al., *A human-specific de novo protein-coding gene associated with human brain functions.* PLoS Comput Biol, 2010. **6**(3): p. e1000734. <http://www.ncbi.nlm.nih.gov/pubmed/20376170>.

266. Betran, E., K. Thornton, and M. Long, *Retroposed new genes out of the X in Drosophila.* Genome Res, 2002. **12**(12): p. 1854-9. <http://www.ncbi.nlm.nih.gov/pubmed/12466289>.

267. Schmidt, E.E. and C.J. Davies, *The origins of polypeptide domains.* Bioessays, 2007. **29**(3): p. 262-70. <http://www.ncbi.nlm.nih.gov/pubmed/17295290>.

268. Tajnik, M., et al., *Intergenic Alu exonisation facilitates the evolution of tissue-specific transcript ends.* Nucleic Acids Res, 2015. **43**(21): p. 10492-505. <http://www.ncbi.nlm.nih.gov/pubmed/26400176>.

269. Kwon, Y.J., et al., *Structure and Expression Analyses of SVA Elements in Relation to Functional Genes.* Genomics Inform, 2013. **11**(3): p. 142-8. <http://www.ncbi.nlm.nih.gov/pubmed/24124410>.

270. Park, S.J., et al., *Gain of a New Exon by a Lineage-Specific Alu Element-Integration Event in the BCS1L Gene during Primate Evolution.* Mol Cells, 2015. **38**(11): p. 950-8. <http://www.ncbi.nlm.nih.gov/pubmed/26537194>.

271. Mandal, A.K., et al., *Transcriptome-wide expansion of non-coding regulatory switches: evidence from co-occurrence of Alu exonization, antisense and editing.* Nucleic Acids Res, 2013. **41**(4): p. 2121-37. <http://www.ncbi.nlm.nih.gov/pubmed/23303787>.

272. Zarnack, K., et al., *Direct competition between hnRNP C and U2AF65 protects the transcriptome from the exonization of Alu elements.* Cell, 2013. **152**(3): p. 453-66. <http://www.ncbi.nlm.nih.gov/pubmed/23374342>.

273. Moller-Krull, M., et al., *Beyond DNA: RNA editing and steps toward Alu exonization in primates.* J Mol Biol, 2008. **382**(3): p. 601-9. <http://www.ncbi.nlm.nih.gov/pubmed/18680752>.

274. Grover, D., et al., *Alu repeat analysis in the complete human genome: trends and variations with respect to genomic composition.* Bioinformatics, 2004. **20**(6): p. 813-7. <http://www.ncbi.nlm.nih.gov/pubmed/14751968>.

275. Hormozdiari, F., et al., *Alu repeat discovery and characterization within human genomes.* Genome Res, 2011. **21**(6): p. 840-9. <http://www.ncbi.nlm.nih.gov/pubmed/21131385>.

276. Corvelo, A. and E. Eyras, *Exon creation and establishment in human genes.* Genome Biol, 2008. **9**(9): p. R141. <http://www.ncbi.nlm.nih.gov/pubmed/18811936>.

277. Wu, M., L. Li, and Z. Sun, *Transposable element fragments in protein-coding regions and their contributions to human functional proteins.* Gene, 2007. **401**(1-2): p. 165-71. <http://www.ncbi.nlm.nih.gov/pubmed/17716834>.

278. Schwartz, S., et al., *Alu exonization events reveal features required for precise recognition of exons by the splicing machinery.* PLoS Comput Biol, 2009. **5**(3): p. e1000300. <http://www.ncbi.nlm.nih.gov/pubmed/19266014>.

279. Lev-Maor, G., et al., *RNA-editing-mediated exon evolution.* Genome Biol, 2007. **8**(2): p. R29. <http://www.ncbi.nlm.nih.gov/pubmed/17326827>.

280. Neme, R. and D. Tautz, *Phylogenetic patterns of emergence of new genes support a model of frequent de novo evolution.* BMC Genomics, 2013. **14**: p. 117. <http://www.ncbi.nlm.nih.gov/pubmed/23433480>.

281. Wu, W., et al., *Flock house virus RNA polymerase initiates RNA synthesis de novo and possesses a terminal nucleotidyl transferase activity.* PLoS One, 2014. **9**(1): p. e86876. <http://www.ncbi.nlm.nih.gov/pubmed/24466277>.

282. Troshchynsky, A., et al., *Functional analyses of polymorphic variants of human terminal deoxynucleotidyl transferase.* Genes Immun, 2015. **16**(6): p. 388-98. <http://www.ncbi.nlm.nih.gov/pubmed/26043173>.

283. Motea, E.A. and A.J. Berdis, *Terminal deoxynucleotidyl transferase: the story of a misguided DNA polymerase.* Biochim Biophys Acta, 2010. **1804**(5): p. 1151-66. <http://www.ncbi.nlm.nih.gov/pubmed/19596089>.

284. Thai, T.H. and J.F. Kearney, *Isoforms of terminal deoxynucleotidyltransferase: developmental aspects and function.* Adv Immunol, 2005. **86**: p. 113-36. <http://www.ncbi.nlm.nih.gov/pubmed/15705420>.

285. Bentolila, L.A., et al., *Constitutive expression of terminal deoxynucleotidyl transferase in transgenic mice is sufficient for N region diversity to occur at any Ig locus throughout B cell differentiation.* J Immunol, 1997. **158**(2): p. 715-23. <http://www.ncbi.nlm.nih.gov/pubmed/8992987>.

286. Komori, T., et al., *Lack of N regions in antigen receptor variable region genes of TdT-deficient lymphocytes.* Science, 1993. **261**(5125): p. 1171-5. <http://www.ncbi.nlm.nih.gov/pubmed/8356451>.

287. Nick McElhinny, S.A. and D.A. Ramsden, *Sibling rivalry: competition between Pol X family members in V(D)J recombination and general double strand break repair.* Immunol Rev, 2004. **200**: p. 156-64. <http://www.ncbi.nlm.nih.gov/pubmed/15242403>.

288. Yamtich, J. and J.B. Sweasy, *DNA polymerase family X: function, structure, and cellular roles.* Biochim Biophys Acta, 2010. **1804**(5): p. 1136-50. <http://www.ncbi.nlm.nih.gov/pubmed/19631767>.

289. Gouge, J., et al., *Structural basis for a novel mechanism of DNA bridging and alignment in eukaryotic DSB DNA repair.* Embo J, 2015. **34**(8): p. 1126-42. <http://www.ncbi.nlm.nih.gov/pubmed/25762590>.

290. Black, S.J., et al., *DNA Polymerase theta: A Unique Multifunctional End-Joining Machine.* Genes (Basel), 2016. **7**(9). <http://www.ncbi.nlm.nih.gov/pubmed/27657134>.

291. Wyatt, D.W., et al., *Essential Roles for Polymerase theta-Mediated End Joining in the Repair of Chromosome Breaks.* Mol Cell, 2016. **63**(4): p. 662-73. <http://www.ncbi.nlm.nih.gov/pubmed/27453047>.

292. Sakofsky, C.J., et al., *Translesion Polymerases Drive Microhomology-Mediated Break-Induced Replication Leading to Complex Chromosomal Rearrangements.* Mol Cell, 2015. **60**(6): p. 860-72. <http://www.ncbi.nlm.nih.gov/pubmed/26669261>.

293. Morrish, T.A., et al., *DNA repair mediated by endonuclease-independent LINE-1 retrotransposition.* Nat Genet, 2002. **31**(2): p. 159-65. <http://www.ncbi.nlm.nih.gov/pubmed/12006980>.

294. Storici, F., et al., *RNA-templated DNA repair.* Nature, 2007. **447**(7142): p. 338-41. <http://www.ncbi.nlm.nih.gov/pubmed/17429354>.

295. Meers, C., H. Keskin, and F. Storici, *DNA repair by RNA: Templated, or not templated, that is the question.* DNA Repair (Amst), 2016. **44**: p. 17-21. <http://www.ncbi.nlm.nih.gov/pubmed/27237587>.

296. Keskin, H., et al., *Transcript-RNA-templated DNA recombination and repair.* Nature, 2014. **515**(7527): p. 436-9. <http://www.ncbi.nlm.nih.gov/pubmed/25186730>.

297. Ono, R., et al., *Double strand break repair by capture of retrotransposon sequences and reverse-transcribed spliced mRNA sequences in mouse zygotes.* Sci Rep, 2015. **5**: p. 12281. <http://www.ncbi.nlm.nih.gov/pubmed/26216318>.

298. Kung, P.C., et al., *Terminal deoxynucleotidyl transferase in the diagnosis of leukemia and malignant lymphoma.* Am J Med, 1978. **64**(5): p. 788-94. <http://www.ncbi.nlm.nih.gov/pubmed/347933>.

299. Munsch, N., et al., *Evolution of DNA polymerase alpha, beta and terminal deoxynucleotidyl transferase in hamster lymphoid populations during the development of different types of tumors.* Biomed Pharmacother, 1982. **36**(10): p. 440-4. <http://www.ncbi.nlm.nih.gov/pubmed/7184515>.

300. Ji, J.P. and L.A. Loeb, *Fidelity of HIV-1 reverse transcriptase copying RNA in vitro.* Biochemistry, 1992. **31**(4): p. 954-8. <http://www.ncbi.nlm.nih.gov/pubmed/1370910>.

301. Menendez-Arias, L., *Molecular basis of fidelity of DNA synthesis and nucleotide specificity of retroviral reverse transcriptases.* Prog Nucleic Acid Res Mol Biol, 2002. **71**: p. 91-147. <http://www.ncbi.nlm.nih.gov/pubmed/12102562>.

302. Smith, H.C., *RNA binding to APOBEC deaminases; Not simply a substrate for C to U editing.* RNA Biol, 2016: p. 1-13. <http://www.ncbi.nlm.nih.gov/pubmed/27869537>.

303. Salter, J.D., R.P. Bennett, and H.C. Smith, *The APOBEC Protein Family: United by Structure, Divergent in Function.* Trends Biochem Sci, 2016. **41**(7): p. 578-94. <http://www.ncbi.nlm.nih.gov/pubmed/27283515>.

304. Bass, B.L., *RNA editing and hypermutation by adenosine deamination.* Trends Biochem Sci, 1997. **22**(5): p. 157-62. <http://www.ncbi.nlm.nih.gov/pubmed/9175473>.

305. Wheeler, E.C., et al., *Noncoding regions of C. elegans mRNA undergo selective adenosine to inosine deamination and contain a small number of editing sites per transcript.* RNA Biol, 2015. **12**(2): p. 162-74. <http://www.ncbi.nlm.nih.gov/pubmed/25826568>.

306. Sakurai, M., et al., *Inosine cyanoethylation identifies A-to-I RNA editing sites in the human transcriptome.* Nat Chem Biol, 2010. **6**(10): p. 733-40. <http://www.ncbi.nlm.nih.gov/pubmed/20835228>.

307. Oz-Gleenberg, I., E. Herzig, and A. Hizi, *Template-independent DNA synthesis activity associated with the reverse transcriptase of the long terminal repeat retrotransposon Tf1.* FEBS J, 2012. **279**(1): p. 142-53. <http://www.ncbi.nlm.nih.gov/pubmed/22035236>.

308. Brosius, J., *RNAs from all categories generate retrosequences that may be exapted as novel genes or regulatory elements.* Gene, 1999. **238**: p. 115–134. <http://www.ncbi.nlm.nih.gov/pubmed/10570990>.

309. Loeb, L.A., C.F. Springgate, and N. Battula, *Errors in DNA replication as a basis of malignant changes.* Cancer Res, 1974. **34**(9): p. 2311-21. <http://www.ncbi.nlm.nih.gov/pubmed/4136142>.

310. Venkatesan, R.N. and L.A. Loeb, *The multiplicity of mutations in human cancers.* Adv Exp Med Biol, 2005. **570**: p. 3-17. <http://www.ncbi.nlm.nih.gov/pubmed/18727496>.

311. Loeb, L.A., *Mutator phenotype may be required for multistage carcinogenesis.* Cancer Res, 1991. **51**(12): p. 3075-9. <http://www.ncbi.nlm.nih.gov/pubmed/2039987>.

312. Kaer, K. and M. Speek, *Retroelements in human disease.* Gene, 2013. **518**(2): p. 231-41. <http://www.ncbi.nlm.nih.gov/pubmed/23333607>.

313. Sinibaldi-Vallebona, P., C. Matteucci, and C. Spadafora, *Retrotransposon-encoded reverse transcriptase in the genesis, progression and cellular plasticity of human cancer.* Cancers (Basel), 2011. **3**(1): p. 1141-57. <http://www.ncbi.nlm.nih.gov/pubmed/24212657>.

314. Fraser, J., et al., *Chromatin conformation signatures of cellular differentiation.* Genome Biol, 2009. **10**(4): p. R37. <http://www.ncbi.nlm.nih.gov/pubmed/19374771>.

315. Dekker, J., et al., *Capturing chromosome conformation.* Science, 2002. **295**(5558): p. 1306-11. <http://www.ncbi.nlm.nih.gov/pubmed/11847345>.

316. Cao, R. and J. Cheng, *Deciphering the association between gene function and spatial gene-gene interactions in 3D human genome conformation.* BMC Genomics, 2015. **16**: p. 880. <http://www.ncbi.nlm.nih.gov/pubmed/26511362>.

317. Phillips-Cremins, J.E., et al., *Architectural Protein Subclasses Shape 3D Organization of Genomes during Lineage Commitment.* Cell, 2013. **153**(6): p. 1281-95. <http://www.ncbi.nlm.nih.gov/pubmed/23706625>.

318. Sanyal, A., et al., *The long-range interaction landscape of gene promoters.* Nature, 2012. **489**(7414): p. 109-13. <http://www.ncbi.nlm.nih.gov/pubmed/22955621>.

319. Thurman, R.E., et al., *The accessible chromatin landscape of the human genome.* Nature, 2012. **489**(7414): p. 75-82. <http://www.ncbi.nlm.nih.gov/pubmed/22955617>.

320. Zedek, F., et al., *Correlated evolution of LTR retrotransposons and genome size in the genus Eleocharis.* BMC Plant Biol, 2010. **10**: p. 265. <http://www.ncbi.nlm.nih.gov/pubmed/21118487>.

321. Ecker, J.R., et al., *Genomics: ENCODE explained.* Nature, 2012. **489**(7414): p. 52-5. <http://www.ncbi.nlm.nih.gov/pubmed/22955614>.

322. Birney, E., *The making of ENCODE: Lessons for big-data projects.* Nature, 2012. **489**(7414): p. 49-51. <http://www.ncbi.nlm.nih.gov/pubmed/22955613>.

323. Skipper, M., R. Dhand, and P. Campbell, *Presenting ENCODE.* Nature, 2012. **489**(7414): p. 45. <http://www.ncbi.nlm.nih.gov/pubmed/22955612>.

324. Piriyapongsa, J., L. Marino-Ramirez, and I.K. Jordan, *Origin and evolution of human microRNAs from transposable elements.* Genetics, 2007. **176**(2): p. 1323-37. <http://www.ncbi.nlm.nih.gov/pubmed/17435244>.

325. Qin, S., et al., *The Role of Transposable Elements in the Origin and Evolution of MicroRNAs in Human.* PLoS One, 2015. **10**(6): p. e0131365. <http://www.ncbi.nlm.nih.gov/pubmed/26115450>.

326. Biemont, C. and C. Vieira, *Genetics: junk DNA as an evolutionary force.* Nature, 2006. **443**(7111): p. 521-4. <http://www.ncbi.nlm.nih.gov/pubmed/17024082>.

327. Brunet, T.D. and W.F. Doolittle, *Multilevel Selection Theory and the Evolutionary Functions of Transposable Elements.* Genome Biol Evol, 2015. **7**(8): p. 2445-57. <http://www.ncbi.nlm.nih.gov/pubmed/26253318>.

328. Wang, K., G. Huang, and Y. Zhu, *Transposable elements play an important role during cotton genome evolution and fiber cell development.* Sci China Life Sci, 2016. **59**(2): p. 112-21. <http://www.ncbi.nlm.nih.gov/pubmed/26687725>.

329. Polavarapu, N., et al., *Evolutionary rates and patterns for human transcription factor binding sites derived from repetitive DNA.* BMC Genomics, 2008. **9**: p. 226. <http://www.ncbi.nlm.nih.gov/pubmed/18485226>.

330. Huda, A., et al., *Prediction of transposable element derived enhancers using chromatin modification profiles.* PLoS One, 2011. **6**(11): p. e27513. <http://www.ncbi.nlm.nih.gov/pubmed/22087331>.

331. Xie, M., et al., *DNA hypomethylation within specific transposable element families associates with tissue-specific enhancer landscape.* Nat Genet, 2013. **45**(7): p. 836-41. <http://www.ncbi.nlm.nih.gov/pubmed/23708189>.

332. Huda, A., et al., *Epigenetic regulation of transposable element derived human gene promoters.* Gene, 2011. **475**(1): p. 39-48. <http://www.ncbi.nlm.nih.gov/pubmed/21215797>.

333. Miller, W.J. and P. Capy, *Mobile genetic elements as natural tools for genome evolution.* Methods Mol Biol, 2004. **260**: p. 1-20. <http://www.ncbi.nlm.nih.gov/pubmed/15020798>.

334. Biemont, C., *A brief history of the status of transposable elements: from junk DNA to major players in evolution.* Genetics, 2010. **186**(4): p. 1085-93. <http://www.ncbi.nlm.nih.gov/pubmed/21156958>.

335. Oliver, K.R. and W.K. Greene, *Transposable elements: powerful facilitators of evolution.* Bioessays, 2009. **31**(7): p. 703-14. <http://www.ncbi.nlm.nih.gov/pubmed/19415638>.

336. Hedges, D.J. and M.A. Batzer, *From the margins of the genome: mobile elements shape primate evolution.* Bioessays, 2005. **27**(8): p. 785-94. <http://www.ncbi.nlm.nih.gov/pubmed/16015599>.

337. Belyayev, A., *Bursts of transposable elements as an evolutionary driving force.* J Evol Biol, 2014. **27**(12): p. 2573-84. <http://www.ncbi.nlm.nih.gov/pubmed/25290698>.

338. Dimitri, P. and N. Junakovic, *Revising the selfish DNA hypothesis: new evidence on accumulation of transposable elements in heterochromatin.* Trends Genet, 1999. **15**(4): p. 123-4. <http://www.ncbi.nlm.nih.gov/pubmed/10203812>.

339. Matharu, N.K. and S.H. Ahanger, *Chromatin Insulators and Topological Domains: Adding New Dimensions to 3D Genome Architecture.* Genes (Basel), 2015. **6**(3): p. 790-811. <http://www.ncbi.nlm.nih.gov/pubmed/26340639>.

340. Feschotte, C., *Transposable elements and the evolution of regulatory networks.* Nat Rev Genet, 2008. **9**(5): p. 397-405. <http://www.ncbi.nlm.nih.gov/pubmed/18368054>.

341. Rebollo, R., M.T. Romanish, and D.L. Mager, *Transposable elements: an abundant and natural source of regulatory sequences for host genes.* Annu Rev Genet, 2012. **46**: p. 21-42. <http://www.ncbi.nlm.nih.gov/pubmed/22905872>.

342. Cowley, M. and R.J. Oakey, *Transposable elements re-wire and fine-tune the transcriptome.* PLoS Genet, 2013. **9**(1): p. e1003234. <http://www.ncbi.nlm.nih.gov/pubmed/23358118>.

343. van de Lagemaat, L.N., et al., *Transposable elements in mammals promote regulatory variation and diversification of genes with specialized functions.* Trends Genet, 2003. **19**(10): p. 530-6. <http://www.ncbi.nlm.nih.gov/pubmed/14550626>.

344. Castanera, R., et al., *Transposable Elements versus the Fungal Genome: Impact on Whole-Genome Architecture and Transcriptional Profiles.* PLoS Genet, 2016. **12**(6): p. e1006108. <http://www.ncbi.nlm.nih.gov/pubmed/27294409>.

345. Bennetzen, J.L. and H. Wang, *The contributions of transposable elements to the structure, function, and evolution of plant genomes.* Annu Rev Plant Biol, 2014. **65**: p. 505-30. <http://www.ncbi.nlm.nih.gov/pubmed/24579996>.

346. Shapiro, J.A., *Exploring the read-write genome: mobile DNA and mammalian adaptation.* Crit Rev Biochem Mol Biol, 2016: p. 1-17. <http://www.ncbi.nlm.nih.gov/pubmed/27599542>.

347. Bartel, D.P., *MicroRNAs: genomics, biogenesis, mechanism, and function.* Cell, 2004. **116**(2): p. 281-97. <http://www.ncbi.nlm.nih.gov/pubmed/14744438>.

348. Smalheiser, N.R. and V.I. Torvik, *Mammalian microRNAs derived from genomic repeats.* Trends Genet, 2005. **21**(6): p. 322-6. <http://www.ncbi.nlm.nih.gov/pubmed/15922829>.

349. Sun, J., et al., *Characterization and evolution of microRNA genes derived from repetitive elements and duplication events in plants.* PLoS One, 2012. **7**(4): p. e34092. <http://www.ncbi.nlm.nih.gov/pubmed/22523544>.

350. Yuan, Z., et al., *MicroRNA genes derived from repetitive elements and expanded by segmental duplication events in mammalian genomes.* PLoS One, 2011. **6**(3): p. e17666. <http://www.ncbi.nlm.nih.gov/pubmed/21436881>.

351. Borchert, G.M., et al., *Comprehensive analysis of microRNA genomic loci identifies pervasive repetitive-element origins.* Mob Genet Elements, 2011. **1**(1): p. 8-17. <http://www.ncbi.nlm.nih.gov/pubmed/22016841>.

352. Hezroni, H., et al., *Principles of long noncoding RNA evolution derived from direct comparison of transcriptomes in 17 species.* Cell Rep, 2015. **11**(7): p. 1110-22. <http://www.ncbi.nlm.nih.gov/pubmed/25959816>.

353. Gaiti, F., et al., *Dynamic and Widespread lncRNA Expression in a Sponge and the Origin of Animal Complexity.* Mol Biol Evol, 2015. **32**(9): p. 2367-82. <http://www.ncbi.nlm.nih.gov/pubmed/25976353>.

354. Kannan, S., et al., *Transposable Element Insertions in Long Intergenic Non-Coding RNA Genes.* Front Bioeng Biotechnol, 2015. **3**: p. 71. <http://www.ncbi.nlm.nih.gov/pubmed/26106594>.

355. Campo-Paysaa, F., et al., *microRNA complements in deuterostomes: origin and evolution of microRNAs.* Evol Dev, 2011. **13**(1): p. 15-27. <http://www.ncbi.nlm.nih.gov/pubmed/21210939>.

356. Witkin, E.M., *Nuclear segregation and the delayed appearance of induced mutants in Escherichia coli.* Cold Spring Harb Symp Quant Biol, 1951. **16**: p. 357-72. <http://www.ncbi.nlm.nih.gov/pubmed/14942750>.

357. Witkin, E.M., *Effects of Temperature on Spontaneous and Induced Mutations in Escherichia Coli.* Proc Natl Acad Sci U S A, 1953. **39**(5): p. 427-33. <http://www.ncbi.nlm.nih.gov/pubmed/16589286>.

358. Witkin, E.M., *The use of sodium nucleate in the study of the mutagenic activity of acriflavine in Escherichia coli.* Proc Natl Acad Sci U S A, 1950. **36**(12): p. 724-31. <http://www.ncbi.nlm.nih.gov/pubmed/14808162>.

359. Al-Khedery, B. and D.R. Allred, *Antigenic variation in Babesia bovis occurs through segmental gene conversion of the ves multigene family, within a bidirectional locus of active transcription.* Mol Microbiol, 2006. **59**(2): p. 402-14. <http://www.ncbi.nlm.nih.gov/pubmed/16390438>.

360. Stringer, J.R., *Antigenic variation in pneumocystis.* J Eukaryot Microbiol, 2007. **54**(1): p. 8-13. <http://www.ncbi.nlm.nih.gov/pubmed/17300510>.

361. Vink, C., G. Rudenko, and H.S. Seifert, *Microbial antigenic variation mediated by homologous DNA recombination.* FEMS Microbiol Rev, 2012. **36**(5): p. 917-48. <http://www.ncbi.nlm.nih.gov/pubmed/22212019>.

362. Hardianti, M.S., et al., *Activation-induced cytidine deaminase expression in follicular lymphoma: association between AID expression and ongoing mutation in FL.* Leukemia, 2004. **18**(4): p. 826-31. <http://www.ncbi.nlm.nih.gov/pubmed/14990977>.

363. Papaemmanuil, E., et al., *RAG-mediated recombination is the predominant driver of oncogenic rearrangement in ETV6-RUNX1 acute lymphoblastic leukemia.* Nat Genet, 2014. **46**(2): p. 116-25. <http://www.ncbi.nlm.nih.gov/pubmed/24413735>.

364. Robbiani, D.F. and M.C. Nussenzweig, *Chromosome translocation, B cell lymphoma, and activation-induced cytidine deaminase.* Annu Rev Pathol, 2013. **8**: p. 79-103. <http://www.ncbi.nlm.nih.gov/pubmed/22974238>.

365. Halper-Stromberg, E., et al., *Fine mapping of V(D)J recombinase mediated rearrangements in human lymphoid malignancies.* BMC Genomics, 2013. **14**: p. 565. <http://www.ncbi.nlm.nih.gov/pubmed/23957733>.

366. Lee, E., et al., *Landscape of somatic retrotransposition in human cancers.* Science, 2012. **337**(6097): p. 967-71. <http://www.ncbi.nlm.nih.gov/pubmed/22745252>.

367. Rode, A., et al., *Chromothripsis in cancer cells: An update.* Int J Cancer, 2016. **138**(10): p. 2322-33. <http://www.ncbi.nlm.nih.gov/pubmed/26455580>.

368. de Pagter, M.S. and W.P. Kloosterman, *The Diverse Effects of Complex Chromosome Rearrangements and Chromothripsis in Cancer Development.* Recent Results Cancer Res, 2015. **200**: p. 165-93. <http://www.ncbi.nlm.nih.gov/pubmed/26376877>.

369. Rausch, T., et al., *Genome sequencing of pediatric medulloblastoma links catastrophic DNA rearrangements with TP53 mutations.* Cell, 2012. **148**(1-2): p. 59-71. <http://www.ncbi.nlm.nih.gov/pubmed/22265402>.

370. Ye, K., et al., *Systematic discovery of complex insertions and deletions in human cancers.* Nat Med, 2015. <http://www.ncbi.nlm.nih.gov/pubmed/26657142>.

371. Chandra, H.S., et al., *Philadelphia Chromosome Symposium: commemoration of the 50th anniversary of the discovery of the Ph chromosome.* Cancer Genet, 2011. **204**(4): p. 171-9. <http://www.ncbi.nlm.nih.gov/pubmed/21536234>.

372. Caporale, L.H., *Overview of the creative genome: effects of genome structure and sequence on the generation of variation and evolution.* Ann N Y Acad Sci, 2012. **1267**(1): p. 1-10. <http://www.ncbi.nlm.nih.gov/pubmed/22954209>.

373. Damiani, G., *The Yin and Yang of anti-Darwinian epigenetics and Darwinian genetics.* Riv Biol, 2007. **100**(3): p. 361-402. <http://www.ncbi.nlm.nih.gov/pubmed/18278738>.

374. Ploeger, A. and F. Galis, *Evo Devo and cognitive science.* Wiley Interdiscip Rev Cogn Sci, 2011. **2**(4): p. 429-40. <http://www.ncbi.nlm.nih.gov/pubmed/26302202>.

375. Affifi, R., *The Semiosis of "Side Effects" in Genetic Interventions.* Biosemiotics, 2016. **9**(3): p. 345-364. <http://www.ncbi.nlm.nih.gov/pubmed/28066514>.

376. Liu, S., et al., *Mu transposon insertion sites and meiotic recombination events co-localize with epigenetic marks for open chromatin across the maize genome.* PLoS Genet, 2009. **5**(11): p. e1000733. <http://www.ncbi.nlm.nih.gov/pubmed/19936291>.

377. Baller, J.A., J. Gao, and D.F. Voytas, *Access to DNA establishes a secondary target site bias for the yeast retrotransposon Ty5.* Proc Natl Acad Sci U S A, 2011. **108**(51): p. 20351-6. <http://www.ncbi.nlm.nih.gov/pubmed/21788500>.

378. Gangadharan, S., et al., *DNA transposon Hermes inserts into DNA in nucleosome-free regions in vivo.* Proc Natl Acad Sci U S A, 2010. **107**(51): p. 21966-72. <http://www.ncbi.nlm.nih.gov/pubmed/21131571>.
