# Supplementary material for: Living Organisms Author Their Read-Write Genomes in Evolution
Source: Biology (Basel). 2017 Dec 6;6(4):42. doi: 10.3390/biology6040042 (PMC5745447; doi:10.3390/biology6040042)
Supplement: Supplementary file 1 [file biology-06-00042-s001.tgz › biology-224185-supplementary & PUBMED links/biology-224185.zip/Shapiro - Living Organisms Author Their Read-Write Genomes in Evolution - Supplemental Material.Renumbered and Approved + PUBMED links/Supplementary Table S1 Key Scientists Advocating non.docx]

| **Supplementary Table 1. Key Scientists Advocating non-Gradualist Evolution in the 19^th^ and 20th Centuries.** | | |
| --- | --- | --- |
| **Evolutionist** | **Non-gradual Evolutionary Process** | **References** |
| William Bateson (1861-1926) | Discontinuous variation | [[1-5](#_ENREF_1)] |
| Hugo de Vries (1848-1935) | Abrupt mutational variation | [[6](#_ENREF_6), [7](#_ENREF_7)] |
| Konstantin Mereschkowsky (1855–1921) | Evolution by symbiogenesis | [[8](#_ENREF_8), [9](#_ENREF_9)] |
| Ivan E. Wallin (1883-1969) | Evolution by symbiogenesis (“Symbionticism”) | [[10-12](#_ENREF_10)] |
| Kozo-Polyansky, Boris Mikhailovich (1890-1957) | Evolution by symbiogenesis | [[13](#_ENREF_13)] |
| George Gaylord Simpson (1902-1984) | Quantum evolution | [[14](#_ENREF_14), [15](#_ENREF_15)] |
| Richard Goldschmidt (1878-1958) | “Hopeful monsters” formed by redirecting developmental programs | [[16-18](#_ENREF_16)] |
| George Ledyard Stebbins (1906-2000) | Hybrid Speciation (“Cataclysmic Evolution”) | [[19-22](#_ENREF_19)] |
| Niles Eldredge (b. 1943) and Stephen J. Gould (1941-2002) | Punctuated equilibrium | [[23-25](#_ENREF_23)] |
| Lynn Margulis (1938-2011) | Evolution by symbiogenesis | [[26-32](#_ENREF_26)] |

REFERENCES

1. Bateson, W., *Materials for the Study of Variation Treated With Especial Regard to Discontinuity in the Origin of Species.*1894, London: Macmillan. .

2. Bateson, P., *William Bateson: a biologist ahead of his time.* J Genet, 2002. **81**(2): p. 49-58. <http://www.ncbi.nlm.nih.gov/pubmed/12532036>.

3. Gillham, N.W., *Evolution by Jumps: Francis Galton and William Bateson and the Mechanism of Evolutionary Change.* Genetics, 2001. **159**: p. 1383-1392. .

4. Peterson, E.L., *William Bateson from Balanoglossus to Materials for the study of variation: the transatlantic roots of discontinuity and the (un)naturalness of selection.* J Hist Biol, 2008. **41**(2): p. 267-305. <http://www.ncbi.nlm.nih.gov/pubmed/19049232>.

5. Gillham, N.W., *Evolution by jumps: Francis Galton and William Bateson and the mechanism of evolutionary change.* Genetics, 2001. **159**(4): p. 1383-92. <http://www.ncbi.nlm.nih.gov/pubmed/11779782>.

6. de Vries, H., *Species and varieties, their origin by mutation; lectures delivered at the University of California*1905, Chicago: Open Court Publishing Co. .

7. de Vries, H., *Die mutationstheorie. Versuche und beobachtungen über die entstehung von arten im pflanzenreich*1901-1903, Leipzig: Veit & comp. .

8. Mereschkowsky, K., *Theorie der zwei Plasmaarten als Grundlage der Symbiogenesis, einer neuen Lehre von der Ent‐stehung der Organismen.* Biologisches Centralblatt, 1910. **30**: p. 353‐367. .

9. Mereschkowsky, K., *Symbiogenesis and the Origin of Species*1926. .

10. Wallin, I.E., *On the nature of mitochondria. I. Observations on mitochondria staining methods applied to bacteria. II. Reactions of bacteria to chemical treatment.* American Journal of Anatomy, 1922. **30**(2): p. 203–229. .

11. Wallin, I.E., *On the nature of mitochondria. III. The demonstration of mitochondria by bacteriological methods. IV. A comparative study of the morphogenesis of root-nodule bacteria and chloroplasts.* American Journal of Anatomy, 1922. **30**(4): p. 451–471. .

12. Wallin, I.E., *Symbionticism and the origin of species*1927, Baltimore: Williams & Wilkins. .

13. Kozo-Polyansky, B.M., *Symbiogenesis: A New Principle of Evolution* 1924 (2010, English translation by Victor Fet), Cambridge: Harvard University Press. .

14. Simpson G.G, *Tempo and Mode in Evolution*1944, New York: Columbia Univ. Press. .

15. Gould, S.J.s., *G. G. Simpson, Paleontology and the Modern Synthesis*, in *The Evolutionary Synthesis*, E. Mayr and W.B. Provine, Editors. 1980, Harvard University Press: Cambridge MA. p. 153–172. .

16. Goldschmidt, R., *The Material Basis of Evolution, Reissued (The Silliman Memorial Lectures Series), 1982*1940, New Haven CT: Yale Univ.Press. .

17. Dietrich, M.R., *Richard Goldschmidt: hopeful monsters and other 'heresies'.* Nat Rev Genet, 2003. **4**(1): p. 68-74. <http://www.ncbi.nlm.nih.gov/pubmed/12509755>.

18. Gould, S.J., *The Return of Hopeful Monsters.* Natural History, 1977. **86**: p. 22-30. .

19. Stebbins, J., G.L., *Cataclysmic Evolution.* Scientific American, 1951. **184**(4): p. 54 –59. .

20. Stebbins, G.L., *The significance of polyploidy in plant evolution.* Am Nat, 1940. **74**: p. 54–66. .

21. Stebbins, G.L., *Processes of Organic Evolution*1971, Englewood Cliffs, New Jersey: Prentice-Hall. .

22. Stebbins, G.L. and F.J. Ayala, *Is a new evolutionary synthesis necessary?* Science, 1981. **213**(4511): p. 967-71. <http://www.ncbi.nlm.nih.gov/pubmed/17789015>.

23. Eldredge, N. and S.J. Gould, *Punctuated equilibria: an alternative to phyletic gradualism*, in *Models in Paleobiology*, T.J.M. Schopf, Editor 1972, Freeman, Cooper and Company: San Francisco. p. 82-115. .

24. Gould, S.J., *Punctuated Equilibrium and the Fossil Record.* Science, 1983. **219**(4584): p. 439-440. <http://www.ncbi.nlm.nih.gov/pubmed/17742803>.

25. Gould, S.J. and N. Eldredge, *Punctuated equilibrium comes of age.* Nature, 1993. **366**(6452): p. 223-7. <http://www.ncbi.nlm.nih.gov/pubmed/8232582>.

26. Sagan, L., *On the origin of mitosing cells.* J Theor Biol, 1967. **14**(3): p. 255-74. <http://www.ncbi.nlm.nih.gov/pubmed/11541392>.

27. Margulis, L., *Symbiosis in Cell Evolution.*1981, London: W.H. Freeman Co. .

28. Sapp, J., *Evolution by Association: A History of Symbiosis*1994, Oxford: Oxford University Press. .

29. Margulis, L., *Symbiosis and evolution.* Sci Am, 1971. **225**(2): p. 48-57. <http://www.ncbi.nlm.nih.gov/pubmed/5089455>.

30. Sapp, J., *Saltational symbiosis.* Theory Biosci, 2010. **129**(2-3): p. 125-33. <http://www.ncbi.nlm.nih.gov/pubmed/20535601>.

31. Margulis, L. and D. Sagan, *Acquiring Genomes: A Theory of the Origins of Species*2002, Amherst, MA: Perseus Books Group. .

32. Margulis, L., *Origin of Eukaryotic Cells*1970: Yale Univ. Press. .
