# Supplementary material for: Living Organisms Author Their Read-Write Genomes in Evolution
Source: Biology (Basel). 2017 Dec 6;6(4):42. doi: 10.3390/biology6040042 (PMC5745447; doi:10.3390/biology6040042)
Supplement: Supplementary file 1 [file biology-06-00042-s001.tgz › biology-224185-supplementary & PUBMED links/biology-224185.zip/Shapiro - Living Organisms Author Their Read-Write Genomes in Evolution - Supplemental Material.Renumbered and Approved + PUBMED links/Supplementary Table S10 Some Reported Instances of Orphan Coding Sequences.docx]

| **Supplementary Table 10. Some Reported Instances of Orphan Coding Sequences (“New genes” and New exons) Discovered in Sequenced Genomes** [[1-13](#_ENREF_1)] | |
| --- | --- |
| **Genome Source** | **Reference(s)** |
| Viruses (bacteriophages) “Almost one-third of all ORFs in 1,456 complete virus genomes correspond to ORFans, a figure significantly larger than that observed in prokaryotes… 38.4% of phage ORFs have no homologs in other phages, and 30.1% have no homologs neither in the viral nor in the prokaryotic world…” | [[14](#_ENREF_14)] |
| “Virus de novo genes…in which an existing gene has been "overprinted" by a new open reading frame, a process that generates a new protein-coding gene overlapping the ancestral gene… young de novo genes have a different codon usage from the rest of the genome…evolve rapidly and are under positive or weak purifying selection...In contrast to young de novo genes, older de novo genes have a codon usage that is similar to the rest of the genome.” | [[15](#_ENREF_15), [16](#_ENREF_16)] |
| Prokaryotes (20,000 orphan sequences) “…only 2.8% of all microbial ORFans have detectable homologs in viruses, while the percentage of non-ORFans with detectable homologs in viruses is 7.9%, a significantly higher figure.” | [[17](#_ENREF_17)] |
| “*Escherichia coli* O157:H7 (EHEC): “72 genes are taxonomically restricted and, therefore, appear to have evolved relatively recently de novo”…“origin of a new gene through overprinting in *Escherichia coli* K12.” | [[18](#_ENREF_18), [19](#_ENREF_19)] |
| Yeast – “BSC4 in *Saccharomyces cerevisiae*…encoding a 132-amino-acid-long peptide… no homologous ORF in…closely related species …Because the corresponding noncoding sequences in *S. paradoxus*, *S. mikatae*, and *S. bayanus* also transcribe, we propose that a new de novo protein-coding gene may have evolved from a previously expressed noncoding sequence.” | [[20](#_ENREF_20)] |
| Yeast and *Drosophila* protein C-termini - the co-option of short segments of noncoding sequence into the C-termini of existing proteins via the loss of a stop codon “…54 examples of C-terminal extensions in *Saccharomyces* and 28 in *Drosophila*, all of them recent enough to still be polymorphic…Four of the *Saccharomyces* C-terminal extensions (to ADH1, ARP8, TPM2, and PIS1)…are predicted to lead to significant modification of a protein domain structure.” | [[21](#_ENREF_21)] |
| Green multicellular algae *Chlamydomonas* and *Volvox carteri*: PHD domain added to condensin II by exonization of mobile DNA sequences; “141 retrogene candidates in total in both genomes, with their fraction being significantly higher in the multicellular Volvox. Majority of the retrogene candidates showed signatures of functional constraints, thus indicating their functionality. Detailed analyses of the identified retrogene candidates, their parental genes, and homologs of both, revealed that most of the retrogene candidates were derived from ancient retroposition events in the common ancestor of the two algae and that the parental genes were subsequently lost from the respective lineages, making many retrogenes 'orphan'.” | [[22](#_ENREF_22), [23](#_ENREF_23)] |
| Plasmodium vivax – “…recent de novo origin of at least 13 protein-coding genes in the genome of *Plasmodium vivax*… five of the genes identified in our analysis contain introns…likely evolved from previously intergenic regions together with the coding sequences.” | [[24](#_ENREF_24)] |
| Nematode *Pristionchus pacificus*; “3818-7545 (39-76 %) of orphan genes are under negative selection” | [[25](#_ENREF_25)] |
| *Drosophila melanogaster*: “There is a significant excess of retrogenes that originate from the X chromosome and retropose to autosomes; new genes retroposed from autosomes are scarce. Further, we found that most of these X-derived autosomal retrogenes had evolved a testis expression pattern.” | [[26](#_ENREF_26)] |
| *Drosophila melanogaster* – “142 segregating and 106 fixed testis-expressed de novo genes in a population sample of *Drosophila melanogaster*…appear to derive primarily from ancestral intergenic, unexpressed open reading frames (ORFs), with natural selection playing a significant role in their spread.” | [[27](#_ENREF_27), [28](#_ENREF_28)] |
| *Drosophila melanogaster* – “…six putatively protein-coding de novo genes described in *Drosophila melanogaster*…two de novo genes emerged from novel long non-coding RNAs…four other de novo genes evolved a translated open reading frame and transcription…suggesting that nascent open reading frames (proto-ORFs)…can contribute to the emergence of a new de novo gene... Sequence and structural evolution of de novo genes was rapid compared to nearby genes...” | [[29](#_ENREF_29)] |
| Insects: “…comparing 30 arthropod genomes, focusing in particular on seven recently sequenced ant genomes…comparison between social *Hymenoptera* (ants and bees) and nonsocial *Diptera* (flies and mosquitoes)…recently split lineages undergo accelerated genomic reorganization, including the rapid gain of many orphan genes…between the two insect orders *Hymenoptera* and *Diptera*, orphan genes are more abundant and emerge more rapidly in *Hymenoptera*, in particular, in leaf-cutter ants. With respect to intragenomic localization, we find that ant orphan genes show little clustering…” | [[30](#_ENREF_30)] |
| Entelegyne spiders (*Araneae*, *Entelegynae*): “…transcriptomes of six entelegyne spider species from three genera (*Cicurina travisae*, *C. vibora*, *Habronattus signatus*, *H. ustulatus*, *Nesticus bishopi*, and *N. cooperi*)… between ~ 550 and 1,100 unique orphan genes were found in each genus.” | [[31](#_ENREF_31)] |
| Rodent: “75 murine genes (69 mouse genes and 6 rat genes) for which there is good evidence of de novo origin since the divergence of mouse and rat. Each of these genes is only found in either the mouse or rat lineages, with no candidate orthologs nor evidence for potentially-unannotated orthologs in the other lineage…For 11 of the 75 candidate novel genes we could identify a mouse-specific mutation that led to the creation of the open reading frame (ORF) specifically in mouse…A large number of them (51 out of 69 mouse genes and 3 out of 6 rat genes) also overlap with other genes, either within introns, or on the opposite strand.” | [[32](#_ENREF_32)] |
| Mouse and human: “…over 5000 new genes were integrated into the ancestral GGI {gene-gene interaction} networks of human and mouse; new genes gradually acquire increasing number of gene partners; some human-specific genes evolved into hub structure with critical phenotypic effects.” | [[33](#_ENREF_33)] |
| Primates: “an unexpected important role of transposable elements in the formation of novel protein-coding genes in the genomes of primates.” | [[34](#_ENREF_34)] |
| Human and Chimpanzee: “…retrocopies of coding transcripts to generate proteins with novel N-terminal domains. Examples include thymopoietin beta (*TMPO*), eukaryotic translation initiation factor 3 subunit 5 (*EIF3F*), and the 5'-inverted retrocopy of small nuclear ribonucleoprotein polypeptide N (*SNRPN*). | [[35](#_ENREF_35)] |
| Humans: “…one human-specific de novo protein-coding gene, *FLJ33706* (alternative gene symbol *C20orf203*)…originated from noncoding DNA sequences: insertion of repeat elements especially *Alu* contributed to the formation of the first coding exon and six standard splice junctions on the branch leading to humans and chimpanzees, and two subsequent substitutions in the human lineage escaped two stop codons and created an open reading frame of 194 amino acids.” | [[36](#_ENREF_36)] |
| Humans: “…we identify 60 new protein-coding genes that originated de novo on the human lineage since divergence from the chimpanzee…RNA-seq data indicate that these genes have their highest expression levels in the cerebral cortex and testes…” | [[37](#_ENREF_37)] |
| Humans: “we identified 24 hominoid-specific de novo protein-coding genes with precise origination timing in vertebrate phylogeny… most of the hominoid-specific de novo protein-coding genes encoded polyadenylated non-coding RNAs in rhesus macaque or chimpanzee with a similar transcript structure and correlated tissue expression profile…the majority of these hominoid-specific de novo protein-coding genes appear to have acquired a regulated transcript structure and expression profile before acquiring coding potential…the coding genes in human often showed higher transcriptional abundance than their non-coding counterparts in rhesus macaque.” | [[38](#_ENREF_38)] |
| Humans: “the de novo origin of at least three human protein-coding genes since the divergence with chimp…chimp, gorilla, gibbon, and macaque share the same disabling sequence difference, supporting the inference that the ancestral sequence was noncoding…We estimate that 0.075% of human genes may have originated through this mechanism leading to a total expectation of 18 such cases in a genome of 24,000 protein-coding genes.” | [[39](#_ENREF_39)] |
| Humans: “We have found 426 different annotated young domains, totalling 995 domain occurrences, which represent about 12.3% of all human domains. We have observed that 61.3% of them arose in newly formed genes, while the remaining 38.7% are found combined with older domains…Young domains are preferentially located at the N-terminus of the protein… young domains show significantly higher non-synonymous to synonymous substitution rates than older domains…” | [[40](#_ENREF_40)] |
| *Arabidopsis*: “…lineage-specific genes within the nuclear (1761 genes) and mitochondrial (28 genes) genomes are identified…Almost a quarter of lineage-specific genes originate from non-lineage-specific paralogs, while the origins of ~10% of lineage-specific genes are partly derived from DNA exapted from transposable elements (twice the proportion observed for non-lineage-specific genes). Lineage-specific genes are also enriched in genes that have overlapping CDS, which is consistent with such novel genes arising from overprinting. Over half of the subset of the 958 lineage-specific genes found only in Arabidopsis thaliana have alignments to intergenic regions in Arabidopsis lyrata, consistent with either de novo origination or differential gene loss and retention… lineage-specific genes have high tissue specificity and low expression levels across multiple tissues and developmental stages. Finally, stress responsiveness is identified as a distinct feature of Brassicaceae-specific genes; where these LSGs are enriched for genes responsive to a wide range of abiotic stresses…” | [[41](#_ENREF_41)] |
| *Arabidopsis*: “…we find that new genes in plants show a bias in expression to mature pollen, and are also enriched in a gene co-expression module that correlates with mature pollen in *Arabidopsis thaliana*. Transposable elements are significantly enriched in the new genes, and the high activity of transposable elements in the vegetative nucleus, compared with the germ cells, suggests that new genes are most easily generated in the vegetative nucleus in the mature pollen. We propose an "out of pollen" hypothesis for the origin of new genes in flowering plants.” | [[42](#_ENREF_42)] |

REFERENCES

1. Long, M., et al., *New gene evolution: little did we know.* Annu Rev Genet, 2013. **47**: p. 307-33. <http://www.ncbi.nlm.nih.gov/pubmed/24050177>.

2. Long, M., et al., *Origin of new genes: evidence from experimental and computational analyses.* Genetica, 2003. **118**(2-3): p. 171-82. <http://www.ncbi.nlm.nih.gov/pubmed/12868607>.

3. Tautz, D. and T. Domazet-Loso, *The evolutionary origin of orphan genes.* Nat Rev Genet, 2011. **12**(10): p. 692-702. <http://www.ncbi.nlm.nih.gov/pubmed/21878963>.

4. Carvunis, A.R., et al., *Proto-genes and de novo gene birth.* Nature, 2012. <http://www.ncbi.nlm.nih.gov/pubmed/22722833>.

5. Basile, W., et al., *High GC content causes orphan proteins to be intrinsically disordered.* PLoS Comput Biol, 2017. **13**(3): p. e1005375. <http://www.ncbi.nlm.nih.gov/pubmed/28355220>.

6. McLysaght, A. and D. Guerzoni, *New genes from non-coding sequence: the role of de novo protein-coding genes in eukaryotic evolutionary innovation.* Philos Trans R Soc Lond B Biol Sci, 2015. **370**(1678): p. 20140332. <http://www.ncbi.nlm.nih.gov/pubmed/26323763>.

7. Kaessmann, H., *Origins, evolution, and phenotypic impact of new genes.* Genome Res, 2010. **20**(10): p. 1313-26. <http://www.ncbi.nlm.nih.gov/pubmed/20651121>.

8. Andersson, D.I., J. Jerlstrom-Hultqvist, and J. Nasvall, *Evolution of new functions de novo and from preexisting genes.* Cold Spring Harb Perspect Biol, 2015. **7**(6). <http://www.ncbi.nlm.nih.gov/pubmed/26032716>.

9. Chen, S., B.H. Krinsky, and M. Long, *New genes as drivers of phenotypic evolution.* Nat Rev Genet, 2013. **14**(9): p. 645-60. <http://www.ncbi.nlm.nih.gov/pubmed/23949544>.

10. Schmitz, J.F. and E. Bornberg-Bauer, *Fact or fiction: updates on how protein-coding genes might emerge de novo from previously non-coding DNA.* F1000Res, 2017. **6**: p. 57. <http://www.ncbi.nlm.nih.gov/pubmed/28163910>.

11. Light, S., W. Basile, and A. Elofsson, *Orphans and new gene origination, a structural and evolutionary perspective.* Curr Opin Struct Biol, 2014. **26**: p. 73-83. <http://www.ncbi.nlm.nih.gov/pubmed/24934869>.

12. Guerzoni, D. and A. McLysaght, *De novo origins of human genes.* PLoS Genet, 2011. **7**(11): p. e1002381. <http://www.ncbi.nlm.nih.gov/pubmed/22102832>.

13. Neme, R. and D. Tautz, *Phylogenetic patterns of emergence of new genes support a model of frequent de novo evolution.* BMC Genomics, 2013. **14**: p. 117. <http://www.ncbi.nlm.nih.gov/pubmed/23433480>.

14. Yin, Y. and D. Fischer, *Identification and investigation of ORFans in the viral world.* BMC Genomics, 2008. **9**: p. 24. <http://www.ncbi.nlm.nih.gov/pubmed/18205946>.

15. Sabath, N., A. Wagner, and D. Karlin, *Evolution of viral proteins originated de novo by overprinting.* Mol Biol Evol, 2012. **29**(12): p. 3767-80. <http://www.ncbi.nlm.nih.gov/pubmed/22821011>.

16. Pavesi, A., G. Magiorkinis, and D.G. Karlin, *Viral proteins originated de novo by overprinting can be identified by codon usage: application to the "gene nursery" of Deltaretroviruses.* PLoS Comput Biol, 2013. **9**(8): p. e1003162. <http://www.ncbi.nlm.nih.gov/pubmed/23966842>.

17. Yin, Y. and D. Fischer, *On the origin of microbial ORFans: quantifying the strength of the evidence for viral lateral transfer.* BMC Evol Biol, 2006. **6**: p. 63. <http://www.ncbi.nlm.nih.gov/pubmed/16914045>.

18. Neuhaus, K., et al., *Translatomics combined with transcriptomics and proteomics reveals novel functional, recently evolved orphan genes in Escherichia coli O157:H7 (EHEC).* BMC Genomics, 2016. **17**: p. 133. <http://www.ncbi.nlm.nih.gov/pubmed/26911138>.

19. Delaye, L., et al., *The origin of a novel gene through overprinting in Escherichia coli.* BMC Evol Biol, 2008. **8**: p. 31. <http://www.ncbi.nlm.nih.gov/pubmed/18226237>.

20. Cai, J., et al., *De novo origination of a new protein-coding gene in Saccharomyces cerevisiae.* Genetics, 2008. **179**(1): p. 487-96. <http://www.ncbi.nlm.nih.gov/pubmed/18493065>.

21. Andreatta, M.E., et al., *The Recent De Novo Origin of Protein C-Termini.* Genome Biol Evol, 2015. **7**(6): p. 1686-701. <http://www.ncbi.nlm.nih.gov/pubmed/26002864>.

22. Philippsen, G.S., et al., *Distribution patterns and impact of transposable elements in genes of green algae.* Gene, 2016. **594**(1): p. 151-159. <http://www.ncbi.nlm.nih.gov/pubmed/27614292>.

23. Jakalski, M., et al., *Comparative genomic analysis of retrogene repertoire in two green algae Volvox carteri and Chlamydomonas reinhardtii.* Biol Direct, 2016. **11**: p. 35. <http://www.ncbi.nlm.nih.gov/pubmed/27487948>.

24. Yang, Z. and J. Huang, *De novo origin of new genes with introns in Plasmodium vivax.* FEBS Lett, 2011. **585**(4): p. 641-4. <http://www.ncbi.nlm.nih.gov/pubmed/21241695>.

25. Prabh, N. and C. Rodelsperger, *Are orphan genes protein-coding, prediction artifacts, or non-coding RNAs?* BMC Bioinformatics, 2016. **17**(1): p. 226. <http://www.ncbi.nlm.nih.gov/pubmed/27245157>.

26. Betran, E., K. Thornton, and M. Long, *Retroposed new genes out of the X in Drosophila.* Genome Res, 2002. **12**(12): p. 1854-9. <http://www.ncbi.nlm.nih.gov/pubmed/12466289>.

27. Zhao, L., et al., *Origin and Spread of de Novo Genes in Drosophila melanogaster Populations.* Science, 2014. <http://www.ncbi.nlm.nih.gov/pubmed/24457212>.

28. Levine, M.T., et al., *Novel genes derived from noncoding DNA in Drosophila melanogaster are frequently X-linked and exhibit testis-biased expression.* Proc Natl Acad Sci U S A, 2006. **103**(26): p. 9935-9. <http://www.ncbi.nlm.nih.gov/pubmed/16777968>.

29. Reinhardt, J.A., et al., *De novo ORFs in Drosophila are important to organismal fitness and evolved rapidly from previously non-coding sequences.* PLoS Genet, 2013. **9**(10): p. e1003860. <http://www.ncbi.nlm.nih.gov/pubmed/24146629>.

30. Wissler, L., et al., *Mechanisms and dynamics of orphan gene emergence in insect genomes.* Genome Biol Evol, 2013. **5**(2): p. 439-55. <http://www.ncbi.nlm.nih.gov/pubmed/23348040>.

31. Carlson, D.E. and M. Hedin, *Comparative transcriptomics of Entelegyne spiders (Araneae, Entelegynae), with emphasis on molecular evolution of orphan genes.* PLoS One, 2017. **12**(4): p. e0174102. <http://www.ncbi.nlm.nih.gov/pubmed/28379977>.

32. Murphy, D.N. and A. McLysaght, *De novo origin of protein-coding genes in murine rodents.* PLoS One, 2012. **7**(11): p. e48650. <http://www.ncbi.nlm.nih.gov/pubmed/23185269>.

33. Zhang, W., et al., *New genes drive the evolution of gene interaction networks in the human and mouse genomes.* Genome Biol, 2015. **16**(1): p. 202. <http://www.ncbi.nlm.nih.gov/pubmed/26424194>.

34. Toll-Riera, M., et al., *Evolution of primate orphan proteins.* Biochem Soc Trans, 2009. **37**(Pt 4): p. 778-82. <http://www.ncbi.nlm.nih.gov/pubmed/19614593>.

35. Kojima, K.K. and N. Okada, *mRNA retrotransposition coupled with 5' inversion as a possible source of new genes.* Mol Biol Evol, 2009. **26**(6): p. 1405-20. <http://www.ncbi.nlm.nih.gov/pubmed/19289598>\.

36. Li, C.Y., et al., *A human-specific de novo protein-coding gene associated with human brain functions.* PLoS Comput Biol, 2010. **6**(3): p. e1000734. <http://www.ncbi.nlm.nih.gov/pubmed/20376170>.

37. Wu, D.D., D.M. Irwin, and Y.P. Zhang, *De novo origin of human protein-coding genes.* PLoS Genet, 2011. **7**(11): p. e1002379. <http://www.ncbi.nlm.nih.gov/pubmed/22102831>.

38. Xie, C., et al., *Hominoid-specific de novo protein-coding genes originating from long non-coding RNAs.* PLoS Genet, 2012. **8**(9): p. e1002942. <http://www.ncbi.nlm.nih.gov/pubmed/23028352>.

39. Knowles, D.G. and A. McLysaght, *Recent de novo origin of human protein-coding genes.* Genome Res, 2009. **19**(10): p. 1752-9. <http://www.ncbi.nlm.nih.gov/pubmed/19726446>.

40. Toll-Riera, M. and M.M. Alba, *Emergence of novel domains in proteins.* BMC Evol Biol, 2013. **13**: p. 47. <http://www.ncbi.nlm.nih.gov/pubmed/23425224>.

41. Donoghue, M.T., et al., *Evolutionary origins of Brassicaceae specific genes in Arabidopsis thaliana.* BMC Evol Biol, 2011. **11**: p. 47. <http://www.ncbi.nlm.nih.gov/pubmed/21332978>.

42. Wu, D.D., et al., *"Out of pollen" hypothesis for origin of new genes in flowering plants: study from Arabidopsis thaliana.* Genome Biol Evol, 2014. **6**(10): p. 2822-9. <http://www.ncbi.nlm.nih.gov/pubmed/25237051>.
