# Supplementary material for: Living Organisms Author Their Read-Write Genomes in Evolution
Source: Biology (Basel). 2017 Dec 6;6(4):42. doi: 10.3390/biology6040042 (PMC5745447; doi:10.3390/biology6040042)
Supplement: Supplementary file 1 [file biology-06-00042-s001.tgz › biology-224185-supplementary & PUBMED links/biology-224185.zip/Shapiro - Living Organisms Author Their Read-Write Genomes in Evolution - Supplemental Material.Renumbered and Approved + PUBMED links/Supplementary Table S11 Origination of Novel Exons from Mobile DNA Elements.docx]

| **Supplementary Table 11. Origination of Novel Exons from Mobile DNA Elements** [[1-8](#_ENREF_1)] [[9](#_ENREF_9)] | | |
| --- | --- | --- |
| **Taxa** | **Mobile DNA Exonized** | **Reference(s)** |
| Green algae | Transposable elements | [[10](#_ENREF_10)] |
| Plants (coffee, rice, *Arabidopsis*, etc.) | Transposable elements | [[11](#_ENREF_11), [12](#_ENREF_12)] |
| Plants | Ds transposons | [[13](#_ENREF_13)] |
| Rice | Ds transposons | [[14](#_ENREF_14)] |
| Non-mammalian vertebrates and invertebrates | “…transcriptomes of vertebrates exhibit significant levels of exonization of TEs, only anecdotal cases were found in invertebrates. In vertebrates, as in mammals, the exonized TEs are mostly alternatively spliced…” | [[15](#_ENREF_15)] |
| Ancestral vertebrate | “…a more than 200-base-pair ultraconserved region, 100% identical in mammals, and 80% identical to the coelacanth SINE, contains a 31-amino-acid-residue alternatively spliced exon of the messenger RNA processing gene PCBP2…” | [[16](#_ENREF_16)] |
| Mammal | “Although…not evolutionarily related, mammalian TMPO and ZNF451…both code for splice isoforms that contain LAP2alpha domains…related to the first ORF from a DIRS1-like retrotransposon…domestication happened separately and resulted in proteins that combine retrotransposon and host protein domains. The alternative splicing of the retrotransposed sequence allowed the production of both the new and the untouched original isoforms...” | [[17](#_ENREF_17)] |
| Mammal | *MIR* retrotransposons | [[18](#_ENREF_18)] [[19](#_ENREF_19)] |
| Mouse | L1 retrotransposons; “antisense insertions results in an increased potential for exonization” | [[20](#_ENREF_20)] |
| Rat | Exonization of L1 and ERV (endogenous retrovirus) in embryonically expressed Rtdpoz-T1 and -T2 locus | [[21](#_ENREF_21)] |
| Human and mouse | “…exonization of transposed elements is biased towards the beginning of the coding sequence in both human and mouse genes…cases of primate-specific *Alu* elements that depend on RNA editing for their exonization…” | [[22-24](#_ENREF_22)] [[25](#_ENREF_25)] |
| Primate | *Alu* exonization in BCS1L, other loci | [[6](#_ENREF_6)] [[26](#_ENREF_26)] |
| Primate | RNA edited *Alu* element in human nuclear prelamin A recognition factor gene transcript | [[25](#_ENREF_25)] |
| Primate | *MIR* retrotransposon | [[27](#_ENREF_27)] |
| Primate | LINE retrotransposon in ZRANB2 locus | [[28](#_ENREF_28)] |
| Human | SINE retrotransposons | [[29](#_ENREF_29)] |
| Human | Anti-sense *Alu* elements; *Alu*-derived segments in two Bcl-family proteins. | [[30](#_ENREF_30)] [[31](#_ENREF_31), [32](#_ENREF_32)] [[33](#_ENREF_33)] |
| Human | Exons derived from *Alu* elements but also the exons from the TEs of other families were preferentially established in zinc finger (ZNF) genes.” | [[7](#_ENREF_7)] |
| Human | “Long Terminal Repeat (LTR) retrotransposons are associated with 1,057 human genes (5.8%). In 256 cases LTR retrotransposons were observed in protein-coding regions, while 50 distinct protein coding exons in 45 genes were comprised exclusively of LTR RetroTransposon Sequence (LRTS)…an alternatively spliced exon of the Interleukin 22 receptor, alpha 2 gene (IL22RA2) derived from a sequence of retrotransposon of the Mammalian apparent LTR retrotransposons (MaLR) family …hypothesize that the recruitment of the part of LTR as a novel exon…a result of a single mutation in the proto-splice site…” | [[34](#_ENREF_34)] |
| Human | “…human nuclear prelamin A recognition factor contains a primate-specific *Alu*-exon that exclusively depends on RNA editing for its exonization.” | [[35](#_ENREF_35)] |

REFERENCES

1. Sorek, R., *The birth of new exons: mechanisms and evolutionary consequences.* RNA, 2007. **13**(10): p. 1603-8. <http://www.ncbi.nlm.nih.gov/pubmed/17709368>.

2. Sorek, R., *When new exons are born.* Heredity, 2009. **103**(4): p. 279-80. <http://www.ncbi.nlm.nih.gov/pubmed/19491926>.

3. Volff, J.N., *Turning junk into gold: domestication of transposable elements and the creation of new genes in eukaryotes.* Bioessays, 2006. **28**(9): p. 913-22. <http://www.ncbi.nlm.nih.gov/pubmed/16937363>.

4. Bowen, N.J. and I.K. Jordan, *Exaptation of protein coding sequences from transposable elements.* Genome Dyn, 2007. **3**: p. 147-62. <http://www.ncbi.nlm.nih.gov/pubmed/18753790>.

5. Schmitz, J. and J. Brosius, *Exonization of transposed elements: A challenge and opportunity for evolution.* Biochimie, 2011. **93**(11): p. 1928-34. <http://www.ncbi.nlm.nih.gov/pubmed/21787833>.

6. Park, S.J., et al., *Gain of a New Exon by a Lineage-Specific Alu Element-Integration Event in the BCS1L Gene during Primate Evolution.* Mol Cells, 2015. **38**(11): p. 950-8. <http://www.ncbi.nlm.nih.gov/pubmed/26537194>.

7. Zhang, W., et al., *Inferring the expression variability of human transposable element-derived exons by linear model analysis of deep RNA sequencing data.* BMC Genomics, 2013. **14**: p. 584. <http://www.ncbi.nlm.nih.gov/pubmed/23984937>.

8. Piriyapongsa, J., et al., *Evaluating the protein coding potential of exonized transposable element sequences.* Biol Direct, 2007. **2**: p. 31. <http://www.ncbi.nlm.nih.gov/pubmed/18036258>.

9. Ponicsan, S.L., J.F. Kugel, and J.A. Goodrich, *Genomic gems: SINE RNAs regulate mRNA production.* Curr Opin Genet Dev, 2010. **20**(2): p. 149-55. <http://www.ncbi.nlm.nih.gov/pubmed/20176473>.

10. Philippsen, G.S., et al., *Distribution patterns and impact of transposable elements in genes of green algae.* Gene, 2016. **594**(1): p. 151-159. <http://www.ncbi.nlm.nih.gov/pubmed/27614292>.

11. Lopes, F.R., et al., *Transposable elements in Coffea (Gentianales: Rubiacea) transcripts and their role in the origin of protein diversity in flowering plants.* Mol Genet Genomics, 2008. **279**(4): p. 385-401. <http://www.ncbi.nlm.nih.gov/pubmed/18231813>.

12. Hoen, D.R. and T.E. Bureau, *Discovery of novel genes derived from transposable elements using integrative genomic analysis.* Mol Biol Evol, 2015. **32**(6): p. 1487-506. <http://www.ncbi.nlm.nih.gov/pubmed/25713212>.

13. Liu, L.Y. and Y.C. Charng, *Genome-wide survey of ds exonization to enrich transcriptomes and proteomes in plants.* Evol Bioinform Online, 2012. **8**: p. 575-87. <http://www.ncbi.nlm.nih.gov/pubmed/23091369>.

14. Chien, T.Y., L.Y. Liu, and Y.C. Charng, *Analysis of new functional profiles of protein isoforms yielded by ds exonization in rice.* Evol Bioinform Online, 2013. **9**: p. 417-27. <http://www.ncbi.nlm.nih.gov/pubmed/24137048>.

15. Sela, N., E. Kim, and G. Ast, *The role of transposable elements in the evolution of non-mammalian vertebrates and invertebrates.* Genome Biol, 2010. **11**(6): p. R59. <http://www.ncbi.nlm.nih.gov/pubmed/20525173>.

16. Bejerano, G., et al., *A distal enhancer and an ultraconserved exon are derived from a novel retroposon.* Nature, 2006. **441**(7089): p. 87-90. <http://www.ncbi.nlm.nih.gov/pubmed/16625209>.

17. Abascal, F., M.L. Tress, and A. Valencia, *Alternative splicing and co-option of transposable elements: the case of TMPO/LAP2alpha and ZNF451 in mammals.* Bioinformatics, 2015. **31**(14): p. 2257-61. <http://www.ncbi.nlm.nih.gov/pubmed/25735770>.

18. Annibalini, G., et al., *MIR retroposon exonization promotes evolutionary variability and generates species-specific expression of IGF-1 splice variants.* Biochim Biophys Acta, 2016. **1859**(5): p. 757-68. <http://www.ncbi.nlm.nih.gov/pubmed/27048986>.

19. Krull, M., et al., *Functional persistence of exonized mammalian-wide interspersed repeat elements (MIRs).* Genome Res, 2007. **17**(8): p. 1139-45. <http://www.ncbi.nlm.nih.gov/pubmed/17623809>.

20. Zemojtel, T., et al., *Exonization of active mouse L1s: a driver of transcriptome evolution?* BMC Genomics, 2007. **8**: p. 392. <http://www.ncbi.nlm.nih.gov/pubmed/17963496>.

21. Huang, C.J., et al., *Transcription of the rat testis-specific Rtdpoz-T1 and -T2 retrogenes during embryo development: co-transcription and frequent exonisation of transposable element sequences.* BMC Mol Biol, 2009. **10**: p. 74. <http://www.ncbi.nlm.nih.gov/pubmed/19630990>.

22. Sela, N., et al., *Characteristics of transposable element exonization within human and mouse.* PLoS One, 2010. **5**(6): p. e10907. <http://www.ncbi.nlm.nih.gov/pubmed/20532223>.

23. Mandal, A.K., et al., *Transcriptome-wide expansion of non-coding regulatory switches: evidence from co-occurrence of Alu exonization, antisense and editing.* Nucleic Acids Res, 2013. **41**(4): p. 2121-37. <http://www.ncbi.nlm.nih.gov/pubmed/23303787>.

24. Zarnack, K., et al., *Direct competition between hnRNP C and U2AF65 protects the transcriptome from the exonization of Alu elements.* Cell, 2013. **152**(3): p. 453-66. <http://www.ncbi.nlm.nih.gov/pubmed/23374342>.

25. Moller-Krull, M., et al., *Beyond DNA: RNA editing and steps toward Alu exonization in primates.* J Mol Biol, 2008. **382**(3): p. 601-9. <http://www.ncbi.nlm.nih.gov/pubmed/18680752>.

26. Krull, M., J. Brosius, and J. Schmitz, *Alu-SINE exonization: en route to protein-coding function.* Mol Biol Evol, 2005. **22**(8): p. 1702-11. <http://www.ncbi.nlm.nih.gov/pubmed/15901843>.

27. Lin, L., et al., *Large-scale analysis of exonized mammalian-wide interspersed repeats in primate genomes.* Hum Mol Genet, 2009. **18**(12): p. 2204-14. <http://www.ncbi.nlm.nih.gov/pubmed/19324900>.

28. Park, S.J., et al., *Intron Retention and TE Exonization Events in ZRANB2.* Comp Funct Genomics, 2012. **2012**: p. 170208. <http://www.ncbi.nlm.nih.gov/pubmed/22778693>.

29. Vorechovsky, I., *Transposable elements in disease-associated cryptic exons.* Hum Genet, 2010. **127**(2): p. 135-54. <http://www.ncbi.nlm.nih.gov/pubmed/19823873>.

30. Huda, A. and P.R. Bushel, *Widespread Exonization of Transposable Elements in Human Coding Sequences is Associated with Epigenetic Regulation of Transcription.* Transcr Open Access, 2013. **1**(1). <http://www.ncbi.nlm.nih.gov/pubmed/24860841>.

31. Corvelo, A. and E. Eyras, *Exon creation and establishment in human genes.* Genome Biol, 2008. **9**(9): p. R141. <http://www.ncbi.nlm.nih.gov/pubmed/18811936>.

32. Wu, M., L. Li, and Z. Sun, *Transposable element fragments in protein-coding regions and their contributions to human functional proteins.* Gene, 2007. **401**(1-2): p. 165-71. <http://www.ncbi.nlm.nih.gov/pubmed/17716834>.

33. Schwartz, S., et al., *Alu exonization events reveal features required for precise recognition of exons by the splicing machinery.* PLoS Comput Biol, 2009. **5**(3): p. e1000300. <http://www.ncbi.nlm.nih.gov/pubmed/19266014>.

34. Piriyapongsa, J., et al., *Exonization of the LTR transposable elements in human genome.* BMC Genomics, 2007. **8**: p. 291. <http://www.ncbi.nlm.nih.gov/pubmed/17725822>.

35. Lev-Maor, G., et al., *RNA-editing-mediated exon evolution.* Genome Biol, 2007. **8**(2): p. R29. <http://www.ncbi.nlm.nih.gov/pubmed/17326827>.
