# Supplementary material for: Living Organisms Author Their Read-Write Genomes in Evolution
Source: Biology (Basel). 2017 Dec 6;6(4):42. doi: 10.3390/biology6040042 (PMC5745447; doi:10.3390/biology6040042)
Supplement: Supplementary file 1 [file biology-06-00042-s001.tgz › biology-224185-supplementary & PUBMED links/biology-224185.zip/Shapiro - Living Organisms Author Their Read-Write Genomes in Evolution - Supplemental Material.Renumbered and Approved + PUBMED links/Supplementary Table S13 Distributed genome network innovation attributed to mobile DNA elements.docx]

| - **Supplementary Table 13 Distributed genome network innovation attributed to mobile DNA elements** [[1](#_ENREF_1), [2](#_ENREF_2)] | | |
| --- | --- | --- |
| **Organisms** | **Phenotypes** | **References** |
| 18 Fungal Genomes | Whole-Genome Architecture and Transcriptional Profiles | [[3](#_ENREF_3)] |
| Plants | Epigenetic Controls | [[4](#_ENREF_4)] |
| Plants | C4 photosynthesis | [[5](#_ENREF_5)] |
| Plants | Stress Response | [[6](#_ENREF_6)] |
| Maize | Abiotic Stress Response | [[7](#_ENREF_7)] |
| Maize | Helitron transposons reshuffle the transcriptome | [[8](#_ENREF_8)] |
| Cotton | Fiber cell development | [[9](#_ENREF_9)] |
| *Coffea* | Drought stress response | [[10](#_ENREF_10)] |
| *Drosophila* | X chromosome dosage compensation | [[11](#_ENREF_11)] |
| Mammals | Estrogen receptor network | [[12](#_ENREF_12)] |
| Mammals | Pregnancy | [[13](#_ENREF_13)] |
| Human | c-Myc regulatory subnetwork | [[14](#_ENREF_14)] |
| Human | Core embryonic stem cell development | [[15](#_ENREF_15)] |

REFERENCES

1. Cowley, M. and R.J. Oakey, *Transposable elements re-wire and fine-tune the transcriptome.* PLoS Genet, 2013. **9**(1): p. e1003234. <http://www.ncbi.nlm.nih.gov/pubmed/23358118>.

2. van de Lagemaat, L.N., et al., *Transposable elements in mammals promote regulatory variation and diversification of genes with specialized functions.* Trends Genet, 2003. **19**(10): p. 530-6. <http://www.ncbi.nlm.nih.gov/pubmed/14550626>.

3. Castanera, R., et al., *Transposable Elements versus the Fungal Genome: Impact on Whole-Genome Architecture and Transcriptional Profiles.* PLoS Genet, 2016. **12**(6): p. e1006108. <http://www.ncbi.nlm.nih.gov/pubmed/27294409>.

4. Bennetzen, J.L. and H. Wang, *The contributions of transposable elements to the structure, function, and evolution of plant genomes.* Annu Rev Plant Biol, 2014. **65**: p. 505-30. <http://www.ncbi.nlm.nih.gov/pubmed/24579996>.

5. Cao, C., et al., *Evidence for the role of transposons in the recruitment of cis-regulatory motifs during the evolution of C4 photosynthesis.* BMC Genomics, 2016. **17**(1): p. 201. <http://www.ncbi.nlm.nih.gov/pubmed/26955946>.

6. Negi, P., A.N. Rai, and P. Suprasanna, *Moving through the Stressed Genome: Emerging Regulatory Roles for Transposons in Plant Stress Response.* Front Plant Sci, 2016. **7**: p. 1448. <http://www.ncbi.nlm.nih.gov/pubmed/27777577>.

7. Makarevitch, I., et al., *Transposable elements contribute to activation of maize genes in response to abiotic stress.* PLoS Genet, 2015. **11**(1): p. e1004915. <http://www.ncbi.nlm.nih.gov/pubmed/25569788>.

8. Barbaglia, A.M., et al., *Gene capture by Helitron transposons reshuffles the transcriptome of maize.* Genetics, 2012. **190**(3): p. 965-75. <http://www.ncbi.nlm.nih.gov/pubmed/22174072>.

9. Wang, K., G. Huang, and Y. Zhu, *Transposable elements play an important role during cotton genome evolution and fiber cell development.* Sci China Life Sci, 2016. **59**(2): p. 112-21. <http://www.ncbi.nlm.nih.gov/pubmed/26687725>.

10. Lopes, F.R., et al., *Transcriptional activity, chromosomal distribution and expression effects of transposable elements in coffea genomes.* PLoS One, 2013. **8**(11): p. e78931. <http://www.ncbi.nlm.nih.gov/pubmed/24244387>.

11. Ellison, C.E. and D. Bachtrog, *Dosage compensation via transposable element mediated rewiring of a regulatory network.* Science, 2013. **342**(6160): p. 846-50. <http://www.ncbi.nlm.nih.gov/pubmed/24233721>.

12. Testori, A., et al., *The role of transposable elements in shaping the combinatorial interaction of transcription factors.* BMC Genomics, 2012. **13**(1): p. 400. <http://www.ncbi.nlm.nih.gov/pubmed/22897927>.

13. Lynch, V.J., et al., *Transposon-mediated rewiring of gene regulatory networks contributed to the evolution of pregnancy in mammals.* Nat Genet, 2011. **43**(11): p. 1154-9. <http://www.ncbi.nlm.nih.gov/pubmed/21946353>.

14. Wang, J., et al., *A c-Myc regulatory subnetwork from human transposable element sequences.* Mol Biosyst, 2009. **5**(12): p. 1831-9. <http://www.ncbi.nlm.nih.gov/pubmed/19763338>.

15. Kunarso, G., et al., *Transposable elements have rewired the core regulatory network of human embryonic stem cells.* Nat Genet, 2010. **42**(7): p. 631-4. <http://www.ncbi.nlm.nih.gov/pubmed/20526341>.
