# Supplementary material for: Living Organisms Author Their Read-Write Genomes in Evolution
Source: Biology (Basel). 2017 Dec 6;6(4):42. doi: 10.3390/biology6040042 (PMC5745447; doi:10.3390/biology6040042)
Supplement: Supplementary file 1 [file biology-06-00042-s001.tgz › biology-224185-supplementary & PUBMED links/biology-224185.zip/Shapiro - Living Organisms Author Their Read-Write Genomes in Evolution - Supplemental Material.Renumbered and Approved + PUBMED links/Supplementary Table S14 Diverse Regulatory Functions Reported for Long Non.docx]

| **Supplementary Table 14. Diverse Regulatory Functions Reported for Long Non-coding lncRNA molecules** | | |
| --- | --- | --- |
| **Organism** | **Function(s)** | **References** |
| Budding yeast | Galactose metabolism; controls speed of transcriptional induction by galactose | [[1](#_ENREF_1)] |
| Fission yeast | Phosphate-induced epigenetic silencing | [[2](#_ENREF_2)] |
| Budding and fission yeast | Cellular responses to environmental changes | [[3](#_ENREF_3)] |
| Fungal pathogen *Cryptococcus neoformans* | lncRNA *RZE1* regulates yeast-to-hypha transition | [[4](#_ENREF_4)] |
| *Plasmodium falciparum* | Gametocyte development | [[5](#_ENREF_5)] |
| *Plasmodium falciparum* | Telomere associated lncRNAs | [[6](#_ENREF_6)] |
| Plants | Flower development and timing | [[7](#_ENREF_7)] |
| *Arabidopsis* | Stress response lncRNAs | [[8](#_ENREF_8)] |
| *Arabidopsis thaliana* | lncRNA *TER* regulates telomerase activity | [[9](#_ENREF_9)] |
| Tomato | Fruit ripening | [[10](#_ENREF_10)] |
| *Drosophila* | Sex determination | [[11](#_ENREF_11)] |
| *Drosophila* | X chromosome dosage compensation | [[12](#_ENREF_12)] |
| Tetrapods | Spermatogenesis, synaptic transmission, placenta development | [[13](#_ENREF_13)] |
| Mammal | mRNA transcription | [[14](#_ENREF_14)] |
| Mammal | Stem cell pluripotency | [[15](#_ENREF_15)] |
| Mammal | Epigenetic chromatin formatting | [[16](#_ENREF_16)] |
| Mammal | Placental membrane integrity | [[17](#_ENREF_17)] |
| Mammal | Androgen receptor-regulated transcription | [[18](#_ENREF_18)] |
| Mammal | Synaptic connectivity in brain | [[19](#_ENREF_19)] |
| Marsupial *Monodelphis domestica* | Female X chromosome inactivation by repeat-rich lncRNA *Rsx* | [[20](#_ENREF_20)] |
| Goat (*Capra hircus*) | Skin pigmentation | [[21](#_ENREF_21)] |
| Mouse | Enhancer methylation and transcription control | [[22](#_ENREF_22)] |
| Mouse | Diurnal metabolic regulation | [[23](#_ENREF_23)] |
| Mouse | Cell reprogramming to pluripotent stem cells | [[24](#_ENREF_24)] |
| Mouse | Imprinted *Igfr2* silencing | [[25](#_ENREF_25)] |
| Mouse | Myogenesis (*SINE*-containing lncRNA acting on STAU-1 mRNA decay) | [[26](#_ENREF_26)] |
| Mouse | lncRNA *Dum* regulates myogenic differentiation and muscle regeneration | [[27](#_ENREF_27)] |
| Mouse | Translation of brain UCHL1 protein involved in preventing neurodegeneration; regulation by *SINEB2* recognition | [[28](#_ENREF_28), [29](#_ENREF_29)] |
| Mouse | lncRNA *Evf2* modulates chromatin formation in forebrain development | [[30](#_ENREF_30)] |
| Mouse | lncRNA-*HIT* mediates TGF beta-induced epithelial to mesenchymal transition in mammary epithelia and is essential for chondrogenic differentiation in the limb mesenchyme (cartilage formation) | [[31](#_ENREF_31), [32](#_ENREF_32)] |
| Mouse | lncRNA *Fendrr* regulates heart and body wall development | [[33](#_ENREF_33)] |
| Mouse | Male germline development | [[34](#_ENREF_34)] |
| Rat | Long-term potentiation of synaptic connectivity in adult brain development | [[35](#_ENREF_35)] |
| Primate | lncRNA *ANRIL* regulates expression of three cyclin-dependent kinase inhibitors and atherogenesis; *Alu* exons acquired in primate lineage | [[36](#_ENREF_36), [37](#_ENREF_37)] |
| Primate | Primate-specific lncRNA *HPAT-5* required for stem cell pluripotency | [[38](#_ENREF_38)] |
| Human | 18,871,097 lncRNA-RNA base-pairings likely involved in processing, stability control and functions of 57,303 transcripts | [[39](#_ENREF_39)] |
| Human | Centromere function | [[40](#_ENREF_40)] |
| Human | Steroid receptor activation | [[41](#_ENREF_41)] |
| Human | Endocrine regulation | [[42](#_ENREF_42)] |
| Human | Rb and p53 signaling pathways | [[43](#_ENREF_43)] |
| Human | lncRNA *RoR* is a p53 repressor in response to DNA damage; acts as microRNA sponge in transcription factor control | [[44](#_ENREF_44)] [[45](#_ENREF_45)] |
| Human | lncRNA *LED* stimulates p53 activated transcription | [[46](#_ENREF_46)] |
| Human | lncRNA *H19* modulates S-adenosylhomocysteine hydrolase and DNA methylation | [[47](#_ENREF_47)] |
| Human | lncRNA *HOTAIR* regulates chromatin dynamics | [[48](#_ENREF_48)] |
| Human | lncRNA *NBR2* regulates AMP-activated protein kinase under energy stress | [[49](#_ENREF_49)] |
| Human | *Xist* lncRNA required for X inactivation | [[50](#_ENREF_50), [51](#_ENREF_51)] |
| Human | lncRNA *XACT* in active X chromosome expression | [[52](#_ENREF_52)] |
| Human | Apoptosis and lysosomal processes (*Alu* recognition; lncRNA *GAS5*) | [[53-55](#_ENREF_53)] |
| Human | STAU-1 mRNA decay (stimulated by *Alu* pairing) | [[56](#_ENREF_56)] |
| Human | Cell cycle regulation by lncRNA *APTR* | [[57](#_ENREF_57)] |
| Human | lncRNA *Firre* controls mRNA retention in the nucleus, nucleolar anchoring of inactive X | [[58](#_ENREF_58), [59](#_ENREF_59)] |
| Human | lncRNA *ROR* regulates stem cell pluripotency; has sequences from >12 mobile elements, including a long *HERVH* 5’ sequence characteristic of several pluripotent cell lncRNAs; acts as microRNA sponge | [[60](#_ENREF_60)] [[45](#_ENREF_45), [61](#_ENREF_61), [62](#_ENREF_62)] |
| Human | Stem cell specificities | [[61](#_ENREF_61), [63](#_ENREF_63)] |
| Human | Stem cell pluripotency and cancer cell proliferation; transcription from retroviral promoters | [[64-66](#_ENREF_64)] |
| Human | Pluripotency and neuronal differentiation regulation of chromatin modifiers and transcription factors | [[67](#_ENREF_67)] |
| Human | lncRNA-mediated regulation of the interferon response | [[68](#_ENREF_68)] |
| Human | lncRNA *ANRIL* regulates inflammatory responses as a novel component of NF-kappaB pathway | [[69](#_ENREF_69)] |
| Human | Innate and adaptive immune responses | [[70-72](#_ENREF_70)] |
| Human | lncRNA *TINCR* regulates epidermal differentiation | [[73](#_ENREF_73)] |
| Human | Neurodevelopment and brain function | [[74-80](#_ENREF_74)] |
| Human | Chromatin modification, epigenetic regulation, alternative splicing, and translational control by *MALAT1*, *HOTAIR* and *TRE* lncRNAs represent important examples of lncRNA-mediated control of cell migration and invasion, epithelial-to-mesenchyme transition and metastasis | [[81](#_ENREF_81), [82](#_ENREF_82)] |

REFERENCES

1. Wang, S. and E.J. Tran, *Unexpected functions of lncRNAs in gene regulation.* Commun Integr Biol, 2013. **6**(6): p. e27610. <http://www.ncbi.nlm.nih.gov/pubmed/24563719>.

2. Shah, S., et al., *lncRNA recruits RNAi and the exosome to dynamically regulate pho1 expression in response to phosphate levels in fission yeast.* Genes Dev, 2014. **28**(3): p. 231-44. <http://www.ncbi.nlm.nih.gov/pubmed/24493644>.

3. Yamashita, A., Y. Shichino, and M. Yamamoto, *The long non-coding RNA world in yeasts.* Biochim Biophys Acta, 2016. **1859**(1): p. 147-54. <http://www.ncbi.nlm.nih.gov/pubmed/26265144>.

4. Chacko, N., et al., *The lncRNA RZE1 Controls Cryptococcal Morphological Transition.* PLoS Genet, 2015. **11**(11): p. e1005692. <http://www.ncbi.nlm.nih.gov/pubmed/26588844>.

5. Broadbent, K.M., et al., *Strand-specific RNA sequencing in Plasmodium falciparum malaria identifies developmentally regulated long non-coding RNA and circular RNA.* BMC Genomics, 2015. **16**: p. 454. <http://www.ncbi.nlm.nih.gov/pubmed/26070627>.

6. Broadbent, K.M., et al., *A global transcriptional analysis of Plasmodium falciparum malaria reveals a novel family of telomere-associated lncRNAs.* Genome Biol, 2011. **12**(6): p. R56. <http://www.ncbi.nlm.nih.gov/pubmed/21689454>.

7. Chekanova, J.A., *Long non-coding RNAs and their functions in plants.* Curr Opin Plant Biol, 2015. **27**: p. 207-16. <http://www.ncbi.nlm.nih.gov/pubmed/26342908>.

8. Di, C., et al., *Characterization of stress-responsive lncRNAs in Arabidopsis thaliana by integrating expression, epigenetic and structural features.* Plant J, 2014. **80**(5): p. 848-61. <http://www.ncbi.nlm.nih.gov/pubmed/25256571>.

9. Nelson, A.D. and D.E. Shippen, *Evolution of TERT-interacting lncRNAs: expanding the regulatory landscape of telomerase.* Front Genet, 2015. **6**: p. 277. <http://www.ncbi.nlm.nih.gov/pubmed/26442096>.

10. Zhu, B., et al., *RNA sequencing and functional analysis implicate the regulatory role of long non-coding RNAs in tomato fruit ripening.* J Exp Bot, 2015. **66**(15): p. 4483-95. <http://www.ncbi.nlm.nih.gov/pubmed/25948705>.

11. Mulvey, B.B., et al., *An interactive network of long non-coding RNAs facilitates the Drosophila sex determination decision.* Biochim Biophys Acta, 2014. **1839**(9): p. 773-84. <http://www.ncbi.nlm.nih.gov/pubmed/24954180>.

12. Quinn, J.J., et al., *Rapid evolutionary turnover underlies conserved lncRNA-genome interactions.* Genes Dev, 2016. **30**(2): p. 191-207. <http://www.ncbi.nlm.nih.gov/pubmed/26773003>.

13. Necsulea, A., et al., *The evolution of lncRNA repertoires and expression patterns in tetrapods.* Nature, 2014. **505**(7485): p. 635-40. <http://www.ncbi.nlm.nih.gov/pubmed/24463510>.

14. Kugel, J.F. and J.A. Goodrich, *The regulation of mammalian mRNA transcription by lncRNAs: recent discoveries and current concepts.* Epigenomics, 2013. **5**(1): p. 95-102. <http://www.ncbi.nlm.nih.gov/pubmed/23414324>.

15. Fort, A., et al., *Deep transcriptome profiling of mammalian stem cells supports a regulatory role for retrotransposons in pluripotency maintenance.* Nat Genet, 2014. **46**(6): p. 558-66. <http://www.ncbi.nlm.nih.gov/pubmed/24777452>.

16. Nakagawa, S. and Y. Kageyama, *Nuclear lncRNAs as epigenetic regulators-beyond skepticism.* Biochim Biophys Acta, 2014. **1839**(3): p. 215-22. <http://www.ncbi.nlm.nih.gov/pubmed/24200874>.

17. Luo, X., et al., *LncRNA pathway involved in premature preterm rupture of membrane (PPROM): an epigenomic approach to study the pathogenesis of reproductive disorders.* PLoS One, 2013. **8**(11): p. e79897. <http://www.ncbi.nlm.nih.gov/pubmed/24312190>.

18. Yang, L., et al., *lncRNA-dependent mechanisms of androgen-receptor-regulated gene activation programs.* Nature, 2013. **500**(7464): p. 598-602. <http://www.ncbi.nlm.nih.gov/pubmed/23945587>.

19. Smalheiser, N.R., *The RNA-centred view of the synapse: non-coding RNAs and synaptic plasticity.* Philos Trans R Soc Lond B Biol Sci, 2014. **369**(1652): p. pii: 20130504. <http://www.ncbi.nlm.nih.gov/pubmed/25135965>.

20. Grant, J., et al., *Rsx is a metatherian RNA with Xist-like properties in X-chromosome inactivation.* Nature, 2012. <http://www.ncbi.nlm.nih.gov/pubmed/22722828>.

21. Ren, H., et al., *Genome-wide analysis of long non-coding RNAs at early stage of skin pigmentation in goats (Capra hircus).* BMC Genomics, 2016. **17**(1): p. 67. <http://www.ncbi.nlm.nih.gov/pubmed/26785828>.

22. Berghoff, E.G., et al., *Evf2 (Dlx6as) lncRNA regulates ultraconserved enhancer methylation and the differential transcriptional control of adjacent genes.* Development, 2013. **140**(21): p. 4407-16. <http://www.ncbi.nlm.nih.gov/pubmed/24089468>.

23. Powell, W.T., et al., *A Prader-Willi locus lncRNA cloud modulates diurnal genes and energy expenditure.* Hum Mol Genet, 2013. **22**(21): p. 4318-28. <http://www.ncbi.nlm.nih.gov/pubmed/23771028>.

24. Kim, D.H., et al., *Single-cell transcriptome analysis reveals dynamic changes in lncRNA expression during reprogramming.* Cell Stem Cell, 2015. **16**(1): p. 88-101. <http://www.ncbi.nlm.nih.gov/pubmed/25575081>.

25. Santoro, F., et al., *Imprinted Igf2r silencing depends on continuous Airn lncRNA expression and is not restricted to a developmental window.* Development, 2013. **140**(6): p. 1184-95. <http://www.ncbi.nlm.nih.gov/pubmed/23444351>.

26. Wang, J., C. Gong, and L.E. Maquat, *Control of myogenesis by rodent SINE-containing lncRNAs.* Genes Dev, 2013. **27**(7): p. 793-804. <http://www.ncbi.nlm.nih.gov/pubmed/23558772>.

27. Wang, L., et al., *LncRNA Dum interacts with Dnmts to regulate Dppa2 expression during myogenic differentiation and muscle regeneration.* Cell Res, 2015. **25**(3): p. 335-50. <http://www.ncbi.nlm.nih.gov/pubmed/25686699>.

28. Carrieri, C., et al., *Long non-coding antisense RNA controls Uchl1 translation through an embedded SINEB2 repeat.* Nature, 2012. **491**(7424): p. 454-7. <http://www.ncbi.nlm.nih.gov/pubmed/23064229>.

29. Zucchelli, S., et al., *SINEUPs are modular antisense long non-coding RNAs that increase synthesis of target proteins in cells.* Front Cell Neurosci, 2015. **9**: p. 174. <http://www.ncbi.nlm.nih.gov/pubmed/26029048>.

30. Cajigas, I., et al., *Evf2 lncRNA/BRG1/DLX1 interactions reveal RNA-dependent inhibition of chromatin remodeling.* Development, 2015. **142**(15): p. 2641-52. <http://www.ncbi.nlm.nih.gov/pubmed/26138476>.

31. Richards, E.J., et al., *Long non-coding RNAs (LncRNA) regulated by transforming growth factor (TGF) beta: LncRNA-hit-mediated TGFbeta-induced epithelial to mesenchymal transition in mammary epithelia.* J Biol Chem, 2015. **290**(11): p. 6857-67. <http://www.ncbi.nlm.nih.gov/pubmed/25605728>.

32. Carlson, H.L., et al., *LncRNA-HIT Functions as an Epigenetic Regulator of Chondrogenesis through Its Recruitment of p100/CBP Complexes.* PLoS Genet, 2015. **11**(12): p. e1005680. <http://www.ncbi.nlm.nih.gov/pubmed/26633036>.

33. Grote, P., et al., *The tissue-specific lncRNA Fendrr is an essential regulator of heart and body wall development in the mouse.* Dev Cell, 2013. **24**(2): p. 206-14. <http://www.ncbi.nlm.nih.gov/pubmed/23369715>.

34. Bao, J., et al., *Expression profiling reveals developmentally regulated lncRNA repertoire in the mouse male germline.* Biol Reprod, 2013. **89**(5): p. 107. <http://www.ncbi.nlm.nih.gov/pubmed/24048575>.

35. Maag, J.L., et al., *Dynamic expression of long noncoding RNAs and repeat elements in synaptic plasticity.* Front Neurosci, 2015. **9**: p. 351. <http://www.ncbi.nlm.nih.gov/pubmed/26483626>.

36. He, S., et al., *ANRIL/CDKN2B-AS shows two-stage clade-specific evolution and becomes conserved after transposon insertions in simians.* BMC Evol Biol, 2013. **13**: p. 247. <http://www.ncbi.nlm.nih.gov/pubmed/24225082>.

37. Holdt, L.M., et al., *Alu elements in ANRIL non-coding RNA at chromosome 9p21 modulate atherogenic cell functions through trans-regulation of gene networks.* PLoS Genet, 2013. **9**(7): p. e1003588. <http://www.ncbi.nlm.nih.gov/pubmed/23861667>.

38. Durruthy-Durruthy, J., et al., *The primate-specific noncoding RNA HPAT5 regulates pluripotency during human preimplantation development and nuclear reprogramming.* Nat Genet, 2016. **48**(1): p. 44-52. <http://www.ncbi.nlm.nih.gov/pubmed/26595768>.

39. Szczesniak, M.W. and I. Makalowska, *lncRNA-RNA Interactions across the Human Transcriptome.* PLoS One, 2016. **11**(3): p. e0150353. <http://www.ncbi.nlm.nih.gov/pubmed/26930590>.

40. Quenet, D. and Y. Dalal, *A long non-coding RNA is required for targeting centromeric protein A to the human centromere.* Elife, 2014. **3**: p. e03254. <http://www.ncbi.nlm.nih.gov/pubmed/25117489>.

41. Novikova, I.V., S.P. Hennelly, and K.Y. Sanbonmatsu, *Structural architecture of the human long non-coding RNA, steroid receptor RNA activator.* Nucleic Acids Res, 2012. **40**(11): p. 5034-51. <http://www.ncbi.nlm.nih.gov/pubmed/22362738>.

42. Knoll, M., H.F. Lodish, and L. Sun, *Long non-coding RNAs as regulators of the endocrine system.* Nat Rev Endocrinol, 2015. **11**(3): p. 151-60. <http://www.ncbi.nlm.nih.gov/pubmed/25560704>.

43. Subramanian, M., M.F. Jones, and A. Lal, *Long Non-Coding RNAs Embedded in the Rb and p53 Pathways.* Cancers (Basel), 2013. **5**(4): p. 1655-75. <http://www.ncbi.nlm.nih.gov/pubmed/24305655>.

44. Zhang, A., et al., *The human long non-coding RNA-RoR is a p53 repressor in response to DNA damage.* Cell Res, 2013. **23**(3): p. 340-50. <http://www.ncbi.nlm.nih.gov/pubmed/23208419>.

45. Wang, Y., et al., *Endogenous miRNA sponge lincRNA-RoR regulates Oct4, Nanog, and Sox2 in human embryonic stem cell self-renewal.* Dev Cell, 2013. **25**(1): p. 69-80. <http://www.ncbi.nlm.nih.gov/pubmed/23541921>.

46. Leveille, N., et al., *Genome-wide profiling of p53-regulated enhancer RNAs uncovers a subset of enhancers controlled by a lncRNA.* Nat Commun, 2015. **6**: p. 6520. <http://www.ncbi.nlm.nih.gov/pubmed/25813522>.

47. Zhou, J., et al., *H19 lncRNA alters DNA methylation genome wide by regulating S-adenosylhomocysteine hydrolase.* Nat Commun, 2015. **6**: p. 10221. <http://www.ncbi.nlm.nih.gov/pubmed/26687445>.

48. Bhan, A. and S.S. Mandal, *LncRNA HOTAIR: A master regulator of chromatin dynamics and cancer.* Biochim Biophys Acta, 2015. **1856**(1): p. 151-64. <http://www.ncbi.nlm.nih.gov/pubmed/26208723>.

49. Liu, X., et al., *LncRNA NBR2 engages a metabolic checkpoint by regulating AMPK under energy stress.* Nat Cell Biol, 2016. **18**(4): p. 431-42. <http://www.ncbi.nlm.nih.gov/pubmed/26999735>.

50. Engreitz, J.M., et al., *The Xist lncRNA Exploits Three-Dimensional Genome Architecture to Spread Across the X Chromosome.* Science, 2013. <http://www.ncbi.nlm.nih.gov/pubmed/23828888>.

51. Froberg, J.E., L. Yang, and J.T. Lee, *Guided by RNAs: X-inactivation as a model for lncRNA function.* J Mol Biol, 2013. **425**(19): p. 3698-706. <http://www.ncbi.nlm.nih.gov/pubmed/23816838>.

52. Vallot, C. and C. Rougeulle, *Long non-coding RNAs and human X-chromosome regulation: a coat for the active X chromosome.* RNA Biol, 2013. **10**(8): p. 1262-5. <http://www.ncbi.nlm.nih.gov/pubmed/23948700>.

53. Mandal, A.K., et al., *Transcriptome-wide expansion of non-coding regulatory switches: evidence from co-occurrence of Alu exonization, antisense and editing.* Nucleic Acids Res, 2013. **41**(4): p. 2121-37. <http://www.ncbi.nlm.nih.gov/pubmed/23303787>.

54. Rossi, M.N. and F. Antonangeli, *LncRNAs: New Players in Apoptosis Control.* Int J Cell Biol, 2014. **2014**: p. 473857. <http://www.ncbi.nlm.nih.gov/pubmed/24627686>.

55. Pickard, M.R., M. Mourtada-Maarabouni, and G.T. Williams, *Long non-coding RNA GAS5 regulates apoptosis in prostate cancer cell lines.* Biochim Biophys Acta, 2013. **1832**(10): p. 1613-23. <http://www.ncbi.nlm.nih.gov/pubmed/23676682>.

56. Gong, C. and L.E. Maquat, *lncRNAs transactivate STAU1-mediated mRNA decay by duplexing with 3' UTRs via Alu elements.* Nature, 2011. **470**(7333): p. 284-8. <http://www.ncbi.nlm.nih.gov/pubmed/21307942>.

57. Negishi, M., et al., *A new lncRNA, APTR, associates with and represses the CDKN1A/p21 promoter by recruiting polycomb proteins.* PLoS One, 2014. **9**(4): p. e95216. <http://www.ncbi.nlm.nih.gov/pubmed/24748121>.

58. Hacisuleyman, E., et al., *Function and evolution of local repeats in the Firre locus.* Nat Commun, 2016. **7**: p. 11021. <http://www.ncbi.nlm.nih.gov/pubmed/27009974>.

59. Yang, F., et al., *The lncRNA Firre anchors the inactive X chromosome to the nucleolus by binding CTCF and maintains H3K27me3 methylation.* Genome Biol, 2015. **16**: p. 52. <http://www.ncbi.nlm.nih.gov/pubmed/25887447>.

60. Loewer, S., et al., *Large intergenic non-coding RNA-RoR modulates reprogramming of human induced pluripotent stem cells.* Nat Genet, 2010. **42**(12): p. 1113-7. <http://www.ncbi.nlm.nih.gov/pubmed/21057500>.

61. Kelley, D. and J. Rinn, *Transposable elements reveal a stem cell-specific class of long noncoding RNAs.* Genome Biol, 2012. **13**(11): p. R107. <http://www.ncbi.nlm.nih.gov/pubmed/23181609>.

62. Santoni, F.A., J. Guerra, and J. Luban, *HERV-H RNA is abundant in human embryonic stem cells and a precise marker for pluripotency.* Retrovirology, 2012. **9**: p. 111. <http://www.ncbi.nlm.nih.gov/pubmed/23253934>.

63. Hu, S. and G. Shan, *LncRNAs in Stem Cells.* Stem Cells Int, 2016. **2016**: p. 2681925. <http://www.ncbi.nlm.nih.gov/pubmed/26880946>.

64. St Laurent, G., 3rd, et al., *VlincRNAs controlled by retroviral elements are a hallmark of pluripotency and cancer.* Genome Biol, 2013. **14**(7): p. R73. <http://www.ncbi.nlm.nih.gov/pubmed/23876380>.

65. Shahryari, A., et al., *Long non-coding RNA SOX2OT: expression signature, splicing patterns, and emerging roles in pluripotency and tumorigenesis.* Front Genet, 2015. **6**: p. 196. <http://www.ncbi.nlm.nih.gov/pubmed/26136768>.

66. Ng, S.Y. and L.W. Stanton, *Long non-coding RNAs in stem cell pluripotency.* Wiley Interdiscip Rev RNA, 2013. **4**(1): p. 121-8. <http://www.ncbi.nlm.nih.gov/pubmed/23139157>.

67. Ng, S.Y., R. Johnson, and L.W. Stanton, *Human long non-coding RNAs promote pluripotency and neuronal differentiation by association with chromatin modifiers and transcription factors.* Embo J, 2012. **31**(3): p. 522-33. <http://www.ncbi.nlm.nih.gov/pubmed/22193719>.

68. Valadkhan, S. and L.S. Gunawardane, *lncRNA-mediated regulation of the interferon response.* Virus Res, 2016. **212**: p. 127-36. <http://www.ncbi.nlm.nih.gov/pubmed/26474526>.

69. Zhou, X., et al., *Long non-coding RNA ANRIL regulates inflammatory responses as a novel component of NF-kappaB pathway.* RNA Biol, 2016. **13**(1): p. 98-108. <http://www.ncbi.nlm.nih.gov/pubmed/26618242>.

70. Imamura, K. and N. Akimitsu, *Long Non-Coding RNAs Involved in Immune Responses.* Front Immunol, 2014. **5**: p. 573. <http://www.ncbi.nlm.nih.gov/pubmed/25431574>.

71. Atianand, M.K. and K.A. Fitzgerald, *Long non-coding RNAs and control of gene expression in the immune system.* Trends Mol Med, 2014. **20**(11): p. 623-31. <http://www.ncbi.nlm.nih.gov/pubmed/25262537>.

72. Aune, T.M. and C.F. Spurlock, 3rd, *Long non-coding RNAs in innate and adaptive immunity.* Virus Res, 2016. **212**: p. 146-60. <http://www.ncbi.nlm.nih.gov/pubmed/26166759>.

73. Kretz, M., et al., *Control of somatic tissue differentiation by the long non-coding RNA TINCR.* Nature, 2013. **493**(7431): p. 231-5. <http://www.ncbi.nlm.nih.gov/pubmed/23201690>.

74. D'Haene, E., et al., *Identification of long non-coding RNAs involved in neuronal development and intellectual disability.* Sci Rep, 2016. **6**: p. 28396. <http://www.ncbi.nlm.nih.gov/pubmed/27319317>.

75. Roberts, T.C., K.V. Morris, and M.J. Wood, *The role of long non-coding RNAs in neurodevelopment, brain function and neurological disease.* Philos Trans R Soc Lond B Biol Sci, 2014. **369**(1652): p. pii: 20130507. <http://www.ncbi.nlm.nih.gov/pubmed/25135968>.

76. Barry, G., et al., *The long non-coding RNA Gomafu is acutely regulated in response to neuronal activation and involved in schizophrenia-associated alternative splicing.* Mol Psychiatry, 2014. **19**(4): p. 486-94. <http://www.ncbi.nlm.nih.gov/pubmed/23628989>.

77. Liu, S.J., et al., *Single-cell analysis of long non-coding RNAs in the developing human neocortex.* Genome Biol, 2016. **17**(1): p. 67. <http://www.ncbi.nlm.nih.gov/pubmed/27081004>.

78. Aprea, J. and F. Calegari, *Long non-coding RNAs in corticogenesis: deciphering the non-coding code of the brain.* Embo J, 2015. **34**(23): p. 2865-84. <http://www.ncbi.nlm.nih.gov/pubmed/26516210>.

79. Clark, B.S. and S. Blackshaw, *Long non-coding RNA-dependent transcriptional regulation in neuronal development and disease.* Front Genet, 2014. **5**: p. 164. <http://www.ncbi.nlm.nih.gov/pubmed/24936207>.

80. Hecht, P.M., et al., *Noncoding RNA in the transcriptional landscape of human neural progenitor cell differentiation.* Front Neurosci, 2015. **9**: p. 392. <http://www.ncbi.nlm.nih.gov/pubmed/26557050>.

81. Dhamija, S. and S. Diederichs, *From junk to master regulators of invasion: lncRNA functions in migration, EMT and metastasis.* Int J Cancer, 2016. <http://www.ncbi.nlm.nih.gov/pubmed/26875870>.

82. Gupta, R.A., et al., *Long non-coding RNA HOTAIR reprograms chromatin state to promote cancer metastasis.* Nature, 2010. **464**(7291): p. 1071-6. <http://www.ncbi.nlm.nih.gov/pubmed/20393566>.
