# Supplementary material for: Living Organisms Author Their Read-Write Genomes in Evolution
Source: Biology (Basel). 2017 Dec 6;6(4):42. doi: 10.3390/biology6040042 (PMC5745447; doi:10.3390/biology6040042)
Supplement: Supplementary file 1 [file biology-06-00042-s001.tgz › biology-224185-supplementary & PUBMED links/biology-224185.zip/Shapiro - Living Organisms Author Their Read-Write Genomes in Evolution - Supplemental Material.Renumbered and Approved + PUBMED links/Supplementary Table S15 Diverse Mutagenic Natural Genetic Engineering Activities.docx]

| **Supplementary Table 15. Diverse Mutagenic Natural Genetic Engineering Activities** | | |
| --- | --- | --- |
| **Mutation Type** | **Biochemical Activity** | **References** |
| Single nucleotide substitutions | Y-family mutagenic trans-lesion DNA polymerase | [[1-4](#_ENREF_1)] |
| Frameshifts | Y-family mutagenic trans-lesion DNA polymerase | [[1-4](#_ENREF_1)] |
| Deletions (bacteria) | Y-family mutagenic trans-lesion DNA polymerase | [[5](#_ENREF_5)] |
| Deletions and translocations (often involving sequence microhomologies) | Mre11, CltP exonucleases; canonical or alternative non-homologous end-joining (NHEJ) complexes | [[6-17](#_ENREF_6)] |
| Deletions and translocations | Non-allelic homologous recombination (NAHR) between mobile DNA repeats | [[18-21](#_ENREF_18)] |
| Deletion | Elevated transcription, Topoisomerase I | [[22](#_ENREF_22)] |
| Deletion | *LINE 1*-mediated | [[23-25](#_ENREF_23)] |
| Deletion | *Alu*-*Alu* NAHR | [[18](#_ENREF_18), [26-29](#_ENREF_26)] |
| Deletion | SVA-mediated NAHR | [[30](#_ENREF_30), [31](#_ENREF_31)] |
| Deletion | sRNA-targeted deletion | [[32](#_ENREF_32)] |
| Translocations | Nonhomologous end joining or microhomology-mediated break-induced replication | [[33](#_ENREF_33)] |
| Somatic hypermutation and kataegis: multiple clustered nucleotide substitutions | AID or APOBEC cytosine deaminase | [[34-40](#_ENREF_34)] |
| Chromothripsis and complex chromosome segment insertions | Loss of p53-dependent checkpoints; replication-based mechanisms with iterative template switches; Rad51 homologous recombination; NHEJ; telomere ligation and breakage-fusion-bridge (BFB) cycle of dicentric product; premature chromosome condensation; segregation of chromosome breakage and repair in micronucleus. (Not all these processes are involved in each chromothripsis event.) | [[40-52](#_ENREF_40)] [[53-55](#_ENREF_53)] |
| Chromothripsis (chromosome shattering) | *L1*-Mediated Retrotransposition and *Alu*/*Alu* Homologous Recombination | [[56](#_ENREF_56)] |

REFERENCES

1. Napolitano, R., et al., *All three SOS-inducible DNA polymerases (Pol II, Pol IV and Pol V) are involved in induced mutagenesis.* Embo J, 2000. **19**(22): p. 6259-65. <http://www.ncbi.nlm.nih.gov/pubmed/11080171>.

2. Goodman, M.F., *Error-prone repair DNA polymerases in prokaryotes and eukaryotes.* Annu Rev Biochem, 2002. **71**: p. 17-50. <http://www.ncbi.nlm.nih.gov/pubmed/12045089>.

3. Andersson, D.I., S. Koskiniemi, and D. Hughes, *Biological roles of translesion synthesis DNA polymerases in eubacteria.* Mol Microbiol, 2010. **77**(3): p. 540-8. <http://www.ncbi.nlm.nih.gov/pubmed/20609084>.

4. Guo, C., et al., *Y-family DNA polymerases in mammalian cells.* Cell Mol Life Sci\, 2009. **66**(14): p. 2363-81. <http://www.ncbi.nlm.nih.gov/pubmed/19367366>.

5. Koskiniemi, S. and D.I. Andersson, *Translesion DNA polymerases are required for spontaneous deletion formation in Salmonella typhimurium.* Proc Natl Acad Sci U S A, 2009. **106**(25): p. 10248-53. <http://www.ncbi.nlm.nih.gov/pubmed/19525399>.

6. Zhuang, J., et al., *Exonuclease function of human Mre11 promotes deletional nonhomologous end joining.* J Biol Chem, 2009. **284**(44): p. 30565-73. <http://www.ncbi.nlm.nih.gov/pubmed/19744924>.

7. Takahashi, T., F.J. Jin, and Y. Koyama, *Nonhomologous end-joining deficiency allows large chromosomal deletions to be produced by replacement-type recombination in Aspergillus oryzae.* Fungal Genet Biol, 2009. **46**(11): p. 815-24. <http://www.ncbi.nlm.nih.gov/pubmed/19654050>.

8. Shrivastav, M., L.P. De Haro, and J.A. Nickoloff, *Regulation of DNA double-strand break repair pathway choice.* Cell Res, 2008. **18**(1): p. 134-47. <http://www.ncbi.nlm.nih.gov/pubmed/18157161>.

9. Villarreal, D.D., et al., *Microhomology directs diverse DNA break repair pathways and chromosomal translocations.* PLoS Genet, 2012. **8**(11): p. e1003026. <http://www.ncbi.nlm.nih.gov/pubmed/23144625>.

10. Ghezraoui, H., et al., *Chromosomal translocations in human cells are generated by canonical nonhomologous end-joining.* Mol Cell, 2014. **55**(6): p. 829-42. <http://www.ncbi.nlm.nih.gov/pubmed/25201414>.

11. Zhang, Y. and M. Jasin, *An essential role for CtIP in chromosomal translocation formation through an alternative end-joining pathway.* Nat Struct Mol Biol, 2011. **18**(1): p. 80-4. <http://www.ncbi.nlm.nih.gov/pubmed/21131978>.

12. Bindra, R.S., et al., *Development of an assay to measure mutagenic non-homologous end-joining repair activity in mammalian cells.* Nucleic Acids Res, 2013. **41**(11): p. e115. <http://www.ncbi.nlm.nih.gov/pubmed/23585275>.

13. Ikeda, H., K. Shiraishi, and Y. Ogata, *Illegitimate recombination mediated by double-strand break and end-joining in Escherichia coli.* Adv Biophys, 2004. **38**: p. 3-20. <http://www.ncbi.nlm.nih.gov/pubmed/15493325>.

14. Simsek, D., et al., *DNA Ligase III Promotes Alternative Nonhomologous End-Joining during Chromosomal Translocation Formation.* PLoS Genet, 2011. **7**(6): p. e1002080. <http://www.ncbi.nlm.nih.gov/pubmed/21655080>.

15. Verdin, H., et al., *Microhomology-Mediated Mechanisms Underlie Non-Recurrent Disease-Causing Microdeletions of the FOXL2 Gene or Its Regulatory Domain.* PLoS Genet, 2013. **9**(3): p. e1003358. <http://www.ncbi.nlm.nih.gov/pubmed/23516377>.

16. Vissers, L.E., et al., *Rare pathogenic microdeletions and tandem duplications are microhomology-mediated and stimulated by local genomic architecture.* Hum Mol Genet, 2009. **18**(19): p. 3579-93. <http://www.ncbi.nlm.nih.gov/pubmed/19578123>.

17. Glover, L., J. Jun, and D. Horn, *Microhomology-mediated deletion and gene conversion in African trypanosomes.* Nucleic Acids Res, 2011. **39**(4): p. 1372-80. <http://www.ncbi.nlm.nih.gov/pubmed/20965968>.

18. Sen, S.K., et al., *Human genomic deletions mediated by recombination between Alu elements.* Am J Hum Genet, 2006. **79**(1): p. 41-53. <http://www.ncbi.nlm.nih.gov/pubmed/16773564>.

19. McVean, G., *What drives recombination hotspots to repeat DNA in humans?* Philos Trans R Soc Lond B Biol Sci, 2010. **365**(1544): p. 1213-8. <http://www.ncbi.nlm.nih.gov/pubmed/20308096>.

20. Hoang, M.L., et al., *Competitive repair by naturally dispersed repetitive DNA during non-allelic homologous recombination.* PLoS Genet, 2010. **6**(12): p. e1001228. <http://www.ncbi.nlm.nih.gov/pubmed/21151956>.

21. Robberecht, C., et al., *Nonallelic homologous recombination between retrotransposable elements is a driver of de novo unbalanced translocations.* Genome Res, 2013. **23**(3): p. 411-8. <http://www.ncbi.nlm.nih.gov/pubmed/23212949>.

22. Takahashi, T., et al., *Topoisomerase 1 provokes the formation of short deletions in repeated sequences upon high transcription in Saccharomyces cerevisiae.* Proceedings of the National Academy of Sciences, 2011. **108**(2): p. 692-697. .

23. Han, K., et al., *Genomic rearrangements by LINE-1 insertion-mediated deletion in the human and chimpanzee lineages.* Nucleic Acids Res, 2005. **33**(13): p. 4040-52. <http://www.ncbi.nlm.nih.gov/pubmed/16034026>.

24. Gilbert, N., S. Lutz-Prigge, and J.V. Moran, *Genomic deletions created upon LINE-1 retrotransposition.* Cell, 2002. **110**(3): p. 315-25. <http://www.ncbi.nlm.nih.gov/pubmed/12176319>.

25. Han, K., et al., *L1 recombination-associated deletions generate human genomic variation.* Proc Natl Acad Sci U S A, 2008. **105**(49): p. 19366-71. <http://www.ncbi.nlm.nih.gov/pubmed/19036926>.

26. Franke, G., et al., *Alu-Alu recombination underlies the vast majority of large VHL germline deletions: Molecular characterization and genotype-phenotype correlations in VHL patients.* Hum Mutat, 2009. **30**(5): p. 776-86. <http://www.ncbi.nlm.nih.gov/pubmed/19280651>.

27. de Smith, A.J., et al., *Small deletion variants have stable breakpoints commonly associated with alu elements.* PLoS One, 2008. **3**(8): p. e3104. <http://www.ncbi.nlm.nih.gov/pubmed/18769679>.

28. Callinan, P.A., et al., *Alu retrotransposition-mediated deletion.* J Mol Biol, 2005. **348**(4): p. 791-800. <http://www.ncbi.nlm.nih.gov/pubmed/15843013>.

29. Morales, M.E., et al., *The contribution of alu elements to mutagenic DNA double-strand break repair.* PLoS Genet, 2015. **11**(3): p. e1005016. <http://www.ncbi.nlm.nih.gov/pubmed/25761216>.

30. Vogt, J., et al., *SVA retrotransposon insertion-associated deletion represents a novel mutational mechanism underlying large genomic copy number changes with non-recurrent breakpoints.* Genome Biol, 2014. **15**(6): p. R80. <http://www.ncbi.nlm.nih.gov/pubmed/24958239>.

31. Lee, J., et al., *Human Genomic Deletions Generated by SVA-Associated Events.* Comp Funct Genomics, 2012. **2012**: p. 807270. <http://www.ncbi.nlm.nih.gov/pubmed/22666087>.

32. Swart, E.C. and M. Nowacki, *The eukaryotic way to defend and edit genomes by sRNA-targeted DNA deletion.* Ann N Y Acad Sci, 2015. <http://www.ncbi.nlm.nih.gov/pubmed/25581723>.

33. Weckselblatt, B., K.E. Hermetz, and M.K. Rudd, *Unbalanced translocations arise from diverse mutational mechanisms including chromothripsis.* Genome Res, 2015. **25**(7): p. 937-47. <http://www.ncbi.nlm.nih.gov/pubmed/26070663>.

34. Goodman, M.F., *Better living with hyper-mutation.* Environ Mol Mutagen, 2016. **57**(6): p. 421-34. <http://www.ncbi.nlm.nih.gov/pubmed/27273795>.

35. Jaszczur, M., et al., *AID and Apobec3G haphazard deamination and mutational diversity.* Cell Mol Life Sci, 2013. **70**(17): p. 3089-108. <http://www.ncbi.nlm.nih.gov/pubmed/23178850>.

36. Peled, J.U., et al., *The biochemistry of somatic hypermutation.* Annu Rev Immunol, 2008. **26**: p. 481-511. <http://www.ncbi.nlm.nih.gov/pubmed/18304001>.

37. Sakofsky, C.J., et al., *Break-induced replication is a source of mutation clusters underlying kataegis.* Cell Rep, 2014. **7**(5): p. 1640-8. <http://www.ncbi.nlm.nih.gov/pubmed/24882007>.

38. Taylor, B.J., et al., *DNA deaminases induce break-associated mutation showers with implication of APOBEC3B and 3A in breast cancer kataegis.* Elife, 2013. **2**: p. e00534. <http://www.ncbi.nlm.nih.gov/pubmed/23599896>.

39. Lada, A.G., et al., *AID/APOBEC cytosine deaminase induces genome-wide kataegis.* Biol Direct, 2012. **7**: p. 47; discussion 47. <http://www.ncbi.nlm.nih.gov/pubmed/23249472>.

40. Maciejowski, J., et al., *Chromothripsis and Kataegis Induced by Telomere Crisis.* Cell, 2015. **163**(7): p. 1641-54. <http://www.ncbi.nlm.nih.gov/pubmed/26687355>.

41. Poot, M., *Of Simple and Complex Genome Rearrangements, Chromothripsis, Chromoanasynthesis, and Chromosome Chaos.* Mol Syndromol, 2017. **8**(3): p. 115-117. <http://www.ncbi.nlm.nih.gov/pubmed/28588432>.

42. Terzoudi, G.I., et al., *Stress induced by premature chromatin condensation triggers chromosome shattering and chromothripsis at DNA sites still replicating in micronuclei or multinucleate cells when primary nuclei enter mitosis.* Mutat Res Genet Toxicol Environ Mutagen, 2015. **793**: p. 185-98. <http://www.ncbi.nlm.nih.gov/pubmed/26520389>.

43. Kloosterman, W.P., et al., *Constitutional chromothripsis rearrangements involve clustered double-stranded DNA breaks and nonhomologous repair mechanisms.* Cell Rep, 2012. **1**(6): p. 648-55. <http://www.ncbi.nlm.nih.gov/pubmed/22813740>.

44. Poot, M., *Chromothripsis after Stumbling through DNA Replication.* Mol Syndromol, 2016. **6**(5): p. 207-9. <http://www.ncbi.nlm.nih.gov/pubmed/26997940>.

45. Crasta, K., et al., *DNA breaks and chromosome pulverization from errors in mitosis.* Nature, 2012. **482**(7383): p. 53-8. <http://www.ncbi.nlm.nih.gov/pubmed/22258507>.

46. Chiang, C., et al., *Complex reorganization and predominant non-homologous repair following chromosomal breakage in karyotypically balanced germline rearrangements and transgenic integration.* Nat Genet, 2012. **44**(4): p. 390-7, S1. <http://www.ncbi.nlm.nih.gov/pubmed/22388000>.

47. Haaf, T., et al., *Sequestration of mammalian Rad51-recombination protein into micronuclei.* J Cell Biol, 1999. **144**(1): p. 11-20. <http://www.ncbi.nlm.nih.gov/pubmed/9885240>.

48. Pellestor, F., et al., *Chromothripsis: potential origin in gametogenesis and preimplantation cell divisions. A review.* Fertil Steril, 2014. **102**(6): p. 1785-96. <http://www.ncbi.nlm.nih.gov/pubmed/25439810>.

49. Pellestor, F., et al., *[Chromothripsis, an unexpected novel form of complexity for chromosomal rearrangements].* Med Sci (Paris), 2014. **30**(3): p. 266-73. <http://www.ncbi.nlm.nih.gov/pubmed/24685217>.

50. Zhang, C.Z., et al., *Chromothripsis from DNA damage in micronuclei.* Nature, 2015. **522**(7555): p. 179-84. <http://www.ncbi.nlm.nih.gov/pubmed/26017310>.

51. Leibowitz, M.L., C.Z. Zhang, and D. Pellman, *Chromothripsis: A New Mechanism for Rapid Karyotype Evolution.* Annu Rev Genet, 2015. **49**: p. 183-211. <http://www.ncbi.nlm.nih.gov/pubmed/26442848>.

52. Storchova, Z. and W.P. Kloosterman, *The genomic characteristics and cellular origin of chromothripsis.* Curr Opin Cell Biol, 2016. **40**: p. 106-13. <http://www.ncbi.nlm.nih.gov/pubmed/27023493>.

53. Gu, S., et al., *Mechanisms for Complex Chromosomal Insertions.* PLoS Genet, 2016. **12**(11): p. e1006446. <http://www.ncbi.nlm.nih.gov/pubmed/27880765>.

54. Iliakis, G., et al., *Mechanisms of DNA double strand break repair and chromosome aberration formation.* Cytogenet Genome Res, 2004. **104**(1-4): p. 14-20. <http://www.ncbi.nlm.nih.gov/pubmed/15162010>.

55. Masset, H., et al., *A Distinct Class of Chromoanagenesis Events Characterized by Focal Copy Number Gains.* Hum Mutat, 2016. **37**(7): p. 661-8. <http://www.ncbi.nlm.nih.gov/pubmed/26936114>.

56. Nazaryan-Petersen, L., et al., *Germline Chromothripsis Driven by L1-Mediated Retrotransposition and Alu/Alu Homologous Recombination.* Hum Mutat, 2016. **37**(4): p. 385-95. <http://www.ncbi.nlm.nih.gov/pubmed/26929209>.
