# Supplementary material for: Living Organisms Author Their Read-Write Genomes in Evolution
Source: Biology (Basel). 2017 Dec 6;6(4):42. doi: 10.3390/biology6040042 (PMC5745447; doi:10.3390/biology6040042)
Supplement: Supplementary file 1 [file biology-06-00042-s001.tgz › biology-224185-supplementary & PUBMED links/biology-224185.zip/Shapiro - Living Organisms Author Their Read-Write Genomes in Evolution - Supplemental Material.Renumbered and Approved + PUBMED links/Supplementary Table S16 Ecological Factors that Stimulate Natural Genetic Engineering.docx]

| **Supplementary Table 16. Ecological Factors that Induce Mutagenic DNA Repair or Modulate NGE Responses** | | |
| --- | --- | --- |
| **Ecological Factor and NGE Effect** | **Affected Organism** | **References** |
| **Growth conditions and cellular differentiation** | | |
| Stationary phase mutagenesis regulated by ComA and ComK | *B. subtilis* | [[1](#_ENREF_1)] |
| Anaerobic growth enhances point mutations, produces different spectrum | *E. coli* | [[2](#_ENREF_2)] |
| Aging colonies, mutational hotspots, retromutation (8-oxo-guanosine, formed exclusively on the transcribed strand) | *E. coli* | [[3](#_ENREF_3)] [[4](#_ENREF_4)] |
| Nutrient-dependent mutability | *E. coli* mutator strains | [[5](#_ENREF_5)] [[6](#_ENREF_6)] |
| Adaptive selection-induced retromutation (damage only to transcribed DNA strand) | *E. coli* | [[7](#_ENREF_7)] |
| Cystic Fibrosis lung growth induces hypermutability | *P. aeruginosa* | [[8](#_ENREF_8)] |
| Phosphorus/carbon limitation increase point mutations, iron/oxygen/carbon limitation increase IS150 insertions, phosphorus limitation increases indels | *E. coli* | [[9](#_ENREF_9)] [[10](#_ENREF_10)] |
| Adenine starvation stimulates Ty1 retrotransposition | Yeast *Saccharomyces cerevisaea* | [[11](#_ENREF_11)] |
| APOBEC kataegis on actively transcribed loci | Yeast *Saccharomyces cerevisaea* | [[12](#_ENREF_12)] |
| Glucose- or phosphate-limited growth produced frequent genomic amplifications, rearrangements and novel retrotransposition. | Yeast *Saccharomyces cerevisiae* | [[13](#_ENREF_13)] |
| “Starvation leads to genome restructuring. By contrast, the frequency of point mutations is less than 2-fold greater.” | Yeast *Saccharomyces cerevisiae* | [[14](#_ENREF_14)] |
| Nitrogen starvation increases copy number variations (CNVs) | Yeast *Saccharomyces cerevisiae* | [[15](#_ENREF_15)] |
| Domestication leads to increase in repetitive DNA and retrotransposons | Maize | [[16](#_ENREF_16)] |
| Early embryogenesis activates *mPing* DNA transposition | Rice | [[17](#_ENREF_17)] |
| Plant regeneration activates chromovirus *LORE1* (ERV) retrotransposition | Model legume *Lotus japonicus* | [[18](#_ENREF_18)] |
| Neural differentiation activates L1 retrotransposition. | Rodents, humans | [[19](#_ENREF_19)] |
| Aging induces retrotransposition (effect counter-acted by calorie restriction) | Mouse germline and somatic tissue | [[20](#_ENREF_20)] |
| Early embryonic development displays a mutator state for copy number variation (CNV) of genomic duplications | Humans | [[21](#_ENREF_21)] |
| **Abiotic stresses** | | |
| UV irradiation stimulates hypermutation | *Pseudomonas aeruginosa* | [[22](#_ENREF_22)] |
| Oxidative stress induce DNA transposon non-allelic homolgous recombination (NAHR) | *Burkholderia cenocepacia* | [[23](#_ENREF_23)] |
| Cis-platin treatment hypermutation | Yeast mutants, *rad1, rad2* *S. cerevisaea* | [[24](#_ENREF_24)] |
| Copper induces expansion and contraction of CUP1 arrays encoding copper-binding protein (copy number variation, CNV) | Budding yeast *Saccharomyces cerevisaea* | [[25](#_ENREF_25)] |
| Heat shock, oxidative and copper sulphate stresses activate LTR-retrotransposons Pyret and MAGGY, DNA transposons Pot3, MINE, Mg-SINE, Grasshopper and MGLR3 | Fungal pathogen *Magnaporthe oryzae* | [[26](#_ENREF_26)] |
| Mild heat stress and UV activate mariner-Mos1 transposition | *Drosophila simulans* | [[27](#_ENREF_27)] |
| Sun exposure increases somatic mutations | Skin fibroblasts | [[28](#_ENREF_28)] [[29](#_ENREF_29)] |
| Uranium induces alternative NHEJ DSB repair processes | Embryonic zebrafish cells | [[30](#_ENREF_30)] |
| “Two mechanisms … of cadmium mutagenicity: (i) induction of reactive oxygen species (ROS); and (ii) inhibition of DNA repair.” | Various mammalian experimental systems | [[31](#_ENREF_31)] |
| Arsenic, vanadium, iron induce VL30 retrotransposition | Mouse NIH3T3 cells | [[32](#_ENREF_32)] [[33](#_ENREF_33)] [[34](#_ENREF_34)] |
| “Environmental stressors, as ionizing radiation (terrestrial, space, and UV-radiation), air pollution (including particulate matter [PM]-derived and gaseous), persistent organic pollutants, and metals” activate mobile DNA elements. | Humans | [[35](#_ENREF_35)] |
| Mercury induces *LINE1* retrotransposition | Human neuroblastoma cell line | [[36](#_ENREF_36)] |
| Heavy metals affect DSB repair: low doses of NiCl_2_ favored homologous recombination (HR) and single strand annealing (SSA), which were inhibited by higher NiCl_2_ doses. | Human U2OS osteosarcoma cell lines | [[37](#_ENREF_37)] |
| Low doses of NiCl_2_ and CdCl_2_ contributed to an increase in mutagenic deletions by *Alu*-*Alu* NAHR…cells exposed to arsenic trioxide preferentially repaired using the "error prone" non-homologous end joining (alt-NHEJ) while inhibiting repair by HR. | Human HEK 293 cells | [[37](#_ENREF_37)] |
| Etomoxir, WY-14643, and salicylamide (genotoxic drugs), Aluminum, low-level As_2_O_3_ induce LINE1 retrotransposition; copper treatment downregulated *L1* retrotransposition. | Human HepG2 cells | [[38](#_ENREF_38)] [[39](#_ENREF_39)] [[40](#_ENREF_40)] |
| Exposure to cadmium chloride and cadmium diacetate inhibits NHEJ and activates MRE11-dependent repair | Human endothelial cells | [[41](#_ENREF_41)] |
| Heat stress activates *ONSEN*, *COPIA* retrotransposition | *Brassicaceae* | [[42](#_ENREF_42)] [[43-45](#_ENREF_43)] |
| Heat stress activates *ONSEN* retrotransposition | *Arabidopsis* | [[46](#_ENREF_46)] [[47](#_ENREF_47)] |
| Climate affects DNA transposon and retrotransposon activity | *Arabidopsis* | [[48](#_ENREF_48)] [[49](#_ENREF_49)] |
| Cold, heat, hypoxic, and oxidative stresses induce mutagenesis of a long CAG repeat tract in human cells | Trinucleotide repeat mutagenesis in humans | [[50](#_ENREF_50)] [[51](#_ENREF_51)] |
| Microsatellite mutation rate is significantly greater at 26°C than at 18°C | *C. elegans* | [[52](#_ENREF_52)] |
| Hyper salinity, stressed lineages accumulate ∼100% more mutations, and these mutations exhibit a distinctive molecular mutational spectrum (specific increases in relative frequency of transversion and insertion/deletion {indel} mutations). | *A. thaliana* | [[53](#_ENREF_53)] |
| Nitric oxide modulator, sodium nitroprusside induces *Tos17* LTR retrotransposition. | Rice | [[54](#_ENREF_54)] |
| Laser irradiation stimulates DNA methylation changes and *mPing* DNA transposition | Rice | [[55](#_ENREF_55)] |
| Fungicides boscalid (respiration inhibitor), iprodione (unclear mode of action), thiophanate methyl (inhibition of microtubulin synthesis) and azoxystrobin and pyraclostrobin (quinone outside inhibitors) raised mutation rates 1.7- to 60-fold compared to neutral conditions. | Plant pathogen *Sclerotinia sclerotiorum* | [[56](#_ENREF_56)] |
| **Biotic stresses and biomolecules** | | |
| Ethanol stress induces transient hypermutator state | *E. coli* | [[57](#_ENREF_57)] |
| Food additive sepiolite stimulates antibiotic resistance plasmid transfer | *E. coli*, *S. Typhimurium*, *M. smegmatis*, and *P. aeruginosa* | [[58](#_ENREF_58)] |
| Joint action of LL-37 (antimicrobial peptide) and free iron induces mutagenesis | *P. aeruginosa* | [[59](#_ENREF_59)] |
| Antibiotics induce SOS response and conjugal DNA transfer | *V. cholera* | [[60-63](#_ENREF_60)] |
| Fluoroquinolone and norfloxacin antibiotics induced point mutations, *IS1* non-allelic homologous recombination (NAHR) deletions, *IS5* NAHR duplications (but not transpositions) | *E. coli* | [[64](#_ENREF_64)] |
| Beta-lactam antibiotics induced RpoS-dependent mutagenesis | *E. coli* | [[60](#_ENREF_60)] |
| Ciprofloxacin enhanced mutability | *E. coli* | [[65](#_ENREF_65)] [[66](#_ENREF_66)] [[67](#_ENREF_67)] |
| Subinhibitory ciprofloxacin, SOS response | *Pseudomonas aeruginosa* | [[68](#_ENREF_68), [69](#_ENREF_69)] |
| Subinhibitory concentrations of ciprofloxacin and vancomycin activate *IS256* transposition, induce SOS response; also chloramphenicol and spectinomycin | *Staphylococcus aureus* | [[70](#_ENREF_70), [71](#_ENREF_71)] |
| Antibiotic selection induces genomic duplications | *E. coli* | [[72](#_ENREF_72)] |
| Tigecycline induces hypermutation | *Acinetobacter baumannii* | [[73](#_ENREF_73)] |
| Cationic antimicrobial peptide human cathelicidin LL-37 induces mutagenesis in CF lungs | *P. aeruginosa* | [[74](#_ENREF_74)] |
| Canavanine proteotoxic stress induces mutagenesis | Yeast *S. cerevisaea* | [[75](#_ENREF_75)] |
| Cyclo(phenylalanine-proline) produced by animals, plants, bacteria and fungi... such as *Lactobacillus reuteri*, *Streptomyces* sp. AMLK‐335, *Vibrio vulnificus*, *V. cholera*, *Pseudomonas aeruginosa* and *P. putida*; induces phosphorylation of H2AX (S139) through ATM-CHK2 activation as well as DNA double strand breaks. Gene expression analysis revealed that cyclo(phenylalanine-proline) repressed a subset of genes related to reactive oxygen species (ROS) scavenging. | Human INT‐407, U2OS and Huh7 cells | [[76](#_ENREF_76)] |
| *Chlamydia trachomatis* infection produces  8-oxo-dG, DSBs | Human cervical, ovarian cells | [[77](#_ENREF_77)] |
| *N. gonorrhea* gonococcal infection causes DNA strand breaks, abolished expression of p53 and increased in expression of cyclin-dependent kinase inhibitors p21 and p27 | Human non-tumor vaginal VK2/E6E7 cells | [[78](#_ENREF_78)] |
| *H. pylori* infection is mutagenic/carcinogenic; CagA, VacA, γGT, urease, NapA proteins induce 8-oxo-G, 8-oxo-dG, AP sites, and DSBs in host DNA, mutagenic DNA damage response | Human gastric cells | [[79](#_ENREF_79)] [[80](#_ENREF_80)] [[81](#_ENREF_81)] [[77](#_ENREF_77)] |
| *Helicobacter pylori* impairs DNA mismatch repair | Human gastric epithelial cells | [[82](#_ENREF_82)] |
| *Haemophilus ducreyi* CDT (HdCDT) DNAse genotoxin induces phosphorylation of the histone H2AX as early as 1 h after intoxication and re-localization of the DNA repair complex Mre11 in HeLa cells with kinetics similar to those observed upon ionizing radiation. | HeLa cells | [[83](#_ENREF_83)] |
| *Campylobacter jejuni*, *Haemophilus ducreyi*, *Actinobacillus actinomycetemcomitans*, *Shigella dysenteriae*, *Helicobacter cinaedi*, *Helicobacter hepaticus*, *Salmonella* species CDT and CDT-like typhoid toxins induce DSBs and SSBs | Human gastric cells | [[77](#_ENREF_77)] |
| *Shigella* strains, *E. coli* strains - Shiga toxin (RNA *N*-glycosidase) produces apurinic sites, SSBs, DSBs | Human cells | [[77](#_ENREF_77)] |
| *Chlamydia trachomatis* infection induces DNA DSB damage and inhibits recruitment of the DDR proteins pATM and 53BP1 to damage sites | Human cells | [[84](#_ENREF_84)] |
| *E. coli* harboring “pks” genomic island that codes for polyketide-peptide genotoxin, Colibactin {DNA cross-linking agent}.…pks(+) *E. coli* induce transient DNA damage response, incomplete DNA repair, anaphase bridges and chromosome aberrations from breakage-fusion-bridge cycles and chromosomal instability. Exposed cells exhibited a significant increase in 6-thioguanine–resistant (*hprt* mutant) colonies and a significant increase of *tk* mutants selected with trifluorothymidine. | Cultured mouse intestinal loop epithelial cells | [[85](#_ENREF_85)] [[86](#_ENREF_86)] |
| *Escherichia coli*, *Klebsiella pneumoniae*, *Enterobacter aerogenes*, *Citrobacter koseri* colibactin genotoxin induces genome instability | Human colorectal cells | [[77](#_ENREF_77)] |
| *Neisseria gonorrhoeae*, *Neisseria meningitides* restriction endonuclease produces 8-oxo-G, DSBs | Human prostate cells | [[77](#_ENREF_77)] |
| *E. coli* depletes host cell DNA mismatch repair (MMR) proteins | Human colonic cell lines | [[87](#_ENREF_87)] |
| *P. aeruginosa* ExoS bacterial toxin is major factor involved in γH2AX induction… infection by *P. aeruginosa* activates the DSB repair machinery of the host cells. | Human immune or lung epithelial cells | [[88](#_ENREF_88)] [[89](#_ENREF_89)] |
| *Listeria monocytogenes* induces DSBs but dampens host DSB response through degradation of MRE11 exonuclease via bacterial factor LLO (pore-forming toxin listeriolysin O) | Human HeLa (CCL-2) and Jeg-3 (HTB-36) cell lines | [[90](#_ENREF_90)] [[91](#_ENREF_91)] |
| *P. syringae* pathovar *tomato* infection induces DSB formation in *Arabidopsis*… abundance of infection-induced DSBs reduced by salicylic acid | *Arabidopsis* | [[92](#_ENREF_92)] |
| Attack by the oomycete pathogen *Peronospora parasitica* stimulates somatic recombination | *Arabidopsis* | [[93](#_ENREF_93)] |
| Tobacco mosaic virus (*TMV*) or oilseed rape mosaic virus (*ORMV*) tobacco leaf infection resulted in a systemic increase in homologous recombination (HR)…a similar phenomenon occurs in *Arabidopsis thaliana* plants infected with *ORMV*. | *Arabidopsis*, tobacco | [[94](#_ENREF_94)] |
| DNA damage response induced by infection with human cytomegalovirus | Human cells | [[95](#_ENREF_95)] |
| Human T-cell lymphotropic virus 1 (*HTLV-1*) retrovirus infection causes genome instability and DNA damage, attenuation of BER, NER, MMR, HR, NHEJ repair pathways, generation of ROS | Human T-cells | [[96](#_ENREF_96)] |
| Hepatitis C virus (*HCV*) infection produces ROS and NO, reduced MMR, BER and NER, modulation of ATM pathway activity | Human cells | [[96](#_ENREF_96)] |
| *Zika* virus infection leads to P53 activation and genotoxic stress | Human neural progenitor cells | [[97](#_ENREF_97)] |
| *Bs1* Transposition detected in maize lines following barley stripe mosaic virus infection | Zea mays | [[98](#_ENREF_98)] |
| Physiological stress, induced by climate change or invasion of new habitats, disrupts epigenetic regulation and activates mobile DNA elements | Diverse organisms | [[99](#_ENREF_99)] [[100](#_ENREF_100)] [[101](#_ENREF_101)] |

REFERENCES

1. Sung, H.M. and R.E. Yasbin, *Adaptive, or stationary-phase, mutagenesis, a component of bacterial differentiation in Bacillus subtilis.* J Bacteriol, 2002. **184**(20): p. 5641-53. <http://www.ncbi.nlm.nih.gov/pubmed/12270822>.

2. Shewaramani, S., et al., *Anaerobically Grown Escherichia coli Has an Enhanced Mutation Rate and Distinct Mutational Spectra.* PLoS Genet, 2017. **13**(1): p. e1006570. <http://www.ncbi.nlm.nih.gov/pubmed/28103245>.

3. Sekowska, A., et al., *Generation of mutation hotspots in ageing bacterial colonies.* Sci Rep, 2016. **6**(1): p. 2. <http://www.ncbi.nlm.nih.gov/pubmed/28442761>.

4. Saint-Ruf, C., et al., *Massive diversification in aging colonies of Escherichia coli.* J Bacteriol, 2014. **196**(17): p. 3059-73. <http://www.ncbi.nlm.nih.gov/pubmed/24982303>.

5. Tsuru, S., et al., *Genomic confirmation of nutrient-dependent mutability of mutators in Escherichia coli.* Genes Cells, 2015. **20**(12): p. 972-81. <http://www.ncbi.nlm.nih.gov/pubmed/26414389>.

6. Ishizawa, Y., et al., *Nutrient-dependent growth defects and mutability of mutators in Escherichia coli.* Genes Cells, 2015. **20**(1): p. 68-76. <http://www.ncbi.nlm.nih.gov/pubmed/25378049>.

7. Morreall, J., et al., *Evidence for Retromutagenesis as a Mechanism for Adaptive Mutation in Escherichia coli.* PLoS Genet, 2015. **11**(8): p. e1005477. <http://www.ncbi.nlm.nih.gov/pubmed/26305558>.

8. Rodriguez-Rojas, A., A. Oliver, and J. Blazquez, *Intrinsic and environmental mutagenesis drive diversification and persistence of Pseudomonas aeruginosa in chronic lung infections.* J Infect Dis, 2012. **205**(1): p. 121-7. <http://www.ncbi.nlm.nih.gov/pubmed/22080096>.

9. Maharjan, R.P. and T. Ferenci, *A shifting mutational landscape in 6 nutritional states: Stress-induced mutagenesis as a series of distinct stress input-mutation output relationships.* PLoS Biol, 2017. **15**(6): p. e2001477. <http://www.ncbi.nlm.nih.gov/pubmed/28594817>.

10. Maharjan, R. and T. Ferenci, *Mutational signatures indicative of environmental stress in bacteria.* Mol Biol Evol, 2015. **32**(2): p. 380-91. <http://www.ncbi.nlm.nih.gov/pubmed/25389207>.

11. Servant, G., et al., *Tye7 regulates yeast Ty1 retrotransposon sense and antisense transcription in response to adenylic nucleotides stress.* Nucleic Acids Res, 2012. **40**(12): p. 5271-82. <http://www.ncbi.nlm.nih.gov/pubmed/22379133>.

12. Lada, A.G., et al., *Disruption of Transcriptional Coactivator Sub1 Leads to Genome-Wide Re-distribution of Clustered Mutations Induced by APOBEC in Active Yeast Genes.* PLoS Genet, 2015. **11**(5): p. e1005217. <http://www.ncbi.nlm.nih.gov/pubmed/25941824>.

13. Gresham, D., et al., *The repertoire and dynamics of evolutionary adaptations to controlled nutrient-limited environments in yeast.* PLoS Genet, 2008. **4**(12): p. e1000303. <http://www.ncbi.nlm.nih.gov/pubmed/19079573>.

14. Kroll, E., et al., *Starvation-associated genome restructuring can lead to reproductive isolation in yeast.* PLoS One, 2013. **8**(7): p. e66414. <http://www.ncbi.nlm.nih.gov/pubmed/23894280>.

15. Hong, J. and D. Gresham, *Molecular specificity, convergence and constraint shape adaptive evolution in nutrient-poor environments.* PLoS Genet, 2014. **10**(1): p. e1004041. <http://www.ncbi.nlm.nih.gov/pubmed/24415948>.

16. Liu, S., et al., *Unbiased K-mer Analysis Reveals Changes in Copy Number of Highly Repetitive Sequences During Maize Domestication and Improvement.* Sci Rep, 2017. **7**: p. 42444. <http://www.ncbi.nlm.nih.gov/pubmed/28186206>.

17. Teramoto, S., et al., *Early embryogenesis-specific expression of the rice transposon Ping enhances amplification of the MITE mPing.* PLoS Genet, 2014. **10**(6): p. e1004396. <http://www.ncbi.nlm.nih.gov/pubmed/24921928>.

18. Fukai, E., et al., *Derepression of the plant Chromovirus LORE1 induces germline transposition in regenerated plants.* PLoS Genet, 2010. **6**(3): p. e1000868. .

19. Richardson, S.R., S. Morell, and G.J. Faulkner, *L1 retrotransposons and somatic mosaicism in the brain.* Annu Rev Genet, 2014. **48**: p. 1-27. <http://www.ncbi.nlm.nih.gov/pubmed/25036377>.

20. De Cecco, M., et al., *Transposable elements become active and mobile in the genomes of aging mammalian somatic tissues.* Aging (Albany NY), 2013. **5**(12): p. 867-83. <http://www.ncbi.nlm.nih.gov/pubmed/24323947>.

21. Liu, P., et al., *An Organismal CNV Mutator Phenotype Restricted to Early Human Development.* Cell, 2017. **168**(5): p. 830-842 e7. <http://www.ncbi.nlm.nih.gov/pubmed/28235197>.

22. Weigand, M.R. and G.W. Sundin, *General and inducible hypermutation facilitate parallel adaptation in Pseudomonas aeruginosa despite divergent mutation spectra.* Proc Natl Acad Sci U S A, 2012. **109**(34): p. 13680-5. <http://www.ncbi.nlm.nih.gov/pubmed/22869726>.

23. Drevinek, P., et al., *Oxidative stress of Burkholderia cenocepacia induces insertion sequence-mediated genomic rearrangements that interfere with macrorestriction-based genotyping.* J Clin Microbiol, 2010. **48**(1): p. 34-40. <http://www.ncbi.nlm.nih.gov/pubmed/19889907>.

24. Segovia, R., et al., *Hypermutation signature reveals a slippage and realignment model of translesion synthesis by Rev3 polymerase in cisplatin-treated yeast.* Proc Natl Acad Sci U S A, 2017. **114**(10): p. 2663-2668. <http://www.ncbi.nlm.nih.gov/pubmed/28223526>.

25. Hull, R.M., et al., *Environmental change drives accelerated adaptation through stimulated copy number variation.* PLoS Biol, 2017. **15**(6): p. e2001333. <http://www.ncbi.nlm.nih.gov/pubmed/28654659>.

26. Chadha, S. and M. Sharma, *Transposable elements as stress adaptive capacitors induce genomic instability in fungal pathogen Magnaporthe oryzae.* PLoS One, 2014. **9**(4): p. e94415. <http://www.ncbi.nlm.nih.gov/pubmed/24709911>.

27. Jardim, S.S., et al., *Effects of heat and UV radiation on the mobilization of transposon mariner-Mos1.* Cell Stress Chaperones, 2015. **20**(5): p. 843-51. <http://www.ncbi.nlm.nih.gov/pubmed/26092118>.

28. Saini, N., et al., *The Impact of Environmental and Endogenous Damage on Somatic Mutation Load in Human Skin Fibroblasts.* PLoS Genet, 2016. **12**(10): p. e1006385. <http://www.ncbi.nlm.nih.gov/pubmed/27788131>.

29. Abyzov, A., et al., *One thousand somatic SNVs per skin fibroblast cell set baseline of mosaic mutational load with patterns that suggest proliferative origin.* Genome Res, 2017. **27**(4): p. 512-523. <http://www.ncbi.nlm.nih.gov/pubmed/28235832>.

30. Pereira, S., et al., *Genotoxicity of uranium contamination in embryonic zebrafish cells.* Aquat Toxicol, 2012. **109**: p. 11-6. <http://www.ncbi.nlm.nih.gov/pubmed/22204984>.

31. Filipic, M., T. Fatur, and M. Vudrag, *Molecular mechanisms of cadmium induced mutagenicity.* Hum Exp Toxicol, 2006. **25**(2): p. 67-77. <http://www.ncbi.nlm.nih.gov/pubmed/16539211>.

32. Markopoulos, G., et al., *Arsenic induces VL30 retrotransposition: the involvement of oxidative stress and heat-shock protein 70.* Toxicol Sci, 2013. **134**(2): p. 312-22. <http://www.ncbi.nlm.nih.gov/pubmed/23708403>.

33. Noutsopoulos, D., et al., *Vanadium induces VL30 retrotransposition at an unusually high level: a possible carcinogenesis mechanism.* J Mol Biol, 2007. **374**(1): p. 80-90. <http://www.ncbi.nlm.nih.gov/pubmed/17920077>.

34. Konisti, S., et al., *H2O2 signals via iron induction of VL30 retrotransposition correlated with cytotoxicity.* Free Radic Biol Med, 2012. **52**(10): p. 2072-81. <http://www.ncbi.nlm.nih.gov/pubmed/22542446>.

35. Miousse, I.R., et al., *Response of transposable elements to environmental stressors.* Mutat Res Rev Mutat Res, 2015. **765**: p. 19-39. <http://www.ncbi.nlm.nih.gov/pubmed/26281766>.

36. Habibi, L., et al., *Mercury specifically induces LINE-1 activity in a human neuroblastoma cell line.* Mutat Res Genet Toxicol Environ Mutagen, 2014. **759**: p. 9-20. <http://www.ncbi.nlm.nih.gov/pubmed/24240092>.

37. Morales, M.E., et al., *Heavy Metal Exposure Influences Double Strand Break DNA Repair Outcomes.* PLoS One, 2016. **11**(3): p. e0151367. <http://www.ncbi.nlm.nih.gov/pubmed/26966913>.

38. Terasaki, N., et al., *In vitro screening for compounds that enhance human L1 mobilization.* PLoS One, 2013. **8**(9): p. e74629. <http://www.ncbi.nlm.nih.gov/pubmed/24040300>.

39. Karimi, A., et al., *Exposure of hepatocellular carcinoma cells to low-level As(2)O(3) causes an extra toxicity pathway via L1 retrotransposition induction.* Toxicol Lett, 2014. **229**(1): p. 111-7. <http://www.ncbi.nlm.nih.gov/pubmed/24960058>.

40. Karimi, A., et al., *Evaluating the extent of LINE-1 mobility following exposure to heavy metals in HepG2 cells.* Biol Trace Elem Res, 2014. **160**(1): p. 143-51. <http://www.ncbi.nlm.nih.gov/pubmed/24894828>.

41. Viau, M., et al., *Cadmium inhibits non-homologous end-joining and over-activates the MRE11-dependent repair pathway.* Mutat Res, 2008. **654**(1): p. 13-21. <http://www.ncbi.nlm.nih.gov/pubmed/18539077>.

42. Ito, H., et al., *Evolution of the ONSEN retrotransposon family activated upon heat stress in Brassicaceae.* Gene, 2013. **518**(2): p. 256-61. <http://www.ncbi.nlm.nih.gov/pubmed/23370337>.

43. Ito, H., et al., *A Stress-Activated Transposon in Arabidopsis Induces Transgenerational Abscisic Acid Insensitivity.* Sci Rep, 2016. **6**: p. 23181. <http://www.ncbi.nlm.nih.gov/pubmed/26976262>.

44. Masuta, Y., et al., *Inducible Transposition of a Heat-Activated Retrotransposon in Tissue Culture.* Plant Cell Physiol, 2016. <http://www.ncbi.nlm.nih.gov/pubmed/28013279>.

45. Pietzenuk, B., et al., *Recurrent evolution of heat-responsiveness in Brassicaceae COPIA elements.* Genome Biol, 2016. **17**(1): p. 209. <http://www.ncbi.nlm.nih.gov/pubmed/27729060>.

46. Matsunaga, W., et al., *A small RNA mediated regulation of a stress-activated retrotransposon and the tissue specific transposition during the reproductive period in Arabidopsis.* Front Plant Sci, 2015. **6**: p. 48. <http://www.ncbi.nlm.nih.gov/pubmed/25709612>.

47. Cavrak, V.V., et al., *How a retrotransposon exploits the plant's heat stress response for its activation.* PLoS Genet, 2014. **10**(1): p. e1004115. <http://www.ncbi.nlm.nih.gov/pubmed/24497839>.

48. Quadrana, L., et al., *The Arabidopsis thaliana mobilome and its impact at the species level.* Elife, 2016. **5**. <http://www.ncbi.nlm.nih.gov/pubmed/27258693>.

49. Ito, H. and T. Kakutani, *Control of transposable elements in Arabidopsis thaliana.* Chromosome Res, 2014. **22**(2): p. 217-23. <http://www.ncbi.nlm.nih.gov/pubmed/24801341>.

50. Chatterjee, N., et al., *Environmental stress induces trinucleotide repeat mutagenesis in human cells.* Proc Natl Acad Sci U S A, 2015. <http://www.ncbi.nlm.nih.gov/pubmed/25775519>.

51. Chatterjee, N., et al., *Environmental Stress Induces Trinucleotide Repeat Mutagenesis in Human Cells by Alt-Nonhomologous End Joining Repair.* J Mol Biol, 2016. **428**(15): p. 2978-80. <http://www.ncbi.nlm.nih.gov/pubmed/27318194>.

52. Matsuba, C., et al., *Temperature, stress and spontaneous mutation in Caenorhabditis briggsae and Caenorhabditis elegans.* Biol Lett, 2013. **9**(1): p. 20120334. <http://www.ncbi.nlm.nih.gov/pubmed/22875817>.

53. Jiang, C., et al., *Environmentally responsive genome-wide accumulation of de novo Arabidopsis thaliana mutations and epimutations.* Genome Res, 2014. **24**(11): p. 1821-9. <http://www.ncbi.nlm.nih.gov/pubmed/25314969>.

54. Ou, X., et al., *DNA methylation changes induced in rice by exposure to high concentrations of the nitric oxide modulator, sodium nitroprusside.* Plant Mol. Biol. Rep., 2015. **33**: p. 1428–1440. .

55. Li, S., et al., *Laser Irradiation-Induced DNA Methylation Changes Are Heritable and Accompanied with Transpositional Activation of mPing in Rice.* Front Plant Sci, 2017. **8**: p. 363. <http://www.ncbi.nlm.nih.gov/pubmed/28377781>.

56. Amaradasa, B.S. and S.E. Everhart, *Effects of Sublethal Fungicides on Mutation Rates and Genomic Variation in Fungal Plant Pathogen, Sclerotinia sclerotiorum.* PLoS One, 2016. **11**(12): p. e0168079. <http://www.ncbi.nlm.nih.gov/pubmed/27959950>.

57. Swings, T., et al., *Adaptive tuning of mutation rates allows fast response to lethal stress in Escherichia coli.* Elife, 2017. **6**. <http://www.ncbi.nlm.nih.gov/pubmed/28460660>.

58. Rodriguez-Beltran, J., et al., *The animal food supplement sepiolite promotes a direct horizontal transfer of antibiotic resistance plasmids between bacterial species.* Antimicrob Agents Chemother, 2013. **57**(6): p. 2651-3. <http://www.ncbi.nlm.nih.gov/pubmed/23529735>.

59. Rodriguez-Rojas, A., O. Makarova, and J. Rolff, *Antimicrobials, stress and mutagenesis.* PLoS Pathog, 2014. **10**(10): p. e1004445. <http://www.ncbi.nlm.nih.gov/pubmed/25299705>.

60. Gutierrez, A., et al., *beta-lactam antibiotics promote bacterial mutagenesis via an RpoS-mediated reduction in replication fidelity.* Nat Commun, 2013. **4**: p. 1610. <http://www.ncbi.nlm.nih.gov/pubmed/23511474>.

61. Baharoglu, Z., E. Krin, and D. Mazel, *RpoS Plays a Central Role in the SOS Induction by Sub-Lethal Aminoglycoside Concentrations in Vibrio cholerae.* PLoS Genet, 2013. **9**(4): p. e1003421. <http://www.ncbi.nlm.nih.gov/pubmed/23613664>.

62. Baharoglu, Z. and D. Mazel, *Vibrio cholerae triggers SOS and mutagenesis in response to a wide range of antibiotics: a route towards multiresistance.* Antimicrob Agents Chemother, 2011. **55**(5): p. 2438-41. <http://www.ncbi.nlm.nih.gov/pubmed/21300836>.

63. Baharoglu, Z., D. Bikard, and D. Mazel, *Conjugative DNA transfer induces the bacterial SOS response and promotes antibiotic resistance development through integron activation.* PLoS Genet, 2010. **6**(10): p. e1001165. <http://www.ncbi.nlm.nih.gov/pubmed/20975940>.

64. Long, H., et al., *Antibiotic treatment enhances the genome-wide mutation rate of target cells.* Proc Natl Acad Sci U S A, 2016. <http://www.ncbi.nlm.nih.gov/pubmed/27091991>.

65. Jee, J., et al., *Rates and mechanisms of bacterial mutagenesis from maximum-depth sequencing.* Nature, 2016. **534**(7609): p. 693-6. <http://www.ncbi.nlm.nih.gov/pubmed/27338792>.

66. Song, L.Y., et al., *Mutational Consequences of Ciprofloxacin in Escherichia coli.* Antimicrob Agents Chemother, 2016. **60**(10): p. 6165-72. <http://www.ncbi.nlm.nih.gov/pubmed/27480851>.

67. Kohanski, M.A., M.A. DePristo, and J.J. Collins, *Sublethal antibiotic treatment leads to multidrug resistance via radical-induced mutagenesis.* Mol Cell, 2010. **37**(3): p. 311-20. <http://www.ncbi.nlm.nih.gov/pubmed/20159551>.

68. Valencia, E.Y., et al., *Ciprofloxacin-Mediated Mutagenesis Is Suppressed by Subinhibitory Concentrations of Amikacin in Pseudomonas aeruginosa.* Antimicrob Agents Chemother, 2017. **61**(3). <http://www.ncbi.nlm.nih.gov/pubmed/28031197>.

69. Zaborskyte, G., et al., *Real-Time Monitoring of nfxB Mutant Occurrence and Dynamics in Pseudomonas aeruginosa Biofilm Exposed to Subinhibitory Concentrations of Ciprofloxacin.* Antimicrob Agents Chemother, 2017. **61**(3). <http://www.ncbi.nlm.nih.gov/pubmed/27993856>.

70. Nagel, M., et al., *Influence of ciprofloxacin and vancomycin on mutation rate and transposition of IS256 in Staphylococcus aureus.* Int J Med Microbiol, 2011. **301**(3): p. 229-36. <http://www.ncbi.nlm.nih.gov/pubmed/21115395>.

71. Schreiber, F., et al., *Antibiotic-induced autoactivation of IS256 in Staphylococcus aureus.* Antimicrob Agents Chemother, 2013. **57**(12): p. 6381-4. <http://www.ncbi.nlm.nih.gov/pubmed/24080654>.

72. Laehnemann, D., et al., *Genomics of rapid adaptation to antibiotics: convergent evolution and scalable sequence amplification.* Genome Biol Evol, 2014. **6**(6): p. 1287-301. <http://www.ncbi.nlm.nih.gov/pubmed/24850796>.

73. Hammerstrom, T.G., et al., *Acinetobacter baumannii Repeatedly Evolves a Hypermutator Phenotype in Response to Tigecycline That Effectively Surveys Evolutionary Trajectories to Resistance.* PLoS One, 2015. **10**(10): p. e0140489. <http://www.ncbi.nlm.nih.gov/pubmed/26488727>.

74. Limoli, D.H., et al., *Cationic antimicrobial peptides promote microbial mutagenesis and pathoadaptation in chronic infections.* PLoS Pathog, 2014. **10**(4): p. e1004083. <http://www.ncbi.nlm.nih.gov/pubmed/24763694>.

75. Shor, E., C.A. Fox, and J.R. Broach, *The yeast environmental stress response regulates mutagenesis induced by proteotoxic stress.* PLoS Genet, 2013. **9**(8): p. e1003680. <http://www.ncbi.nlm.nih.gov/pubmed/23935537>.

76. Lee, K., et al., *Cyclo(phenylalanine-proline) induces DNA damage in mammalian cells via reactive oxygen species.* J Cell Mol Med, 2015. **19**(12): p. 2851-64. <http://www.ncbi.nlm.nih.gov/pubmed/26416514>.

77. Chumduri, C., et al., *Subversion of host genome integrity by bacterial pathogens.* Nat Rev Mol Cell Biol, 2016. <http://www.ncbi.nlm.nih.gov/pubmed/27534801>.

78. Vielfort, K., et al., *Neisseria gonorrhoeae infection causes DNA damage and affects the expression of p21, p27 and p53 in non-tumor epithelial cells.* J Cell Sci, 2013. **126**(Pt 1): p. 339-47. <http://www.ncbi.nlm.nih.gov/pubmed/23108670>.

79. Touati, E., *When bacteria become mutagenic and carcinogenic: lessons from H. pylori.* Mutat Res, 2010. **703**(1): p. 66-70. <http://www.ncbi.nlm.nih.gov/pubmed/20709622>.

80. Hanada, K., et al., *Helicobacter pylori infection introduces DNA double-strand breaks in host cells.* Infect Immun, 2014. **82**(10): p. 4182-9. <http://www.ncbi.nlm.nih.gov/pubmed/25069978>.

81. Toller, I.M., et al., *Carcinogenic bacterial pathogen Helicobacter pylori triggers DNA double-strand breaks and a DNA damage response in its host cells.* Proc Natl Acad Sci U S A, 2011. **108**(36): p. 14944-9. <http://www.ncbi.nlm.nih.gov/pubmed/21896770>.

82. Kim, J.J., et al., *Helicobacter pylori impairs DNA mismatch repair in gastric epithelial cells.* Gastroenterology, 2002. **123**(2): p. 542-53. <http://www.ncbi.nlm.nih.gov/pubmed/12145807>.

83. Li, L., et al., *The Haemophilus ducreyi cytolethal distending toxin activates sensors of DNA damage and repair complexes in proliferating and non-proliferating cells.* Cell Microbiol, 2002. **4**(2): p. 87-99. <http://www.ncbi.nlm.nih.gov/pubmed/11896765>.

84. Chumduri, C., et al., *Chlamydia infection promotes host DNA damage and proliferation but impairs the DNA damage response.* Cell Host Microbe, 2013. **13**(6): p. 746-58. <http://www.ncbi.nlm.nih.gov/pubmed/23768498>.

85. Cuevas-Ramos, G., et al., *Escherichia coli induces DNA damage in vivo and triggers genomic instability in mammalian cells.* Proc Natl Acad Sci U S A, 2010. **107**(25): p. 11537-42. <http://www.ncbi.nlm.nih.gov/pubmed/20534522>.

86. Vizcaino, M.I. and J.M. Crawford, *The colibactin warhead crosslinks DNA.* Nat Chem, 2015. **7**(5): p. 411-7. <http://www.ncbi.nlm.nih.gov/pubmed/25901819>.

87. Maddocks, O.D., K.M. Scanlon, and M.S. Donnenberg, *An Escherichia coli effector protein promotes host mutation via depletion of DNA mismatch repair proteins.* MBio, 2013. **4**(3): p. e00152-13. <http://www.ncbi.nlm.nih.gov/pubmed/23781066>.

88. Elsen, S., et al., *The opportunistic pathogen Pseudomonas aeruginosa activates the DNA double-strand break signaling and repair pathway in infected cells.* Cell Mol Life Sci, 2013. **70**(22): p. 4385-97. <http://www.ncbi.nlm.nih.gov/pubmed/23760206>.

89. Wu, M., et al., *Host DNA repair proteins in response to Pseudomonas aeruginosa in lung epithelial cells and in mice.* Infect Immun, 2011. **79**(1): p. 75-87. <http://www.ncbi.nlm.nih.gov/pubmed/20956573>.

90. Samba-Louaka, A., et al., *Listeria monocytogenes dampens the DNA damage response.* PLoS Pathog, 2014. **10**(10): p. e1004470. <http://www.ncbi.nlm.nih.gov/pubmed/25340842>.

91. Leitao, E., et al., *Listeria monocytogenes induces host DNA damage and delays the host cell cycle to promote infection.* Cell Cycle, 2014. **13**(6): p. 928-40. <http://www.ncbi.nlm.nih.gov/pubmed/24552813>.

92. Song, J. and A.F. Bent, *Microbial pathogens trigger host DNA double-strand breaks whose abundance is reduced by plant defense responses.* PLoS Pathog, 2014. **10**(4): p. e1004030. <http://www.ncbi.nlm.nih.gov/pubmed/24699527>.

93. Lucht, J.M., et al., *Pathogen stress increases somatic recombination frequency in Arabidopsis.* Nat Genet, 2002. **30**(3): p. 311-4. <http://www.ncbi.nlm.nih.gov/pubmed/11836502>.

94. Yao, Y., P. Kathiria, and I. Kovalchuk, *A systemic increase in the recombination frequency upon local infection of Arabidopsis thaliana plants with oilseed rape mosaic virus depends on plant age, the initial inoculum concentration and the time for virus replication.* Front Plant Sci, 2013. **4**: p. 61. <http://www.ncbi.nlm.nih.gov/pubmed/23519399>.

95. Xiaofei, E. and T.F. Kowalik, *The DNA damage response induced by infection with human cytomegalovirus and other viruses.* Viruses, 2014. **6**(5): p. 2155-85. <http://www.ncbi.nlm.nih.gov/pubmed/24859341>.

96. Ryan, E.L., R. Hollingworth, and R.J. Grand, *Activation of the DNA Damage Response by RNA Viruses.* Biomolecules, 2016. **6**(1): p. 2. <http://www.ncbi.nlm.nih.gov/pubmed/26751489>.

97. Ghouzzi, V.E., et al., *ZIKA virus elicits P53 activation and genotoxic stress in human neural progenitors similar to mutations involved in severe forms of genetic microcephaly and p53.* Cell Death Dis, 2016. **7**(10): p. e2440. <http://www.ncbi.nlm.nih.gov/pubmed/27787521>.

98. Grandbastien, M.A., *LTR retrotransposons, handy hitchhikers of plant regulation and stress response.* Biochim Biophys Acta, 2015. **1849**(4): p. 403-16. <http://www.ncbi.nlm.nih.gov/pubmed/25086340>.

99. Garcia Guerreiro, M.P., et al., *Distribution of the transposable elements bilbo and gypsy in original and colonizing populations of Drosophila subobscura.* BMC Evol Biol, 2008. **8**: p. 234. <http://www.ncbi.nlm.nih.gov/pubmed/18702820>.

100. Garcia Guerreiro, M.P., *What makes transposable elements move in the Drosophila genome?* Heredity (Edinb), 2012. **108**(5): p. 461-8. <http://www.ncbi.nlm.nih.gov/pubmed/21971178>.

101. Negi, P., A.N. Rai, and P. Suprasanna, *Moving through the Stressed Genome: Emerging Regulatory Roles for Transposons in Plant Stress Response.* Front Plant Sci, 2016. **7**: p. 1448. <http://www.ncbi.nlm.nih.gov/pubmed/27777577>.
