# Supplementary material for: Living Organisms Author Their Read-Write Genomes in Evolution
Source: Biology (Basel). 2017 Dec 6;6(4):42. doi: 10.3390/biology6040042 (PMC5745447; doi:10.3390/biology6040042)
Supplement: Supplementary file 1 [file biology-06-00042-s001.tgz › biology-224185-supplementary & PUBMED links/biology-224185.zip/Shapiro - Living Organisms Author Their Read-Write Genomes in Evolution - Supplemental Material.Renumbered and Approved + PUBMED links/Supplementary Table S17 Different Genome Changes Observed in Cancer Cells http.docx]

| **Supplementary Table 17. Different Genome Changes Observed in Cancer Cells (**<http://shapiro.bsd.uchicago.edu/Cancer%20Genome%20Changes.pdf>**)** | |
| --- | --- |
| **Genome Change** | **References** |
| Stress-induced mutagenic activity | [[1](#_ENREF_1)] |
| Hypermutability following loss of replication proofreading functions | [[2](#_ENREF_2)] |
| Massive genome rearrangements (“karyotype chaos”) | [[3-5](#_ENREF_3)] |
| Homology-independent rearrangements (NHEJ) | [[6](#_ENREF_6)] |
| Retrotransposon activation | [[7](#_ENREF_7)] |
| Non-canonical termination of homologous recombination | [[8](#_ENREF_8)] |
| Kataegis and somatic hypermutation | [[9-16](#_ENREF_9)] |
| Cytosine deaminase-dependent chromosome translocation | [[12](#_ENREF_12), [13](#_ENREF_13), [17](#_ENREF_17)] |
| Chromothripsis | [[18-22](#_ENREF_18)] |
| Chromothripsis linked to oncogene amplification | [[23](#_ENREF_23)] |
| Complex insertion-deletion mutations (indels) | [[24](#_ENREF_24)] |
| Tandem duplications as well as formation of “amplicons” with rearranged and amplified chromosomal segments, a.k.a. copy number variations (CNVs) | [[25-27](#_ENREF_25)] |
| Formation of amplified circular extrachromosomal DNAs | [[28](#_ENREF_28)] |
| Processed pseudogene formation | [[29](#_ENREF_29)] |
| L1 retrotransposition | [[30-33](#_ENREF_30)] |
| Extensive L1 retrotransduction of non-repetitive DNA | [[34](#_ENREF_34)] |
| Transfer of mitochondrial DNA into nuclear genome | [[35](#_ENREF_35)] |
| RAG transposase/recombinase-mediated chromosome rearrangement in immune system tumors | [[36](#_ENREF_36), [37](#_ENREF_37)] |
| Somatic hypermutation involving a reverse transcriptase-based mutator activity | [[38](#_ENREF_38)] |

REFERENCES

1. Cisneros, L., et al., *Ancient genes establish stress-induced mutation as a hallmark of cancer.* PLoS One, 2017. **12**(4): p. e0176258. <http://www.ncbi.nlm.nih.gov/pubmed/28441401>.

2. Shlien, A., et al., *Combined hereditary and somatic mutations of replication error repair genes result in rapid onset of ultra-hypermutated cancers.* Nat Genet, 2015. <http://www.ncbi.nlm.nih.gov/pubmed/25642631>.

3. Stephens, P.J., et al., *Massive genomic rearrangement acquired in a single catastrophic event during cancer development.* Cell, 2011. **144**(1): p. 27-40. <http://www.ncbi.nlm.nih.gov/pubmed/21215367>.

4. Nones, K., et al., *Genomic catastrophes frequently arise in esophageal adenocarcinoma and drive tumorigenesis.* Nat Commun, 2014. **5**: p. 5224. <http://www.ncbi.nlm.nih.gov/pubmed/25351503>.

5. Rangel, N., M. Forero-Castro, and M. Rondon-Lagos, *New Insights in the Cytogenetic Practice: Karyotypic Chaos, Non-Clonal Chromosomal Alterations and Chromosomal Instability in Human Cancer and Therapy Response.* Genes (Basel), 2017. **8**(6). <http://www.ncbi.nlm.nih.gov/pubmed/28587191>.

6. Malhotra, A., et al., *Breakpoint profiling of 64 cancer genomes reveals numerous complex rearrangements spawned by homology-independent mechanisms.* Genome Res, 2013. **23**(5): p. 762-76. <http://www.ncbi.nlm.nih.gov/pubmed/23410887>.

7. Anwar, S.L., W. Wulaningsih, and U. Lehmann, *Transposable Elements in Human Cancer: Causes and Consequences of Deregulation.* Int J Mol Sci, 2017. **18**(5). <http://www.ncbi.nlm.nih.gov/pubmed/28471386>.

8. Hartlerode, A.J., et al., *Complex Breakpoints and Template Switching Associated with Non-canonical Termination of Homologous Recombination in Mammalian Cells.* PLoS Genet, 2016. **12**(11): p. e1006410. <http://www.ncbi.nlm.nih.gov/pubmed/27832076>.

9. Nik-Zainal, S., et al., *The life history of 21 breast cancers.* Cell, 2012. **149**(5): p. 994-1007. <http://www.ncbi.nlm.nih.gov/pubmed/22608083>.

10. Stephens, P.J., et al., *The landscape of cancer genes and mutational processes in breast cancer.* Nature, 2012. **486**(7403): p. 400-4. <http://www.ncbi.nlm.nih.gov/pubmed/22722201>.

11. Nik-Zainal, S., et al., *Mutational processes molding the genomes of 21 breast cancers.* Cell, 2012. **149**(5): p. 979-93. <http://www.ncbi.nlm.nih.gov/pubmed/22608084>.

12. Kazanov, M.D., et al., *APOBEC-Induced Cancer Mutations Are Uniquely Enriched in Early-Replicating, Gene-Dense, and Active Chromatin Regions.* Cell Rep, 2015. **13**(6): p. 1103-9. <http://www.ncbi.nlm.nih.gov/pubmed/26527001>.

13. Harris, R.S., *Molecular mechanism and clinical impact of APOBEC3B-catalyzed mutagenesis in breast cancer.* Breast Cancer Res, 2015. **17**: p. 8. <http://www.ncbi.nlm.nih.gov/pubmed/25848704>.

14. Chan, K., et al., *An APOBEC3A hypermutation signature is distinguishable from the signature of background mutagenesis by APOBEC3B in human cancers.* Nat Genet, 2015. **47**(9): p. 1067-72. <http://www.ncbi.nlm.nih.gov/pubmed/26258849>.

15. Hardianti, M.S., et al., *Activation-induced cytidine deaminase expression in follicular lymphoma: association between AID expression and ongoing mutation in FL.* Leukemia, 2004. **18**(4): p. 826-31. <http://www.ncbi.nlm.nih.gov/pubmed/14990977>.

16. Supek, F. and B. Lehner, *Clustered Mutation Signatures Reveal that Error-Prone DNA Repair Targets Mutations to Active Genes.* Cell, 2017. **170**(3): p. 534-547 e23. <http://www.ncbi.nlm.nih.gov/pubmed/28753428>.

17. Robbiani, D.F. and M.C. Nussenzweig, *Chromosome translocation, B cell lymphoma, and activation-induced cytidine deaminase.* Annu Rev Pathol, 2013. **8**: p. 79-103. <http://www.ncbi.nlm.nih.gov/pubmed/22974238>.

18. Cai, H., et al., *Chromothripsis-like patterns are recurring but heterogeneously distributed features in a survey of 22,347 cancer genome screens.* BMC Genomics, 2014. **15**: p. 82. <http://www.ncbi.nlm.nih.gov/pubmed/24476156>.

19. Kloosterman, W.P., et al., *Chromothripsis is a common mechanism driving genomic rearrangements in primary and metastatic colorectal cancer.* Genome Biol, 2011. **12**(10): p. R103. <http://www.ncbi.nlm.nih.gov/pubmed/22014273>.

20. Kloosterman, W.P., J. Koster, and J.J. Molenaar, *Prevalence and clinical implications of chromothripsis in cancer genomes.* Curr Opin Oncol, 2014. **26**(1): p. 64-72. <http://www.ncbi.nlm.nih.gov/pubmed/24305569>.

21. de Pagter, M.S. and W.P. Kloosterman, *The Diverse Effects of Complex Chromosome Rearrangements and Chromothripsis in Cancer Development.* Recent Results Cancer Res, 2015. **200**: p. 165-93. <http://www.ncbi.nlm.nih.gov/pubmed/26376877>.

22. Rode, A., et al., *Chromothripsis in cancer cells: An update.* Int J Cancer, 2016. **138**(10): p. 2322-33. <http://www.ncbi.nlm.nih.gov/pubmed/26455580>.

23. Furgason, J.M., et al., *Whole genome sequence analysis links chromothripsis to EGFR, MDM2, MDM4, and CDK4 amplification in glioblastoma.* Oncoscience, 2015. **2**(7): p. 618-28. <http://www.ncbi.nlm.nih.gov/pubmed/26328271>.

24. Ye, K., et al., *Systematic discovery of complex insertions and deletions in human cancers.* Nat Med, 2015. <http://www.ncbi.nlm.nih.gov/pubmed/26657142>.

25. Inaki, K., et al., *Systems consequences of amplicon formation in human breast cancer.* Genome Res, 2014. **24**(10): p. 1559-71. <http://www.ncbi.nlm.nih.gov/pubmed/25186909>.

26. Menghi, F., et al., *The tandem duplicator phenotype as a distinct genomic configuration in cancer.* Proc Natl Acad Sci U S A, 2016. **113**(17): p. E2373-82. <http://www.ncbi.nlm.nih.gov/pubmed/27071093>.

27. Beroukhim, R., et al., *The landscape of somatic copy-number alteration across human cancers.* Nature, 2010. **463**(7283): p. 899-905. <http://www.ncbi.nlm.nih.gov/pubmed/20164920>.

28. Turner, K.M., et al., *Extrachromosomal oncogene amplification drives tumour evolution and genetic heterogeneity.* Nature, 2017. **543**(7643): p. 122-125. <http://www.ncbi.nlm.nih.gov/pubmed/28178237>.

29. Cooke, S.L., et al., *Processed pseudogenes acquired somatically during cancer development.* Nat Commun, 2014. **5**: p. 3644. <http://www.ncbi.nlm.nih.gov/pubmed/24714652>.

30. Scott, E.C. and S.E. Devine, *The Role of Somatic L1 Retrotransposition in Human Cancers.* Viruses, 2017. **9**(6). <http://www.ncbi.nlm.nih.gov/pubmed/28561751>.

31. Helman, E., et al., *Somatic retrotransposition in human cancer revealed by whole-genome and exome sequencing.* Genome Res, 2014. **24**(7): p. 1053-63. <http://www.ncbi.nlm.nih.gov/pubmed/24823667>.

32. Lee, E., et al., *Landscape of somatic retrotransposition in human cancers.* Science, 2012. **337**(6097): p. 967-71. <http://www.ncbi.nlm.nih.gov/pubmed/22745252>.

33. Kemp, J.R. and M.S. Longworth, *Crossing the LINE Toward Genomic Instability: LINE-1 Retrotransposition in Cancer.* Front Chem, 2015. **3**: p. 68. <http://www.ncbi.nlm.nih.gov/pubmed/26734601>.

34. Tubio, J.M., et al., *Mobile DNA in cancer. Extensive transduction of nonrepetitive DNA mediated by L1 retrotransposition in cancer genomes.* Science, 2014. **345**(6196): p. 1251343. <http://www.ncbi.nlm.nih.gov/pubmed/25082706>.

35. Ju, Y.S., et al., *Frequent somatic transfer of mitochondrial DNA into the nuclear genome of human cancer cells.* Genome Res, 2015. **25**(6): p. 814-24. <http://www.ncbi.nlm.nih.gov/pubmed/25963125>.

36. Papaemmanuil, E., et al., *RAG-mediated recombination is the predominant driver of oncogenic rearrangement in ETV6-RUNX1 acute lymphoblastic leukemia.* Nat Genet, 2014. **46**(2): p. 116-25. <http://www.ncbi.nlm.nih.gov/pubmed/24413735>.

37. Halper-Stromberg, E., et al., *Fine mapping of V(D)J recombinase mediated rearrangements in human lymphoid malignancies.* BMC Genomics, 2013. **14**: p. 565. <http://www.ncbi.nlm.nih.gov/pubmed/23957733>.

38. Steele, E.J., *Somatic hypermutation in immunity and cancer: Critical analysis of strand-biased and codon-context mutation signatures.* DNA Repair (Amst), 2016. **45**: p. 1-24. <http://www.ncbi.nlm.nih.gov/pubmed/27449479>.
