# Supplementary material for: Living Organisms Author Their Read-Write Genomes in Evolution
Source: Biology (Basel). 2017 Dec 6;6(4):42. doi: 10.3390/biology6040042 (PMC5745447; doi:10.3390/biology6040042)
Supplement: Supplementary file 1 [file biology-06-00042-s001.tgz › biology-224185-supplementary & PUBMED links/biology-224185.zip/Shapiro - Living Organisms Author Their Read-Write Genomes in Evolution - Supplemental Material.Renumbered and Approved + PUBMED links/Supplementary Table S3 Photosynthetic eukaryotic lineages resulting from symbiogenesis.docx]

| **Supplementary Table 3. Photosynthetic eukaryotic lineages resulting from symbiogenesis** [[1-6](#_ENREF_1)] | |
| --- | --- |
| **Taxonomic Group** | **Symbiogenetic origin** |
| *Archaeaplastida* |  |
| Green algae (*Chlorophyta*) | Primary cyanobacterial endosymbiosis |
| *Glaucophytes* (order *Chlorococcales*) | Primary cyanobacterial endosymbiosis |
| Red algae (*Rhodophyta*) | Primary cyanobacterial endosymbiosis |
| Land plants (*Embryophyta*) | Primary cyanobacterial endosymbiosis |
|  | |
| Euglyphid amoeba *Paulinella chromatophora* | Primary cyanobacterial endosymbiosis (*Synechococcus*-*Prochloron* clade) [[7-9](#_ENREF_7)] |
|  | |
| *Euglenids* (flagellated algae) | Secondary green algal endosymbiosis |
| *Chlorarachniophytes* (marine algae) | Secondary green algal endosymbiosis |
| *Chromalveolates* (multiple lineages including organisms responsible for a large fraction of atmospheric oxygen, such as brown algae, coccolithotrphs, cryptophytes and diatoms) | Secondary red algal endosymbiosis |
|  | |
| *Dinoflagellates* (flagellated marine and fresh water protists) | Tertiary *chromalveolate* endosymbiosis or serial green or red alga endosymbioses [[10-12](#_ENREF_10)] |
| *Warwowiid* dinoflagellates with camera eye-like “ocelloids” formed by serial symbiogenetic events | [[13](#_ENREF_13)], [[14](#_ENREF_14), [15](#_ENREF_15)] |

REFERENCES

1. Embley, T.M. and W. Martin, *Eukaryotic evolution, changes and challenges.* Nature, 2006. **440**(7084): p. 623-30. <http://www.ncbi.nlm.nih.gov/pubmed/16572163>.

2. Keeling, P.J., *The endosymbiotic origin, diversification and fate of plastids.* Philos Trans R Soc Lond B Biol Sci, 2010. **365**(1541): p. 729-48. <http://www.ncbi.nlm.nih.gov/pubmed/20124341>.

3. Meheust, R., et al., *Protein networks identify novel symbiogenetic genes resulting from plastid endosymbiosis.* Proc Natl Acad Sci U S A, 2016. **113**(13): p. 3579-84. <http://www.ncbi.nlm.nih.gov/pubmed/26976593>.

4. Moreira, D. and P. Deschamps, *What was the real contribution of endosymbionts to the eukaryotic nucleus? Insights from photosynthetic eukaryotes.* Cold Spring Harb Perspect Biol, 2014. **6**(7): p. a016014. <http://www.ncbi.nlm.nih.gov/pubmed/24984774>.

5. Harold, F.M., *In Search of Cell History: The Evolution of Life's Building Blocks*2014, Chicago: University Of Chicago Press. .

6. Bhattacharya, D., et al., *Genome of the red alga Porphyridium purpureum.* Nat Commun, 2013. **4**: p. 1941. <http://www.ncbi.nlm.nih.gov/pubmed/23770768>.

7. Nowack, E.C. and A.R. Grossman, *Trafficking of protein into the recently established photosynthetic organelles of Paulinella chromatophora.* Proc Natl Acad Sci U S A, 2012. **109**(14): p. 5340-5. <http://www.ncbi.nlm.nih.gov/pubmed/22371600>.

8. Mackiewicz, P., A. Bodyl, and P. Gagat, *Possible import routes of proteins into the cyanobacterial endosymbionts/plastids of Paulinella chromatophora.* Theory Biosci, 2012. **131**(1): p. 1-18. <http://www.ncbi.nlm.nih.gov/pubmed/22209953>.

9. McFadden, G.I., *Origin and evolution of plastids and photosynthesis in eukaryotes.* Cold Spring Harb Perspect Biol, 2014. **6**(4): p. a016105. <http://www.ncbi.nlm.nih.gov/pubmed/24691960>.

10. Dorrell, R.G. and C.J. Howe, *Integration of plastids with their hosts: Lessons learned from dinoflagellates.* Proc Natl Acad Sci U S A, 2015. **112**(33): p. 10247-54. <http://www.ncbi.nlm.nih.gov/pubmed/25995366>.

11. Hackett, J.D., et al., *Dinoflagellates: a remarkable evolutionary experiment.* Am J Bot, 2004. **91**(10): p. 1523-34. <http://www.ncbi.nlm.nih.gov/pubmed/21652307>.

12. Yoon, H.S., et al., *Tertiary endosymbiosis driven genome evolution in dinoflagellate algae.* Mol Biol Evol, 2005. **22**(5): p. 1299-308. <http://www.ncbi.nlm.nih.gov/pubmed/15746017>.

13. Gavelis, G.S., et al., *Eye-like ocelloids are built from different endosymbiotically acquired components.* Nature, 2015. **523**(7559): p. 204-7. <http://www.ncbi.nlm.nih.gov/pubmed/26131935>.

14. Hayakawa, S., et al., *Function and evolutionary origin of unicellular camera-type eye structure.* PLoS One, 2015. **10**(3): p. e0118415. <http://www.ncbi.nlm.nih.gov/pubmed/25734540>.

15. Gomez, F., P. Lopez-Garcia, and D. Moreira, *Molecular phylogeny of the ocelloid-bearing dinoflagellates erythropsidinium and warnowia (warnowiaceae, dinophyceae).* J Eukaryot Microbiol, 2009. **56**(5): p. 440-5. <http://www.ncbi.nlm.nih.gov/pubmed/19737196>.
