# Supplementary material for: Living Organisms Author Their Read-Write Genomes in Evolution
Source: Biology (Basel). 2017 Dec 6;6(4):42. doi: 10.3390/biology6040042 (PMC5745447; doi:10.3390/biology6040042)
Supplement: Supplementary file 1 [file biology-06-00042-s001.tgz › biology-224185-supplementary & PUBMED links/biology-224185.zip/Shapiro - Living Organisms Author Their Read-Write Genomes in Evolution - Supplemental Material.Renumbered and Approved + PUBMED links/Supplementary Table S4 Selected Examples of Speciation and Adaptive Radiation.docx]

| **Supplementary Table 4. Selected Examples of Speciation and Adaptive Radiation Involving Interspecific Hybridization and Changes in Chromosome Number (*cf.* [**[**1-3**](#_ENREF_1)**])** | |
| --- | --- |
| **Taxon** | **References** |
| **Fungi** | [[4](#_ENREF_4)] |
| *Saccharomyces* yeast | [[5-16](#_ENREF_5)] |
| **Ciliated protists** | [[17](#_ENREF_17)] |
| **Plants** | [[18-24](#_ENREF_18)] [[25](#_ENREF_25)] |
| *Tragopogon (Asteraceae)* | [[26](#_ENREF_26), [27](#_ENREF_27)] |
| *Pinus densata* | [[28](#_ENREF_28)] |
| *Primula* | [[29](#_ENREF_29)] |
| Sunflowers (*Helianthus anomalus*) | [[30-32](#_ENREF_30)] |
| Irises (*Iris fulva, I. hexagona, and I. nelsonii*) | [[33-35](#_ENREF_33)] |
| *Nicotiana* (*Solanaceae*) | [[36-38](#_ENREF_36)] |
| *Orchidaceae* | [[39](#_ENREF_39), [40](#_ENREF_40)] |
| *Brassica napus* | [[41-43](#_ENREF_41)] |
| *Arabidopsis* | [[44-47](#_ENREF_44)] |
| Potatoes (*Solanum stoloniferum* and *S. hjertingii*) | [[48-50](#_ENREF_48)] |
| Wheats (*Aegilops-Triticum* group) | [[51-56](#_ENREF_51)] |
| Cotton (*Gossypium*) | [[57](#_ENREF_57), [58](#_ENREF_58)] |
| **Animals** | [[59-61](#_ENREF_59)] |
| Tephritid fruitflies | [[62](#_ENREF_62), [63](#_ENREF_63)] |
| Mosquitoes | [[64-66](#_ENREF_64)] |
| Tiger Swallowtail Butterflies | [[67-69](#_ENREF_67)] |
| *Heliconius* butterflies | [[70-79](#_ENREF_70)] |
| Ants | [[80-84](#_ENREF_80)] |
| Sculpins (*Cottus* sp., *Teleostei*) | [[85](#_ENREF_85), [86](#_ENREF_86)] |
| Sailfin silversides (*Teleostei*) | [[87](#_ENREF_87), [88](#_ENREF_88)] |
| East African *Cichlids* | [[40](#_ENREF_40), [89-97](#_ENREF_89)] |
| Sparrows | [[98-100](#_ENREF_98)] |
| Yellow-rumped (Audubon’s) warbler | [[101-105](#_ENREF_101)] |
| Galapagos finches (*Geospiza*) | [[3](#_ENREF_3), [106-113](#_ENREF_106)] |
| Clymene dolphin (*Delphininae (Cetacea, Mammalia)*) | [[114](#_ENREF_114), [115](#_ENREF_115)] |
| Bats | [[116](#_ENREF_116), [117](#_ENREF_117)] |
| Chinese hares (genus *Lepus*) | [[118](#_ENREF_118), [119](#_ENREF_119)] |
| Cats (*Felidae*) | [[120](#_ENREF_120)] |
| Colobine monkeys | [[121](#_ENREF_121)] |
| Southern African baboons | [[122-126](#_ENREF_122)] |

REFERENCES

1. Abbott, R., et al., *Hybridization and speciation.* J Evol Biol, 2013. **26**(2): p. 229-46. <http://www.ncbi.nlm.nih.gov/pubmed/23323997>.

2. Pennisi, E., *Shaking up the Tree of Life.* Science, 2016. **354**(6314): p. 817-821. <http://www.ncbi.nlm.nih.gov/pubmed/27856860>.

3. Grant, B.R. and P.R. Grant, *Watching speciation in action.* Science, 2017. **355**(6328): p. 910-911. <http://www.ncbi.nlm.nih.gov/pubmed/28254901>.

4. Albertin, W. and P. Marullo, *Polyploidy in fungi: evolution after whole-genome duplication.* Proc Biol Sci, 2012. **279**(1738): p. 2497-509. <http://www.ncbi.nlm.nih.gov/pubmed/22492065>.

5. Greig, D., et al., *Hybrid speciation in experimental populations of yeast.* Science, 2002. **298**(5599): p. 1773-5. <http://www.ncbi.nlm.nih.gov/pubmed/12459586>.

6. Boynton, P.J. and D. Greig, *The ecology and evolution of non-domesticated Saccharomyces species.* Yeast, 2014. **31**(12): p. 449-62. <http://www.ncbi.nlm.nih.gov/pubmed/25242436>.

7. Stelkens, R.B., et al., *Hybridization facilitates evolutionary rescue.* Evol Appl, 2014. **7**(10): p. 1209-17. <http://www.ncbi.nlm.nih.gov/pubmed/25558281>.

8. Muller, L.A. and J.H. McCusker, *A multispecies-based taxonomic microarray reveals interspecies hybridization and introgression in Saccharomyces cerevisiae.* FEMS Yeast Res, 2009. **9**(1): p. 143-52. <http://www.ncbi.nlm.nih.gov/pubmed/19054123>.

9. Sipiczki, M., *Interspecies hybridization and recombination in Saccharomyces wine yeasts.* FEMS Yeast Res, 2008. **8**(7): p. 996-1007. <http://www.ncbi.nlm.nih.gov/pubmed/18355270>.

10. Wolfe, K.H., *Origin of the Yeast Whole-Genome Duplication.* PLoS Biol, 2015. **13**(8): p. e1002221. <http://www.ncbi.nlm.nih.gov/pubmed/26252643>.

11. Marcet-Houben, M. and T. Gabaldon, *Beyond the Whole-Genome Duplication: Phylogenetic Evidence for an Ancient Interspecies Hybridization in the Baker's Yeast Lineage.* PLoS Biol, 2015. **13**(8): p. e1002220. <http://www.ncbi.nlm.nih.gov/pubmed/26252497>.

12. da Silva, T., et al., *Hybridization within Saccharomyces Genus Results in Homoeostasis and Phenotypic Novelty in Winemaking Conditions.* PLoS One, 2015. **10**(5): p. e0123834. <http://www.ncbi.nlm.nih.gov/pubmed/25946464>.

13. Nakao, Y., et al., *Genome sequence of the lager brewing yeast, an interspecies hybrid.* DNA Res, 2009. **16**(2): p. 115-29. <http://www.ncbi.nlm.nih.gov/pubmed/19261625>.

14. Gonzalez, S.S., E. Barrio, and A. Querol, *Molecular characterization of new natural hybrids of Saccharomyces cerevisiae and S. kudriavzevii in brewing.* Appl Environ Microbiol, 2008. **74**(8): p. 2314-20. <http://www.ncbi.nlm.nih.gov/pubmed/18296532>.

15. Morales, L. and B. Dujon, *Evolutionary role of interspecies hybridization and genetic exchanges in yeasts.* Microbiol Mol Biol Rev, 2012. **76**(4): p. 721-39. <http://www.ncbi.nlm.nih.gov/pubmed/23204364>.

16. Ortiz-Merino, R.A., et al., *Evolutionary restoration of fertility in an interspecies hybrid yeast, by whole-genome duplication after a failed mating-type switch.* PLoS Biol, 2017. **15**(5): p. e2002128. <http://www.ncbi.nlm.nih.gov/pubmed/28510588>.

17. Aury, J.M., *Global trends of whole-genome duplications revealed by the ciliate Paramecium tetraurelia.* Nature, 2006. **444**: p. 171-178. .

18. Soltis, D.E., *Polyploidy and angiosperm diversification.* Am. J. Bot., 2009. **96**: p. 336-348. .

19. Soltis, P.S. and D.E. Soltis, *The role of hybridization in plant speciation.* Annu. Rev. Plant Biol., 2009. **60**: p. 561-588. .

20. Doyle, J.J., et al., *Evolutionary genetics of genome merger and doubling in plants.* Annu Rev Genet, 2008. **42**: p. 443-61. <http://www.ncbi.nlm.nih.gov/pubmed/18983261>.

21. Hegarty, M., et al., *Lessons from natural and artificial polyploids in higher plants.* Cytogenet Genome Res, 2013. **140**(2-4): p. 204-25. <http://www.ncbi.nlm.nih.gov/pubmed/23816545>.

22. Cui, L., *Widespread genome duplications throughout the history of flowering plants.* Genome Res., 2006. **16**: p. 738-749. .

23. De Bodt, S., S. Maere, and Y. Van de Peer, *Genome duplication and the origin of angiosperms.* Trends Ecol. Evol., 2005. **20**: p. 591-597. .

24. Payseur, B.A. and L.H. Rieseberg, *A genomic perspective on hybridization and speciation.* Mol Ecol, 2016. **25**(11): p. 2337-60. <http://www.ncbi.nlm.nih.gov/pubmed/26836441>.

25. Goulet, B.E., F. Roda, and R. Hopkins, *Hybridization in Plants: Old Ideas, New Techniques.* Plant Physiol, 2017. **173**(1): p. 65-78. <http://www.ncbi.nlm.nih.gov/pubmed/27895205>.

26. Lim, K.Y., et al., *Rapid chromosome evolution in recently formed polyploids in Tragopogon (Asteraceae).* PLoS One, 2008. **3**(10): p. e3353. <http://www.ncbi.nlm.nih.gov/pubmed/18843372>.

27. Symonds, V.V., P.S. Soltis, and D.E. Soltis, *Dynamics of polyploid formation in Tragopogon (Asteraceae): recurrent formation, gene flow, and population structure.* Evolution, 2010. **64**(7): p. 1984-2003. <http://www.ncbi.nlm.nih.gov/pubmed/20199558>.

28. Mao, J.F. and X.R. Wang, *Distinct niche divergence characterizes the homoploid hybrid speciation of Pinus densata on the Tibetan plateau.* Am Nat, 2011. **177**(4): p. 424-39. <http://www.ncbi.nlm.nih.gov/pubmed/21460565>.

29. Guggisberg, A., et al., *Genomic origin and organization of the allopolyploid Primula egaliksensis investigated by in situ hybridization.* Ann Bot, 2008. **101**(7): p. 919-27. <http://www.ncbi.nlm.nih.gov/pubmed/18308718>.

30. Ungerer, M.C., et al., *Rapid hybrid speciation in wild sunflowers.* Proc Natl Acad Sci U S A, 1998. **95**(20): p. 11757-62. <http://www.ncbi.nlm.nih.gov/pubmed/9751738>.

31. Rieseberg, L.H., et al., *Hybridization and the colonization of novel habitats by annual sunflowers.* Genetica, 2007. **129**(2): p. 149-65. <http://www.ncbi.nlm.nih.gov/pubmed/16955330>.

32. Rieseberg, L.H., et al., *Major ecological transitions in wild sunflowers facilitated by hybridization.* Science, 2003. **301**(5637): p. 1211-6. <http://www.ncbi.nlm.nih.gov/pubmed/12907807>.

33. Arnold, M.L., C.M. Buckner, and J.J. Robinson, *Pollen-mediated introgression and hybrid speciation in Louisiana irises.* Proc Natl Acad Sci U S A, 1991. **88**(4): p. 1398-402. <http://www.ncbi.nlm.nih.gov/pubmed/11607153>.

34. Arnold, M.L., et al., *Asymmetric introgressive hybridization among louisiana iris species.* Genes (Basel), 2010. **1**(1): p. 9-22. <http://www.ncbi.nlm.nih.gov/pubmed/24710008>.

35. Hamlin, J.A. and M.L. Arnold, *Determining population structure and hybridization for two iris species.* Ecol Evol, 2014. **4**(6): p. 743-55. <http://www.ncbi.nlm.nih.gov/pubmed/24683457>.

36. McCarthy, E.W., et al., *The effect of polyploidy and hybridization on the evolution of floral colour in Nicotiana (Solanaceae).* Ann Bot, 2015. **115**(7): p. 1117-31. <http://www.ncbi.nlm.nih.gov/pubmed/25979919>.

37. Kelly, L.J., et al., *Intragenic recombination events and evidence for hybrid speciation in Nicotiana (Solanaceae).* Mol Biol Evol, 2010. **27**(4): p. 781-99. <http://www.ncbi.nlm.nih.gov/pubmed/19897524>.

38. Fuentes, I., et al., *Horizontal genome transfer as an asexual path to the formation of new species.* Nature, 2014. **511**(7508): p. 232-5. <http://www.ncbi.nlm.nih.gov/pubmed/24909992>.

39. Vega, Y., et al., *Outcomes of extensive hybridization and introgression in Epidendrum (Orchidaceae): can we rely on species boundaries?* PLoS One, 2013. **8**(11): p. e80662. <http://www.ncbi.nlm.nih.gov/pubmed/24224057>.

40. Hulsey, C.D. and F.J. Garcia-de-Leon, *Introgressive hybridization in a trophically polymorphic cichlid.* Ecol Evol, 2013. **3**(13): p. 4536-47. <http://www.ncbi.nlm.nih.gov/pubmed/24340193>.

41. Albertin, W., et al., *Differential regulation of gene products in newly synthesized Brassica napus allotetraploids is not related to protein function nor subcellular localization.* BMC Genomics, 2007. **8**: p. 56. <http://www.ncbi.nlm.nih.gov/pubmed/17313678>.

42. Chalhoub, B., et al., *Plant genetics. Early allopolyploid evolution in the post-Neolithic Brassica napus oilseed genome.* Science, 2014. **345**(6199): p. 950-3. <http://www.ncbi.nlm.nih.gov/pubmed/25146293>.

43. Albertin, W., et al., *Numerous and rapid nonstochastic modifications of gene products in newly synthesized Brassica napus allotetraploids.* Genetics, 2006. **173**(2): p. 1101-13. <http://www.ncbi.nlm.nih.gov/pubmed/16624896>.

44. Casneuf, T., et al., *Nonrandom divergence of gene expression following gene and genome duplications in the flowering plant Arabidopsis thaliana.* Genome Biol, 2006. **7**(2): p. R13. <http://www.ncbi.nlm.nih.gov/pubmed/16507168>.

45. Schmickl, R., et al., *The evolutionary history of the Arabidopsis arenosa complex: diverse tetraploids mask the Western Carpathian center of species and genetic diversity.* PLoS One, 2012. **7**(8): p. e42691. <http://www.ncbi.nlm.nih.gov/pubmed/22880083>.

46. Schmickl, R. and M.A. Koch, *Arabidopsis hybrid speciation processes.* Proc Natl Acad Sci U S A, 2011. **108**(34): p. 14192-7. <http://www.ncbi.nlm.nih.gov/pubmed/21825128>.

47. Schmickl, R., et al., *The evolutionary history of the Arabidopsis lyrata complex: a hybrid in the amphi-Beringian area closes a large distribution gap and builds up a genetic barrier.* BMC Evol Biol, 2010. **10**: p. 98. <http://www.ncbi.nlm.nih.gov/pubmed/20377907>.

48. Marfil, C.F., E.L. Camadro, and R.W. Masuelli, *Phenotypic instability and epigenetic variability in a diploid potato of hybrid origin, Solanum ruiz-lealii.* BMC Plant Biol, 2009. **9**: p. 21. <http://www.ncbi.nlm.nih.gov/pubmed/19232108>.

49. Pendinen, G., et al., *Genomic in situ hybridization reveals both auto- and allopolyploid origins of different North and Central American hexaploid potato (Solanum sect. Petota) species.* Genome, 2012. **55**(6): p. 407-15. <http://www.ncbi.nlm.nih.gov/pubmed/22594521>.

50. Pendinen, G., et al., *Allopolyploid speciation of the Mexican tetraploid potato species Solanum stoloniferum and S. hjertingii revealed by genomic in situ hybridization.* Genome, 2008. **51**(9): p. 714-20. <http://www.ncbi.nlm.nih.gov/pubmed/18772949>.

51. Anderson, E., Stebbins, G.L., Jr. , *Hybridization as an evolutionary stimulus.* Evolution, 1954. **8**: p. 378–388. .

52. Stebbins, J., G.L., *Cataclysmic Evolution.* Scientific American, 1951. **184**(4): p. 54 –59. .

53. Ozkan, H., A.A. Levy, and M. Feldman, *Allopolyploidy-induced rapid genome evolution in the wheat (Aegilops-Triticum) group.* Plant Cell, 2001. **13**(8): p. 1735-47. <http://www.ncbi.nlm.nih.gov/pubmed/11487689>.

54. Ozkan, H., M. Tuna, and K. Arumuganathan, *Nonadditive changes in genome size during allopolyploidization in the wheat (aegilops-triticum) group.* J Hered, 2003. **94**(3): p. 260-4. <http://www.ncbi.nlm.nih.gov/pubmed/12816968>.

55. Qi, B., et al., *Global transgenerational gene expression dynamics in two newly synthesized allohexaploid wheat (Triticum aestivum) lines.* BMC Biol, 2012. **10**: p. 3. <http://www.ncbi.nlm.nih.gov/pubmed/22277161>.

56. Marcussen, T., et al., *Ancient hybridizations among the ancestral genomes of bread wheat.* Science, 2014. **345**(6194): p. 1250092. <http://www.ncbi.nlm.nih.gov/pubmed/25035499>.

57. Flagel, L., et al., *Duplicate gene expression in allopolyploid Gossypium reveals two temporally distinct phases of expression evolution.* BMC Biol, 2008. **6**: p. 16. <http://www.ncbi.nlm.nih.gov/pubmed/18416842>.

58. Flagel, L.E. and J.F. Wendel, *Evolutionary rate variation, genomic dominance and duplicate gene expression evolution during allotetraploid cotton speciation.* New Phytol, 2010. **186**(1): p. 184-93. <http://www.ncbi.nlm.nih.gov/pubmed/20002320>.

59. Dowling, T.S., CL, *The role of hybridization and introgression in the diversification of animals.* Annu Rev Ecol Syst, 1997. **28**: p. 593–619. .

60. Schwenk, K., N. Brede, and B. Streit, *Introduction. Extent, processes and evolutionary impact of interspecific hybridization in animals.* Philos Trans R Soc Lond B Biol Sci, 2008. **363**(1505): p. 2805-11. <http://www.ncbi.nlm.nih.gov/pubmed/18534946>.

61. Mallet, J., *Hybridization, ecological races and the nature of species: empirical evidence for the ease of speciation.* Philos Trans R Soc Lond B Biol Sci, 2008. **363**(1506): p. 2971-86. <http://www.ncbi.nlm.nih.gov/pubmed/18579473>.

62. Schwarz, D., et al., *Host shift to an invasive plant triggers rapid animal hybrid speciation.* Nature, 2005. **436**(7050): p. 546-9. <http://www.ncbi.nlm.nih.gov/pubmed/16049486>.

63. Schwarz, D., et al., *A novel preference for an invasive plant as a mechanism for animal hybrid speciation.* Evolution, 2007. **61**(2): p. 245-56. <http://www.ncbi.nlm.nih.gov/pubmed/17348936>.

64. Wen, D., et al., *Reticulate evolutionary history and extensive introgression in mosquito species revealed by phylogenetic network analysis.* Mol Ecol, 2016. **25**(11): p. 2361-72. <http://www.ncbi.nlm.nih.gov/pubmed/26808290>.

65. Crawford, J.E., et al., *Reticulate Speciation and Barriers to Introgression in the Anopheles gambiae Species Complex.* Genome Biol Evol, 2015. **7**(11): p. 3116-31. <http://www.ncbi.nlm.nih.gov/pubmed/26615027>.

66. Norris, L.C., et al., *Adaptive introgression in an African malaria mosquito coincident with the increased usage of insecticide-treated bed nets.* Proc Natl Acad Sci U S A, 2015. **112**(3): p. 815-20. <http://www.ncbi.nlm.nih.gov/pubmed/25561525>.

67. Kunte, K., et al., *Sex Chromosome Mosaicism and Hybrid Speciation among Tiger Swallowtail Butterflies.* PLoS Genet, 2011. **7**(9): p. e1002274. <http://www.ncbi.nlm.nih.gov/pubmed/21931567>.

68. Ording, G.J., et al., *Allochronic isolation and incipient hybrid speciation in tiger swallowtail butterflies.* Oecologia, 2010. **162**(2): p. 523-31. <http://www.ncbi.nlm.nih.gov/pubmed/19937057>.

69. Dupuis, J.R. and F.A. Sperling, *Hybrid dynamics in a species group of swallowtail butterflies.* J Evol Biol, 2016. <http://www.ncbi.nlm.nih.gov/pubmed/27364539>.

70. Mallet, J., et al., *Natural hybridization in heliconiine butterflies: the species boundary as a continuum.* BMC Evol Biol, 2007. **7**: p. 28. <http://www.ncbi.nlm.nih.gov/pubmed/17319954>.

71. Pardo-Diaz, C., et al., *Adaptive Introgression across Species Boundaries in Heliconius Butterflies.* PLoS Genet, 2012. **8**(6): p. e1002752. <http://www.ncbi.nlm.nih.gov/pubmed/22737081>.

72. Heliconius, G.C., *Butterfly genome reveals promiscuous exchange of mimicry adaptations among species.* Nature, 2012. **487**(7405): p. 94-8. <http://www.ncbi.nlm.nih.gov/pubmed/22722851>.

73. Nadeau, N.J., et al., *Genomic islands of divergence in hybridizing Heliconius butterflies identified by large-scale targeted sequencing.* Philos Trans R Soc Lond B Biol Sci, 2012. **367**(1587): p. 343-53. <http://www.ncbi.nlm.nih.gov/pubmed/22201164>.

74. Zhang, W., et al., *Genome-wide introgression among distantly related Heliconius butterfly species.* Genome Biol, 2016. **17**(1): p. 25. <http://www.ncbi.nlm.nih.gov/pubmed/26921238>.

75. Salazar, C., et al., *Genetic evidence for hybrid trait speciation in heliconius butterflies.* PLoS Genet, 2010. **6**(4): p. e1000930. <http://www.ncbi.nlm.nih.gov/pubmed/20442862>.

76. Dasmahapatra, K.K., et al., *Genetic analysis of a wild-caught hybrid between non-sister Heliconius butterfly species.* Biol Lett, 2007. **3**(6): p. 660-3. <http://www.ncbi.nlm.nih.gov/pubmed/17804337>.

77. Jiggins, C.D., et al., *Review. Hybrid trait speciation and Heliconius butterflies.* Philos Trans R Soc Lond B Biol Sci, 2008. **363**(1506): p. 3047-54. <http://www.ncbi.nlm.nih.gov/pubmed/18579480>.

78. Brower, A.V., *Introgression of wing pattern alleles and speciation via homoploid hybridization in Heliconius butterflies: a review of evidence from the genome.* Proc Biol Sci, 2013. **280**(1752): p. 20122302. <http://www.ncbi.nlm.nih.gov/pubmed/23235702>.

79. Mavarez, J., et al., *Speciation by hybridization in Heliconius butterflies.* Nature, 2006. **441**(7095): p. 868-71. <http://www.ncbi.nlm.nih.gov/pubmed/16778888>.

80. Kronauer, D.J., et al., *Hybridization in East African swarm-raiding army ants.* Front Zool, 2011. **8**: p. 20. <http://www.ncbi.nlm.nih.gov/pubmed/21859477>.

81. Nonacs, P., *Interspecific hybridization in ants: at the intersection of ecology, evolution, and behavior.* Ecology, 2006. **87**(9): p. 2143-7. <http://www.ncbi.nlm.nih.gov/pubmed/16995613>.

82. Leniaud, L., et al., *Social hybridogenesis in the clonal ant Cataglyphis hispanica.* Curr Biol, 2012. **22**(13): p. 1188-93. <http://www.ncbi.nlm.nih.gov/pubmed/22683263>.

83. Darras, H. and S. Aron, *Introgression of mitochondrial DNA among lineages in a hybridogenetic ant.* Biol Lett, 2015. **11**(2): p. 20140971. <http://www.ncbi.nlm.nih.gov/pubmed/25652221>.

84. Helms Cahan, S. and L. Keller, *Complex hybrid origin of genetic caste determination in harvester ants.* Nature, 2003. **424**(6946): p. 306-9. <http://www.ncbi.nlm.nih.gov/pubmed/12867980>.

85. Renaut, S., *Contemporary hybrid speciation in sculpins (Cottus spp.).* Mol Ecol, 2011. **20**(7): p. 1320-1. <http://www.ncbi.nlm.nih.gov/pubmed/21426430>.

86. Czypionka, T., et al., *Transcriptome changes after genome-wide admixture in invasive sculpins (Cottus).* Mol Ecol, 2012. **21**(19): p. 4797-810. <http://www.ncbi.nlm.nih.gov/pubmed/22650446>.

87. Stelbrink, B., et al., *Age estimates for an adaptive lake fish radiation, its mitochondrial introgression, and an unexpected sister group: Sailfin silversides of the Malili Lakes system in Sulawesi.* BMC Evol Biol, 2014. **14**: p. 94. <http://www.ncbi.nlm.nih.gov/pubmed/24886257>.

88. Herder, F., et al., *Adaptive radiation and hybridization in Wallace's Dreamponds: evidence from sailfin silversides in the Malili Lakes of Sulawesi.* Proc Biol Sci, 2006. **273**(1598): p. 2209-17. <http://www.ncbi.nlm.nih.gov/pubmed/16901841>.

89. Keller, I., et al., *Population genomic signatures of divergent adaptation, gene flow and hybrid speciation in the rapid radiation of Lake Victoria cichlid fishes.* Mol Ecol, 2013. **22**(11): p. 2848-63. <http://www.ncbi.nlm.nih.gov/pubmed/23121191>.

90. Brawand, D., et al., *The genomic substrate for adaptive radiation in African cichlid fish.* Nature, 2014. **513**(7518): p. 375-81. <http://www.ncbi.nlm.nih.gov/pubmed/25186727>.

91. Selz, O.M., et al., *Relaxed trait covariance in interspecific cichlid hybrids predicts morphological diversity in adaptive radiations.* J Evol Biol, 2014. **27**(1): p. 11-24. <http://www.ncbi.nlm.nih.gov/pubmed/24330234>.

92. Selz, O.M., et al., *Behavioural isolation may facilitate homoploid hybrid speciation in cichlid fish.* J Evol Biol, 2014. **27**(2): p. 275-89. <http://www.ncbi.nlm.nih.gov/pubmed/24372872>.

93. Svensson, O., et al., *Hybridization generates a hopeful monster: a hermaphroditic selfing cichlid.* R Soc Open Sci, 2016. **3**(3): p. 150684. <http://www.ncbi.nlm.nih.gov/pubmed/27069660>.

94. Smith, P.F., A. Konings, and I. Kornfield, *Hybrid origin of a cichlid population in Lake Malawi: implications for genetic variation and species diversity.* Mol Ecol, 2003. **12**(9): p. 2497-504. <http://www.ncbi.nlm.nih.gov/pubmed/12919487>.

95. Schwarzer, J., et al., *Repeated trans-watershed hybridization among haplochromine cichlids (Cichlidae) was triggered by Neogene landscape evolution.* Proc Biol Sci, 2012. **279**(1746): p. 4389-98. <http://www.ncbi.nlm.nih.gov/pubmed/22951733>.

96. Magalhaes, I.S., et al., *Untangling the evolutionary history of a highly polymorphic species: introgressive hybridization and high genetic structure in the desert cichlid fish Herichtys minckleyi.* Mol Ecol, 2015. **24**(17): p. 4505-20. <http://www.ncbi.nlm.nih.gov/pubmed/26175313>.

97. Joyce, D.A., et al., *Repeated colonization and hybridization in Lake Malawi cichlids.* Curr Biol, 2011. **21**(3): p. R108-9. <http://www.ncbi.nlm.nih.gov/pubmed/21300271>.

98. Hermansen, J.S., et al., *Hybrid speciation in sparrows I: phenotypic intermediacy, genetic admixture and barriers to gene flow.* Mol Ecol, 2011. **20**(18): p. 3812-22. <http://www.ncbi.nlm.nih.gov/pubmed/21771138>.

99. Eroukhmanoff, F., et al., *Local adaptation within a hybrid species.* Heredity (Edinb), 2013. **111**(4): p. 286-92. <http://www.ncbi.nlm.nih.gov/pubmed/23695379>.

100. Elgvin, T.O., et al., *Hybrid speciation in sparrows II: a role for sex chromosomes?* Mol Ecol, 2011. **20**(18): p. 3823-37. <http://www.ncbi.nlm.nih.gov/pubmed/21762432>.

101. Jacobsen, F. and K.E. Omland, *Increasing evidence of the role of gene flow in animal evolution: hybrid speciation in the yellow-rumped warbler complex.* Mol Ecol, 2011. **20**(11): p. 2236-9. <http://www.ncbi.nlm.nih.gov/pubmed/21739625>.

102. Vallender, R., et al., *Complex hybridization dynamics between golden-winged and blue-winged warblers (Vermivora chrysoptera and Vermivora pinus) revealed by AFLP, microsatellite, intron and mtDNA markers.* Mol Ecol, 2007. **16**(10): p. 2017-29. <http://www.ncbi.nlm.nih.gov/pubmed/17498229>.

103. Brelsford, A., B. Mila, and D.E. Irwin, *Hybrid origin of Audubon's warbler.* Mol Ecol, 2011. **20**(11): p. 2380-9. <http://www.ncbi.nlm.nih.gov/pubmed/21435063>.

104. Toews, D.P., A. Brelsford, and D.E. Irwin, *Isotopic variation across the Audubon's-myrtle warbler hybrid zone.* J Evol Biol, 2014. **27**(6): p. 1179-91. <http://www.ncbi.nlm.nih.gov/pubmed/24779396>.

105. Mila, B., T.B. Smith, and R.K. Wayne, *Speciation and rapid phenotypic differentiation in the yellow-rumped warbler Dendroica coronata complex.* Mol Ecol, 2007. **16**(1): p. 159-73. <http://www.ncbi.nlm.nih.gov/pubmed/17181728>.

106. Grant, P.R., B.R. Grant, and K. Petren, *Hybridization in the recent past.* Am Nat, 2005. **166**(1): p. 56-67. <http://www.ncbi.nlm.nih.gov/pubmed/15937789>.

107. Grant, P., Grant, BR, *Hybridization of bird species.* Science, 1992. **256**: p. 193–197. .

108. Grant, P.R., et al., *Convergent evolution of Darwin's finches caused by introgressive hybridization and selection.* Evolution, 2004. **58**(7): p. 1588-99. <http://www.ncbi.nlm.nih.gov/pubmed/15341160>.

109. Grant, B.R. and P.R. Grant, *Fission and fusion of Darwin's finches populations.* Philos Trans R Soc Lond B Biol Sci, 2008. **363**(1505): p. 2821-9. <http://www.ncbi.nlm.nih.gov/pubmed/18508750>.

110. Grant, P.R. and B.R. Grant, *40 Years of Evolution. Darwin’s Finches on Daphne Major Island*2014: Princeton Univ. Press. .

111. Lamichhaney, S., et al., *Evolution of Darwin's finches and their beaks revealed by genome sequencing.* Nature, 2015. **518**(7539): p. 371-5. <http://www.ncbi.nlm.nih.gov/pubmed/25686609>.

112. Palmer, D.H. and M.R. Kronforst, *Divergence and gene flow among Darwin's finches: A genome-wide view of adaptive radiation driven by interspecies allele sharing.* Bioessays, 2015. **37**(9): p. 968-74. <http://www.ncbi.nlm.nih.gov/pubmed/26200327>.

113. Almen, M.S., et al., *Adaptive radiation of Darwin's finches revisited using whole genome sequencing.* Bioessays, 2016. **38**(1): p. 14-20. <http://www.ncbi.nlm.nih.gov/pubmed/26606649>.

114. Amaral, A.R., et al., *Species tree of a recent radiation: the subfamily Delphininae (Cetacea, Mammalia).* Mol Phylogenet Evol, 2012. **64**(1): p. 243-53. <http://www.ncbi.nlm.nih.gov/pubmed/22503758>.

115. Amaral, A.R., et al., *Hybrid speciation in a marine mammal: the clymene dolphin (Stenella clymene).* PLoS One, 2014. **9**(1): p. e83645. <http://www.ncbi.nlm.nih.gov/pubmed/24421898>.

116. Khan, F.A., C.D. Phillips, and R.J. Baker, *Timeframes of speciation, reticulation, and hybridization in the bulldog bat explained through phylogenetic analyses of all genetic transmission elements.* Syst Biol, 2014. **63**(1): p. 96-110. <http://www.ncbi.nlm.nih.gov/pubmed/24149076>.

117. Bogdanowicz, W., K. Piksa, and A. Tereba, *Hybridization hotspots at bat swarming sites.* PLoS One, 2012. **7**(12): p. e53334. <http://www.ncbi.nlm.nih.gov/pubmed/23300912>.

118. Liu, J., et al., *Reticulate evolution: frequent introgressive hybridization among Chinese hares (genus lepus) revealed by analyses of multiple mitochondrial and nuclear DNA loci.* BMC Evol Biol, 2011. **11**: p. 223. <http://www.ncbi.nlm.nih.gov/pubmed/21794180>.

119. Wu, Y., et al., *Bidirectional introgressive hybridization between Lepus capensis and Lepus yarkandensis.* Mol Phylogenet Evol, 2011. **59**(3): p. 545-55. <http://www.ncbi.nlm.nih.gov/pubmed/21463697>.

120. Li, G., et al., *Phylogenomic evidence for ancient hybridization in the genomes of living cats (Felidae).* Genome Res, 2016. **26**(1): p. 1-11. <http://www.ncbi.nlm.nih.gov/pubmed/26518481>.

121. Roos, C., et al., *Nuclear versus mitochondrial DNA: evidence for hybridization in colobine monkeys.* BMC Evol Biol, 2011. **11**: p. 77. <http://www.ncbi.nlm.nih.gov/pubmed/21435245>.

122. Keller, C., et al., *Introgressive hybridization in southern African baboons shapes patterns of mtDNA variation.* Am J Phys Anthropol, 2010. **142**(1): p. 125-36. <http://www.ncbi.nlm.nih.gov/pubmed/19918986>.

123. Zinner, D., et al., *Mitochondrial phylogeography of baboons (Papio spp.): indication for introgressive hybridization?* BMC Evol Biol, 2009. **9**: p. 83. <http://www.ncbi.nlm.nih.gov/pubmed/19389236>.

124. Alberts, S.C. and J. Altmann, *Immigration and hybridization patterns of yellow and anubis baboons in and around Amboseli, Kenya.* Am J Primatol, 2001. **53**(4): p. 139-54. <http://www.ncbi.nlm.nih.gov/pubmed/11283975>.

125. Tung, J., et al., *Genetic evidence reveals temporal change in hybridization patterns in a wild baboon population.* Mol Ecol, 2008. **17**(8): p. 1998-2011. <http://www.ncbi.nlm.nih.gov/pubmed/18363664>.

126. Ackermann, R.R., et al., *Further evidence for phenotypic signatures of hybridization in descendant baboon populations.* J Hum Evol, 2014. **76**: p. 54-62. <http://www.ncbi.nlm.nih.gov/pubmed/24935168>.
