# Supplementary material for: Living Organisms Author Their Read-Write Genomes in Evolution
Source: Biology (Basel). 2017 Dec 6;6(4):42. doi: 10.3390/biology6040042 (PMC5745447; doi:10.3390/biology6040042)
Supplement: Supplementary file 1 [file biology-06-00042-s001.tgz › biology-224185-supplementary & PUBMED links/biology-224185.zip/Shapiro - Living Organisms Author Their Read-Write Genomes in Evolution - Supplemental Material.Renumbered and Approved + PUBMED links/Supplementary Table S5 Genomic consequences of experimental interspecific hybridization in plants and ]

| **Supplementary Table 5 Genomic consequences of experimental interspecific hybridization in plants and animals** [[1](#_ENREF_1)] | | |
| --- | --- | --- |
| **Genome Effect** | **References** | |
|  | **Plants** | **Animals** |
| Changes in ploidy (mostly WGD) | [[2-10](#_ENREF_2)] | [[11](#_ENREF_11), [12](#_ENREF_12)] |
| Alteration of epigenetic modifications to the genome | [[13](#_ENREF_13), [14](#_ENREF_14)] [[8](#_ENREF_8), [15-21](#_ENREF_15)] [[17](#_ENREF_17), [22-35](#_ENREF_22)] | [[36-44](#_ENREF_36)] |
| Alterations in expression patterns across the genome | [[8](#_ENREF_8), [17](#_ENREF_17), [19](#_ENREF_19), [33](#_ENREF_33), [34](#_ENREF_34), [45-49](#_ENREF_45)] | [[50-52](#_ENREF_50)] [[41](#_ENREF_41), [53-56](#_ENREF_53)] |
| Activation and spread of mobile DNA elements | [[1](#_ENREF_1), [57-59](#_ENREF_57)] [[57](#_ENREF_57), [60-62](#_ENREF_60)] [[32](#_ENREF_32), [63-74](#_ENREF_63)] | [[39](#_ENREF_39), [40](#_ENREF_40), [61](#_ENREF_61), [75-84](#_ENREF_75)] |
| Genome restructuring involving mobile DNA elements | [[65](#_ENREF_65), [85-87](#_ENREF_85)] | [[39](#_ENREF_39), [61](#_ENREF_61), [79-81](#_ENREF_79), [88-90](#_ENREF_88)] |
| Changes in chromosome structure and karyotype | [[59](#_ENREF_59), [91-99](#_ENREF_91)] | [[100-104](#_ENREF_100)] |
| Alteration of tandem repetitive DNA arrays and centromeres | [[105](#_ENREF_105)] | [[106](#_ENREF_106), [107](#_ENREF_107)] |

REFERENCES

1. Parisod, C., et al., *Impact of transposable elements on the organization and function of allopolyploid genomes.* New Phytol, 2010. **186**(1): p. 37-45. <http://www.ncbi.nlm.nih.gov/pubmed/20002321>.

2. Hegarty, M. and S. Hiscock, *Polyploidy: doubling up for evolutionary success.* Curr. Biol., 2007. **17**: p. R927-R929. .

3. Soltis, D.E., *Polyploidy and angiosperm diversification.* Am. J. Bot., 2009. **96**: p. 336-348. .

4. Soltis, D.E., P.S. Soltis, and J.A. Tate, *Advances in the study of polyploidy since plant speciation.* New Phytol., 2003. **161**: p. 173-191. .

5. Doyle, J.J., et al., *Evolutionary genetics of genome merger and doubling in plants.* Annu Rev Genet, 2008. **42**: p. 443-61. <http://www.ncbi.nlm.nih.gov/pubmed/18983261>.

6. Buggs, R.J., P.S. Soltis, and D.E. Soltis, *Does hybridization between divergent progenitors drive whole-genome duplication?* Mol Ecol, 2009. **18**(16): p. 3334-9. <http://www.ncbi.nlm.nih.gov/pubmed/19627486>.

7. Tayale, A. and C. Parisod, *Natural pathways to polyploidy in plants and consequences for genome reorganization.* Cytogenet Genome Res, 2013. **140**(2-4): p. 79-96. <http://www.ncbi.nlm.nih.gov/pubmed/23751271>.

8. Hegarty, M.J., et al., *Transcriptome shock after interspecific hybridization in senecio is ameliorated by genome duplication.* Curr Biol, 2006. **16**(16): p. 1652-9. <http://www.ncbi.nlm.nih.gov/pubmed/16920628>.

9. Baack, E.J., K.D. Whitney, and L.H. Rieseberg, *Hybridization and genome size evolution: timing and magnitude of nuclear DNA content increases in Helianthus homoploid hybrid species.* New Phytol, 2005. **167**(2): p. 623-30. <http://www.ncbi.nlm.nih.gov/pubmed/15998412>.

10. Paun, O., et al., *Hybrid speciation in angiosperms: parental divergence drives ploidy.* New Phytol, 2009. **182**(2): p. 507-18. <http://www.ncbi.nlm.nih.gov/pubmed/19220761>.

11. Liu, S., *Distant hybridization leads to different ploidy fishes.* Sci China Life Sci, 2010. **53**(4): p. 416-25. <http://www.ncbi.nlm.nih.gov/pubmed/20596907>.

12. Liu, S., et al., *The formation of the polyploid hybrids from different subfamily fish crossings and its evolutionary significance.* Genetics, 2007. **176**(2): p. 1023-34. <http://www.ncbi.nlm.nih.gov/pubmed/17507678>.

13. Greaves, I.K., et al., *Inheritance of Trans Chromosomal Methylation patterns from Arabidopsis F1 hybrids.* Proc Natl Acad Sci U S A, 2014. **111**(5): p. 2017-22. <http://www.ncbi.nlm.nih.gov/pubmed/24449910>.

14. Wang, X., et al., *Tissue culture-induced genetic and epigenetic alterations in rice pure-lines, F1 hybrids and polyploids.* BMC Plant Biol, 2013. **13**: p. 77. <http://www.ncbi.nlm.nih.gov/pubmed/23642214>.

15. Shaked, H., et al., *Sequence elimination and cytosine methylation are rapid and reproducible responses of the genome to wide hybridization and allopolyploidy in wheat.* Plant Cell, 2001. **13**(8): p. 1749-59. <http://www.ncbi.nlm.nih.gov/pubmed/11487690>.

16. Comai, L., et al., *Do the different parental 'heteromes' cause genomic shock in newly formed allopolyploids?* Philos Trans R Soc Lond B Biol Sci, 2003. **358**(1434): p. 1149-55. <http://www.ncbi.nlm.nih.gov/pubmed/12831481>.

17. Josefsson, C., B. Dilkes, and L. Comai, *Parent-dependent loss of gene silencing during interspecies hybridization.* Curr Biol, 2006. **16**(13): p. 1322-8. <http://www.ncbi.nlm.nih.gov/pubmed/16824920>.

18. Jones, R.N. and M. Hegarty, *Order out of chaos in the hybrid plant nucleus.* Cytogenet Genome Res, 2009. **126**(4): p. 376-89. <http://www.ncbi.nlm.nih.gov/pubmed/20016131>.

19. Hegarty, M.J., et al., *Changes to gene expression associated with hybrid speciation in plants: further insights from transcriptomic studies in Senecio.* Philos Trans R Soc Lond B Biol Sci, 2008. **363**(1506): p. 3055-69. <http://www.ncbi.nlm.nih.gov/pubmed/18579474>.

20. Buggs, R.J., et al., *Transcriptomic shock generates evolutionary novelty in a newly formed, natural allopolyploid plant.* Curr Biol, 2011. **21**(7): p. 551-6. <http://www.ncbi.nlm.nih.gov/pubmed/21419627>.

21. Buggs, R.J., et al., *Gene loss and silencing in Tragopogon miscellus (Asteraceae): comparison of natural and synthetic allotetraploids.* Heredity, 2009. **103**(1): p. 73-81. <http://www.ncbi.nlm.nih.gov/pubmed/19277058>.

22. Sehrish, T., et al., *Gene silencing via DNA methylation in naturally occurring Tragopogon miscellus (Asteraceae) allopolyploids.* BMC Genomics, 2014. **15**: p. 701. <http://www.ncbi.nlm.nih.gov/pubmed/25145399>.

23. Salmon, A., M.L. Ainouche, and J.F. Wendel, *Genetic and epigenetic consequences of recent hybridization and polyploidy in Spartina (Poaceae).* Mol Ecol, 2005. **14**(4): p. 1163-75. <http://www.ncbi.nlm.nih.gov/pubmed/15773943>.

24. Marfil, C.F., E.L. Camadro, and R.W. Masuelli, *Phenotypic instability and epigenetic variability in a diploid potato of hybrid origin, Solanum ruiz-lealii.* BMC Plant Biol, 2009. **9**: p. 21. <http://www.ncbi.nlm.nih.gov/pubmed/19232108>.

25. Nasrallah, J.B., et al., *Epigenetic mechanisms for breakdown of self-incompatibility in interspecific hybrids.* Genetics, 2007. **175**(4): p. 1965-73. <http://www.ncbi.nlm.nih.gov/pubmed/17237505>.

26. Groszmann, M., et al., *Changes in 24-nt siRNA levels in Arabidopsis hybrids suggest an epigenetic contribution to hybrid vigor.* Proc Natl Acad Sci U S A, 2011. **108**(6): p. 2617-22. <http://www.ncbi.nlm.nih.gov/pubmed/21266545>.

27. Greaves, I.K., et al., *Trans chromosomal methylation in Arabidopsis hybrids.* Proc Natl Acad Sci U S A, 2012. **109**(9): p. 3570-5. <http://www.ncbi.nlm.nih.gov/pubmed/22331882>.

28. Sanei, M., et al., *Loss of centromeric histone H3 (CENH3) from centromeres precedes uniparental chromosome elimination in interspecific barley hybrids.* Proc Natl Acad Sci U S A, 2011. **108**(33): p. E498-505. <http://www.ncbi.nlm.nih.gov/pubmed/21746892>.

29. Hegarty, M.J., et al., *Nonadditive changes to cytosine methylation as a consequence of hybridization and genome duplication in Senecio (Asteraceae).* Mol Ecol, 2011. **20**(1): p. 105-13. <http://www.ncbi.nlm.nih.gov/pubmed/21073590>.

30. Martienssen, R.A., *Heterochromatin, small RNA and post-fertilization dysgenesis in allopolyploid and interploid hybrids of Arabidopsis.* New Phytol, 2010. **186**(1): p. 46-53. <http://www.ncbi.nlm.nih.gov/pubmed/20409176>.

31. Moghaddam, A.M., et al., *Additive inheritance of histone modifications in Arabidopsis thaliana intra-specific hybrids.* Plant J, 2011. **67**(4): p. 691-700. <http://www.ncbi.nlm.nih.gov/pubmed/21554454>.

32. Zhang, X., et al., *Genomic change, retrotransposon mobilization and extensive cytosine methylation alteration in Brassica napus introgressions from two intertribal hybridizations.* PLoS One, 2013. **8**(2): p. e56346. <http://www.ncbi.nlm.nih.gov/pubmed/23468861>.

33. He, G., et al., *Conservation and divergence of transcriptomic and epigenomic variation in maize hybrids.* Genome Biol, 2013. **14**(6): p. R57. <http://www.ncbi.nlm.nih.gov/pubmed/23758703>.

34. Shivaprasad, P.V., et al., *Extraordinary transgressive phenotypes of hybrid tomato are influenced by epigenetics and small silencing RNAs.* Embo J, 2012. **31**(2): p. 257-66. <http://www.ncbi.nlm.nih.gov/pubmed/22179699>.

35. Cara, N., C.F. Marfil, and R.W. Masuelli, *Epigenetic patterns newly established after interspecific hybridization in natural populations of Solanum.* Ecol Evol, 2013. **3**(11): p. 3764-79. <http://www.ncbi.nlm.nih.gov/pubmed/24198938>.

36. Vrana, P.B., et al., *Genetic and epigenetic incompatibilities underlie hybrid dysgenesis in Peromyscus.* Nat Genet, 2000. **25**(1): p. 120-4. <http://www.ncbi.nlm.nih.gov/pubmed/10802670>.

37. Vrana, P.B., et al., *Genomic imprinting is disrupted in interspecific Peromyscus hybrids.* Nat Genet, 1998. **20**(4): p. 362-5. <http://www.ncbi.nlm.nih.gov/pubmed/9843208>.

38. Brown, J.D., D. Golden, and R.J. O'Neill, *Methylation perturbations in retroelements within the genome of a Mus interspecific hybrid correlate with double minute chromosome formation.* Genomics, 2008. **91**(3): p. 267-73. <http://www.ncbi.nlm.nih.gov/pubmed/18226492>.

39. O'Neill, R.J., M.J. O'Neill, and J.A. Graves, *Undermethylation associated with retroelement activation and chromosome remodelling in an interspecific mammalian hybrid.* Nature, 1998. **393**(6680): p. 68-72. <http://www.ncbi.nlm.nih.gov/pubmed/9590690>.

40. Brown, J.D., V. Piccuillo, and R.J. O'Neill, *Retroelement demethylation associated with abnormal placentation in Mus musculus x Mus caroli hybrids.* Biol Reprod, 2012. **86**(3): p. 88. <http://www.ncbi.nlm.nih.gov/pubmed/22116807>.

41. Kelleher, E.S., N.B. Edelman, and D.A. Barbash, *Drosophila interspecific hybrids phenocopy piRNA-pathway mutants.* PLoS Biol, 2012. **10**(11): p. e1001428. <http://www.ncbi.nlm.nih.gov/pubmed/23189033>.

42. Xiao, J., et al., *DNA methylation analysis of allotetraploid hybrids of red crucian carp (Carassius auratus red var.) and common carp (Cyprinus carpio L.).* PLoS One, 2013. **8**(2): p. e56409. <http://www.ncbi.nlm.nih.gov/pubmed/23457564>.

43. Wiley, C.D., et al., *Patterns of hybrid loss of imprinting reveal tissue- and cluster-specific regulation.* PLoS One, 2008. **3**(10): p. e3572. <http://www.ncbi.nlm.nih.gov/pubmed/18958286>.

44. Schutt, S., et al., *DNA methylation in placentas of interspecies mouse hybrids.* Genetics, 2003. **165**(1): p. 223-8. <http://www.ncbi.nlm.nih.gov/pubmed/14504229>.

45. Chelaifa, H., A. Monnier, and M. Ainouche, *Transcriptomic changes following recent natural hybridization and allopolyploidy in the salt marsh species Spartina x townsendii and Spartina anglica (Poaceae).* New Phytol, 2010. **186**(1): p. 161-74. <http://www.ncbi.nlm.nih.gov/pubmed/20149114>.

46. Hegarty, M.J., *Changes to gene expression associated with hybrid speciation in plants: further insights from transcriptomic studies in Senecio.* Philos. Trans. R. Soc. Lond. B, 2008. **363**: p. 3055-3069. .

47. Kunz, C., et al., *Studies on the effects of a flanking repetitive sequence on the expression of single-copy transgenes in Nicotiana sylvestris and in N. sylvestris-N. tomentosiformis hybrids.* Plant Mol Biol, 2003. **52**(1): p. 203-15. <http://www.ncbi.nlm.nih.gov/pubmed/12825700>.

48. Hegarty, M.J., et al., *Extreme changes to gene expression associated with homoploid hybrid speciation.* Mol Ecol, 2009. **18**(5): p. 877-89. <http://www.ncbi.nlm.nih.gov/pubmed/19175502>.

49. Comai, L., et al., *Phenotypic instability and rapid gene silencing in newly formed arabidopsis allotetraploids.* Plant Cell, 2000. **12**(9): p. 1551-68. <http://www.ncbi.nlm.nih.gov/pubmed/11006331>.

50. Renaut, S. and L. Bernatchez, *Transcriptome-wide signature of hybrid breakdown associated with intrinsic reproductive isolation in lake whitefish species pairs (Coregonus spp. Salmonidae).* Heredity, 2011. **106**(6): p. 1003-11. <http://www.ncbi.nlm.nih.gov/pubmed/21119703>.

51. Hill-Burns, E.M. and A.G. Clark, *Functional regulatory divergence of the innate immune system in interspecific Drosophila hybrids.* Mol Biol Evol, 2010. **27**(11): p. 2596-605. <http://www.ncbi.nlm.nih.gov/pubmed/20551040>.

52. Wolf, J.B., R.J. Oakey, and R. Feil, *Imprinted gene expression in hybrids: perturbed mechanisms and evolutionary implications.* Heredity (Edinb), 2014. <http://www.ncbi.nlm.nih.gov/pubmed/24619185>.

53. L'Hote, D., et al., *Gene expression regulation in the context of mouse interspecific mosaic genomes.* Genome Biol, 2008. **9**(8): p. R133. <http://www.ncbi.nlm.nih.gov/pubmed/18752664>.

54. Erwin, A.A., et al., *piRNAs Are Associated with Diverse Transgenerational Effects on Gene and Transposon Expression in a Hybrid Dysgenic Syndrome of D. virilis.* PLoS Genet, 2015. **11**(8): p. e1005332. <http://www.ncbi.nlm.nih.gov/pubmed/26241928>.

55. Lopez-Maestre, H., et al., *Identification of misexpressed genetic elements in hybrids between Drosophila-related species.* Sci Rep, 2017. **7**: p. 40618. <http://www.ncbi.nlm.nih.gov/pubmed/28091568>.

56. Czypionka, T., et al., *Transcriptome changes after genome-wide admixture in invasive sculpins (Cottus).* Mol Ecol, 2012. **21**(19): p. 4797-810. <http://www.ncbi.nlm.nih.gov/pubmed/22650446>.

57. Michalak, P., *An eruption of mobile elements in genomes of hybrid sunflowers.* Heredity (Edinb), 2010. **104**(4): p. 329-30. <http://www.ncbi.nlm.nih.gov/pubmed/20068587>.

58. Grandbastien, M.A., et al., *Stress activation and genomic impact of Tnt1 retrotransposons in Solanaceae.* Cytogenet Genome Res, 2005. **110**(1-4): p. 229-41. <http://www.ncbi.nlm.nih.gov/pubmed/16093677>.

59. Madlung, A., et al., *Genomic changes in synthetic Arabidopsis polyploids.* Plant J, 2005. **41**(2): p. 221-30. <http://www.ncbi.nlm.nih.gov/pubmed/15634199>.

60. Michalak, P., *Epigenetic, transposon and small RNA determinants of hybrid dysfunctions.* Heredity, 2009. **102**(1): p. 45-50. <http://www.ncbi.nlm.nih.gov/pubmed/18545265>.

61. Vela, D., et al., *A genome-wide survey of genetic instability by transposition in Drosophila hybrids.* PLoS One, 2014. **9**(2): p. e88992. <http://www.ncbi.nlm.nih.gov/pubmed/24586475>.

62. Fontdevila, A., *Hybrid genome evolution by transposition.* Cytogenet Genome Res, 2005. **110**(1-4): p. 49-55. <http://www.ncbi.nlm.nih.gov/pubmed/16093657>.

63. Ungerer, M.C., S.C. Strakosh, and K.M. Stimpson, *Proliferation of Ty3/gypsy-like retrotransposons in hybrid sunflower taxa inferred from phylogenetic data.* BMC Biol, 2009. **7**: p. 40. <http://www.ncbi.nlm.nih.gov/pubmed/19594956>.

64. Kawakami, T., et al., *Different scales of Ty1/copia-like retrotransposon proliferation in the genomes of three diploid hybrid sunflower species.* Heredity, 2010. **104**(4): p. 341-50. <http://www.ncbi.nlm.nih.gov/pubmed/20068588>.

65. Ungerer, M.C., S.C. Strakosh, and Y. Zhen, *Genome expansion in three hybrid sunflower species is associated with retrotransposon proliferation.* Curr Biol, 2006. **16**(20): p. R872-3. <http://www.ncbi.nlm.nih.gov/pubmed/17055967>.

66. Ungerer, M.C. and T. Kawakami, *Transcriptional dynamics of LTR retrotransposons in early generation and ancient sunflower hybrids.* Genome Biol Evol, 2013. **5**(2): p. 329-37. <http://www.ncbi.nlm.nih.gov/pubmed/23335122>.

67. Wang, N., et al., *Transpositional reactivation of the Dart transposon family in rice lines derived from introgressive hybridization with Zizania latifolia.* BMC Plant Biol, 2010. **10**: p. 190. <http://www.ncbi.nlm.nih.gov/pubmed/20796287>.

68. Scascitelli, M., M. Cognet, and K.L. Adams, *An interspecific plant hybrid shows novel changes in parental splice forms of genes for splicing factors.* Genetics, 2010. **184**(4): p. 975-83. <http://www.ncbi.nlm.nih.gov/pubmed/20100939>.

69. Wang, N., et al., *Transpositional reactivation of the Dart transposon family in rice lines derived from introgressive hybridization with Zizania latifolia.* BMC Plant Biol, 2010. **10**: p. 190. <http://www.ncbi.nlm.nih.gov/pubmed/20796287>.

70. Yaakov, B. and K. Kashkush, *Mobilization of Stowaway-like MITEs in newly formed allohexaploid wheat species.* Plant Mol Biol, 2012. **80**(4-5): p. 419-27. <http://www.ncbi.nlm.nih.gov/pubmed/22933118>.

71. Yaakov, B. and K. Kashkush, *Massive alterations of the methylation patterns around DNA transposons in the first four generations of a newly formed wheat allohexaploid.* Genome, 2011. **54**(1): p. 42-9. <http://www.ncbi.nlm.nih.gov/pubmed/21217805>.

72. Kraitshtein, Z., et al., *Genetic and epigenetic dynamics of a retrotransposon after allopolyploidization of wheat.* Genetics, 2010. **186**(3): p. 801-12. <http://www.ncbi.nlm.nih.gov/pubmed/20823338>.

73. Yaakov, B. and K. Kashkush, *Methylation, transcription, and rearrangements of transposable elements in synthetic allopolyploids.* Int J Plant Genomics, 2011. **2011**: p. 569826. <http://www.ncbi.nlm.nih.gov/pubmed/21760771>.

74. Yaakov, B., et al., *Copy number variation of transposable elements in Triticum-Aegilops genus suggests evolutionary and revolutionary dynamics following allopolyploidization.* Plant Cell Rep, 2013. **32**(10): p. 1615-24. <http://www.ncbi.nlm.nih.gov/pubmed/23807536>.

75. Guerreiro, M.P., *Interspecific hybridization as a genomic stressor inducing mobilization of transposable elements in Drosophila.* Mob Genet Elements, 2014. **4**: p. e34394. <http://www.ncbi.nlm.nih.gov/pubmed/25136509>.

76. Carnelossi, E.A., et al., *Specific activation of an I-like element in Drosophila interspecific hybrids.* Genome Biol Evol, 2014. **6**(7): p. 1806-17. <http://www.ncbi.nlm.nih.gov/pubmed/24966182>.

77. Scheinker, V.S., et al., *A long terminal repeat-containing retrotransposon is mobilized during hybrid dysgenesis in Drosophila virilis.* Proc Natl Acad Sci U S A, 1990. **87**(24): p. 9615-9. <http://www.ncbi.nlm.nih.gov/pubmed/2175908>.

78. Yannopoulos, G., et al., *hobo is responsible for the induction of hybrid dysgenesis by strains of Drosophila melanogaster bearing the male recombination factor 23.5MRF.* Cell, 1987. **49**(4): p. 487-95. <http://www.ncbi.nlm.nih.gov/pubmed/3032457>.

79. Bucheton, A., *I transposable elements and I-R hybrid dysgenesis in Drosophila.* Trends Genet, 1990. **6**(1): p. 16-21. <http://www.ncbi.nlm.nih.gov/pubmed/2158161>.

80. Fawcett, D.H., et al., *Transposable elements controlling I-R hybrid dysgenesis in D. melanogaster are similar to mammalian LINEs.* Cell, 1986. **47**(6): p. 1007-15. <http://www.ncbi.nlm.nih.gov/pubmed/2430722>.

81. Petrov, D.A., et al., *Diverse transposable elements are mobilized in hybrid dysgenesis in Drosophila virilis.* Proceedings of the National Academy of Sciences of the United States of America, 1995. **92**(17): p. 8050-8054. .

82. Labrador, M., et al., *Interspecific hybridization increases transposition rates of Osvaldo.* Mol Biol Evol, 1999. **16**(7): p. 931-7. <http://www.ncbi.nlm.nih.gov/pubmed/10406110>.

83. Garcia Guerreiro, M.P., *Behaviour of the transposable elements copia and mdg1 in hybrids between the sibling species Drosophila melanogaster and D. simulans.* Heredity (Edinb), 1996. **77 ( Pt 1)**: p. 40-6. <http://www.ncbi.nlm.nih.gov/pubmed/8682693>.

84. Arkhipova, I.R. and F. Rodriguez, *Genetic and epigenetic changes involving (retro)transposons in animal hybrids and polyploids.* Cytogenet Genome Res, 2013. **140**(2-4): p. 295-311. <http://www.ncbi.nlm.nih.gov/pubmed/23899811>.

85. Senerchia, N., F. Felber, and C. Parisod, *Genome reorganization in F1 hybrids uncovers the role of retrotransposons in reproductive isolation.* Proc Biol Sci, 2015. **282**(1804): p. 20142874. <http://www.ncbi.nlm.nih.gov/pubmed/25716787>.

86. Parisod, C., et al., *Rapid structural and epigenetic reorganization near transposable elements in hybrid and allopolyploid genomes in Spartina.* New Phytol, 2009. **184**(4): p. 1003-15. <http://www.ncbi.nlm.nih.gov/pubmed/19780987>.

87. Bento, M., et al., *Retrotransposons represent the most labile fraction for genomic rearrangements in polyploid plant species.* Cytogenet Genome Res, 2013. **140**(2-4): p. 286-94. <http://www.ncbi.nlm.nih.gov/pubmed/23899810>.

88. Kidwell, M.G., J.F. Kidwell, and J.A. Sved, *Hybrid Dysgenesis in DROSOPHILA MELANOGASTER: A Syndrome of Aberrant Traits Including Mutation, Sterility and Male Recombination.* Genetics, 1977. **86**(4): p. 813-33. <http://www.ncbi.nlm.nih.gov/pubmed/17248751>.

89. Kidwell, M.G., *Hybrid dysgenesis in Drosophila melanogaster: nature and inheritance of P element regulation.* Genetics, 1985. **111**(2): p. 337-50. <http://www.ncbi.nlm.nih.gov/pubmed/2996978>.

90. Romero-Soriano, V., et al., *Drosophila Females Undergo Genome Expansion after Interspecific Hybridization.* Genome Biol Evol, 2016. **8**(3): p. 556-61. <http://www.ncbi.nlm.nih.gov/pubmed/26872773>.

91. Lim, K.Y., et al., *Rapid chromosome evolution in recently formed polyploids in Tragopogon (Asteraceae).* PLoS One, 2008. **3**(10): p. e3353. <http://www.ncbi.nlm.nih.gov/pubmed/18843372>.

92. Chester, M., et al., *Extensive chromosomal variation in a recently formed natural allopolyploid species, Tragopogon miscellus (Asteraceae).* Proc Natl Acad Sci U S A, 2012. **109**(4): p. 1176-81. <http://www.ncbi.nlm.nih.gov/pubmed/22228301>.

93. Lai, Z., et al., *Extensive chromosomal repatterning and the evolution of sterility barriers in hybrid sunflower species.* Genetics, 2005. **171**(1): p. 291-303. <http://www.ncbi.nlm.nih.gov/pubmed/16183908>.

94. Nicolas, S.D., et al., *Non-random distribution of extensive chromosome rearrangements in Brassica napus depends on genome organization.* Plant J, 2012. <http://www.ncbi.nlm.nih.gov/pubmed/22268419>.

95. Marfil, C.F., et al., *Genomic instability in Solanum tuberosum x Solanum kurtzianum interspecific hybrids.* Genome, 2006. **49**(2): p. 104-13. <http://www.ncbi.nlm.nih.gov/pubmed/16498460>.

96. Han, F.P., et al., *Rapid genomic changes in interspecific and intergeneric hybrids and allopolyploids of Triticeae.* Genome, 2003. **46**(4): p. 716-23. <http://www.ncbi.nlm.nih.gov/pubmed/12897878>.

97. Li, Z.Y. and X.H. Ge, *Unique chromosome behavior and genetic control in Brassica x Orychophragmus wide hybrids: a review.* Plant Cell Rep, 2007. **26**(6): p. 701-10. <http://www.ncbi.nlm.nih.gov/pubmed/17221227>.

98. Xie, S., et al., *An assessment of chromosomal rearrangements in neopolyploids of Lilium hybrids.* Genome, 2010. **53**(6): p. 439-46. <http://www.ncbi.nlm.nih.gov/pubmed/20555433>.

99. Gao, X., et al., *High frequency of HMW-GS sequence variation through somatic hybridization between Agropyron elongatum and common wheat.* Planta, 2010. **231**(2): p. 245-50. <http://www.ncbi.nlm.nih.gov/pubmed/19902245>.

100. Sakai, C., et al., *Chromosome elimination in the interspecific hybrid medaka between Oryzias latipes and O. hubbsi.* Chromosome Res, 2007. **15**(6): p. 697-709. <http://www.ncbi.nlm.nih.gov/pubmed/17603754>.

101. Brown, J.D., et al., *Interspecific hybridization induced amplification of Mdm2 on double minutes in a Mus hybrid.* Cytogenet Genome Res, 2002. **98**(2-3): p. 184-8. <http://www.ncbi.nlm.nih.gov/pubmed/12698001>.

102. Ivanitskaya, E., L. Rashkovetsky, and E. Nevo, *Chromosomes in a hybrid zone of Israeli mole rars (Spalax, Rodentia).* Genetika, 2010. **46**(10): p. 1301-4. <http://www.ncbi.nlm.nih.gov/pubmed/21250542>.

103. Qin, Q., et al., *Rapid genomic changes in allopolyploids of Carassius auratus red var. (female symbol) x Megalobrama amblycephala (male symbol).* Sci Rep, 2016. **6**: p. 34417. <http://www.ncbi.nlm.nih.gov/pubmed/27703178>.

104. Qin, Q., et al., *Abnormal chromosome behavior during meiosis in the allotetraploid of Carassius auratus red var. (female symbol)xMegalobrama amblycephala (male symbol).* BMC Genet, 2014. **15**: p. 95. <http://www.ncbi.nlm.nih.gov/pubmed/25178799>.

105. Guo, X., et al., *De Novo Centromere Formation and Centromeric Sequence Expansion in Wheat and its Wide Hybrids.* PLoS Genet, 2016. **12**(4): p. e1005997. <http://www.ncbi.nlm.nih.gov/pubmed/27110907>.

106. Metcalfe, C.J., et al., *Genomic instability within centromeres of interspecific marsupial hybrids.* Genetics, 2007. **177**(4): p. 2507-17. <http://www.ncbi.nlm.nih.gov/pubmed/18073443>.

107. Baicharoen, S., et al., *Locational diversity of alpha satellite DNA and intergeneric hybridization aspects in the Nomascus and Hylobates genera of small apes.* PLoS One, 2014. **9**(10): p. e109151. <http://www.ncbi.nlm.nih.gov/pubmed/25290445>.
