# Supplementary material for: Living Organisms Author Their Read-Write Genomes in Evolution
Source: Biology (Basel). 2017 Dec 6;6(4):42. doi: 10.3390/biology6040042 (PMC5745447; doi:10.3390/biology6040042)
Supplement: Supplementary file 1 [file biology-06-00042-s001.tgz › biology-224185-supplementary & PUBMED links/biology-224185.zip/Shapiro - Living Organisms Author Their Read-Write Genomes in Evolution - Supplemental Material.Renumbered and Approved + PUBMED links/Supplementary Table S6 Modes of Horizontal DNA Transfer.docx]

| **Supplementary Table 6. Modes of Horizontal DNA Transfer** | |
| --- | --- |
| **DNA Transfer Mode** | **Donor-Recipient Taxa (References)** |
| Liberation and uptake of extracellular DNA (“Transformation”) | **Archaea-archaea** [[1-4](#_ENREF_1)]  **Bacteria-bacteria** [[5-11](#_ENREF_5)]  **Bacteria-Archaea** [[12](#_ENREF_12)]  **Yeast-yeast** [[13](#_ENREF_13), [14](#_ENREF_14)]  ***Stramenopile* red alga-alga** [[15](#_ENREF_15)]  **Plant-bacteria** [[16](#_ENREF_16)] |
| Encapsidation in and delivery by a virus or virus-like particle (“Transduction”) | **Archaea-archaea** [[17](#_ENREF_17), [18](#_ENREF_18)]  **Bacteria-bacteria** [[19-23](#_ENREF_19)]  **Insect-insect** [[24](#_ENREF_24), [25](#_ENREF_25)]  **Bacteria-mammal** [[26](#_ENREF_26)]  **Mammal-mammal** [[27](#_ENREF_27)] |
| Establishment of a DNA transport pore between two cell envelopes (“conjugation”) | **Archaea-archaea** [[28-30](#_ENREF_28)]  **Bacteria-bacteria** [[31](#_ENREF_31), [32](#_ENREF_32)]  **Bacteria-yeast** [[33-37](#_ENREF_33)]  **Bacteria-fungi** and **mushrooms** [[35](#_ENREF_35), [38-40](#_ENREF_38)]  **Bacteria-plant** [[41-44](#_ENREF_41)]  **Bacteria-mammalian cells** [[35](#_ENREF_35), [45-47](#_ENREF_45)]  **Bacteria-diverse eukaryotes** [[48](#_ENREF_48)] |
| Unknown mechanism | **Fungus-fungus** [[49](#_ENREF_49)] |
| Cell fusion | **Archaea-archaea** [[50](#_ENREF_50), [51](#_ENREF_51)] |
| Phagotrophism | **Bacteria-protist** [[52](#_ENREF_52)] |
| **Additional DNA transfer modes** | |
| Protoplast fusion | **Bacteria** [[53-56](#_ENREF_53)]  **Yeast** [[57](#_ENREF_57), [58](#_ENREF_58)]  **Fungi** [[59-61](#_ENREF_59)]  **Plants** [[62-65](#_ENREF_62)] |
| Liposomal or membrane vesicle-mediated transfer | **Archaea** [[66-68](#_ENREF_66)]  **Mammalian cells** [[69-72](#_ENREF_69)] |
| Sperm-mediated DNA transfer | **Insect larva** [[73](#_ENREF_73)]  **Mammals** [[74-78](#_ENREF_74)] |
| Parasite- and endosymbiont-mediated transfer (inferred) | **Red algae** [[79](#_ENREF_79), [80](#_ENREF_80)]  **Aphids, parasitoid wasps**, and a **beetle** [[81](#_ENREF_81)]  ***Drosophila*** [[82](#_ENREF_82), [83](#_ENREF_83)]  **Butterflies** [[84](#_ENREF_84), [85](#_ENREF_85)]  **Vertebrates** [[86](#_ENREF_86)]  **Reptile-mammal** [[87](#_ENREF_87)]  **Mammals** [[88](#_ENREF_88)] |

REFERENCES

1. Cline, S.W., L.C. Schalkwyk, and W.F. Doolittle, *Transformation of the archaebacterium Halobacterium volcanii with genomic DNA.* J Bacteriol, 1989. **171**(9): p. 4987-91. <http://www.ncbi.nlm.nih.gov/pubmed/2768194>.

2. Worrell, V.E., et al., *Genetic transformation system in the archaebacterium Methanobacterium thermoautotrophicum Marburg.* J Bacteriol, 1988. **170**(2): p. 653-6. <http://www.ncbi.nlm.nih.gov/pubmed/3422229>.

3. Chimileski, S., et al., *Extracellular DNA metabolism in Haloferax volcanii.* Front Microbiol, 2014. **5**: p. 57. <http://www.ncbi.nlm.nih.gov/pubmed/24600440>.

4. van Wolferen, M., et al., *The archaeal Ced system imports DNA.* Proc Natl Acad Sci U S A, 2016. **113**(9): p. 2496-501. <http://www.ncbi.nlm.nih.gov/pubmed/26884154>.

5. Johnsborg, O., V. Eldholm, and L.S. Havarstein, *Natural genetic transformation: prevalence, mechanisms and function.* Res Microbiol, 2007. **158**(10): p. 767-78. <http://www.ncbi.nlm.nih.gov/pubmed/17997281>.

6. Lorenz, M.G. and W. Wackernagel, *Bacterial gene transfer by natural genetic transformation in the environment.* Microbiol Rev, 1994. **58**(3): p. 563-602. <http://www.ncbi.nlm.nih.gov/pubmed/7968924>.

7. Chen, I. and D. Dubnau, *DNA uptake during bacterial transformation.* Nat Rev Microbiol, 2004. **2**(3): p. 241-9. <http://www.ncbi.nlm.nih.gov/pubmed/15083159>.

8. Claverys, J.P., B. Martin, and P. Polard, *The genetic transformation machinery: composition, localization, and mechanism.* FEMS Microbiol Rev, 2009. **33**(3): p. 643-56. <http://www.ncbi.nlm.nih.gov/pubmed/19228200>.

9. Seitz, P. and M. Blokesch, *Cues and regulatory pathways involved in natural competence and transformation in pathogenic and environmental Gram-negative bacteria.* FEMS Microbiol Rev, 2013. **37**(3): p. 336-63. <http://www.ncbi.nlm.nih.gov/pubmed/22928673>.

10. Johnston, C., et al., *Bacterial transformation: distribution, shared mechanisms and divergent control.* Nat Rev Microbiol, 2014. **12**(3): p. 181-96. <http://www.ncbi.nlm.nih.gov/pubmed/24509783>.

11. Keen, E.C., et al., *Novel "Superspreader" Bacteriophages Promote Horizontal Gene Transfer by Transformation.* MBio, 2017. **8**(1). <http://www.ncbi.nlm.nih.gov/pubmed/28096488>.

12. Dodsworth, J.A., et al., *Inter-domain conjugal transfer of DNA from Bacteria to Archaea.* Appl Environ Microbiol, 2010. **76**(16): p. 5644-7. <http://www.ncbi.nlm.nih.gov/pubmed/20581182>.

13. Bouchonville, K., et al., *Aneuploid chromosomes are highly unstable during DNA transformation of Candida albicans.* Eukaryot Cell, 2009. **8**(10): p. 1554-66. <http://www.ncbi.nlm.nih.gov/pubmed/19700634>.

14. Mitrikeski, P.T., *Ecologically driven competence for exogenous DNA uptake in yeast.* Curr Microbiol, 2015. **70**(6): p. 883-93. <http://www.ncbi.nlm.nih.gov/pubmed/25824091>.

15. Vieler, A., et al., *Genome, functional gene annotation, and nuclear transformation of the heterokont oleaginous alga Nannochloropsis oceanica CCMP1779.* PLoS Genet, 2012. **8**(11): p. e1003064. <http://www.ncbi.nlm.nih.gov/pubmed/23166516>.

16. Pontiroli, A., et al., *Visual evidence of horizontal gene transfer between plants and bacteria in the phytosphere of transplastomic tobacco.* Appl Environ Microbiol, 2009. **75**(10): p. 3314-22. <http://www.ncbi.nlm.nih.gov/pubmed/19329660>.

17. Bertani, G., *Transduction-like gene transfer in the methanogen Methanococcus voltae.* J Bacteriol, 1999. **181**(10): p. 2992-3002. <http://www.ncbi.nlm.nih.gov/pubmed/10321998>.

18. Meile, L., P. Abendschein, and T. Leisinger, *Transduction in the archaebacterium Methanobacterium thermoautotrophicum Marburg.* J Bacteriol, 1990. **172**(6): p. 3507-8. <http://www.ncbi.nlm.nih.gov/pubmed/2345156>.

19. Penades, J.R., et al., *Bacteriophage-mediated spread of bacterial virulence genes.* Curr Opin Microbiol, 2015. **23**: p. 171-8. <http://www.ncbi.nlm.nih.gov/pubmed/25528295>.

20. Miller, R.V., *Environmental bacteriophage-host interactions: factors contribution to natural transduction.* Antonie Van Leeuwenhoek, 2001. **79**(2): p. 141-7. <http://www.ncbi.nlm.nih.gov/pubmed/11520000>.

21. Waddell, T.E., et al., *Generalized transduction by lytic bacteriophages.* Methods Mol Biol, 2009. **501**: p. 293-303. <http://www.ncbi.nlm.nih.gov/pubmed/19066829>.

22. Balcazar, J.L., *Bacteriophages as vehicles for antibiotic resistance genes in the environment.* PLoS Pathog, 2014. **10**(7): p. e1004219. <http://www.ncbi.nlm.nih.gov/pubmed/25078987>.

23. Lang, A.S., O. Zhaxybayeva, and J.T. Beatty, *Gene transfer agents: phage-like elements of genetic exchange.* Nat Rev Microbiol, 2012. **10**(7): p. 472-82. <http://www.ncbi.nlm.nih.gov/pubmed/22683880>.

24. Gilbert, C., et al., *Population genomics supports baculoviruses as vectors of horizontal transfer of insect transposons.* Nat Commun, 2014. **5**: p. 3348. <http://www.ncbi.nlm.nih.gov/pubmed/24556639>.

25. Coates, B.S., *Horizontal transfer of a non-autonomous Helitron among insect and viral genomes.* BMC Genomics, 2015. **16**: p. 137. <http://www.ncbi.nlm.nih.gov/pubmed/25766741>.

26. Wadia, J., A. Eguchi, and S.F. Dowdy, *DNA delivery into mammalian cells using bacteriophage lambda displaying the TAT transduction domain.* Cold Spring Harb Protoc, 2013. **2013**(1). <http://www.ncbi.nlm.nih.gov/pubmed/23282641>.

27. Gallaher, S.D., et al., *Robust in vivo transduction of a genetically stable Epstein-Barr virus episome to hepatocytes in mice by a hybrid viral vector.* J Virol, 2009. **83**(7): p. 3249-57. <http://www.ncbi.nlm.nih.gov/pubmed/19158239>.

28. Stedman, K.M., et al., *pING family of conjugative plasmids from the extremely thermophilic archaeon Sulfolobus islandicus: insights into recombination and conjugation in Crenarchaeota.* J Bacteriol, 2000. **182**(24): p. 7014-20. <http://www.ncbi.nlm.nih.gov/pubmed/11092863>.

29. Prangishvili, D., et al., *Conjugation in archaea: frequent occurrence of conjugative plasmids in Sulfolobus.* Plasmid, 1998. **40**(3): p. 190-202. <http://www.ncbi.nlm.nih.gov/pubmed/9806856>.

30. Schleper, C., et al., *A multicopy plasmid of the extremely thermophilic archaeon Sulfolobus effects its transfer to recipients by mating.* J Bacteriol, 1995. **177**(15): p. 4417-26. <http://www.ncbi.nlm.nih.gov/pubmed/7635827>.

31. Koraimann, G. and M.A. Wagner, *Social behavior and decision making in bacterial conjugation.* Front Cell Infect Microbiol, 2014. **4**: p. 54. <http://www.ncbi.nlm.nih.gov/pubmed/24809026>.

32. Guglielmini, J., et al., *The Repertoire of ICE in Prokaryotes Underscores the Unity, Diversity, and Ubiquity of Conjugation.* PLoS Genet, 2011. **7**(8): p. e1002222. <http://www.ncbi.nlm.nih.gov/pubmed/21876676>.

33. Heinemann, J.A. and G.F. Sprague, Jr., *Transmission of plasmid DNA to yeast by conjugation with bacteria.* Methods Enzymol, 1991. **194**: p. 187-95. <http://www.ncbi.nlm.nih.gov/pubmed/2005787>.

34. Sawasaki, Y., K. Inomata, and K. Yoshida, *Trans-kingdom conjugation between Agrobacterium tumefaciens and Saccharomyces cerevisiae, a bacterium and a yeast.* Plant Cell Physiol, 1996. **37**(1): p. 103-6. <http://www.ncbi.nlm.nih.gov/pubmed/8720926>.

35. Lacroix, B., et al., *A case of promiscuity: Agrobacterium's endless hunt for new partners.* Trends Genet, 2006. **22**(1): p. 29-37. <http://www.ncbi.nlm.nih.gov/pubmed/16289425>.

36. Piers, K.L., et al., *Agrobacterium tumefaciens-mediated transformation of yeast.* Proc Natl Acad Sci U S A\, 1996. **93\**(4\): p. 1613-8\. <http://www.ncbi.nlm.nih.gov/pubmed/8643679>\.

37. Moriguchi, K., et al., *A Fast and Practical Yeast Transformation Method Mediated by Escherichia coli Based on a Trans-Kingdom Conjugal Transfer System: Just Mix Two Cultures and Wait One Hour.* PLoS One, 2016. **11**(2): p. e0148989. <http://www.ncbi.nlm.nih.gov/pubmed/26849654>.

38. de Groot, M.J., et al., *Agrobacterium tumefaciens-mediated transformation of filamentous fungi.* Nat Biotechnol, 1998. **16**(9): p. 839-42. <http://www.ncbi.nlm.nih.gov/pubmed/9743116>.

39. Knight, C.J., A.M. Bailey, and G.D. Foster, *Investigating Agrobacterium-mediated transformation of Verticillium albo-atrum on plant surfaces.* PLoS One, 2010. **5**(10): p. e13684. <http://www.ncbi.nlm.nih.gov/pubmed/21060684>.

40. Fitzpatrick, D.A., M.E. Logue, and G. Butler, *Evidence of recent interkingdom horizontal gene transfer between bacteria and Candida parapsilosis.* BMC Evol Biol, 2008. **8**: p. 181. <http://www.ncbi.nlm.nih.gov/pubmed/18577206>.

41. Zupan, J.R. and P. Zambryski, *Transfer of T-DNA from Agrobacterium to the plant cell.* Plant Physiol, 1995. **107**(4): p. 1041-7. <http://www.ncbi.nlm.nih.gov/pubmed/7770515>.

42. Chung, S.M., M. Vaidya, and T. Tzfira, *Agrobacterium is not alone: gene transfer to plants by viruses and other bacteria.* Trends Plant Sci, 2006. **11**(1): p. 1-4. <http://www.ncbi.nlm.nih.gov/pubmed/16297655>.

43. Lacroix, B. and V. Citovsky, *A Functional Bacterium-to-Plant DNA Transfer Machinery of Rhizobium etli.* PLoS Pathog, 2016. **12**(3): p. e1005502. <http://www.ncbi.nlm.nih.gov/pubmed/26968003>.

44. Broothaerts, W., et al., *Gene transfer to plants by diverse species of bacteria.* Nature, 2005. **433**(7026): p. 629-33. <http://www.ncbi.nlm.nih.gov/pubmed/15703747>.

45. Waters, V.L., *Conjugation between bacterial and mammalian cells.* Nat Genet, 2001. **29**(4): p. 375-6. <http://www.ncbi.nlm.nih.gov/pubmed/11726922>.

46. Kunik, T., et al., *Genetic transformation of HeLa cells by Agrobacterium.* Proc Natl Acad Sci U S A, 2001. **98**(4): p. 1871-6. <http://www.ncbi.nlm.nih.gov/pubmed/11172043>.

47. Schroder, G., et al., *Conjugative DNA transfer into human cells by the VirB/VirD4 type IV secretion system of the bacterial pathogen Bartonella henselae.* Proc Natl Acad Sci U S A, 2011. **108**(35): p. 14643-8. <http://www.ncbi.nlm.nih.gov/pubmed/21844337>.

48. Lacroix, B. and V. Citovsky, *Transfer of DNA from Bacteria to Eukaryotes.* MBio, 2016. **7**(4). <http://www.ncbi.nlm.nih.gov/pubmed/27406565>.

49. Fitzpatrick, D.A., *Horizontal gene transfer in fungi.* FEMS Microbiol Lett, 2012. **329**(1): p. 1-8. <http://www.ncbi.nlm.nih.gov/pubmed/22112233>.

50. Naor, A. and U. Gophna, *Cell fusion and hybrids in Archaea: prospects for genome shuffling and accelerated strain development for biotechnology.* Bioengineered, 2013. **4**(3): p. 126-9. <http://www.ncbi.nlm.nih.gov/pubmed/23111319>.

51. Naor, A., et al., *Low species barriers in halophilic archaea and the formation of recombinant hybrids.* Curr Biol, 2012. **22**(15): p. 1444-8. <http://www.ncbi.nlm.nih.gov/pubmed/22748314>.

52. Doolittle, W.F., *You are what you eat: A gene transfer ratchet could account for bacterial genes in eukaryotic nuclear genomes.* Trends Genet., 1998. **14**: p. 307-311. .

53. Dai, M., et al., *Visualization of protoplast fusion and quantitation of recombination in fused protoplasts of auxotrophic strains of Escherichia coli.* Metab Eng, 2005. **7**(1): p. 45-52. <http://www.ncbi.nlm.nih.gov/pubmed/15974564>.

54. Hopwood, D.A., et al., *Genetic recombination through protoplast fusion in Streptomyces.* Nature, 1977. **268**(5616): p. 171-4. <http://www.ncbi.nlm.nih.gov/pubmed/593313>.

55. Gokhale, D.V., U.S. Puntambekar, and D.N. Deobagkar, *Protoplast fusion: a tool for intergeneric gene transfer in bacteria.* Biotechnol Adv, 1993. **11**(2): p. 199-217. <http://www.ncbi.nlm.nih.gov/pubmed/14545006>.

56. Gasson, M.J., *Genetic transfer systems in lactic acid bacteria.* Antonie Van Leeuwenhoek, 1983. **49**(3): p. 275-82. <http://www.ncbi.nlm.nih.gov/pubmed/6414366>.

57. Curran, B.P. and V.C. Bugeja, *Protoplast fusion in Saccharomyces cerevisiae.* Methods Mol Biol, 1996. **53**: p. 45-9. <http://www.ncbi.nlm.nih.gov/pubmed/8925003>.

58. Steensels, J., et al., *Improving industrial yeast strains: exploiting natural and artificial diversity.* FEMS Microbiol Rev, 2014. **38**(5): p. 947-95. <http://www.ncbi.nlm.nih.gov/pubmed/24724938>.

59. Peberdy, J.F., *Developments in protoplast fusion in fungi.* Microbiol Sci, 1987. **4**(4): p. 108-14. <http://www.ncbi.nlm.nih.gov/pubmed/3153180>.

60. Biot-Pelletier, D. and V.J. Martin, *Evolutionary engineering by genome shuffling.* Appl Microbiol Biotechnol, 2014. **98**(9): p. 3877-87. <http://www.ncbi.nlm.nih.gov/pubmed/24595425>.

61. Baltz, R.H., *Genetic methods and strategies for secondary metabolite yield improvement in actinomycetes.* Antonie Van Leeuwenhoek, 2001. **79**(3-4): p. 251-9. <http://www.ncbi.nlm.nih.gov/pubmed/11816967>.

62. Gamborg, O.L. and F.B. Holl, *Plant protoplast fusion and hybridization.* Basic Life Sci, 1977. **9**: p. 299-316. <http://www.ncbi.nlm.nih.gov/pubmed/336026>.

63. Davey, M.R., et al., *Plant protoplasts: status and biotechnological perspectives.* Biotechnol Adv, 2005. **23**(2): p. 131-71. <http://www.ncbi.nlm.nih.gov/pubmed/15694124>.

64. Fahleson, J. and K. Glimelius, *Protoplast fusion for symmetric somatic hybrid production in Brassicaceae.* Methods Mol Biol, 1999. **111**: p. 195-209. <http://www.ncbi.nlm.nih.gov/pubmed/10080989>.

65. Binding, H., G. Krumbiegel-Schroeren, and R. Nehls, *Protoplast fusion and early development of fusants.* Results Probl Cell Differ, 1986. **12**: p. 37-66. <http://www.ncbi.nlm.nih.gov/pubmed/3529271>.

66. Metcalf, W.W., et al., *A genetic system for Archaea of the genus Methanosarcina: liposome-mediated transformation and construction of shuttle vectors.* Proc Natl Acad Sci U S A, 1997. **94**(6): p. 2626-31. <http://www.ncbi.nlm.nih.gov/pubmed/9122246>.

67. Gaudin, M., et al., *Hyperthermophilic archaea produce membrane vesicles that can transfer DNA.* Environ Microbiol Rep, 2013. **5**(1): p. 109-16. <http://www.ncbi.nlm.nih.gov/pubmed/23757139>.

68. Marguet, E., et al., *Membrane vesicles, nanopods and/or nanotubes produced by hyperthermophilic archaea of the genus Thermococcus.* Biochem Soc Trans, 2013. **41**(1): p. 436-42. <http://www.ncbi.nlm.nih.gov/pubmed/23356325>.

69. Ogorevc, E., V. Kralj-Iglic, and P. Veranic, *The role of extracellular vesicles in phenotypic cancer transformation.* Radiol Oncol, 2013. **47**(3): p. 197-205. <http://www.ncbi.nlm.nih.gov/pubmed/24133383>.

70. Fischer, S., et al., *Indication of Horizontal DNA Gene Transfer by Extracellular Vesicles.* PLoS One, 2016. **11**(9): p. e0163665. <http://www.ncbi.nlm.nih.gov/pubmed/27684368>.

71. Cai, J., et al., *Extracellular vesicle-mediated transfer of donor genomic DNA to recipient cells is a novel mechanism for genetic influence between cells.* J Mol Cell Biol, 2013. **5**(4): p. 227-38. <http://www.ncbi.nlm.nih.gov/pubmed/23580760>.

72. Kawamura, Y., et al., *Extracellular vesicles as trans-genomic agents: emerging roles in disease and evolution.* Cancer Sci, 2017. <http://www.ncbi.nlm.nih.gov/pubmed/28256033>.

73. Zhou, W., et al., *Germline transformation of the silkworm Bombyx mori L. by sperm-mediated gene transfer.* Biol Reprod, 2012. **87**(6): p. 144. <http://www.ncbi.nlm.nih.gov/pubmed/23100618>.

74. Smith, K. and C. Spadafora, *Sperm-mediated gene transfer: applications and implications.* Bioessays, 2005. **27**(5): p. 551-62. <http://www.ncbi.nlm.nih.gov/pubmed/15832378>.

75. Spadafora, C., *Sperm-mediated gene transfer: mechanisms and implications.* Soc Reprod Fertil Suppl, 2007. **65**: p. 459-67. <http://www.ncbi.nlm.nih.gov/pubmed/17644984>.

76. Arias, M.E., et al., *Effect of transfection and co-incubation of bovine sperm with exogenous DNA on sperm quality and functional parameters for its use in sperm-mediated gene transfer.* Zygote, 2017. **25**(1): p. 85-97. <http://www.ncbi.nlm.nih.gov/pubmed/27928970>.

77. Zhao, Y., et al., *Spontaneous uptake of exogenous DNA by goat spermatozoa and selection of donor bucks for sperm-mediated gene transfer.* Mol Biol Rep, 2012. **39**(3): p. 2659-64. <http://www.ncbi.nlm.nih.gov/pubmed/21667250>.

78. Lavitrano, M., et al., *Sperm-mediated gene transfer.* Reprod Fertil Dev, 2006. **18**(1-2): p. 19-23. <http://www.ncbi.nlm.nih.gov/pubmed/16478599>.

79. Goff, L.J. and A.W. Coleman, *Fate of Parasite and Host Organelle DNA during Cellular Transformation of Red Algae by Their Parasites.* Plant Cell, 1995. **7**(11): p. 1899-1911. <http://www.ncbi.nlm.nih.gov/pubmed/12242362>.

80. Qiu, H., H.S. Yoon, and D. Bhattacharya, *Algal endosymbionts as vectors of horizontal gene transfer in photosynthetic eukaryotes.* Front Plant Sci, 2013. **4**: p. 366. <http://www.ncbi.nlm.nih.gov/pubmed/24065973>.

81. Guo, X., et al., *Evidence of horizontal transfer of non-autonomous Lep1 Helitrons facilitated by host-parasite interactions.* Sci Rep, 2014. **4**: p. 5119. <http://www.ncbi.nlm.nih.gov/pubmed/24874102>.

82. Houck, M.A., et al., *Possible horizontal transfer of Drosophila genes by the mite Proctolaelaps regalis.* Science, 1991. **253**(5024): p. 1125-8. <http://www.ncbi.nlm.nih.gov/pubmed/1653453>.

83. Ortiz, M.F., et al., *An evaluation of the ecological relationship between Drosophila species and their parasitoid wasps as an opportunity for horizontal transposon transfer.* Mol Genet Genomics, 2015. **290**(1): p. 67-78. <http://www.ncbi.nlm.nih.gov/pubmed/25146840>.

84. Schneider, S.E. and J.H. Thomas, *Accidental genetic engineers: horizontal sequence transfer from parasitoid wasps to their Lepidopteran hosts.* PLoS One, 2014. **9**(10): p. e109446. <http://www.ncbi.nlm.nih.gov/pubmed/25296163>.

85. Li, Z.W., et al., *Pathogen-origin horizontally transferred genes contribute to the evolution of Lepidopteran insects.* BMC Evol Biol, 2011. **11**: p. 356. <http://www.ncbi.nlm.nih.gov/pubmed/22151541>.

86. Gilbert, C., et al., *A role for host-parasite interactions in the horizontal transfer of transposons across phyla.* Nature, 2010. **464**(7293): p. 1347-50. <http://www.ncbi.nlm.nih.gov/pubmed/20428170>.

87. Piskurek, O. and N. Okada, *Poxviruses as possible vectors for horizontal transfer of retroposons from reptiles to mammals.* Proc Natl Acad Sci U S A, 2007. **104**(29): p. 12046-51. <http://www.ncbi.nlm.nih.gov/pubmed/17623783>.

88. Filee, J., et al., *Mariner transposons are sailing in the genome of the blood-sucking bug Rhodnius prolixus.* BMC Genomics, 2015. **16**: p. 1061. <http://www.ncbi.nlm.nih.gov/pubmed/26666222>.
