# Supplementary material for: Living Organisms Author Their Read-Write Genomes in Evolution
Source: Biology (Basel). 2017 Dec 6;6(4):42. doi: 10.3390/biology6040042 (PMC5745447; doi:10.3390/biology6040042)
Supplement: Supplementary file 1 [file biology-06-00042-s001.tgz › biology-224185-supplementary & PUBMED links/biology-224185.zip/Shapiro - Living Organisms Author Their Read-Write Genomes in Evolution - Supplemental Material.Renumbered and Approved + PUBMED links/Supplementary Table S7 Selected examples of inter.docx]

| **Supplementary Table 7 Selected examples of inter-phylum adaptive horizontal DNA transfers based on genomic data** | | | | |
| --- | --- | --- | --- | --- |
| **Donor** | **Recipient** | **Function(s)** | **Reference(s)** | |
| **Prokaryote-prokaryote** [[1](#_ENREF_1)] | | | | |
| Bacteria | Methanogenic Archaea  (early *Haloarchaea*) | 1,089 transfers, carbon metabolism, membrane transporters, menaquinone biosynthesis, and complexes I-IV of the eubacterial respiratory chain; converted strictly anaerobic, chemolithoautotrophic methanogen into heterotrophic, oxygen-respiring haloarchaeal common ancestor. | [[2](#_ENREF_2)] | |
| Bacteria | Methanogenic Archaea *Methosarcina spp.* | ~5-11% of genetic loci, including gluconeogenesis, proline biosynthesis, transport processes, DNA-repair, environmental sensing, gene regulation, and stress response, such as the bacterial GroEL/GroES chaperone system and the presence of tetrahydrofolate-dependent enzymes | [[3](#_ENREF_3), [4](#_ENREF_4)] | |
| Bacteria | Mesophilic clades descended from thermophilic Archaea: *Thaumarchaeota*, *MG II/III euryarchaeotes*, *Halobacteriales* | Mesophilic metabolic activites (energy conversion, amino acid transport and metabolism, and lipid or membrane biogenesis): *Thaumarchaeota* (937 loci), *MG II/III euryarchaeotes* (1677 loci), *Halobacteriales* (1047 loci) | [[5](#_ENREF_5), [6](#_ENREF_6)] | |
| Bacteria | Early Archaeal progenitors | “13 archaeal higher taxa…correspond to 2,264 group-specific gene acquisitions from bacteria” | [[7](#_ENREF_7), [8](#_ENREF_8)] | |
| **Prokaryote-eukaryote microbe** [[9](#_ENREF_9), [10](#_ENREF_10)] | | | | |
| *Actinobacterium* | Basidiomycete *Agaricomycotina* | Alpha-amylase | | [[11](#_ENREF_11)] |
| Bacteria | Plant pathogenic fungi *Pyrenophora teres* and *Pyrenophora tritici-repentis* | Extracellular proteins, interference with plant defense-response, degradation of plant cell walls, carbohydrate metabolism | | [[10](#_ENREF_10), [12](#_ENREF_12)] |
| Bacteria (40%), fungi (25%), and viruses (22%) | Animal pathogenic fungus *Aspergillus fumigatus* | Central and intermediary metabolism, virulence (including lipase, 4 peptide transporters, gliotoxin synthesis) | | [[13](#_ENREF_13)] |
| Bacteria and Archaea | Red alga *Galdieria sulphuraria* | Growth in high temperature, toxic metal-rich, acidic environments | | [[14](#_ENREF_14)] |
| Bacteria | Red alga *Porphyridium purpureum* | Non-plastid functions encoded in nuclear genome | | [[15](#_ENREF_15)] |
| Actinobacteria, proteobacteria, archaea | Chromalveolates (dinoflagellates of the genera *Karenia* and *Karlodinium*) | Energy metabolism, sugar and amino acid metabolism, cell membrane biosynthesis, substrate transport, DNA repair | | [[16](#_ENREF_16)] |
| Proteobacteria, cyanobacteria and archaea | Diatom *Phaeodactylum tricornutum* | 7.5% of genetic loci (784 loci); organic carbon and nitrogen utilization (xylanases and glucanases, prismane, carbon-nitrogen hydrolase, amidohydrolase), diatom urea cycle (carbamoyl transferase, carbamate kinase, ornithine cyclodeaminase) and polyamine metabolism related to diatom cell wall silicification (*S*-adenosylmethionine (SAM)-dependent decarboxylases and methyltransferases); also cell wall component synthesis, unorthodox DNA replication, repair and recombination mechanisms | | [[17](#_ENREF_17)] |
| Bacteria, archaea | Rumen ciliates | Catabolism of complex carbohydrates | | [[18](#_ENREF_18)] |
| *Actinobacteria* | Plant pathogenic fungal *Phytophthora* species | Cutinase | | [[19](#_ENREF_19)] |
| Bacteria | Fish ciliate scuticociliatosis pathogen *Pseudocohnilembus persalinus* | Cell adhesion, hemolysis and heme utilization | | [[20](#_ENREF_20)] |
| Bacteria, eukaryotes *Dictyostelium*  *discoideum,*  *Entamoeba histolytica,*  *Gibberella zeae,*  *Mastigamoeba balamuthi* | Diplomonad fish parasite *Spironucleus salmonicida* | Amino acid metabolism, DNA repair, protein folding, membrane transport, glycolysis/gluconeogenesis, glyoxylate and dicarboxylate metabolism, pentose phosphate pathway, pyruvate metabolism, starch and sucrose metabolism, nitrogen metabolism, oxidative phosphorylation, purine and pyrimidine metabolism | | [[21](#_ENREF_21)] |
| β,γ-Proteobacteria, Chlamydiae, other bacteria | Red alga *Cyanidioschyzon* | Amino acid, vitamin, lipid, porphyrin biosynthesis; ATP/ADP transport; RNA processing, translation | | [[22](#_ENREF_22)] |
| Arthrobacter | Ascomycete fungi *Penicillium canescens* and *Scopulariopsis* sp | Beta-glucuronidase (enables utilization of glucuronides in vertebrate urine) | | [[23](#_ENREF_23)] |
| Rumen bacteria *Fibrobacter succinogenes* | Rumen fungus *Orpinomyces joyonii* | Endoglucanase (polysaccharide digestion) | | [[24](#_ENREF_24)] |
| Bacteria | Eukaryotic unicellular parasites (*Entamoeba histolytica*, *E. dispar*, *Trichomonas vaginalis*, *Giardia lamblia*, *Trypanosoma brucei*, *T. cruzi*, *Plasmodium falciparum*) | Amino acid, sugar, nucleotide, lipid, and, energy metabolism, host glycan degradation, vitamin and membrane biosynthesis, translation | | [[25-29](#_ENREF_25)] |
| **Eukaryote microbe–eukaryote microbe** [[10](#_ENREF_10), [30](#_ENREF_30), [31](#_ENREF_31)] | | | | |
| Phytopathogenic fungi in genera *Magnaporthiopsis*  or *Colletotrichum* | Phytopathogenic fungi in genera *Magnaporthiopsis*  or *Colletotrichum* | 33-90 horizontally transferred loci enriched for plant cell wall degrading enzymes | | [[32](#_ENREF_32)] |
| Fungi | Plant parasitic oomycetes, *e.g., Phytophthora ramorum* | Secreted proteins for plant cell wall digestion, resisting plant defenses, effector functions | | [[33](#_ENREF_33)] |
| Filamentous plant pathogenic Ascomycete fungus *Magnaporthe grisea* | Filamentous plant Oomycete pathogen *Phytophthora sp.* | Sugar-transporter, purine permease, extracellular dioxygenase/Protocatechuate 3,4-dioxygenase β-subunit, aldose 1-epimerase (osmotropic lifestyle) | | [[34](#_ENREF_34)] |
| Fungal pathogen *Stagonospora nodorum* | Fungal pathogen *Pyrenophora tritici-repentis* | ToxA virulence factor, extends host range | | [[35](#_ENREF_35)] |
| Algae, bacteria | Choanoflagellate *Monosiga brevicollis* | 405 genetic loci (4.4% nuclear genome); carbohydrate and amino acid metabolism, 45 transporters, responses to oxidative, osmotic, antibiotic, or heavy metal stresses, biosynthesis of vitamins C and K12, porphyrins and phospholipids. | | [[36](#_ENREF_36)] [[37](#_ENREF_37)] [[38](#_ENREF_38)] [[39](#_ENREF_39)] |
| **Prokaryote-animal** [[40-42](#_ENREF_40)] | | | | |
| Bacteria, likely including some related to marine species *Vibrio campbellii*, *Desulfovibrio hydrothermalis*, and *Arcobacter nitrofigilis* | Sponge *Amphimedon queenslandica*, a model metazoan ancestor [[43](#_ENREF_43)] | 227 loci transferred, including metallopeptidase, carbohydrate metabolism, polymer degradation, transport, proteolysis, nitrogen metabolism, extracellular matrix biosynthesis | | [[44](#_ENREF_44)] |
| Mainly bacteria, but also fungi, protists, and algae | Bdelloid rotifers | 8-9% of genetic loci, including toxin degradation, biosynthesis of antioxidants and key metabolites, amino acid metabolism, non-ribosomal peptide synthetases | | [[45-47](#_ENREF_45)] |
| Bacteria, fungi | Bdelloid rotifer, *Adineta ricciae* | 16 cellulolytic enzymes | | [[48](#_ENREF_48)] |
| Bacteria | Sponge *Astrosclera willeyana* | Biomineralization | | [[49](#_ENREF_49)] |
| Bacteria | Starlet sea anemone, *Nematostella vectensis* | Shikimic acid biosynthesis, glyoxylate cycle | | [[50](#_ENREF_50), [51](#_ENREF_51)] |
| Bacteria | Cnidarian *Hydra magnipapillata* | Carbohydrate, lipid, nucleotide, amino acid, cofactor, vitamin and xenobiotic metabolism, glycan biosynthesis; transamination, methylation, and acetylation of sugars, polysaccharides, or glycoproteins; bacterial lipopolysaccharide (LPS) biosynthetic pathway | | [[52](#_ENREF_52)] |
| Bacteria | Filarial nematode parasite *Onchocerca flexuosa* | Independence from *Wolbachia* endosymbionts; synthesis of riboflavin, heme and nucleotides; inosine monophosphate and uridine monophosphate biosynthesis | | [[53](#_ENREF_53), [54](#_ENREF_54)] |
| Bacteria, chiefly *rhizobacteria*, and fungi | Plant parasitic nematodes | Cellulose and phytopolymer digestion (multiple transfers to distinct nematode lineages), B vitamin biosynthesis, plant resistance-breaking effector proteins | | [[55-65](#_ENREF_55)] |
| Bacteria | Stick and Leaf Insects | Pectinases | | [[66](#_ENREF_66)] |
| Bacteria | Phytophagous mites and *Lepidoptera* | Detoxification of plant defense cyanogenic glucosides | | [[67](#_ENREF_67)] |
| Bacteria and fungi | Asian longhorned beetle *Anoplophora glabripennis* | Enzymes involved in digestion of woody plant tissues and detoxification of plant allelochemicals | | [[68](#_ENREF_68)] |
| Bacteria and fungi | Herbivorous arthropods (>20 distinct insect and chelicerate species) | Overcoming plant defenses, assimilation of intracellular plant metabolites, digestion of plant cell walls | | [[69](#_ENREF_69)] |
| Bacteria | Coffee berry borer beetle, *Hypothenemus hampe* | Glycosyl hydrolase (galactomannan, major coffee storage polymer, is the substrate) | | [[70](#_ENREF_70)] |
| Bacteria | Silkworm *Bombyx mori* and related *Lepidoptera* | Glycosyl hydrolase family, oxidoreductase family, and amino acid metabolism | | [[71](#_ENREF_71)] |
| *Archaea*, Bacteria, Fungi, Protists, and Plants | 26 animal species (4 *Caenorhabditis*, 12 *Drosophila*, and 10 primates) | Multiple metabolic functions (biosynthetic and degradative), innate immunity responses, antioxidant activities | | [[72](#_ENREF_72)] |
| Bacteria | Arthropods, echinoderms, and vertebrates, including platypus and opossum, but not in splacental mammals | Glyoxylate cycle | | [[51](#_ENREF_51)] |
| Bacteria, Archaea, fungi and plants | Tardigrade *Hypsibius dujardini* (desiccation-resistant animals subject to oxidative stress) | 17.5% of tardigrade genetic loci, particularly oxidative stress tolerance functions: catalases; DNA repair functions, including recombination proteins and translesion polymerases; polyamine biosynthesis; heat shock chaperones | | [[73](#_ENREF_73)] |
| Bacteria | Urochordate *Ciona intestinali*s | Cellulose synthase | | [[74](#_ENREF_74)] |
| **Eukaryotic microbe-animal** [[41](#_ENREF_41)] | | | | |
| Fungi | Pea aphid, two-spotted spider mite *Tetranychus urticae* | Carotenoid pigment biosynthesis, cyanate metabolism | | [[75-77](#_ENREF_75)] |
| Algae and other photosynthetic eukaryotes | Tunicate *Ciona intestinali*s | Molecule transport, cellular regulation and methylation signaling | | [[78](#_ENREF_78)] |
| **Animal-eukaryotic microbe** | | | | |
| Arthropod | Microsporidia intracellular parasites *Encephalitozoon intestinalis* and *Encephalitozoon cuniculi* | Purine nucleotide phosphorylase, folylpolyglutamate synthase | | [[79](#_ENREF_79), [80](#_ENREF_80)] |
| **Prokaryote-plant (non-plastid)** | | | | |
| Bacteria, Archaea, viruses, and sea anemones | Moss *Physcomitrella patens* (primitive land plant) | Xylem formation, plant defense, nitrogen recycling, plus biosynthesis of starch, polyamines, hormones and glutathione; actinoporin water stress adaptation | | [[81](#_ENREF_81), [82](#_ENREF_82)] |
| Bacteria, fungi | Plants | Shikimate biosynthesis, phenylpropanoid pathway leading to synthesis of flavonoids and lignin, TAL transaldolase involved in vascular biogenesis | | [[83](#_ENREF_83), [84](#_ENREF_84)] |
| **Eukarote microbe-plant** | | | | |
| Fungi | Plants (Bryophyte *Physcomitrella patens* and Lycophyte *Selaginella moellendorffil*) | L-fucose permease sugar transporter, , membrane transporter, bifunctional iucA/iucC siderophore biosynthesis protein, phospholipase/carboxylesterase family protein. Both cases that involve HGT from a fungus to the bryophyte moss *P. patens*, the HGT is positioned next to a putative transposable element. | | [[85](#_ENREF_85)] |
| **Plant-microbe** | | | | |
| Plants | Bacteria, fungi, *amoebozoa* | Expansins (plant cell-wall loosening proteins) | | [[86](#_ENREF_86)] |
| Plants | Fungi (Basidiomycete *Laccaria bicolor*, Chytrydiomycete *Bhatrachochytrium dendrobatidis*, Ascomycete *Sclerotinia sclerotiorum*) | Phosphate-responsive protein, zinc binding alcohol dehydrogenase, DUF239 domain protein, zinc finger (C2H2 type) protein | | [[85](#_ENREF_85)] |
| **Plant-plant** [[87](#_ENREF_87), [88](#_ENREF_88)] | | | | |
| Parasitic plant (*Cuscuta sp.*) | Host plant (*Plantago sp.*) | Mitochondrial loci *atp1*, *atp6* and *matR* | | [[89](#_ENREF_89), [90](#_ENREF_90)] |
| *Tetrastigma rafflesiae Miq* (obligate host) | Parasitic flowering plant *Rafflesia cantleyi* Solms-Laubach | Respiration, carbohydrate metabolism, mitochondrial translation, and protein turnover; mitochondrial sequences | | [[91](#_ENREF_91), [92](#_ENREF_92)] [[93](#_ENREF_93)] |
| Grasses, *Panicoideae* genera | 16 diploid barley (*Hordeum*) species | Ribosomal RNA (rDNA) sequences | | [[94](#_ENREF_94)] |
| Bryophyte hornworts | Fern | Novel chimeric photoreceptor--neochrome | | [[95](#_ENREF_95)] |
| *Andropogoneae*, *Cenchrinae* (2 species), and *Melinidinae* C4 plants | Grass lineage *Alloteropsis* (4 independent transfers) | C_4_ photosynthesis activities (phosphoenolpyruvate carboxylase, phosphoenolpyruvate carboxykinase) | | [[96](#_ENREF_96)] |
| Plant | Plants (*Amborella* and others) | Mitochondrial genomes | | [[97-100](#_ENREF_97)] |
| Papilionoid legume | *Phelipanche aegyptiaca*, parasitic species of family *Orobanchaceae* | albumin 1 KNOTTIN-like proteins | | [[101](#_ENREF_101)] |
| *Brassicaceae* host plant | Root parasitic plant *Orobanche aegyptiaca*, shoot parasitic plant *Cuscuta australis* | Strictosidine synthase (independent transfers) | | [[102](#_ENREF_102)] |
| **Animal-animal** | [[41](#_ENREF_41)] |  | |  |
| Fish (herring or sea raven) | Rainbow smelt, *Osmerus mordax* | Type II anti-freeze protein | | [[103](#_ENREF_103), [104](#_ENREF_104)] |
| **Eukaryote-prokaryote** [[105](#_ENREF_105)] | | | | |
| Eukaryotic cells (Impossible to identify because *Legionella* infects and grows in amoebae, protozoa, *paramecium*, macrophages, and many other eukaryotic cells) | Bacteria *Legionella pneumophila* | Eukaryote-like regulatory “effector” proteins injected in the course of infecting eukaryotic cells | | [[106-110](#_ENREF_106)] |
| **Virus-prokaryote** | | | | |
| Halovirus | *Halobacterium salinarum* (Archaea) | B-type DNA polymerase B1 | | [[111](#_ENREF_111)] |
| **Virus-eukaryote** | | | | |
| T3/T7 family bacteriophage | Ancestral eukaryote nuclear and mitochondrial genomes | A-type DNA polymerase Gamma of the mitochondrion (Opisthokonts only, metazoa and fungi), mitochondrial single-subunit RNA Polymerase (mt-ssRNAP), nucleus-encoded mitochondrial replicative helicase (all eukaryotes) | | [[111-113](#_ENREF_111)] |
| Double-stranded RNA viruses (*totiviruses* and *partitiviruses*) | Eukaryote nuclear genomes (plants, arthropods, fungi, nematodes, and protozoa) | Capsid protein and RNA-dependent RNA polymerases | | [[114](#_ENREF_114)] |
| Circular single-stranded DNA viruses (*geminiviruses*, *nanoviruses* and *circoviruses*) | Eukaryotic nuclear genomes (plants, fungi, animals and protists) | Replication initiation protein (Rep)-related sequences | | [[115](#_ENREF_115)] |
| **Cellular organisms-virus** | | | | |
| Bacteria | Diverse temperate bacteriophages (bacterial viruses capable of insertion into “lysogen” bacterial genome as repressed prophage) | Expressed in lysogens: DNA adenine methylase, DNA cytosine methylase, porin (outer membrane transport), mammalian serum resistance, phage attack resistance, improved survival in Peyer's patches, mammalian cell binding, superoxide dismutase (lysogen more virulent in mice), mammalian cell ruffling and cell invasion, Shiga-like toxin (kills mammalian cells by damaging rRNA) | | [[116](#_ENREF_116)] |
| Bacteria (primarily, endosymbionts and parasites), bacteriophages, protists, animals | Nucleocytoplasmic large DNA viruses (NCLDVs), including *Poxviruses* and *Iridoviruses* infecting insects and vertebrates, *Mimiviruses* and *Phycodnaviruses* infecting amoebae, protists and algae | Small and large subunit rDNAs, DNA polymerase, two subunits of the DNA-dependent RNA polymerase, and two subunits of the ribonucleotide reductase, DNA ligase, dUTPase, serine/threonine kinase, thymidine kinase, ribonucleotide reductase, virus-specific signaling and regulatory domains, ubiquitin signaling; defenses against apoptosis, immune response (the MHC class I, interleukin-10, interleukin-24, interleukin-18, the interferon gamma receptor, and tumor necrosis factor receptor II), including growth factors and potential inhibitors of cytokine signaling, peptidases; glutaredoxin and glutathione peroxidase involved in resistance of cells to oxidative stress. | | [[117-123](#_ENREF_117)] |
| Mammal | Influenza virus | 28S rDNA insert in hemagglutinin sequence increases pathogenicity | | [[124](#_ENREF_124)] |
| Gamma-proteobacteria and insects (viral hosts) | Baculovirus | DNA ligase, ribonucleotide reductase 1, SNF2 global transactivator, inhibitor of apoptosis, chitinase, and UDP-glucosyltransferase | | [[125](#_ENREF_125)] |
| Marine phytoplankton *prasinophytes* | Prasinovirus | Glycosyltransferases, methyltransferases and amino acid synthesis enzymes | | [[126](#_ENREF_126)] |

REFERENCES

1. Koonin, E.V., *Horizontal gene transfer: essentiality and evolvability in prokaryotes, and roles in evolutionary transitions.* F1000Res, 2016. **5**. <http://www.ncbi.nlm.nih.gov/pubmed/27508073>.

2. Nelson-Sathi, S., et al., *Acquisition of 1,000 eubacterial genes physiologically transformed a methanogen at the origin of Haloarchaea.* Proc Natl Acad Sci U S A, 2012. **109**(50): p. 20537-42. <http://www.ncbi.nlm.nih.gov/pubmed/23184964>.

3. Garushyants, S.K., M.D. Kazanov, and M.S. Gelfand, *Horizontal gene transfer and genome evolution in Methanosarcina.* BMC Evol Biol, 2015. **15**: p. 102. <http://www.ncbi.nlm.nih.gov/pubmed/26044078>.

4. Deppenmeier, U., et al., *The genome of Methanosarcina mazei: evidence for lateral gene transfer between bacteria and archaea.* J Mol Microbiol Biotechnol, 2002. **4**(4): p. 453-61. <http://www.ncbi.nlm.nih.gov/pubmed/12125824>.

5. Lopez-Garcia, P., et al., *Bacterial gene import and mesophilic adaptation in archaea.* Nat Rev Microbiol, 2015. **13**(7): p. 447-56. <http://www.ncbi.nlm.nih.gov/pubmed/26075362>.

6. Deschamps, P., et al., *Pangenome evidence for extensive interdomain horizontal transfer affecting lineage core and shell genes in uncultured planktonic thaumarchaeota and euryarchaeota.* Genome Biol Evol, 2014. **6**(7): p. 1549-63. <http://www.ncbi.nlm.nih.gov/pubmed/24923324>.

7. Nelson-Sathi, S., et al., *Origins of major archaeal clades correspond to gene acquisitions from bacteria.* Nature, 2015. **517**: p. 77–80. <http://www.ncbi.nlm.nih.gov/pubmed/25317564>.

8. Groussin, M., et al., *Gene Acquisitions from Bacteria at the Origins of Major Archaeal Clades Are Vastly Overestimated.* Mol Biol Evol, 2016. **33**(2): p. 305-10. <http://www.ncbi.nlm.nih.gov/pubmed/26541173>.

9. Marcet-Houben, M. and T. Gabaldon, *Acquisition of prokaryotic genes by fungal genomes.* Trends Genet, 2009. **26**(1): p. 5-8. <http://www.ncbi.nlm.nih.gov/pubmed/19969385>.

10. Soanes, D. and T.A. Richards, *Horizontal gene transfer in eukaryotic plant pathogens.* Annu Rev Phytopathol, 2014. **52**: p. 583-614. <http://www.ncbi.nlm.nih.gov/pubmed/25090479>.

11. Da Lage, J.L., et al., *Gene make-up: rapid and massive intron gains after horizontal transfer of a bacterial alpha-amylase gene to Basidiomycetes.* BMC Evol Biol, 2013. **13**: p. 40. <http://www.ncbi.nlm.nih.gov/pubmed/23405862>.

12. Sun, B.F., et al., *Multiple interkingdom horizontal gene transfers in Pyrenophora and closely related species and their contributions to phytopathogenic lifestyles.* PLoS One, 2013. **8**(3): p. e60029. <http://www.ncbi.nlm.nih.gov/pubmed/23555871>.

13. Mallet, L.V., J. Becq, and P. Deschavanne, *Whole genome evaluation of horizontal transfers in the pathogenic fungus Aspergillus fumigatus.* BMC Genomics, 2010. **11**: p. 171. <http://www.ncbi.nlm.nih.gov/pubmed/20226043>.

14. Schonknecht, G., et al., *Gene transfer from bacteria and archaea facilitated evolution of an extremophilic eukaryote.* Science, 2013. **339**(6124): p. 1207-10. <http://www.ncbi.nlm.nih.gov/pubmed/23471408>.

15. Qiu, H., H.S. Yoon, and D. Bhattacharya, *Algal endosymbionts as vectors of horizontal gene transfer in photosynthetic eukaryotes.* Front Plant Sci, 2013. **4**: p. 366. <http://www.ncbi.nlm.nih.gov/pubmed/24065973>.

16. Nosenko, T. and D. Bhattacharya, *Horizontal gene transfer in chromalveolates.* BMC Evol Biol, 2007. **7**: p. 173. <http://www.ncbi.nlm.nih.gov/pubmed/17894863>.

17. Bowler, C., et al., *The Phaeodactylum genome reveals the evolutionary history of diatom genomes.* Nature, 2008. **456**(7219): p. 239-44. <http://www.ncbi.nlm.nih.gov/pubmed/18923393>.

18. Ricard, G., et al., *Horizontal gene transfer from Bacteria to rumen Ciliates indicates adaptation to their anaerobic, carbohydrates-rich environment.* BMC Genomics, 2006. **7**: p. 22. <http://www.ncbi.nlm.nih.gov/pubmed/16472398>.

19. Belbahri, L., et al., *Evolution of the cutinase gene family: evidence for lateral gene transfer of a candidate Phytophthora virulence factor.* Gene, 2008. **408**(1-2): p. 1-8. <http://www.ncbi.nlm.nih.gov/pubmed/18024004>.

20. Xiong, J., et al., *Genome of the facultative scuticociliatosis pathogen Pseudocohnilembus persalinus provides insight into its virulence through horizontal gene transfer.* Sci Rep, 2015. **5**: p. 15470. <http://www.ncbi.nlm.nih.gov/pubmed/26486372>.

21. Andersson, J.O., et al., *A genomic survey of the fish parasite Spironucleus salmonicida indicates genomic plasticity among diplomonads and significant lateral gene transfer in eukaryote genome evolution.* BMC Genomics, 2007. **8**: p. 51. <http://www.ncbi.nlm.nih.gov/pubmed/17298675>.

22. Huang, J. and J.P. Gogarten, *Concerted gene recruitment in early plant evolution.* Genome Biol, 2008. **9**(7): p. R109. <http://www.ncbi.nlm.nih.gov/pubmed/18611267>.

23. Wenzl, P., et al., *A functional screen identifies lateral transfer of beta-glucuronidase (gus) from bacteria to fungi.* Mol Biol Evol, 2005. **22**(2): p. 308-16. <http://www.ncbi.nlm.nih.gov/pubmed/15483318>.

24. Garcia-Vallve, S., A. Romeu, and J. Palau, *Horizontal gene transfer of glycosyl hydrolases of the rumen fungi.* Mol Biol Evol, 2000. **17**(3): p. 352-61. <http://www.ncbi.nlm.nih.gov/pubmed/10723736>.

25. Loftus, B., et al., *The genome of the protist parasite Entamoeba histolytica.* Nature, 2005. **433**(7028): p. 865-8. <http://www.ncbi.nlm.nih.gov/pubmed/15729342>.

26. Hirt, R.P., C. Alsmark, and T.M. Embley, *Lateral gene transfers and the origins of the eukaryote proteome: a view from microbial parasites.* Curr Opin Microbiol, 2015. **23**: p. 155-62. <http://www.ncbi.nlm.nih.gov/pubmed/25483352>.

27. Strese, A., A. Backlund, and C. Alsmark, *A recently transferred cluster of bacterial genes in Trichomonas vaginalis--lateral gene transfer and the fate of acquired genes.* BMC Evol Biol, 2014. **14**: p. 119. <http://www.ncbi.nlm.nih.gov/pubmed/24898731>.

28. Alsmark, C., et al., *Patterns of prokaryotic lateral gene transfers affecting parasitic microbial eukaryotes.* Genome Biol, 2013. **14**(2): p. R19. <http://www.ncbi.nlm.nih.gov/pubmed/23442822>.

29. Alsmark, U.C., et al., *Horizontal gene transfer in eukaryotic parasites: a case study of Entamoeba histolytica and Trichomonas vaginalis.* Methods Mol Biol, 2009. **532**: p. 489-500. <http://www.ncbi.nlm.nih.gov/pubmed/19271203>.

30. Andersson, J.O., *Horizontal gene transfer between microbial eukaryotes.* Methods Mol Biol, 2009. **532**: p. 473-87. <http://www.ncbi.nlm.nih.gov/pubmed/19271202>.

31. Andersson, J.O., *Gene transfer and diversification of microbial eukaryotes.* Annu Rev Microbiol, 2009. **63**: p. 177-93. <http://www.ncbi.nlm.nih.gov/pubmed/19575565>.

32. Qiu, H., et al., *Extensive horizontal gene transfers between plant pathogenic fungi.* BMC Biol, 2016. **14**: p. 41. <http://www.ncbi.nlm.nih.gov/pubmed/27215567>.

33. Richards, T.A., et al., *Horizontal gene transfer facilitated the evolution of plant parasitic mechanisms in the oomycetes.* Proc Natl Acad Sci U S A, 2011. **108**(37): p. 15258-63. <http://www.ncbi.nlm.nih.gov/pubmed/21878562>.

34. Richards, T.A., et al., *Evolution of filamentous plant pathogens: gene exchange across eukaryotic kingdoms.* Curr Biol, 2006. **16**(18): p. 1857-64. <http://www.ncbi.nlm.nih.gov/pubmed/16979565>.

35. Mehrabi, R., et al., *Horizontal gene and chromosome transfer in plant pathogenic fungi affecting host range.* FEMS Microbiol Rev, 2011. **35**(3): p. 542-54. <http://www.ncbi.nlm.nih.gov/pubmed/21223323>.

36. Nedelcu, A.M., et al., *Adaptive eukaryote-to-eukaryote lateral gene transfer: stress-related genes of algal origin in the closest unicellular relatives of animals.* J Evol Biol, 2008. **21**(6): p. 1852-60. <http://www.ncbi.nlm.nih.gov/pubmed/18717747>.

37. Yue, J., et al., *The scale and evolutionary significance of horizontal gene transfer in the choanoflagellate Monosiga brevicollis.* BMC Genomics, 2013. **14**: p. 729. <http://www.ncbi.nlm.nih.gov/pubmed/24156600>.

38. Tucker, R.P., *Horizontal gene transfer in choanoflagellates.* J Exp Zool B Mol Dev Evol, 2013. **320**(1): p. 1-9. <http://www.ncbi.nlm.nih.gov/pubmed/22997182>.

39. Sun, G., et al., *Algal genes in the closest relatives of animals.* Mol Biol Evol, 2010. **27**(12): p. 2879-89. <http://www.ncbi.nlm.nih.gov/pubmed/20627874>.

40. Alegado, R.A. and N. King, *Bacterial Influences on Animal Origins.* Cold Spring Harb Perspect Biol, 2014. <http://www.ncbi.nlm.nih.gov/pubmed/25280764>.

41. Boto, L., *Horizontal gene transfer in the acquisition of novel traits by metazoans.* Proc Biol Sci, 2014. **281**(1777): p. 20132450. <http://www.ncbi.nlm.nih.gov/pubmed/24403327>.

42. Dunning Hotopp, J.C., *Horizontal gene transfer between bacteria and animals.* Trends Genet, 2011. **27**(4): p. 157-63. <http://www.ncbi.nlm.nih.gov/pubmed/21334091>.

43. Srivastava, M., et al., *The Amphimedon queenslandica genome and the evolution of animal complexity.* Nature, 2010. **466**(7307): p. 720-6. <http://www.ncbi.nlm.nih.gov/pubmed/20686567>.

44. Conaco, C., et al., *Detection of Prokaryotic Genes in the Amphimedon queenslandica Genome.* PLoS One, 2016. **11**(3): p. e0151092. <http://www.ncbi.nlm.nih.gov/pubmed/26959231>.

45. Boschetti, C., et al., *Biochemical diversification through foreign gene expression in bdelloid rotifers.* PLoS Genet, 2012. **8**(11): p. e1003035. <http://www.ncbi.nlm.nih.gov/pubmed/23166508>.

46. Eyres, I., et al., *Horizontal gene transfer in bdelloid rotifers is ancient, ongoing and more frequent in species from desiccating habitats.* BMC Biol, 2015. **13**(1): p. 90. <http://www.ncbi.nlm.nih.gov/pubmed/26537913>.

47. Gladyshev, E.A., M. Meselson, and I.R. Arkhipova, *Massive horizontal gene transfer in bdelloid rotifers.* Science, 2008. **320**(5880): p. 1210-3. <http://www.ncbi.nlm.nih.gov/pubmed/18511688>.

48. Szydlowski, L., et al., *Multiple horizontally acquired genes from fungal and prokaryotic donors encode cellulolytic enzymes in the bdelloid rotifer Adineta ricciae.* Gene, 2015. **566**(2): p. 125-37. <http://www.ncbi.nlm.nih.gov/pubmed/25863176>.

49. Jackson, D.J., et al., *A horizontal gene transfer supported the evolution of an early metazoan biomineralization strategy.* BMC Evol Biol, 2011. **11**: p. 238. <http://www.ncbi.nlm.nih.gov/pubmed/21838889>.

50. Starcevic, A., et al., *Enzymes of the shikimic acid pathway encoded in the genome of a basal metazoan, Nematostella vectensis, have microbial origins.* Proc Natl Acad Sci U S A, 2008. **105**(7): p. 2533-7. <http://www.ncbi.nlm.nih.gov/pubmed/18268342>.

51. Kondrashov, F.A., et al., *Evolution of glyoxylate cycle enzymes in Metazoa: evidence of multiple horizontal transfer events and pseudogene formation.* Biol Direct, 2006. **1**: p. 31. <http://www.ncbi.nlm.nih.gov/pubmed/17059607>.

52. Chapman, J.A., et al., *The dynamic genome of Hydra.* Nature, 2010. **464**(7288): p. 592-6. <http://www.ncbi.nlm.nih.gov/pubmed/20228792>.

53. McNulty, S.N., et al., *Transcriptomic and proteomic analyses of a Wolbachia-free filarial parasite provide evidence of trans-kingdom horizontal gene transfer.* PLoS One, 2012. **7**(9): p. e45777. <http://www.ncbi.nlm.nih.gov/pubmed/23049857>.

54. McNulty, S.N., et al., *Endosymbiont DNA in endobacteria-free filarial nematodes indicates ancient horizontal genetic transfer.* PLoS One, 2010. **5**(6): p. e11029. <http://www.ncbi.nlm.nih.gov/pubmed/20543958>.

55. Haegeman, A., J.T. Jones, and E.G. Danchin, *Horizontal gene transfer in nematodes: a catalyst for plant parasitism?* Mol Plant Microbe Interact, 2011. **24**(8): p. 879-87. <http://www.ncbi.nlm.nih.gov/pubmed/21539433>.

56. Mayer, W.E., et al., *Horizontal gene transfer of microbial cellulases into nematode genomes is associated with functional assimilation and gene turnover.* BMC Evol Biol, 2011. **11**: p. 13. <http://www.ncbi.nlm.nih.gov/pubmed/21232122>.

57. Mitreva, M., G. Smant, and J. Helder, *Role of horizontal gene transfer in the evolution of plant parasitism among nematodes.* Methods Mol Biol, 2009. **532**: p. 517-35. <http://www.ncbi.nlm.nih.gov/pubmed/19271205>.

58. Scholl, E.H. and D.M. Bird, *Computational and phylogenetic validation of nematode horizontal gene transfer.* BMC Biol, 2011. **9**: p. 9. <http://www.ncbi.nlm.nih.gov/pubmed/21342537>.

59. Paganini, J., et al., *Contribution of lateral gene transfers to the genome composition and parasitic ability of root-knot nematodes.* PLoS One, 2012. **7**(11): p. e50875. <http://www.ncbi.nlm.nih.gov/pubmed/23226415>.

60. Bird, D.M., C.H. Opperman, and K.G. Davies, *Interactions between bacteria and plant-parasitic nematodes: now and then.* Int J Parasitol, 2003. **33**(11): p. 1269-76. <http://www.ncbi.nlm.nih.gov/pubmed/13678641>.

61. Craig, J.P., et al., *Evidence for horizontally transferred genes involved in the biosynthesis of vitamin B(1), B(5), and B(7) in Heterodera glycines.* J Nematol, 2009. **41**(4): p. 281-90. <http://www.ncbi.nlm.nih.gov/pubmed/22736827>.

62. Eves-van den Akker, S., et al., *The genome of the yellow potato cyst nematode, Globodera rostochiensis, reveals insights into the basis of parasitism and virulence.* Genome Biol, 2016. **17**(1): p. 124. <http://www.ncbi.nlm.nih.gov/pubmed/27286965>.

63. Craig, J.P., et al., *Analysis of a horizontally transferred pathway involved in vitamin B6 biosynthesis from the soybean cyst nematode Heterodera glycines.* Mol Biol Evol, 2008. **25**(10): p. 2085-98. <http://www.ncbi.nlm.nih.gov/pubmed/18586696>.

64. Opperman, C.H., et al., *Sequence and genetic map of Meloidogyne hapla: A compact nematode genome for plant parasitism.* Proc Natl Acad Sci U S A, 2008. **105**(39): p. 14802-7. <http://www.ncbi.nlm.nih.gov/pubmed/18809916>.

65. Danchin, E.G., *What Nematode genomes tell us about the importance of horizontal gene transfers in the evolutionary history of animals.* Mob Genet Elements, 2011. **1**(4): p. 269-273. <http://www.ncbi.nlm.nih.gov/pubmed/22545237>.

66. Shelomi, M., et al., *Horizontal Gene Transfer of Pectinases from Bacteria Preceded the Diversification of Stick and Leaf Insects.* Sci Rep, 2016. **6**: p. 26388. <http://www.ncbi.nlm.nih.gov/pubmed/27210832>.

67. Wybouw, N., et al., *A gene horizontally transferred from bacteria protects arthropods from host plant cyanide poisoning.* Elife, 2014. **3**: p. e02365. <http://www.ncbi.nlm.nih.gov/pubmed/24843024>.

68. McKenna, D.D., et al., *Genome of the Asian longhorned beetle (Anoplophora glabripennis), a globally significant invasive species, reveals key functional and evolutionary innovations at the beetle-plant interface.* Genome Biol, 2016. **17**(1): p. 227. <http://www.ncbi.nlm.nih.gov/pubmed/27832824>.

69. Wybouw, N., et al., *Horizontal Gene Transfer Contributes to the Evolution of Arthropod Herbivory.* Genome Biol Evol, 2016. **8**(6): p. 1785-801. <http://www.ncbi.nlm.nih.gov/pubmed/27307274>.

70. Acuna, R., et al., *Adaptive horizontal transfer of a bacterial gene to an invasive insect pest of coffee.* Proc Natl Acad Sci U S A, 2012. **109**(11): p. 4197-202. <http://www.ncbi.nlm.nih.gov/pubmed/22371593>.

71. Li, Z.W., et al., *Pathogen-origin horizontally transferred genes contribute to the evolution of Lepidopteran insects.* BMC Evol Biol, 2011. **11**: p. 356. <http://www.ncbi.nlm.nih.gov/pubmed/22151541>.

72. Crisp, A., et al., *Expression of multiple horizontally acquired genes is a hallmark of both vertebrate and invertebrate genomes.* Genome Biol, 2015. **16**(1): p. 50. <http://www.ncbi.nlm.nih.gov/pubmed/25785303>.

73. Boothby, T.C., et al., *Evidence for extensive horizontal gene transfer from the draft genome of a tardigrade.* Proc Natl Acad Sci U S A, 2015. <http://www.ncbi.nlm.nih.gov/pubmed/26598659>.

74. Nakashima, K., et al., *The evolutionary origin of animal cellulose synthase.* Dev Genes Evol, 2004. **214**(2): p. 81-8. <http://www.ncbi.nlm.nih.gov/pubmed/14740209>.

75. Moran, N.A. and T. Jarvik, *Lateral transfer of genes from fungi underlies carotenoid production in aphids.* Science, 2010. **328**(5978): p. 624-7. <http://www.ncbi.nlm.nih.gov/pubmed/20431015>.

76. Altincicek, B., J.L. Kovacs, and N.M. Gerardo, *Horizontally transferred fungal carotenoid genes in the two-spotted spider mite Tetranychus urticae.* Biol Lett, 2012. **8**(2): p. 253-7. <http://www.ncbi.nlm.nih.gov/pubmed/21920958>.

77. Wybouw, N., et al., *A horizontally transferred cyanase gene in the spider mite Tetranychus urticae is involved in cyanate metabolism and is differentially expressed upon host plant change.* Insect Biochem Mol Biol, 2012. **42**(12): p. 881-9. <http://www.ncbi.nlm.nih.gov/pubmed/22960016>.

78. Ni, T., et al., *Ancient gene transfer from algae to animals: mechanisms and evolutionary significance.* BMC Evol Biol, 2012. **12**: p. 83. <http://www.ncbi.nlm.nih.gov/pubmed/22690978>.

79. Selman, M., et al., *Acquisition of an animal gene by microsporidian intracellular parasites.* Curr Biol, 2011. **21**(15): p. R576-7. <http://www.ncbi.nlm.nih.gov/pubmed/21820617>.

80. Pombert, J.F., et al., *Gain and loss of multiple functionally related, horizontally transferred genes in the reduced genomes of two microsporidian parasites.* Proc Natl Acad Sci U S A, 2012. **109**(31): p. 12638-43. <http://www.ncbi.nlm.nih.gov/pubmed/22802648>.

81. Yue, J., et al., *Widespread impact of horizontal gene transfer on plant colonization of land.* Nat Commun, 2012. **3**: p. 1152. <http://www.ncbi.nlm.nih.gov/pubmed/23093189>.

82. Hoang, Q.T., et al., *An actinoporin plays a key role in water stress in the moss Physcomitrella patens.* New Phytol, 2009. **184**(2): p. 502-10. <http://www.ncbi.nlm.nih.gov/pubmed/19674339>.

83. Emiliani, G., et al., *A horizontal gene transfer at the origin of phenylpropanoid metabolism: a key adaptation of plants to land.* Biol Direct, 2009. **4**: p. 7. <http://www.ncbi.nlm.nih.gov/pubmed/19220881>.

84. Yang, Z., et al., *Ancient horizontal transfer of transaldolase-like protein gene and its role in plant vascular development.* New Phytol, 2015. **206**(2): p. 807-16. <http://www.ncbi.nlm.nih.gov/pubmed/25420550>.

85. Richards, T.A., et al., *Phylogenomic analysis demonstrates a pattern of rare and ancient horizontal gene transfer between plants and fungi.* Plant Cell, 2009. **21**(7): p. 1897-911. <http://www.ncbi.nlm.nih.gov/pubmed/19584142>.

86. Nikolaidis, N., N. Doran, and D.J. Cosgrove, *Plant expansins in bacteria and fungi: evolution by horizontal gene transfer and independent domain fusion.* Mol Biol Evol, 2014. **31**(2): p. 376-86. <http://www.ncbi.nlm.nih.gov/pubmed/24150040>.

87. Gao, C., et al., *Horizontal gene transfer in plants.* Funct Integr Genomics, 2014. **14**(1): p. 23-9. <http://www.ncbi.nlm.nih.gov/pubmed/24132513>.

88. Bock, R., *The give-and-take of DNA: horizontal gene transfer in plants.* Trends Plant Sci, 2009. <http://www.ncbi.nlm.nih.gov/pubmed/19910236>.

89. Mower, J.P., et al., *Horizontal acquisition of multiple mitochondrial genes from a parasitic plant followed by gene conversion with host mitochondrial genes.* BMC Biol, 2010. **8**: p. 150. <http://www.ncbi.nlm.nih.gov/pubmed/21176201>.

90. Mower, J.P., et al., *Plant genetics: gene transfer from parasitic to host plants.* Nature, 2004. **432**(7014): p. 165-6. <http://www.ncbi.nlm.nih.gov/pubmed/15538356>.

91. Xi, Z., et al., *Horizontal transfer of expressed genes in a parasitic flowering plant.* BMC Genomics, 2012. **13**(1): p. 227. <http://www.ncbi.nlm.nih.gov/pubmed/22681756>.

92. Davis, C.C. and K.J. Wurdack, *Host-to-parasite gene transfer in flowering plants: phylogenetic evidence from Malpighiales.* Science, 2004. **305**(5684): p. 676-8. <http://www.ncbi.nlm.nih.gov/pubmed/15256617>.

93. Xi, Z., et al., *Massive mitochondrial gene transfer in a parasitic flowering plant clade.* PLoS Genet, 2013. **9**(2): p. e1003265. <http://www.ncbi.nlm.nih.gov/pubmed/23459037>.

94. Mahelka, V., et al., *Multiple horizontal transfers of nuclear ribosomal genes between phylogenetically distinct grass lineages.* Proc Natl Acad Sci U S A, 2017. **114**(7): p. 1726-1731. <http://www.ncbi.nlm.nih.gov/pubmed/28137844>.

95. Li, F.W., et al., *Horizontal transfer of an adaptive chimeric photoreceptor from bryophytes to ferns.* Proc Natl Acad Sci U S A, 2014. **111**(18): p. 6672-7. <http://www.ncbi.nlm.nih.gov/pubmed/24733898>.

96. Christin, P.A., et al., *Adaptive evolution of C(4) photosynthesis through recurrent lateral gene transfer.* Curr Biol, 2012. **22**(5): p. 445-9. <http://www.ncbi.nlm.nih.gov/pubmed/22342748>.

97. Gandini, C.L. and M.V. Sanchez-Puerta, *Foreign Plastid Sequences in Plant Mitochondria are Frequently Acquired Via Mitochondrion-to-Mitochondrion Horizontal Transfer.* Sci Rep, 2017. **7**: p. 43402. <http://www.ncbi.nlm.nih.gov/pubmed/28262720>.

98. Bergthorsson, U., et al., *Widespread horizontal transfer of mitochondrial genes in flowering plants.* Nature, 2003. **424**(6945): p. 197-201. <http://www.ncbi.nlm.nih.gov/pubmed/12853958>.

99. Bergthorsson, U., et al., *Massive horizontal transfer of mitochondrial genes from diverse land plant donors to the basal angiosperm Amborella.* Proc Natl Acad Sci U S A, 2004. **101**(51): p. 17747-52. <http://www.ncbi.nlm.nih.gov/pubmed/15598737>.

100. Rice, D.W., et al., *Horizontal transfer of entire genomes via mitochondrial fusion in the angiosperm Amborella.* Science, 2013. **342**(6165): p. 1468-73. <http://www.ncbi.nlm.nih.gov/pubmed/24357311>.

101. Zhang, Y., et al., *Evolution of a horizontally acquired legume gene, albumin 1, in the parasitic plant Phelipanche aegyptiaca and related species.* BMC Evol Biol, 2013. **13**: p. 48. <http://www.ncbi.nlm.nih.gov/pubmed/23425243>.

102. Zhang, D., et al., *Root parasitic plant Orobanche aegyptiaca and shoot parasitic plant Cuscuta australis obtained Brassicaceae-specific strictosidine synthase-like genes by horizontal gene transfer.* BMC Plant Biol, 2014. **14**: p. 19. <http://www.ncbi.nlm.nih.gov/pubmed/24411025>.

103. Graham, L.A., et al., *Lateral transfer of a lectin-like antifreeze protein gene in fishes.* PLoS One, 2008. **3**(7): p. e2616. <http://www.ncbi.nlm.nih.gov/pubmed/18612417>.

104. Graham, L.A., et al., *Smelt was the likely beneficiary of an antifreeze gene laterally transferred between fishes.* BMC Evol Biol, 2012. **12**: p. 190. <http://www.ncbi.nlm.nih.gov/pubmed/23009612>.

105. Doolittle, R.F., et al., *A naturally occurring horizontal gene transfer from a eukaryote to a prokaryote.* J Mol Evol, 1990. **31**(5): p. 383-8. <http://www.ncbi.nlm.nih.gov/pubmed/2124629>.

106. Lurie-Weinberger, M.N., et al., *The origins of eukaryotic-like proteins in Legionella pneumophila.* Int J Med Microbiol, 2010. **300**(7): p. 470-81. <http://www.ncbi.nlm.nih.gov/pubmed/20537944>.

107. de la Casa-Esperon, E., *Horizontal transfer and the evolution of host-pathogen interactions.* Int J Evol Biol, 2012. **2012**: p. 679045. <http://www.ncbi.nlm.nih.gov/pubmed/23227424>.

108. Gomez-Valero, L., et al., *Comparative and functional genomics of legionella identified eukaryotic like proteins as key players in host-pathogen interactions.* Front Microbiol, 2011. **2**: p. 208. <http://www.ncbi.nlm.nih.gov/pubmed/22059087>.

109. Burstein, D., et al., *Genomic analysis of 38 Legionella species identifies large and diverse effector repertoires.* Nat Genet, 2016. **48**(2): p. 167-75. <http://www.ncbi.nlm.nih.gov/pubmed/26752266>.

110. Rolando, M. and C. Buchrieser, *Legionella pneumophila type IV effectors hijack the transcription and translation machinery of the host cell.* Trends Cell Biol, 2014. **24**(12): p. 771-8. <http://www.ncbi.nlm.nih.gov/pubmed/25012125>.

111. Filee, J., et al., *Evolution of DNA polymerase families: evidences for multiple gene exchange between cellular and viral proteins.* J Mol Evol, 2002. **54**(6): p. 763-73. <http://www.ncbi.nlm.nih.gov/pubmed/12029358>.

112. Shutt, T.E. and M.W. Gray, *Bacteriophage origins of mitochondrial replication and transcription proteins.* Trends Genet, 2006. **22**(2): p. 90-5. <http://www.ncbi.nlm.nih.gov/pubmed/16364493>.

113. Filee, J. and P. Forterre, *Viral proteins functioning in organelles: a cryptic origin?* Trends Microbiol, 2005. **13**(11): p. 510-3. <http://www.ncbi.nlm.nih.gov/pubmed/16157484>.

114. Liu, H., et al., *Widespread horizontal gene transfer from double-stranded RNA viruses to eukaryotic nuclear genomes.* J Virol, 2010. **84**(22): p. 11876-87. <http://www.ncbi.nlm.nih.gov/pubmed/20810725>.

115. Liu, H., et al., *Widespread horizontal gene transfer from circular single-stranded DNA viruses to eukaryotic genomes.* BMC Evol Biol, 2011. **11**: p. 276. <http://www.ncbi.nlm.nih.gov/pubmed/21943216>.

116. Hendrix, R.W., et al., *The origins and ongoing evolution of viruses.* Trends Microbiol, 2000. **8**(11): p. 504-8. <http://www.ncbi.nlm.nih.gov/pubmed/11121760>.

117. Filee, J., N. Pouget, and M. Chandler, *Phylogenetic evidence for extensive lateral acquisition of cellular genes by Nucleocytoplasmic large DNA viruses.* BMC Evol Biol, 2008. **8**: p. 320. <http://www.ncbi.nlm.nih.gov/pubmed/19036122>.

118. Tidona, C.A. and G. Darai, *Iridovirus homologues of cellular genes--implications for the molecular evolution of large DNA viruses.* Virus Genes, 2000. **21**(1-2): p. 77-81. <http://www.ncbi.nlm.nih.gov/pubmed/11022791>.

119. Iyer, L.M., et al., *Evolutionary genomics of nucleo-cytoplasmic large DNA viruses.* Virus Res, 2006. **117**(1): p. 156-84. <http://www.ncbi.nlm.nih.gov/pubmed/16494962>.

120. Colson, P. and D. Raoult, *Gene repertoire of amoeba-associated giant viruses.* Intervirology, 2010. **53**(5): p. 330-43. <http://www.ncbi.nlm.nih.gov/pubmed/20551685>.

121. Aherfi, S., et al., *Giant Viruses of Amoebas: An Update.* Front Microbiol, 2016. **7**: p. 349. <http://www.ncbi.nlm.nih.gov/pubmed/27047465>.

122. Hughes, A.L. and R. Friedman, *Poxvirus genome evolution by gene gain and loss.* Mol Phylogenet Evol, 2005. **35**(1): p. 186-95. <http://www.ncbi.nlm.nih.gov/pubmed/15737590>.

123. Filee, J., P. Siguier, and M. Chandler, *I am what I eat and I eat what I am: acquisition of bacterial genes by giant viruses.* Trends Genet, 2007. **23**(1): p. 10-5. <http://www.ncbi.nlm.nih.gov/pubmed/17109990>.

124. Becker, Y., *Evolution of viruses by acquisition of cellular RNA or DNA nucleotide sequences and genes: an introduction.* Virus Genes, 2000. **21**(1-2): p. 7-12. <http://www.ncbi.nlm.nih.gov/pubmed/11022785>.

125. Hughes, A.L. and R. Friedman, *Genome-wide survey for genes horizontally transferred from cellular organisms to baculoviruses.* Mol Biol Evol, 2003. **20**(6): p. 979-87. <http://www.ncbi.nlm.nih.gov/pubmed/12716988>.

126. Weynberg, K.D., M.J. Allen, and W.H. Wilson, *Marine Prasinoviruses and Their Tiny Plankton Hosts: A Review.* Viruses, 2017. **9**(3). <http://www.ncbi.nlm.nih.gov/pubmed/28294997>.
