# Supplementary material for: Living Organisms Author Their Read-Write Genomes in Evolution
Source: Biology (Basel). 2017 Dec 6;6(4):42. doi: 10.3390/biology6040042 (PMC5745447; doi:10.3390/biology6040042)
Supplement: Supplementary file 1 [file biology-06-00042-s001.tgz › biology-224185-supplementary & PUBMED links/biology-224185.zip/Shapiro - Living Organisms Author Their Read-Write Genomes in Evolution - Supplemental Material.Renumbered and Approved + PUBMED links/Supplementary Table S8 Highly cited protein domain search terms in the PubMed database.docx]

| **Supplementary Table 8. Highly cited protein domain search terms in the PubMed database** | | |
| --- | --- | --- |
| **Domain** | **Function** | **Reference** |
| Kinase domain | Catalytic (phosphorylation) | [[1](#_ENREF_1)] |
| Transmembrane domain | Subcellular localization | [[2](#_ENREF_2)] |
| PDZ domain | Protein-protein interaction module | [[3](#_ENREF_3), [4](#_ENREF_4)] |
| SH3 domain | Proline-rich protein-protein interaction module | [[5](#_ENREF_5), [6](#_ENREF_6)] |
| DNA binding domain | Molecular recognition | [[7-9](#_ENREF_7)] |
| PAS domain | Sequence-specific DNA binding | [[10](#_ENREF_10)] |
| WW domain | Protein-protein interaction module | [[11](#_ENREF_11)] |
| PH domain | Phosphatidylinositol binding in membranes | [[12](#_ENREF_12)] |
| SET domain | Binding methylated DNA | [[13](#_ENREF_13), [14](#_ENREF_14)] |
| SH2 domain | Binding proteins containing phosphor-tyrosine residues | [[15](#_ENREF_15), [16](#_ENREF_16)] |

REFERENCES

1. Deshmukh, K., K. Anamika, and N. Srinivasan, *Evolution of domain combinations in protein kinases and its implications for functional diversity.* Prog Biophys Mol Biol, 2010. **102**(1): p. 1-15. <http://www.ncbi.nlm.nih.gov/pubmed/20026163>.

2. Mittal, A. and S. Singh, *Insights into eukaryotic evolution from transmembrane domain lengths.* J Biomol Struct Dyn, 2017: p. 1-220. <http://www.ncbi.nlm.nih.gov/pubmed/28641482>.

3. Kim, J., et al., *Rewiring of PDZ domain-ligand interaction network contributed to eukaryotic evolution.* PLoS Genet, 2012. **8**(2): p. e1002510. <http://www.ncbi.nlm.nih.gov/pubmed/22346764>.

4. Sakarya, O., et al., *Evolutionary expansion and specialization of the PDZ domains.* Mol Biol Evol, 2010. **27**(5): p. 1058-69. <http://www.ncbi.nlm.nih.gov/pubmed/20026484>.

5. Tatarova, Z., et al., *SH3 Domain Tyrosine Phosphorylation - Sites, Role and Evolution.* PLoS One, 2012. **7**(5): p. e36310. <http://www.ncbi.nlm.nih.gov/pubmed/22615764>.

6. Tonikian, R., et al., *Bayesian Modeling of the Yeast SH3 Domain Interactome Predicts Spatiotemporal Dynamics of Endocytosis Proteins.* PLoS Biol, 2009. **7**(10): p. e1000218. .

7. Lehti-Shiu, M.D., et al., *Diversity, expansion, and evolutionary novelty of plant DNA-binding transcription factor families.* Biochim Biophys Acta, 2017. **1860**(1): p. 3-20. <http://www.ncbi.nlm.nih.gov/pubmed/27522016>.

8. Yamasaki, K., et al., *DNA-binding domains of plant-specific transcription factors: structure, function, and evolution.* Trends Plant Sci, 2013. **18**(5): p. 267-76. <http://www.ncbi.nlm.nih.gov/pubmed/23040085>.

9. Cheatle Jarvela, A.M. and V.F. Hinman, *Evolution of transcription factor function as a mechanism for changing metazoan developmental gene regulatory networks.* Evodevo, 2015. **6**(1): p. 3. <http://www.ncbi.nlm.nih.gov/pubmed/25685316>.

10. Wu, D. and F. Rastinejad, *Structural characterization of mammalian bHLH-PAS transcription factors.* Curr Opin Struct Biol, 2016. **43**: p. 1-9. <http://www.ncbi.nlm.nih.gov/pubmed/27721191>.

11. Dodson, E.J., et al., *Versatile communication strategies among tandem WW domain repeats.* Exp Biol Med (Maywood), 2015. **240**(3): p. 351-60. <http://www.ncbi.nlm.nih.gov/pubmed/25710931>.

12. Kume, A., et al., *The Pleckstrin Homology Domain of Diacylglycerol Kinase eta Strongly and Selectively Binds to Phosphatidylinositol 4,5-Bisphosphate.* J Biol Chem, 2016. **291**(15): p. 8150-61. <http://www.ncbi.nlm.nih.gov/pubmed/26887948>.

13. Shimbo, T. and P.A. Wade, *Proteins That Read DNA Methylation.* Adv Exp Med Biol, 2016. **945**: p. 303-320. <http://www.ncbi.nlm.nih.gov/pubmed/27826844>.

14. Zhang, L. and H. Ma, *Complex evolutionary history and diverse domain organization of SET proteins suggest divergent regulatory interactions.* New Phytol, 2012. **195**(1): p. 248-63. <http://www.ncbi.nlm.nih.gov/pubmed/22510098>.

15. Reebye, V., et al., *A perspective on non-catalytic Src homology (SH) adaptor signalling proteins.* Cell Signal, 2012. **24**(2): p. 388-92. <http://www.ncbi.nlm.nih.gov/pubmed/22024281>.

16. Liu, B.A., *Classification and Lineage Tracing of SH2 Domains Throughout Eukaryotes.* Methods Mol Biol, 2017. **1555**: p. 59-75. <http://www.ncbi.nlm.nih.gov/pubmed/28092027>.
