# Supplementary material for: Living Organisms Author Their Read-Write Genomes in Evolution
Source: Biology (Basel). 2017 Dec 6;6(4):42. doi: 10.3390/biology6040042 (PMC5745447; doi:10.3390/biology6040042)
Supplement: Supplementary file 1 [file biology-06-00042-s001.tgz › biology-224185-supplementary & PUBMED links/biology-224185.zip/Shapiro - Living Organisms Author Their Read-Write Genomes in Evolution - Supplemental Material.Renumbered and Approved + PUBMED links/Supplementary Table S9 Selected Reports of Exon Rearrangements and Retroposition.docx]

| **Supplementary Table 9 Selected Reports of Exon Rearrangements and Retroposition by Mobile DNA element Activity** | |
| --- | --- |
| **Rearrangement** | **References** |
| Group II intron retrotransposition and exon shuffling in yeast | [[1](#_ENREF_1)] |
| Chimeric LINE-mediated retrogene in rice blast fungus *Magnaporthe grisea* | [[2](#_ENREF_2)] |
| DNA “splicing” by transposase-related excision functions in ciliated protists | [[3](#_ENREF_3)] |
| Exon shuffling by DNA transposons in plants (Pack-MULEs, etc.) | [[4-7](#_ENREF_4)] |
| LINE 1-mediated duplications in dicots | [[8](#_ENREF_8)] |
| CACTA transposon-mobilized exon in beans | [[9](#_ENREF_9)] |
| Helitron-mediated exon shuffling in maize | [[10](#_ENREF_10), [11](#_ENREF_11)] |
| Chimeric coding loci mediated by Ac/Ds transposons in maize | [[12](#_ENREF_12), [13](#_ENREF_13)] |
| Retrotransposon-mediated exon shuffling in maize | [[14](#_ENREF_14)] |
| Retrotransposon-mediated exon shuffling in *Medicago sativa* | [[15](#_ENREF_15)] |
| Helitron-mediated exon shuffling in *Lepidoptera* | [[16](#_ENREF_16)] |
| FB transposon-mediated exon shuffling in *Drosophila* | [[17](#_ENREF_17)] |
| Retrotransduction by non-LTR retrotransposons (LINEs and SINEs) in *Drosophila* | [[18](#_ENREF_18)] |
| Exon shuffling by retrotransposition in mammals | [[19](#_ENREF_19), [20](#_ENREF_20)] |
| Retrotransposon exon shuffling in primates | [[21](#_ENREF_21)] |
| TRIM5-Cyclophilin A (TRIMCyp) fusion by retrotransposition in tree shrews and owl monkeys | [[13](#_ENREF_13), [22](#_ENREF_22)] |
| Exon shuffling by retrotransposition in humans | [[23](#_ENREF_23)] |
| Retrotransposed copies in the human genome | [[24-26](#_ENREF_24)] |
| L1/LINE mediated retrotransduction in humans | [[27](#_ENREF_27), [28](#_ENREF_28)] |
| *Alu* SINE mediated retrotransduction in humans | [[29-31](#_ENREF_29)] |
| *SVA* SINE mediated retrotransduction in humans | [[32](#_ENREF_32)] |
| *SVA* SINE fusion with exon in humans | [[33](#_ENREF_33)] |
| Human genome chimeric “retrogenes,” by LINE-mediated template switches at the RNA level | [[34-36](#_ENREF_34)] |
| Chimeric human retrotransposon | [[33](#_ENREF_33)] |

REFERENCES

1. Hiller, R., et al., *Transposition and exon shuffling by group II intron RNA molecules in pieces.* J Mol Biol, 2000. **297**(2): p. 301-8. <http://www.ncbi.nlm.nih.gov/pubmed/10715202>.

2. Gogvadze, E., et al., *Tripartite chimeric pseudogene from the genome of rice blast fungus Magnaporthe grisea suggests double template jumps during long interspersed nuclear element (LINE) reverse transcription.* BMC Genomics, 2007. **8**: p. 360. <http://www.ncbi.nlm.nih.gov/pubmed/17922896>.

3. Chen, X., et al., *Combinatorial DNA Rearrangement Facilitates the Origin of New Genes in Ciliates.* Genome Biol Evol, 2015. **7**(10): p. 2859-70. <http://www.ncbi.nlm.nih.gov/pubmed/26338187>.

4. Dooner, H.K. and C.F. Weil, *Give-and-take: interactions between DNA transposons and their host plant genomes.* Curr Opin Genet Dev, 2007. **17**(6): p. 486-92. <http://www.ncbi.nlm.nih.gov/pubmed/17919898>.

5. Jiang, N., et al., *Pack-MULE transposable elements mediate gene evolution in plants.* Nature, 2004. **431**(7008): p. 569-73. <http://www.ncbi.nlm.nih.gov/pubmed/15457261>.

6. Lisch, D., *Pack-MULEs: theft on a massive scale.* Bioessays, 2005. **27**(4): p. 353-5. <http://www.ncbi.nlm.nih.gov/pubmed/15770680>.

7. Jiang, N., et al., *Pack-Mutator-like transposable elements (Pack-MULEs) induce directional modification of genes through biased insertion and DNA acquisition.* Proc Natl Acad Sci U S A, 2011. **108**(4): p. 1537-42. <http://www.ncbi.nlm.nih.gov/pubmed/21220310>.

8. Zhu, Z., et al., *LINE-1-like retrotransposons contribute to RNA-based gene duplication in dicots.* Sci Rep, 2016. **6**: p. 24755. <http://www.ncbi.nlm.nih.gov/pubmed/27098918>.

9. Zabala, G. and L. Vodkin, *Novel exon combinations generated by alternative splicing of gene fragments mobilized by a CACTA transposon in Glycine max.* BMC Plant Biol, 2007. **7**: p. 38. <http://www.ncbi.nlm.nih.gov/pubmed/17629935>.

10. Morgante, M., et al., *Gene duplication and exon shuffling by helitron-like transposons generate intraspecies diversity in maize.* Nat Genet, 2005. **37**(9): p. 997-1002. <http://www.ncbi.nlm.nih.gov/pubmed/16056225>.

11. Lai, J., et al., *Gene movement by Helitron transposons contributes to the haplotype variability of maize.* Proc Natl Acad Sci U S A, 2005. **102**(25): p. 9068-73. <http://www.ncbi.nlm.nih.gov/pubmed/15951422>.

12. Wang, D., et al., *Alternative Transposition Generates New Chimeric Genes and Segmental Duplications at the Maize p1 Locus.* Genetics, 2015. **201**(3): p. 925-35. <http://www.ncbi.nlm.nih.gov/pubmed/26434719>.

13. Zhang, J., F. Zhang, and T. Peterson, *Transposition of reversed Ac element ends generates novel chimeric genes in maize.* PLoS Genet, 2006. **2**(10): p. e164. <http://www.ncbi.nlm.nih.gov/pubmed/17029561>.

14. Elrouby, N. and T.E. Bureau, *Bs1, a new chimeric gene formed by retrotransposon-mediated exon shuffling in maize.* Plant Physiol, 2010. **153**(3): p. 1413-24. <http://www.ncbi.nlm.nih.gov/pubmed/20488894>.

15. Vegh, Z., et al., *The nucleotide sequence of a nodule-specific gene, Nms-25 of Medicago sativa: its primary evolution via exon-shuffling and retrotransposon-mediated DNA rearrangements.* Plant Mol Biol, 1990. **15**(2): p. 295-306. <http://www.ncbi.nlm.nih.gov/pubmed/1966488>.

16. Coates, B.S., et al., *Mobilizing the genome of Lepidoptera through novel sequence gains and end creation by non-autonomous Lep1 Helitrons.* DNA Res, 2012. **19**(1): p. 11-21. <http://www.ncbi.nlm.nih.gov/pubmed/22086996>.

17. Moschetti, R., et al., *FB elements can promote exon shuffling: a promoter-less white allele can be reactivated by FB mediated transposition in Drosophila melanogaster.* Mol Genet Genomics, 2004. **271**(4): p. 394-401. <http://www.ncbi.nlm.nih.gov/pubmed/15060822>.

18. Betrán, E., K. Thornton, and M. Long, *Retroposed new genes out of the X in Drosophila.* Genome Res, 2002. **12**: p. 1854-1859. <http://www.ncbi.nlm.nih.gov/pubmed/12466289>.

19. Dupuy, D., V.G. Duperat, and B. Arveiler, *SCAN domain-containing 2 gene (SCAND2) is a novel nuclear protein derived from the zinc finger family by exon shuffling.* Gene, 2002. **289**(1-2): p. 1-6. <http://www.ncbi.nlm.nih.gov/pubmed/12036577>.

20. Ding, W., et al., *L1 elements, processed pseudogenes and retrogenes in mammalian genomes.* IUBMB Life, 2006. **58**(12): p. 677-85. <http://www.ncbi.nlm.nih.gov/pubmed/17424906>.

21. Xing, J., et al., *Emergence of primate genes by retrotransposon-mediated sequence transduction.* Proc Natl Acad Sci U S A, 2006. **103**(47): p. 17608-13. <http://www.ncbi.nlm.nih.gov/pubmed/17101974>.

22. Sayah, D.M., et al., *Cyclophilin A retrotransposition into TRIM5 explains owl monkey resistance to HIV-1.* Nature, 2004. **430**(6999): p. 569-73. <http://www.ncbi.nlm.nih.gov/pubmed/15243629>.

23. Ohshima, K. and K. Igarashi, *Inference for the initial stage of domain shuffling: tracing the evolutionary fate of the PIPSL retrogene in hominoids.* Mol Biol Evol, 2010. **27**(11): p. 2522-33. <http://www.ncbi.nlm.nih.gov/pubmed/20525901>.

24. Vinckenbosch, N., I. Dupanloup, and H. Kaessmann, *Evolutionary fate of retroposed gene copies in the human genome.* Proc Natl Acad Sci U S A, 2006. **103**(9): p. 3220-5. <http://www.ncbi.nlm.nih.gov/pubmed/16492757>.

25. Marques, A.C., et al., *Emergence of young human genes after a burst of retroposition in primates.* PLoS Biol, 2005. **3**(11): p. e357. <http://www.ncbi.nlm.nih.gov/pubmed/16201836>.

26. Ohshima, K., et al., *Whole-genome screening indicates a possible burst of formation of processed pseudogenes and Alu repeats by particular L1 subfamilies in ancestral primates.* Genome Biol, 2003. **4**(11): p. R74. <http://www.ncbi.nlm.nih.gov/pubmed/14611660>.

27. Esnault, C., J. Maestre, and T. Heidmann, *Human LINE retrotransposons generate processed pseudogenes.* Nat Genet, 2000. **24**(4): p. 363-7. <http://www.ncbi.nlm.nih.gov/pubmed/10742098>.

28. Ejima, Y. and L. Yang, *Trans mobilization of genomic DNA as a mechanism for retrotransposon-mediated exon shuffling.* Hum Mol Genet, 2003. **12**(11): p. 1321-8. <http://www.ncbi.nlm.nih.gov/pubmed/12761047>.

29. Farre, D., P. Engel, and A. Angulo, *Novel Role of 3'UTR-Embedded Alu Elements as Facilitators of Processed Pseudogene Genesis and Host Gene Capture by Viral Genomes.* PLoS One, 2016. **11**(12): p. e0169196. <http://www.ncbi.nlm.nih.gov/pubmed/28033411>.

30. Hancks, D.C., et al., *Exon-trapping mediated by the human retrotransposon SVA.* Genome Res, 2009. **19**(11): p. 1983-91. <http://www.ncbi.nlm.nih.gov/pubmed/19635844>.

31. Volff, J.N. and J. Brosius, *Modern genomes with retro-look: retrotransposed elements, retroposition and the origin of new genes.* Genome Dyn, 2007. **3**: p. 175-90. <http://www.ncbi.nlm.nih.gov/pubmed/18753792>.

32. Taniguchi-Ikeda, M., et al., *Pathogenic exon-trapping by SVA retrotransposon and rescue in Fukuyama muscular dystrophy.* Nature, 2011. **478**(7367): p. 127-31. <http://www.ncbi.nlm.nih.gov/pubmed/21979053>.

33. Bantysh, O.B. and A.A. Buzdin, *Novel Family of Human Transposable Elements Formed Due to Fusion of the First Exon of Gene MAST2 with Retrotransposon SVA.* Biochemistry (Mosc), 2009. **74**(12): p. 1393-9. <http://www.ncbi.nlm.nih.gov/pubmed/19961423>.

34. Buzdin, A., et al., *The human genome contains many types of chimeric retrogenes generated through in vivo RNA recombination.* Nucleic Acids Res, 2003. **31**(15): p. 4385-90. <http://www.ncbi.nlm.nih.gov/pubmed/12888497>.

35. Buzdin, A.A., *Retroelements and formation of chimeric retrogenes.* Cell Mol Life Sci, 2004. **61**(16): p. 2046-59. <http://www.ncbi.nlm.nih.gov/pubmed/15316654>.

36. Buzdin, A., E. Gogvadze, and M.H. Lebrun, *Chimeric retrogenes suggest a role for the nucleolus in LINE amplification.* FEBS Lett, 2007. **581**(16): p. 2877-82. <http://www.ncbi.nlm.nih.gov/pubmed/17560999>.
